# Yuccalechins A–C from the *Yucca schidigera* Roezl ex Ortgies Bark: Elucidation of the Relative and Absolute Configurations of Three New Spirobiflavonoids and Their Cholinesterase Inhibitory Activities

**DOI:** 10.3390/molecules24224162

**Published:** 2019-11-16

**Authors:** Łukasz Pecio, Mostafa Alilou, Solomiia Kozachok, Ilkay Erdogan Orhan, Gokcen Eren, Fatma Sezer Senol Deniz, Hermann Stuppner, Wiesław Oleszek

**Affiliations:** 1 Department of Biochemistry and Crop Quality, Institute of Soil Science and Plant Cultivation-State Research Institute, Czartoryskich 8, 24-100 Puławy, Poland; skozachok@iung.pulawy.pl (S.K.); wieslaw.oleszek@iung.pulawy.pl (W.O.); 2 Institute of Pharmacy/Pharmacognosy, Center for Molecular Biosciences Innsbruck, University of Innsbruck, Innrain 80/82, Innsbruck 6020, Austria; hermann.stuppner@uibk.ac.at; 3 Department of Pharmacognosy, Faculty of Pharmacy, Gazi University, 06330 Ankara, Turkey; iorhan@gazi.edu.tr (I.E.O.); fssenol@gazi.edu.tr (F.S.S.D.); 4 Department of Pharmaceutical Chemistry, Faculty of Pharmacy, Gazi University, 06330 Ankara, Turkey; gokcene@gazi.edu.tr

**Keywords:** *Yucca schidigera*, Asparagaceae, spirobiflavonoid, absolute configuration, DP4+, ECD, Alzheimer’s disease

## Abstract

The ethyl acetate fraction of the methanolic extract of *Yucca schidigera* Roezl ex Ortgies bark exhibited moderate acetylcholinesterase (AChE) and butyrylcholinesterase (BChE) inhibitory activity (IC_50_ 47.44 and 47.40 µg mL^−1^, respectively). Gel filtration on Sephadex LH-20 and further RP-C_18_ preparative HPLC of EtOAc fraction afforded 15 known and 3 new compounds, stereoisomers of larixinol. The structures of the isolated spirobiflavonoids **15**, **26**, and **29** were elucidated using 1D and 2D NMR and MS spectroscopic techniques. The relative configuration of isolated compounds was assigned based on coupling constants and ROESY (rotating-frame Overhauser spectroscopy) correlations along with applying the DP4+ probability method in case of ambiguous chiral centers. Determination of absolute configuration was performed by comparing calculated electronic circular dichroism (ECD) spectra with experimental ones. Compounds **26** and **29**, obtained in sufficient amounts, were evaluated for activities against AChE and BChE, and they showed a weak inhibition only towards AChE (IC_50_ 294.18 µM for **26**, and 655.18 µM for **29**). Furthermore, molecular docking simulations were performed to investigate the possible binding modes of **26** and **29** with AChE.

## 1. Introduction

*Yucca schidigera* Roezl ex Ortgies (syn. *Yucca mohavensis* Sargent) belonging to the Asparagaceae family, is a small evergreen tree (up to 5 m tall), distributed from Southern Nevada to Mexico (Baja California) [1]. Native Americans used the plant for food and fiber, and its extracts have been used for centuries in folk medicine to treat a wide variety of inflammatory disorders, headaches, arthritis, rheumatism, and bacterial infections (gonorrhea) [2], but nowadays it is mostly used in cosmetics, the beverage industry, as foaming agent in soft drinks, and as a food supplement in the form of condensed yucca syrup [3]. The syrup is obtained from mechanically pressed logs of yucca, and steroidal glycosides (furostanol- and spirostanol-type) with middle- and short-length saccharide chains are its predominant constituents [4,5], while the bark contains more polar, bidesmosidic saponins [6]. These products possess the generally recognized as safe (GRAS) label given by the FDA, which allows human dietary use. However, there are several publications mentioning a wide variety of phenolic compounds present in the bark of the plant (the waste) byproducts of *Y. schidigera* in commercial applications [7,8,9,10]. Among them, derivatives of *trans*-resveratrol (*trans*-3,4′,5-trihydroxystilbene) and *trans*-3,3′,5,5′-tetrahydroxy-4′-methoxystilbene, such as yuccaols A–E and yuccaone A, which are unique spiro-compounds including C_15_ and C_14_ units condensed to form a γ-lactone ring, very rarely occur in the plant kingdom [11]. These compounds are known for their various antioxidant, radical scavenging, inhibiting iNOS expression, and platelet aggregation activities in vitro [7,12]. At first, yuccaols A–E and yuccaone A were isolated from *Y. schidigera* bark [7,9]. Lately, yuccaols C–E have been identified in roots of *Y. gloriosa* L. [13] and yuccaols C and D in *Y. pringlei* Greenm [14]. Similarly to yuccaols, gloriosaols A–E [13] and yuccalides A–C [15] possess spiro-structures and have been isolated from *Y. gloriosa* roots. Spirobiflavonoids also belong to the group mentioned, and the first compound of this class, named larixinol, was identified in 1986 in the bark of *Larix gmelini* [16]. Two spirobiflavonoids, daphnodorins M and N, were isolated from the roots and the bark of *Daphne acutiloba* [17]. Later, another four new spirobiflavonoids, named olgensisinols A–D, along with a known one, vitisinol, were isolated from the stem bark of *L. olgensis* HENRY var. *koreana* NAKAI [18], two new spirobiflavonoids from *Abies chensiensis* (3-epi-larixinol and 3,2′-epi-larixinol) [19], and six from *A. sachalinensis* [20]. By 2010, only 16 compounds of this class were identified in plants [11,21].

Simultaneously, dementia is a growing problem in public health as the elderly form a higher proportion of the population. It is estimated that by 2050, over 100 million people worldwide will suffer from this disease [22]. The most common form of dementia in the elderly (ages 65–90) is Alzheimer’s disease (AD) [23]. It is, thus, no surprise that a large volume of research is being directed at understanding the causes of and potential treatments for AD. Although the etiology of AD has not been fully elucidated yet, modern treatment strategies typically comprise anticholinesterases, including acetylcholinesterase (AChE) (EC 3.1.1.7) and butyrylcholinesterase (BChE) (EC 3.1.1.8); antioxidants; α, β, and γ secretase inhibitors; *N*-methyl-D-aspartate receptor agonists [24]; and inhibitors of the phosphorylation of AD-associated protein [25]. Given the complexity of the disease, other mechanisms are also involved in AD development, such as oxidative stress, neuroinflammation, excitotoxicity, metal dyshomeostasis, and mitochondrial damage [26]. However, there is abundant evidence that defects in cholinergic synaptic transmission, in particular nicotinic acetylcholine receptor (nAChR)-mediated signaling, plays a major role. The most remarkable biochemical change in AD patients is a reduction of acetylcholine levels in the hippocampus and cortex of the brain. Thus, inhibition of AChE and BChE is the major approach for treating AD [27,28]. Over recent decades, several pharmaceuticals have become available on the market for clinical use; however, none of them has the ability to discontinue the disease. This is why there is still a great need for the discovery of new drug candidates of natural and synthetic origin with multitarget activities to treat AD [24,25]. There are numerous AChE/BChE inhibitors (AChEi/BChEi) of natural and semi-synthetic origin identified, most of which belong to the alkaloid, terpenoid, and phenolic groups of natural products [25]. The oldest known, discovered in 1846, is a highly toxic parasympathomimetic derivative of indole: physostigmine. There are many natural and synthetic derivatives of indole, many times patented as AChEi [28]. Another alkaloid, galantamine, belonging to the isoquinoline group of compounds, was approved in 2001 as AChEi [29]. Huperzine A, a sesquiterpene alkaloid isolated in 1986 from *Lycopodium serratum* Thunb., was approved in China in 1990 as an anti-Alzheimer drug, and was patented and distributed in the USA as a dietary supplement [30].

A great number of phenolic compounds have been identified as candidates for AD treatment [25,31,32]. They constitute of one of the widest chemical classes amongst plant secondary metabolites. To date, phenolic substances have been identified with many pharmacological effects including antioxidant, anti-inflammatory, antimutagenic, chemopreventive, anticancer, and antiviral activities. Some plant phenolics have been demonstrated to inhibit both AChE and BChE to varying extents. Most of these studies focused on in vitro tests, and only few studies were performed on insects, tissue, and animal models, with rarely any clinical studies [33]. Phenolics, besides their AChE and BChE inhibitory activities, also have very important antioxidant activity, which may enhance their protective effects. It has been proven that oxidative stress caused by reactive oxygen species (ROS) is involved in the aging processes. It has been suggested that free radicals damage mitochondria in certain areas of the brain that are particularly important for memory and cognitive processes and are associated with the pathogenesis of AD [34,35,36]. Hence, supplementation of the diet with antioxidants in people may reduce the risk of AD [34]. This was a major point of a number of studies performed on plants with high antioxidant potential [37]. Moreover, numerous reports indicated multitarget effects of resveratrol on AD [25]. Resveratrol oligomers showed a significant AChE/BChE inhibitory activity [38], and it was suggested to be used as a starting compound in the design of multitargeted drugs for the treatment of AD [39]. The diverse biological effects of the constituents of *Y. schidigera* bark encouraged us to investigate further the structurally related compounds using modern chromatographic and spectroscopic techniques.

Furthermore, as nature possesses the ability to create innumerable complex chemical structures, very often with chiral properties [40,41], the need to properly assign stereochemistry of natural products has emerged [42,43]. The use of quantum chemical calculations and computer-assisted structure elucidation (CASE) methods in solving structural validation problems simplified this task and reduced the risk of misinterpretations [44,45]. DP4-based nuclear magnetic resonance (NMR) chemical shift calculation is one of the most advanced approaches for stereochemical assignments of organic molecules when only one set of experimental data is available [46]. This method implements gauge-independent atomic orbital (GIAO) NMR for chemical shift calculations of geometries obtained by Merck molecular force fields (MMFFs) [47,48,49,50]. As it failed in assignment of some challenging molecules, Grimblat et al. [51] developed a DP4+ probability-based chemical shift analysis, where using B3LYP/6-31+G** geometries and adding the unscaled shift values significantly increased the performance of the method, which led to more accurate and confident results in establishing the stereochemistry of challenging isomeric compounds. However, relying solely on this approach often is not sufficient in determining the absolute configuration (AC) of natural compounds. One of the approaches, when the chiral compound possesses an appropriate chromophore, is the use of electronic circular dichroism (ECD) by comparing the experimental spectrum with the one calculated by time-dependent density functional theory (TDDFT) [52,53,54]. This, along with the careful study of nuclear Overhauser effects (NOEs) observed in the NMR spectra and using H–H or C–H coupling constants, provides an unambiguous tool for assignment of the AC.

Thus, the aim of this work was to study numerous phenolics of the plant, evaluate the AChE/BChE inhibitory activity of newly isolated compounds, and to assign their stereochemistry and absolute configurations using DP4+ probability-based chemical shift analysis and quantum chemical calculation methods.

## 2. Results

### 2.1. Identification of the Constituents Found in the Y. schidigera Ethyl Acetate Fraction

**Figure 1:**
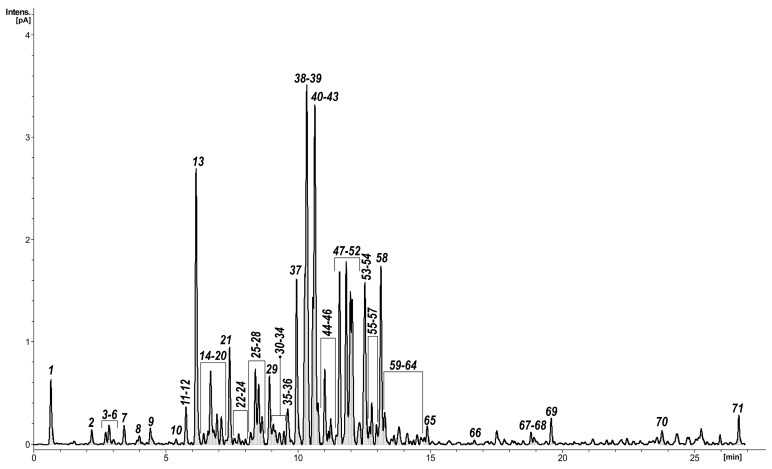
Ultra-high-performance liquid chromatography–charged aerosol detector (UHPLC-CAD) profile of the *Yucca schidigera* ethyl acetate fraction.

**Figure 2:**
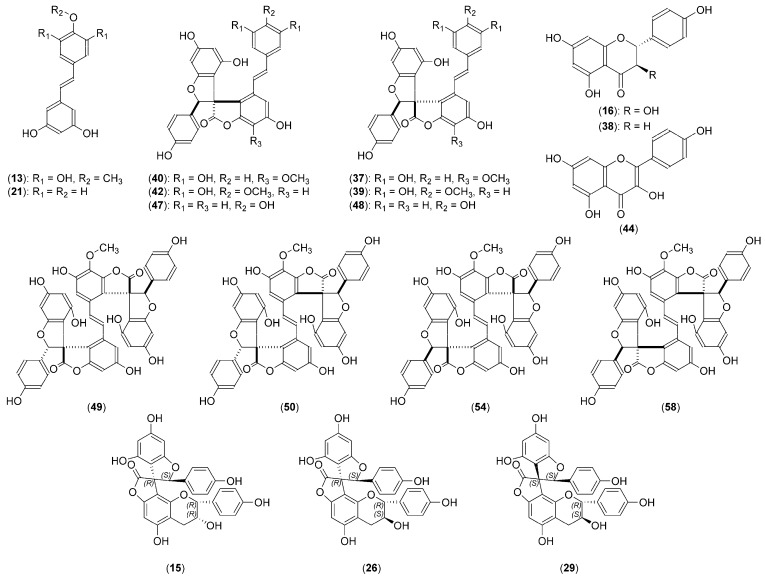
General structures of phenolic compounds isolated from *Y. schidigera* bark.

Initial chromatographic ultra-high-performance liquid chromatography–charged aerosol detector–mass spectrometry (UHPLC–CAD–MS) analysis of the ethyl acetate fraction of the methanolic extract obtained from *Y. schidigera* bark revealed the presence of numerous (over 70) peaks, tentatively identified as phenolic acids (**2**, **4**, **5**), flavan-3-ols (**7**), flavanonols (**12**, **16**), flavonols (**20**, **23**, **31**, **44**), flavanones (**38**), stilbenoids (**13**, **21**), spirobiflavonoids (**15**, **26**, **29**, **30**, **36**), yuccaols (**37**, **39**, **42**, **47**, **48**), yuccalide A (**40**), and gloriosaols (**46**, **49**, **50**, **52**, **54**, **58**, **60**) (Figure 1, Table 1, peak numbers assigned based on retention times). Their identity was confirmed by the application of accurate mass measurements, MS/MS fragmentation patterns, ultraviolet–visible (UV–vis) spectra, and by comparison with existing literature data. To ensure the identity of the main fraction components, a multistep purification procedure was applied and led to the isolation of 18 compounds (Figure 2), which were further analyzed using various spectral techniques: 1D and 2D NMR, quadrupole time-of-flight-high-resolution electrospray ionization mass spectrometry (QTOF–HRESIMS), optical rotations, ECD, and, for compounds **15**, **26**, **29**, calculation of both ECD and NMR spectra. As a result, we isolated three new compounds and confirmed the presence of aromadendrin, naringenin, resveratrol, *trans*-3,3′,5,5′-tetrahydroxy-4′-methoxystilbene, yuccaols A–E, yuccalide A, and surprisingly, gloriosaols A and C–E, previously reported in roots of *Y. gloriosa* [13,55].

### 2.2. Structural Characterization of the New Phenolic Compounds

Yuccalechin A (**15**) was isolated as an off-white, amorphous solid exhibiting a UV absorption maximum at 215 nm, with a specific rotation of [α]^D^_20_ = +95. The negative HRESIMS spectra of **15** showed deprotonated molecule at m/z 541.1131, and its molecular formula was determined as C_30_H_21_O_10_ (calcd. 541.1140). The ^13^C-NMR spectra of **15** showed 25 signals, sorted by the distortionless enhancement by polarization transfer with retention of quaternaries (DEPTQ) and heteronuclear single quantum coherence (HSQC) experiments into 1 CH_2_, 9 CH, and 15 quaternary carbons with a characteristic signal at δ_C_ 177.1, assignable to the γ-lactone carbonyl in C-1″. The difference in the observed number of carbon atoms compared to HRESIMS was explained by the presence of two similar sets of aromatic protons, corresponding to the *para*-substituted aromatic groups at δ_H_ 7.15 (2H, d, *J* = 8.5 Hz, H-2′/H-6′) and δ_H_ 6.72 (2H, d, *J* = 8.5 Hz, H-3′/H-5′), and at δ_H_ 7.06 (2H, d, *J* = 8.5 Hz, H-11″/H-15″) and δ_H_ 6.68 (2H, d, *J* = 8.5 Hz, H-12″/H-14″), due to AA′XX′ systems (Table 2). The third aromatic set of *meta*-coupled protons appeared at δ_H_ 5.81 (1H, d, *J* = 2.0 Hz, H-6″) and at δ_H_ 5.98 (1H, d, *J* = 2.0 Hz, H-8″) and suggested the presence of a 2,4,6-tri-*O*-substituted phenyl group. Additionally, the ^1^H-NMR and 2D-COSY (correlation spectroscopy) spectra exhibited three oxygenated methines at δ_H_ 6.25 (1H, s, H-3″), correlating in the HSQC spectrum with the spiro-center C-atom at δ_C_ 91.2 (C-3″), δ_H_ 4.91 (1H, br s, H-2), and δ_H_ 4.46 (1H, ddd, *J* = 4.4, 2.9, 1.6 Hz, H-3), and a methylene group at δ_H_ 2.96 (1H, dd, *J* = 17.0, 4.4 Hz, H-4β)/2.90 (1H, dd, *J* = 17.0, 2.9 Hz, H-4α), suggesting the epiafzelechin-type substructure [66] (Figure 2).

**Figure 3:**
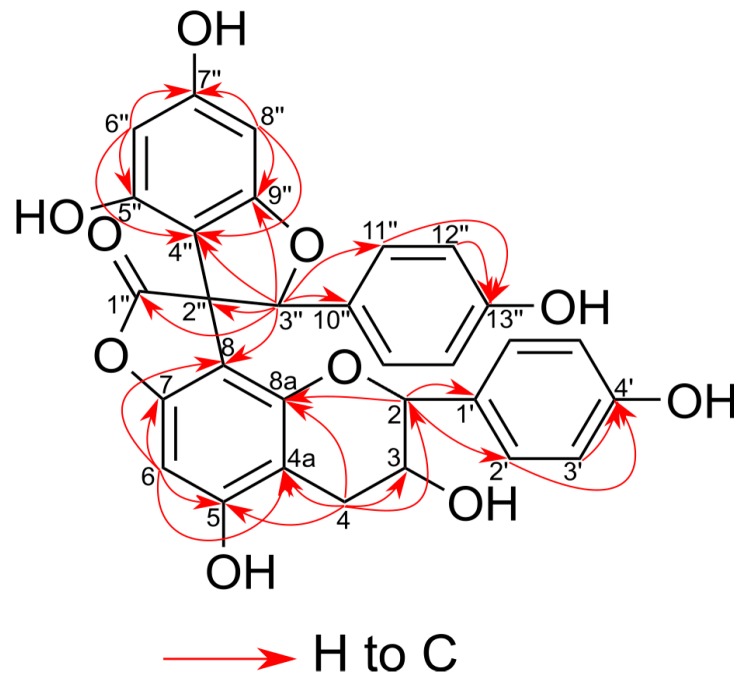
Key heteronuclear multiple bond coherence (HMBC) correlations for compounds **15**, **26**, and **29**.

Analysis of long-range correlations visible in the heteronuclear multiple bond coherence (HMBC) spectrum gave characteristic cross-peaks (Figure 3) between H-3″ and spiro-center C-2″ (δ 61.5), γ-lactone C-1″ (δ 177.1), *p*-hydroxyphenyl C-10″ (δ 128.3), and dihydrobenzopyran C-8 (δ 106.7), while the H-2 correlated with C-8a (δ 153.1) and C-1′ (δ 130.6) of the B-ring. These data were in close agreement with those of larixinol [16,67].

**Figure 4:**
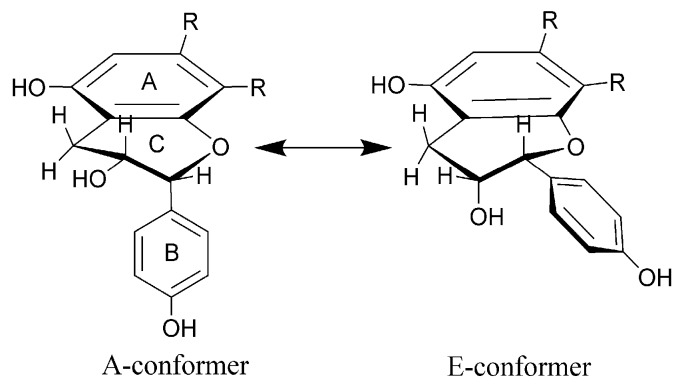
Structures of A and E conformers. Ring B is in a pseudo-axial position in the A-conformer and in a pseudo-equatorial position in the E-conformer.

The flavan-3-ol compounds, like (+)-(2*R*,3*S*)-catechin and (–)-(2*R*,3*R*)-epicatechin, consist of two benzene rings (A and B) and a pyran C-ring. They have two stereocenters and, therefore, four possible diastereomers, 2,3-*trans*-(2*R*,3*S*)/(2*S*,3*R*) and 2,3-*cis*-(2*R*,3*R*)/(2*S*,3*S*), with the C-ring being conformationally flexible. The rapid flexing within the C-ring can bring the B-ring into a pseudoequatorial (E-conformer) or a pseudoaxial (A-conformer) position (Figure 4). The equilibrium between different forms depends on the solvent used, hence H–H and C–H coupling constants should be considered as time-averaged values (e.g., A:E ratios of about 41:59 (DMSO), 30:70 (dioxane), and 33:67 (water) for (+)-catechin) [68,69]. The 2,3-*trans*-flavan-3-ols showed the preference of the B-ring to be in an equatorial position, while for the 2,3-*cis*-compounds the distorted equatorial position, or a significant population of the axial C-2 aryl conformations was present [70].

According to the small coupling constant (*J* ≈ 2 Hz), protons H-2 and H-3 in **15** were deduced as having *cis-*relative configurations, similarly to epicatechin [71] and epiafzelechin [66]. The orientation of protons H-2 and H-3 can be deduced also from the direct H-C coupling constants, which depend on the stereochemistry of the heteroatom-substituted cyclohexane C-ring, presenting smaller values for axial H-C than for the equatorial atoms (typically Δ*J* = 4 Hz)—the so-called normal Perlin effect—which reflects the greater length of the axial C-H bonds. The bond lengthening in the axial position occurs as a result of hyper-conjugative σ_C-H_ → σ*_C__-H_ interactions of anti-periplanar C-H bonds in the axial position [72,73]. On the other hand, one has to remember that axial protons at the β-carbons (i.e., C-3 of C-ring) of hetero-substituted cyclohexanes could also present coupling constants larger than those of the equatorial protons (the reverse Perlin effect) [74,75], but no evidence was found to substantiate it in the case of **15**. Furthermore, the ^1^*J*_CH_ coupling displays an angular dependence for C-H bonds adjacent to π-bonds. In many cases, σ_C-H_ → π* interactions lengthen C–H bonds, with maximized overlap of interacting orbitals when the C–H bond is aligned with the π-bond, thus giving the minimum ^1^*J*_CH_ value [76]. In order to explore these previously undescribed properties of flavan-3-ols, we measured a series of C–H couplings (^1^*J*_CH_, ^2^*J*_CH_ and ^3^*J*_CH_) using the F2-coupled HSQC [77] and HSQC–HECADE experiments [78] for (+)-catechin and (—)-epicatechin (close analogs of (+)-afzelechin and (–)-epiafzelechin) and compounds **15**, **26**, and **29** (Table 3). The accurate values of ^1^*J*_CH_ for C-2 (144.0 Hz) and C-3 (146.2 Hz) for compound **15** suggested that H-2 adopted a pseudoaxial, and H-3 a pseudoequatorial orientation.

Additionally, ^2^*J*_CH_ coupling provides conformational information that can identify the position of an oxygen functionality—when it is gauche to its geminal proton, the coupling becomes large (4–7 Hz), and when it is anti, ^2^*J*_CH_ becomes small (0–2 Hz). On the other hand, ^3^*J*_CH_ coupling follows a Karplus-type dependence, thus showing smaller values for gauche (1–3 Hz) than for anti-periplanar conformation (5–8 Hz) [79,80]. The data gathered in Table 3 for compound **15** agreed with the relative 2,3-*cis*-configuration.

**Figure 5:**
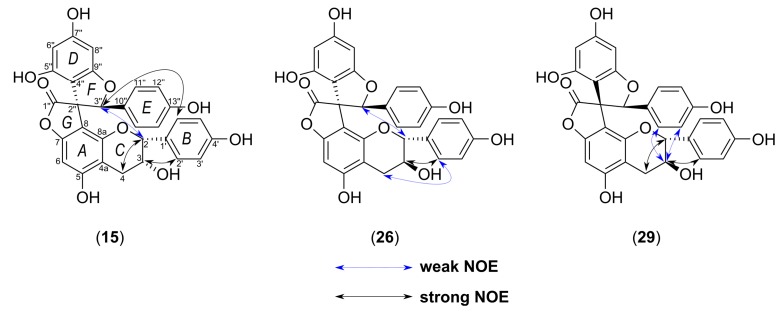
Structures of compounds **15**, **26**, and **29** isolated from the bark of *Y. schidigera* and key nuclear Overhauser effect (NOE) correlations.

The relative configuration between the C-2″/C-3″ of the benzofuran F-ring was deduced primarily on the basis of the chemical shift of C-1″(177.1), as reported by Nakashima et al. [15]. The article reported that ^13^C NMR spectra of yuccaols A–E and gloriosaols A–E in CD_3_OD showed the γ-lactone carbonyl atoms appearing within range δ_C_ 174.6–176.9 for compounds with *cis*-configurations between C-2″ and C-3″, while for those with *trans*-configuration between δ_C_ 178.6–181.1. Interestingly, we observed that yuccaols A–E, yuccalide A and gloriosaol A, and gloriosaols C–E isolated in this study had ^1^*J*_CH_ couplings measured for the C-3″ appearing within range 154.2–155.0 Hz for compounds with the *cis*-configuration between C-2″ and C-3″ (2″*R*,3″*S* or 2″*S*,3″*R*), and 152.9–153.3 Hz for compounds adopting the *trans*-configuration (2″*R*,3″*R* or 2″*S*,3″*S*), and they were inversely correlated with the chemical shifts of corresponding C-1″ carbon atoms. The ^1^*J*_CH_ for C-3″ in compounds **15**, **26**, and **29** were 157.2 (*cis*-), 156.3 (*cis*-), and 150.8 Hz (*trans*-), respectively, while the chemical shifts of C-1″ were 177.1, 177.1, and 180.6, respectively. Additionally, the DP4+ probability calculation was implemented as confirmation for establishing the relative configuration of **15**. Based on the aforementioned observation and 2D-ROESY correlations from H-3″ to H-2 and to H-2′/H-6′ (Figure 5), the relative configuration of **15** was determined as 2″R*,3″S*,2R*,3R*.

However, because of the flexibility of the C-ring, to confirm the stereochemistry of chiral centers C-2 and C-3, all the possible isomers had to be generated and optimized using the DFT/B3LYP/6-31G(d,p)/IEFPCM/methanol (B3LYP = Becke, 3-parameter, Lee–Yang–Parr; IEFPCM = integral equation formalism for the polarizable continuum model) level of theory. Among them, only 2″R*,3″S*,2R*,3R* and 2″R*,3″S*,2S*,3R* were consistent with observed NOEs and coupling constants. Subsequently, geometrical optimization and further NMR calculations of the two isomers were performed using the gauge-independent atomic orbital (GIAO) method by implementing mpw1pw91/6-311G+ (d,p)/IEFPCM/methanol. The DP4+ probability calculation indicated that the probability of all atoms (sum of scaled and unscaled probabilities of all H and C atoms) for isomer 2″R*,3″S*,2R*,3R* was 100%, which was in accordance with the observed coupling constants (Figure S11). Furthermore, the mean average error (MAE) and corrected mean average error (CMAE) values along with correlation coefficients were calculated to evaluate correct/incorrect assignments. As shown in Table 4, MAE and CMAE values showed higher errors for incorrect isomers, confirming the relative configuration of **15** as for 2″R*,3″S*,2R*,3R*.

**Figure 6:**
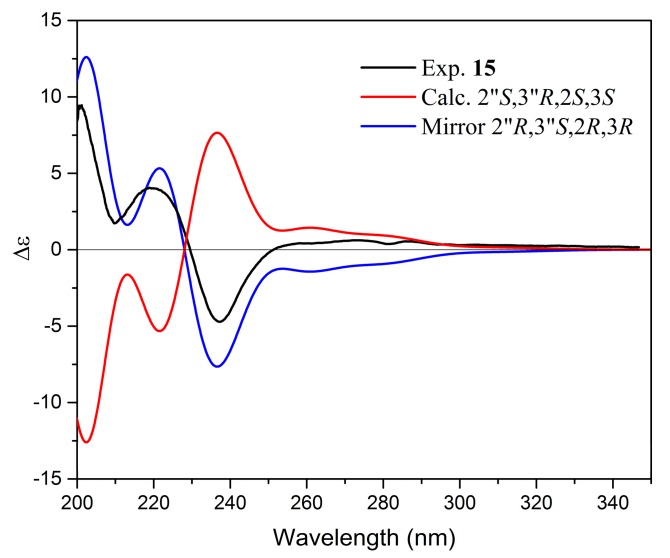
Comparison of experimental and calculated electronic circular dichroism (ECD) spectra of **15**.

In order to determine the absolute configuration of **15**, the 3D structure was optimized by conformational analysis (refer to method section). All conformers occurring in the energy window of 5 kcal mol^−1^ were subjected to geometrical optimization and minimization (Figure S10). Further calculations of excitation states, rotatory strength, and, hence, simulated ECD spectrum were performed by the TD-DFT/B3LYP/6-31G(d,p)/IEFPCM/methanol level of theory. All spectra obtained were Boltzmann averaged and, after applying UV correction of +10 nm and half band of 0.2 eV, compared to the experimentally obtained spectrum of **15**, recorded in methanol. The ECD spectrum calculated was in good agreement with the experimental one (Figure 6), while its enantiomer showed opposite Cotton effects. Therefore, the absolute configuration of **15** was assigned as 2″*R*,3″*S*,2*R*,3*R*.

Finally, the structure of yuccalechin A, a new spirobiflavonoid, was established as (2*S*,2′*R*,3*R*,3′*R*)-3′,4,5′,6-tetrahydroxy-2,2′-bis(4-hydroxyphenyl)-3′,4′-dihydro-2*H*,2′*H*,8′*H*-spiro[benzofuran-3,9′-furo [2,3-*h*]chromen]-8′-one.

**Figure 7:**
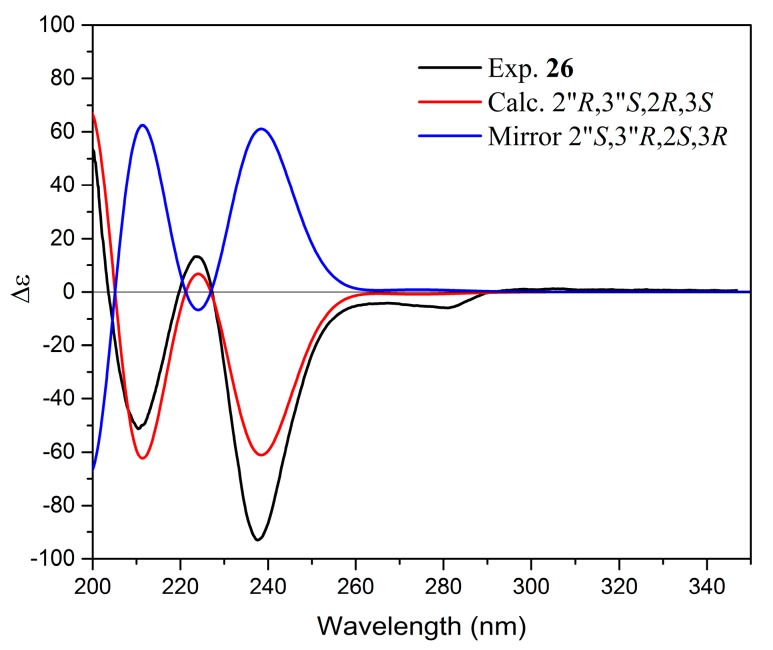
Comparison of experimental and calculated ECD spectra of **26**.

Yuccalechin B (**26**) was obtained as an off-white, amorphous solid exhibiting UV absorption maximum at 210 nm, with a specific rotation of [α]^D^_20_ = −175. The negative HRESIMS spectra of **26** showed a deprotonated molecule at m/z 541.1123, and its molecular formula was determined as C_30_H_21_O_10_ (calcd 541.1140), the same as **15**. The ^1^H and ^13^C-NMR spectra of compound **26** were similar to those of **15**, except for the signals of H-2 (δ 4.99, 1H, d, *J* = 5.8 Hz)/C-2 (δ 81.6), H-3 (δ 4.05, 1H, dt, *J* = 5.8, 5.5 Hz)/C-3 (δ 68.1), H-4 (δ 2.68, 2H, d, *J* = 5.5 Hz)/C-4 (δ 26.8), H-2′/H-6′ (δ 6.98, 2H, d, *J* = 8.5 Hz), and of H-3″ (δ 6.13, 1H, s) (Table 2). The analysis of 2D-NMR spectra provided sufficient data to ascribe the planar structure of **26** as identical with the one of **15**, but the difference in the observed retention time (Figure 1, Table 1) and different optical rotation suggested that compound **26** was a stereoisomer of **15**. The ^1^H-^1^H vicinal coupling constants observed for the C-ring suggested that it adopted 2,3-*trans*-configuration (afzelechin-type), with a di-pseudo-equatorial orientation of H-2/H-3, as suggested by the large ^1^*J*_CH_ coupling constants, large ^2^*J*_C3-H2_, and small ^2^*J*_C2-H3_, which were reasonably similar to that of (+)-catechin (Table 3) and NOEs observed between H-2′/H-6′ and both H-3 and H-4 (Figure 5). The relative configuration between C-2″/C-3″ of the benzofuran ring was deduced from the chemical shift of C-1″ (177.1) and ^1^J_CH_ for C-3″ (156.3 Hz); therefore, it was a *cis*-isomer. In order to establish and prove the relative configuration of C-2/C-3, possible isomers of the C-ring—2″*R*,3″*S*,2*R*,3*S*, 2″*R*,3″*S*,2*S*,3*R,* and 2″*S*,3″*S*,2*S*,3*S*—were generated and imported to conformational analysis followed by geometrical optimization (Figure S24) and DP4+ probability analysis of calculated chemical shifts. However, after the first optimization step, isomer 2″*R*,3″*S*,2*S*,3*R* was excluded from further calculation. There were two reasons for this: a) the lowest energy and most stable conformers showed over space correlations between H-3″ and H-2′/6′, while this is the connection only observed in the case of compound **15**, and b) low-energy conformers of 2″*R*,3″*S*,2*S*,3*R* showed ax-eq configuration between H-2 and H-3, which were not in agreement with observed coupling constants. The DP4+ calculations showed that 2″*R*,3″*S*,2*R*,3*S* with a probability of 99.19% was the correct isomer (Figure S25). The calculated MAE and CMAE values were lower for the correct isomer and further confirmed by the higher correlation coefficient value for that one. Overall, the results obtained were compatible with the result of DP4+ probability calculations (Table 4). Therefore, the relative configuration of **26** was established as 2″R*,3″S*,2R*,3S*. Furthermore, to determine the absolute configuration of **26**, optimized conformers were subjected to ECD spectra simulations using TD-DFT/cam-B3LYP/6-31G(d,p)/IEFPCM/methanol. A comparison of the Boltzmann-averaged spectrum with one obtained experimentally, after applying UV correction of +23 nm and half band of 0.25 eV, resulted in the absolute configuration of **26** determined as 2″*R*,3″*S*,2*R*,3*S* (Figure 7). This outcome coincided with the structure of 3,2′-epi-larixinol, a compound isolated from the aerial parts of *Abies chensiensis* [19], but differing in both NMR chemical shifts and optical rotation compared to **26**. However, the authors did not imply the AC of the aforementioned compound and relied solely on NOEs in determining the relative configuration; thus, our findings still hold true.

In this way, the structure of yuccalechin B was established as (2*S*,2′*R*,3*R*,3′*S*)-3′,4,5′,6-tetrahydroxy-2,2′-bis(4-hydroxyphenyl)-3′,4′-dihydro-2*H*,2′*H*,8′*H*-spiro[benzofuran-3,9′-furo.[2,3-*h*]chromen]-8′-one.

**Figure 8:**
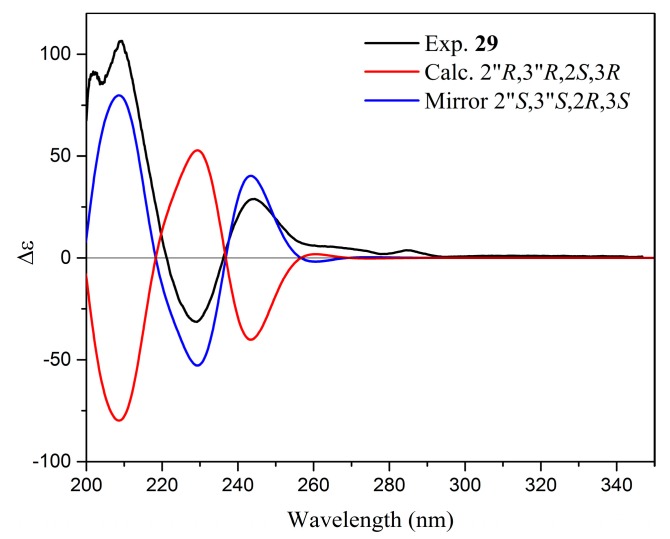
Comparison of experimental and calculated ECD spectra of **29**.

Yuccalechin C (**29**) was obtained as an off-white, amorphous solid exhibiting UV absorption maximum at 211 nm, with a specific rotation of [α]^D^_20_ = +148. The negative HRESIMS spectra of **29** showed a deprotonated molecule at m/z 541.1121, and its molecular formula was determined as C_30_H_21_O_10_ (calcd 541.1140), the same as **15** and **26**. The planar structure of **29** was assigned to be identical to compounds **15** and **26** based on the analysis of 1D and 2D-NMR spectra. However, the ^1^H and ^13^C-NMR spectra presented certain differences when compared to yuccalechin B (**26**), namely signals of upfield-shifted H-2 (δ 4.19, 1H, d, *J* = 9.5 Hz), H-3 (δ 3.85, 1H, ddd, *J* = 9.8, 9.5, 5.8 Hz), and H-3″ (δ 5.72, 1H, s) and downfield-shifted C-3 (δ 30.5), C-1″ (δ 180.6), and C-3″ (δ 94.5). These changes, along with a difference in chromatographic behavior (RT = 8.93 min vs. 8.39 min for **26**) and optical rotation, indicated that compound **29** was a stereoisomer of **15** and **26**. Similarly to **26**, protons H-2 and H-3 of the C-ring were in 2,3-*trans*-configuration (afzelechin-type), but with an energetically favorable di-axial orientation, as indicated by a large ^3^*J*_H-H_ coupling constant, small ^1^*J*_CH_ coupling constants, relatively large ^2^*J*_C3-H2_ and ^2^*J*_C2-H3_ (Table 3)_,_ and NOEs observed between H-2′/H-6′ and H-3 and between H-2 and H-4β (Figure 5). The relative configuration of the spiro-center was apparent from the chemical shift of C-1″ (δ 180.6) and ^1^*J*_CH_ for C-3″ (150.8 Hz), and as a result, the relative configuration between C-2″/C-3″ of the F-ring was deduced as *trans*. Subsequently, to prove the relative configuration, two possible isomers 2″*R*,3″*R*,2*S*,3*R* and 2″*R*,3″*R*,2*R*,3*S* were subjected to an NMR calculation followed by a DP4+ probability calculation. Results implied that the 2″*R*,3″*R*,2*S*,3*R* isomer was the correct isomer with a probability value of 100% (Figure S35). Although the MAE and CMAE values were lower and in favor of the incorrect isomer, the correlation coefficient was higher for correct assignment (Δ*r* = 0.00795). Additionally, the ECD calculation of optimized structures (Figure S34) was performed using TD-DFT/cam-B3LYP/6-31G(d,p)/CPCM/methanol (CPCM = conductor-like polarizable continuum model) and resulted in establishing the absolute configuration of **29** as 2″*S*,3″*S*,2*R*,3*S* (Figure 8).

As a result, the structure of yuccalechin C was elucidated as (2*S*,2′*R*,3*S*,3′*S*)-3′,4,5′,6-tetrahydroxy-2,2′-bis(4-hydroxyphenyl)-3′,4′-dihydro-2*H*,2′*H*,8′*H*-spiro[benzofuran-3,9′-furo[2,3-*h*]chromen]-8′-one.

### 2.3. Anti-Cholinesterase Activities of trans-3,3′,5,5′-Tetrahydroxy-4′-methoxystilbene and Yuccalechins B and C

The modified Ellman’s assay was used to measure the anti-acetylcholinesterase and anti-butyrylcholinesterase activities of **13**, **26**, and **29** [81,82]. As a result, spirobiflavonoids (**26** and **29**) had very low and selective activities against AChE, while the stilbene derivative, *trans*-3,3′,5,5′-tetrahydroxy-4′-methoxystilbene (**13**), was almost inactive against AChE/BChE (Table 5). The result obtained for the latter seems to be in line with our previous results, showing very weak activity of *trans*-resveratrol and much higher, selective against BChE, activity of piceatannol (*trans*-3,3′,4′,5-tetrahydroxystilbene) [83].

### 2.4. Molecular Docking Simulations of Yuccalechins B and C

To explore possible binding interactions of **26** and **29**, molecular docking simulations were carried out on hAChE (PDB: 4EY7) using a Glide module implemented in the *Schrödinger* Small-Molecule Drug Discovery Suite. The results revealed that **26** and **29** exhibited binding energies of –8.43 and –7.76 kcal mol^−1^, respectively, against AChE, which were modulated by hydrogen bonds and π–π stacking contacts inside the active site.

**Figure 9:**
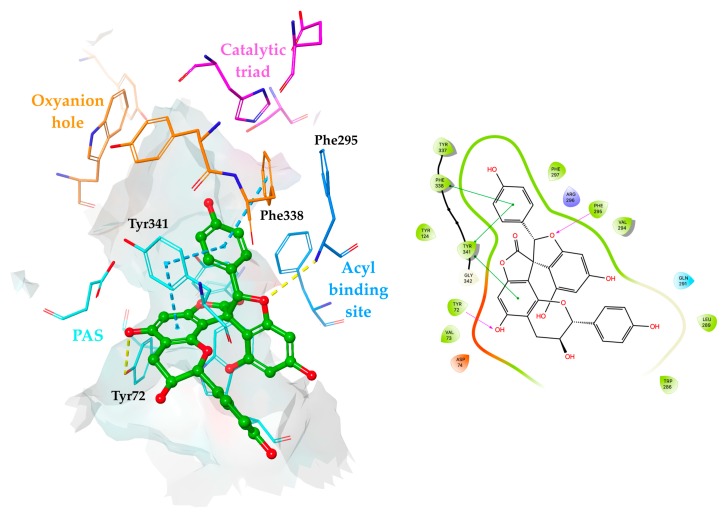
Proposed binding mode and 2D interaction map for **26** (presented as a green ball and stick model) in the hAChE active site (PDB: 4EY7). Hydrogen bonds and π–π stacking contacts are represented by yellow and cyan dashed lines, respectively.

According to molecular docking results, the orientation of **26** was driven by the interactions with the peripheral anionic site (PAS) comprising residues. Compound **26** was located in the bottleneck of the active site gorge via π–π stacking contacts with Tyr341 and forming a hydrogen bond with Tyr72 at the entrance to the gorge through the 5-positioned hydroxyl group of the A-ring. The oxygen atom of the F-ring pointed toward the acyl binding site and was found to interact with the Phe295 backbone via hydrogen bonding. Moreover, the π–π stacking contact between the E-ring and the oxyanion hole residue Phe338 stabilized the occupation of **26** in the binding site (Figure 9). The observation for **26** was found to be in good agreement with the data reported, indicating the aromatic residues at the PAS as a major site of interaction for polycyclic aromatic compounds [84].

**Figure 10:**
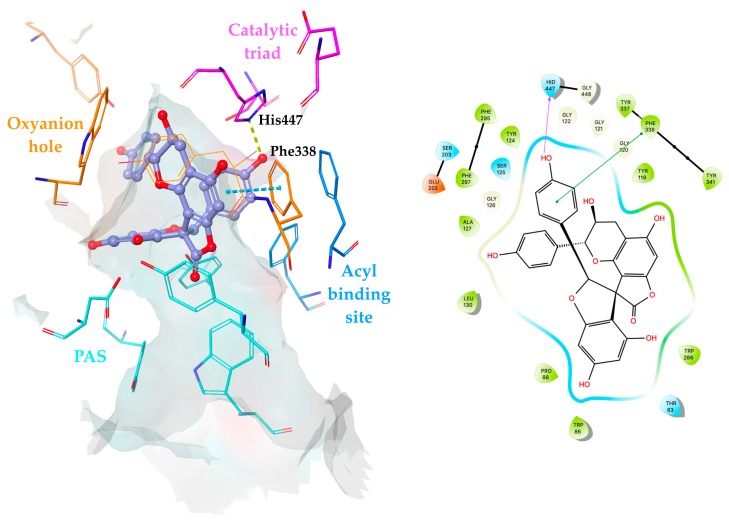
Proposed binding mode and 2D interaction map for **29** (presented as a purple ball and stick model) in the hAChE active site (PDB: 4EY7). Hydrogen bonds and π–π stacking contacts are represented by yellow and cyan dashed lines, respectively.

As depicted in Figure 10, **29** was positioned deeply in the enzyme gorge occupying the region between the oxyanion hole and the acyl binding pocket, which was close to catalytic triad residues. The E-ring displayed interactions with His447 and Phe338 through hydrogen bonding and π–π stacking contacts, respectively, which were found to be responsible for stabilization of **29** at the binding site.

Based on the results of the molecular docking simulations, the reduced number of favorable π–π stacking and hydrogen bonding contacts with key residues, which tend to increase the binding affinity, might be the explanation for the lower inhibitory capacity of these compounds against AChE.

## 3. Discussion

The multistep purification of *Yucca schidigera* bark led to the isolation of three new spirobiflavonoids and confirmed the presence of numerous phenolic compounds, among which aromadendrin, naringenin, yuccalide A, and gloriosaols A and C–E are reported for the first time in this plant. Structures of isolated compounds were elucidated using various spectroscopic methods, including HRESIMS, UV, and ECD spectroscopy and optical rotations. For the new compounds, the relative configuration was established based on NMR chemical shifts, H–H and C–H coupling constants, DP4+ probability calculations, and NOE effects observed in the 2D-ROESY NMR spectra. Here, we report for the first time the usage of ^1^*J*_CH_, ^2^*J*_CH_, and ^3^*J*_CH_ coupling constants in the determination of relative stereochemistry of flavan-3-ols and spirobiflavonoids. Additionally, the absolute configuration of chiral spirobiflavonoids has been described for the first time using ab initio calculations of ECD spectra. The identification of stereochemistry of such compounds reported so far was based on a comparison of the ECD spectra with larixinol (abiesinol E), which possesses the 2″*R*,3″*R*,2*R*,3*R* absolute configuration established by the X-ray crystallographic analysis [19], or chemical methods [20]. Our work, to the best of our knowledge, is the first report on the cholinesterase inhibitory activity of spirobiflavonoids. Tested compounds, yuccalechins B (**26**) and C (**29**), turned out very weak, but they were selective inhibitors of AChE.

## 4. Materials and Methods 

### 4.1. Chemicals and Reagents

Methanol and chloroform as well as acetic acid, *n*-hexane, and ethyl acetate, all of analytical reagent grade, were purchased from Fisher Chemical (Loughborough, UK) and Merck (Darmstadt, Germany), respectively. Acetonitrile and methanol (LC-MS grade) were purchased from Merck (Darmstadt, Germany), while MS-grade formic acid and other chemicals were purchased from Sigma-Aldrich (Steinheim, Germany). Ultrapure water was prepared using a Milli-Q water purification system (Millipore, Milford, MA, USA).

### 4.2. Plant Material

*Yucca schidigera* Roezl ex Ortgies bark was purchased from the commercial source Desert King Int. (Chula Vista, CA, USA). A voucher specimen (No. IUNG-DBCQ-YS01) has been deposited in the Department of Biochemistry and Crop Quality, Institute of Soil Science and Plant Cultivation, State Research Institute, Poland.

### 4.3. Extraction and Isolation

The yucca bark was powdered using an Ultra Centrifugal Mill ZM 200 (Retsch, Germany) with 0.5 mm sieves, and then 202.8 g of the powder was extracted with 100% MeOH (3 L × 3, 1 day each) using an ultrasonic bath (Polsonic 33, Warsaw, Poland) at room temperature. All extraction, isolation, and separation procedures were performed in the dark to avoid any isomerization of compounds. Combined filtered solutions were concentrated under reduced pressure at 35 °C followed by dilution with water and defatted with *n*-hexane in a separating funnel. The solution obtained was evaporated to eliminate MeOH and subsequently was extracted with ethyl acetate to yield 12.3 g of the EtOAc fraction after evaporation and lyophilization (Gamma 2–16 LSC freeze dryer, Martin Christ Gefriertrocknungsanlagen GmbH, Germany). Part of the EtOAc fraction (1.0 g × 3) was separated by gel-filtration chromatography on a glass column (100 × 3.2 cm i.d., Millipore Corp., Bedford MA, USA) filled with Sephadex LH-20 (40–120 µm, Sigma-Aldrich, Steinheim, Germany), eluted with MeOH at a flow rate of 2.5 mL min^−1^, and connected to a Gilson HPLC-ELSD apparatus (Gilson Inc., Middleton, WI, USA). As a result of this separation, 8 fractions (Fr. 1–8) were obtained. The fractions selected (Fr. 3–8) were subsequently purified by flash chromatography (FC) on silica gel cartridges (25 µm, SNAP Ultra, Biotage, Uppsala, Sweden). The samples were dry-loaded onto cartridges and eluted in a stepwise gradient of acetone in the solvent mixtures of chloroform:acetone:acetic acid 75:1.65:8.5; 75:3.3:8.5; 75:16.5:8.5; and 75:24.75:8.5 [85] at a flow rate of 43–55 mL min^−1^. Collected fractions (each 16 mL) were combined according to thin layer chromatography (TLC) carried out on silica gel 60 F_254S_ plates (Merck, Darmstadt, Germany) after visualization under UV light at 254/360 nm. The yielded subfractions were concentrated under reduced pressure and immediately submitted to solid phase extraction (SPE) performed on Oasis HLB 12 cc Vac cartridges (Waters Corp., Milford, MA, USA) for the elimination of acetic acid from the samples. Finally, individual compounds were purified using semi-preparative HPLC.

### 4.4. Semi-Preparative HPLC

Further purification of the FC fractions was carried out either on a Dionex chromatographic system (Dionex™, Sunnyvale, CA, USA) equipped with a PDA-100 detector, a P680 HPLC pump, an ASI-100 automated sample injector, a TCC-100 thermostatted column compartment, a Gilson FC 204 fraction collector (Gilson Inc., Middleton, WI, USA), or on a Gilson chromatographic system (Gilson Inc., Middleton, WI, USA) equipped with an evaporative light-scattering detector (ELSD, Gilson PrepELS II), a Gilson 321 HPLC pump, and a Gilson GX-271 liquid handler/fraction collector with a 2 mL sample loop. The columns used for separations included Kromasil 100-5-C18 (25 × 1.0 cm i.d., 5 µm, AkzoNobel, Bohus, Sweden), column #1; Cosmosil πNAP (25 × 1.0 cm i.d., 5 μm, Nacalai Tesque, INC., Kyoto, Japan), column #2; and Atlantis Prep T3 (25 × 1.0 cm i.d., 5 μm, Waters, Milford, MA), column #3. The separation protocol was individually improved for each fraction. Semi-preparative HPLC analyses were carried out using isocratic or gradient conditions with aqueous acetonitrile or methanol solutions containing 0.1% formic acid (FA). The mobile phase flow rate ranged from 3.4 to 4.0 mL min^−1^, and the columns were held at 35–50 °C. The separation of subfractions SFr. 4–5 in column #1, at 40 °C in an isocratic mode of MeCN/H_2_O/FA (21:79:0.1, *v/v/v*) at a flow rate of 4.0 mL min^−1^, afforded compounds **15** (yuccalechin A, 1.67 mg), **26** (yuccalechin B, 14.3 mg), and **29** (yuccalechin C, 13.34 mg); SFr. 3-2 yielded compound **16** (aromadendrin, 9.4 mg); SFr. 4-1 yielded compound **38** (naringenin, 23.02 mg); SFr. 5-3 yielded compound **13** (*trans*-3,3′,5,5′-tetrahydroxy-4′-methoxystilbene, 23.21 mg); and Fr. 5-1 yielded compound **21** (*trans*-resveratrol, 13.86 mg). The most abundant, Fr. 6 (1012 mg), was divided into 7 subfractions, and SFr. 6-1 afforded compound **44** (kaempferol, 7.21 mg), SFr. 6-4 afforded **47** (yuccaol A, 9.96 mg) and **48** (yuccaol B, 10.1 mg), SFr. 6-5 afforded **37** (yuccaol E, 38.7 mg), **39** (yuccaol C, 116.22 mg), and **42** (yuccaol D, 63.92 mg), SFr. 6-6 afforded **40** (yuccalide A, 3.64 mg), and SFr. 6-7 afforded **49** (gloriosaol E, 19.94 mg) and **50** (gloriosaol D, 19.31 mg). Lastly, Fr. 7 and 8 yielded molecules **54** (gloriosaol A, 14.63) and **58** (gloriosaol C, 11.08 mg), respectively. ^1^H- and ^13^C-NMR spectra of all isolated compounds are available in the Supplementary Materials. Prior to the biological activity assays, the purities of compounds **13**, **26**, and **29** were checked by NMR spectroscopy (electronic reference to access in vivo concentrations 2 (ERETIC2) method) [86] using D-(-)-quinic acid (C_7_H_12_O_6_, 98%, Sigma-Aldrich, St. Louis, MO, USA) in deuterated methanol (30 mM) as the reference sample. The purity was over 60% for all compounds tested.

### 4.5. High-Resolution LC-MS

The EtOAc fraction was subjected to high-resolution LC-MS analysis. Chromatographic separation was performed on a Thermo Scientific Ultimate 3000RS chromatographic system on a Waters BEH C18 column (150 × 2.1 mm i.d.; 1.7 µm, Milford, USA). The effluent was analyzed using a photodiode array detector (200–600 nm, 10 Hz acquisition frequency), Q-TOF MS (Bruker Impact II HD, Bruker, Billerica, MA, USA), and a charged aerosol detector (CAD, Thermo Corona Veo RS) as described in detail in our previous publication [87].

### 4.6. NMR Spectroscopy

The 1D and 2D NMR spectra (^1^H, ^13^C DEPTQ, ^1^H-^13^C HSQC, ^1^H-^13^C H2BC, ^1^H-^13^C HMBC, ^1^H-^13^C F2-coupled HSQC, ^1^H-^13^C HSQC-HECADE, ^1^H-^1^H COSY, and ^1^H-^1^H ROESY) were recorded using an Avance III HD Ascend 500 MHz spectrometer (Bruker BioSpin, Rheinstetten, Germany) in deuterated methanol (MeOH-*d_4_*) at 30 °C. NMR spectra were calibrated to the signal of residual solvent: δ 3.31 for ^1^H and 49.0 for ^13^C.

### 4.7. Optical Rotation [α]

Optical rotations were determined on a P-2000 polarimeter (Jasco, Easton, PA, USA) in MeOH solutions at concentrations of 1 mg mL^−1^.

### 4.8. Electronic Circular Dichroism (ECD) Spectroscopy

Circular dichroism spectra of all analyzed compounds were made at room temperature on a Jasco J-1500 magnetic circular dichroism spectrometer (1 mm or 5 mm pathlength was used) in MeOH. CD spectra were collected at a scan rate of 100 nm min^−1^ with a response time of 1 s. Measurements were taken in the 200–500 nm range. All spectra were baseline corrected, and the final plot was taken from five accumulated plots. The concentrations of compounds were 50 mM.

### 4.9. Enzyme Inhibition Assay

AChE and BChE inhibitory activities were measured by a slightly modified spectrophotometric method of Ellman et al. [81]. Electric eel AChE (Type-VI-S, EC 3.1.1.7, Sigma, St. Louis, MO, USA) and horse serum BChE (EC 3.1.1.8, Sigma, St. Louis, MO, USA) were used as the enzyme sources, while acetylthiocholine iodide and butyrylthiocholine chloride (Sigma, St. Louis, MO, USA) were employed as the substrates of the reaction. 5,5′-Dithio-bis(2-nitrobenzoic) acid (DTNB, Sigma, St. Louis, MO, USA) was used to measure the anticholinesterase activity as the coloring agent. All reagents and conditions were exactly the same as described in our previous publication [88]. Hydrolysis of acetylthiocholine iodide/butyrylthiocholine chloride was monitored by the formation of the yellow 5-thio-2-nitrobenzoate anion, as a result of the reaction of DTNB with thiocholines, catalyzed by enzymes at 412 nm utilizing a 96-well microplate reader (VersaMax Molecular Devices, USA). The measurements and calculations were evaluated by using Softmax PRO 4.3.2.LS software. The percent inhibitions of AChE/BChE were determined by comparing the reaction rates of the samples relative to a blank sample (ethanol in phosphate buffer pH = 8), using the formula (E − S)/E × 100, where E is the activity of enzyme without test sample, and S is the activity of the enzyme with the test sample. The experiments were conducted in six replicates. Galantamine (Sigma, St. Louis, MO, USA) was used as the reference.

### 4.10. DP4+ Probability Calculation

Intended compounds were subjected to conformational analysis by MacroModel 9.1 (Schrödinger. LLC, New York, NY, USA) using an OPLS-3 force field in H_2_O. Geometrical optimization of using DFT/6-31G(d) in the gas phase (compound **29**) or DFT/6-31G(d,p)/IEFPCM/methanol (compound **15** and **26**) in Gaussian 16 was performed [89]. Subsequently, they were submitted to NMR chemical shift calculations using the gauge-independent atomic orbitals (GIAOs) method in rmpw1pw91/6-311G+(d,p)/IEFPCM/methanol. The shift tensors obtained were further adjusted to chemical shifts by using TMS proton and carbon chemical shifts, which were calculated using the same method. All chemical shifts were Boltzmann-averaged, and unscaled chemical shifts were used for the DP4+ probability calculation based on the method and the interactive Excel sheet published by Grimblat et al. [51]

### 4.11. ECD Spectra Calculation

3D structures of isolated compounds were drawn in Maestro (Schrödinger. LLC, New York, NY, USA) and subjected to conformational analysis using MacroModel 9.1 (Schrödinger. LLC, New York, NY, USA) and OPLS-3 as a force field in H_2_O. Geometrical optimization and energy calculations of conformers occurring in the energy window of 5 kcal mol^−1^ were performed by implementing DFT/6-31G(d) in the gas phase for compound **29** or DFT/6-31G(d,p)/IEFPCM/methanol for compound **26** and **29**. Subsequently, ECD spectra of optimized compounds were simulated by using TD-DFT/B3LYP/6-31G(d,p)/IEFPCM/methanol (compound **15**) or TD-DFT/cam-B3LYP/6-31G(d,p)/IEFPCM/methanol (compound **26** and **29**). ECD spectra obtained (with a half-band of 0.2–0.3 eV) were Boltzmann-averaged, and a UV correction of +10 to +25 nm was applied to compare them with experimental spectra obtained in methanol.

### 4.12. Molecular Docking Studies

Molecular docking studies were carried out using the induced fit docking (IFD) protocol implemented in the Schrödinger Small-Molecule Drug Discovery Suite (Small-Molecule Drug Discovery Suite 2019-3, Schrödinger, LLC, New York, NY, 2019). The compounds built via a builder panel in Maestro were subjected to ligand preparation by LigPrep (Schrödinger Release 2019-3: LigPrep, Schrödinger, LLC, New York, NY, 2019) using default conditions. The crystal structure of hAChE (PDB: 4EY7) [90] was retrieved from the Protein Data Bank. The protein was prepared using the protein preparation wizard tool. Water molecules were removed from the crystallographic structure followed by the addition of hydrogen atoms. All atom charges and atom types were assigned. Finally, energy minimization and refinement of the structure was performed up to 0.3 Å RMSD by applying an OPLS3e force field. The centroid of the active site residues was defined as a grid box. Van der Waals (vdW) radius scaling factor 1.00, partial charge cutoff 0.25, and an OPLS3e force field were used for receptor grid generation. The compounds prepared by LigPrep were docked into AChE using the IFD protocol [91], which considers flexibility of both the compounds and receptor. Residues Asp74, Trp86, Tyr124, Tyr133, Ser203, Trp286, Phe295, Phe297, Try337, Phe338, and His447 lining the binding site of AChE were kept as flexible. The initial docking protocol was set to employ a 0.50 vdW radius scaling factor, and the resulting top 20 poses of each compound were taken. An extra-precision (XP) algorithm was employed in redocking of the compounds, with the low energy refined structures generated by the Prime MM-GBSA (molecular mechanics - generalized Born surface area) method. The best conformation for each compound was chosen based on the lowest XP glide score.

## Figures and Tables

**Table 1:** Compounds identified in the *Y. schidigera* ethyl acetate fraction using ultra-high-performance liquid chromatography–quadrupole time-of-flight–tandem mass spectrometry (UHPLC-QTOF-MS/MS).

No.	Compound Name	RT (min)	λ_max_ (nm)	Neutral Formula	Error * (ppm)	Mσ **	Observed [M − H]^−^	Major Fragments (%)	Reference
1.	Mixture of polar compounds	0.66	-	-	-	-	-	-	-
2.	*p*-Hydroxybenzoic acid	2.20	255	C_7_H_6_O_3_	1.5	1.4	137.0242	137.0243 (100), 93.0342 (69)	[56]
3.	Hydroxymethoxyacetophenone	2.73	-	C_9_H_10_O_3_	2.2	6.2	165.0554	165.0553 (100), 123.0450 (25), 152.0112 (6)	[57]
4.	Hydroxybenzylmalic acid	2.73	-	C_11_H_12_O_6_	2.2	1.9	239.0556	179.0347 (100), 239.0557 (77), 177.0553 (55), 149.0604 (32), 133.0656 (11), 195.0659 (11)	[58]
5.	Caffeic acid	2.86	215, 323	C_9_H_8_O_4_	2.3	0.7	179.0346	135.0449 (100), 179.0347 (65)	[56]
6.	Unknown	3.02	-	C_23_H_18_O_10_	0.9	20.4	453.0823	310.0484 (100), 325.0716 (49), 376.0585 (48), 256.0372 (39)	
7.	(Epi)afzelechin	3.43	-	C_15_H_14_O_5_	1.1	1.4	273.0766	273.0766 (100), 205.0867 (39), 255.0660 (29), 137.0247 (29), 229.0868 (28)	[11]
8.	Unknown	4.00	-	C_22_H_16_O_8_	-0.1	17.4	407.0773	345.0768 (100), 163.0037 (92), 269.0455 (80), 293.0819 (68), 279.0661 (67)	-
9.	Unknown	4.42	-	C_29_H_22_O_9_	0.7	9.6	513.1187	293.0817 (100), 379.0823 (70), 275.0713 (65), 335.0924 (61), 361.0721 (59)	-
10.	Unknown	5.39	-	C_20_H_24_O_7_	0.2	23.0	375.1448	297.1133 (100), 282.0900 (28), 151.0401 (22), 315.1256 (9), 136.0186 (6)	-
11.	Unknown	5.77	323	C_14_H_12_O_4_	-0.3	1.4	243.0663	243.0661 (100), 201.0554 (7), 225.0558 (3)	-
12.	Dihydrorobinetin	5.77	263	C_15_H_12_O_7_	-0.3	0.9	303.0511	183.0299 (100), 139.0402 (67), 165.0195 (20), 137.0246 (9), 97.0294 (4), 95.0500 (3)	[59]
13.	*Trans*-3,3′,5,5′-tetrahydroxy-4′-methoxystilbene	6.15	227, 315	C_15_H_14_O_5_	1.0	2.6	273.0766	258.0526 (100), 240.0434 (9), 273.0766 (5), 196.0528 (4), 188.0477 (2), 172.0532 (2)	[8,9]
14.	Unknown	6.43	-	C_30_H_24_O_11_	2.4	18.5	559.1232	373.0715 (100), 433.0922 (42), 418.0688 (32), 401.0657 (22), 125.0245 (16)	-
15.	Yuccalechin A	6.60	215	C_30_H_22_O_10_	1.8	6.0	541.1131	308.0346 (100), 281.0468 (88), 267.0313 (60), 269.0453 (41), 415.0843 (30)	-
16.	Aromadendrin (dihydrokaempferol)	6.70	295	C_15_H_12_O_6_	1.3	4.8	287.0557	259.0608 (100), 125.0243 (68), 287.0243 (39), 243.0658 (25), 201.0554 (14)	[60]
17.	Unknown	6.81	-	C_29_H_24_O_10_	1.9	25.9	531.1286	257.0459 (100), 393.0605 (65), 531.1293 (64), 378.0752 (45), 269.0489 (40)	-
18.	Glyceryl azelate	6.81	-	C_12_H_22_O_6_	1.3	11.9	261.1340	187.0974 (100), 125.0971 (31), 261.1342 (15), 169.0869 (13)	[61]
19.	Azelaic acid	6.94	-	C_9_H_16_O_4_	1.8	0.4	187.0972	187.0973 (100), 125.0970 (73), 169.0868 (20), 97.0655 (3)	[62]
20.	Myricetin	7.10	370	C_15_H_10_O_8_	0.8	1.4	317.0300	317.0301 (100), 178.9983 (59), 151.0035 (42), 137.0244 (19)	[60]
21.	*Trans*-resveratrol	7.42	215, 307	C_14_H_12_O_3_	3.3	4.5	227.0706	227.0707 (100), 185.0603 (8), 183.0809 (2)	[9,63]
22.	Oxododecanedioic acid	7.60	-	C_12_H_20_O_5_	2.9	10.7	243.1231	225.1124 (100), 243.1232 (45), 181.1230 (24), 207.1021 (20), 199.1335 (17)	-
23.	Tetrahydroxyflavone	7.60	-	C_15_H_10_O_6_	2.2	14.5	285.0398	285.0400 (100), 151.0033 (91), 257.0450 (55), 107.0137 (15), 213.0545 (11)	[64]
24.	Unknown	7.77	-	C_28_H_22_O_8_	2.5	5.6	485.1230	257.0451 (100), 485.1227 (61), 391.0819 (35), 347.0921 (34), 227.0706 (24)	-
25.	Unknown	8.21	-	C_20_H_26_O_6_	3.0	4.8	361.1646	361.1645 (100), 346.1417 (51), 165.0548 (27), 179.0701 (8), 122.0374 (7)	-
26.	Yuccalechin B	8.39	210	C_30_H_22_O_10_	3.1	16.5	541.1123	308.0334 (100), 281.0459 (99), 267.0331 (71), 415.0829 (32), 361.0719 (31)	-
27.	Unknown	8.51	319	C_29_H_24_O_19_	3.6	1.9	515.1329	362.0784 (100), 515.1332 (70), 241.0500 (59), 253.0507 (42), 291.0664 (19)	-
28.	Unknown	8.64	319	C_15_H_12_O_6_	2.8	13.3	287.0553	287.0553 (100), 219.0656 (25), 199.0423 (16), 227.0354 (11), 185.0238 (8)	-
29.	Yuccalechin C	8.93	211	C_30_H_22_O_10_	3.5	4.7	541.1121	308.0337 (100), 281.0459 (93), 267.0306 (43), 269.0449 (39), 415.0828 (30)	-
30.	Larixinol isomer IV	9.07	-	C_30_H_22_O_10_	3.0	15.3	541.1124	267.0300 (100), 269.0450 (71), 281.0463 (62), 308.0339 (48), 445.1277 (46)	-
31.	Quercetin	9.14	219, 367	C_15_H_10_O_7_	2.7	3.0	301.0346	301.0344 (100), 151.0031 (77), 178.9979 (51), 121.0292 (10), 273.0401 (10)	[64]
32.	Unknown	9.30	-	C_30_H_22_O_10_	3.6	6.7	541.1121	269.0444 (100), 390.0732 (39), 362.0798 (15), 308.0335 (15), 281.0459 (13)	-
33.	Unknown	9.30	-	C_16_H_12_O_8_	2.7	14.8	331.0451	316.0214 (100), 331.0448 (9)	-
34.	Unknown	9.48	-	C_15_H_12_O_5_	3.3	2.1	271.0603	253.0496 (100), 227.0704 (52), 271.0602 (26), 185.0598 (4), 225.0551 (3)	-
35.	Unknown	9.62	315	C_24_H_20_O_8_	3.9	5.3	435.1068	420.0834 (100), 435.1069 (96), 281.0462 (62), 393.0964 (50), 240.0420 (31)	-
36.	Larixinol isomer V	9.62	315	C_30_H_22_O_10_	3.4	4.1	541.1122	308.0329 (100), 281.0460 (97), 267.0328 (60), 415.0826 (34), 497.1219 (23)	[20]
37.	Yuccaol E	9.96	207, 319	C_30_H_22_O_10_	3.7	8.9	541.1120	498.0936 (100), 513.1171 (52), 267.0310 (27), 429.0974 (11), 375.0511 (11)	[7,63]
38.	Naringenin	10.27	211, 291	C_15_H_12_O_5_	3.5	1.6	271.0612	271.0603 (100), 151.0031 (83), 119.0499 (23), 177.0188 (11), 107.0133 (6)	[64]
39.	Yuccaol C	10.33	211, 319	C_30_H_22_O_10_	3.2	4.5	541.1123	267.0303 (100), 513.1171 (65), 498.0934 (63), 239.0348 (36), 429.0981 (23)	[9,63,65]
40.	Yuccalide A	10.57	211, 323	C_30_H_22_O_10_	4.9	12.0	541.1114	498.0934 (100), 267.0304 (76), 513.1168 (72), 239.0348 (26), 429.0979 (18)	[15]
41.	Unknown	10.57	211, 323	C_29_H_20_O_9_	3.6	8.9	511.1016	267.0302 (100), 483.1069 (36), 239.0345 (33), 385.0713 (26), 399.0857 (22)	-
42.	Yuccaol D	10.64	211, 323	C_30_H_22_O_10_	3.6	7.4	541.1121	498.0934 (100), 267.0303 (89), 513.1168 (77), 239.0348 (30), 429.0979 (21)	[7,63,65]
43.	Unknown	10.76	215	C_30_H_22_O_9_	4.0	5.3	525.1170	399.0868 (100), 267.0300 (84), 269.0443 (66), 361.0695 (42), 333.0766 (38)	-
44.	Kaempferol	11.02	265, 363	C_15_H_10_O_6_	3.3	8.2	285.0395	285.0394 (100)	[64]
45.	Unknown	11.16	-	C_30_H_22_O_9_	3.3	4.3	525.1174	399.0865 (100), 267.0310 (72), 361.0707 (60), 307.0607 (38), 293.0445 (36)	-
46.	Gloriosaol isomer I	11.24	320	C_45_H_30_O_15_	3.6	22.9	809.1483	267.0296 (100), 239.0343 (55), 211.0395 (11), 513.1166 (3), 541.1121 (3)	-
47.	Yuccaol A	11.58	207, 327	C_29_H_20_O_8_	3.1	7.2	495.1070	267.0310 (100), 467.1120 (67), 239.0347 (60), 399.1224 (21), 357.1122 (16)	[9,63,65]
48.	Yuccaol B	11.83	207, 327	C_29_H_20_O_8_	2.6	9.2	495.1073	267.0312 (100), 239.0349 (50), 467.1122 (44), 399.1229 (16), 357.1124 (12)	[9,63,65]
49.	Gloriosaol E	11.98	207, 323	C_45_H_30_O_15_	3.8	12.0	809.1481	267.0294 (100), 239.0343 (44), 211.0395 (8), 541.1118 (3), 513.1171 (3)	[13,65]
50.	Gloriosaol D	12.05	207, 323	C_45_H_30_O_15_	3.6	7.3	809.1483	267.0294 (100), 239.0343 (45), 211.0395 (9), 541.1117 (3), 513.1171 (3)	[13,65]
51.	Unknown	12.33	-	C_30_H_22_O_9_	3.1	5.3	525.1175	267.0314 (100), 497.1226 (94), 239.0359 (41), 413.1018 (29), 482.0994 (25)	-
52.	Gloriosaol isomer II	12.33	-	C_45_H_30_O_15_	3.3	14.3	809.1485	267.0297 (100), 239.0345 (43), 211.0396 (9), 541.1125 (4), 513.1166 (3)	-
53.	Unknown	12.53	283	C_16_H_16_O_3_	2.7	15.0	255.1020	255.1020 (100)	-
54.	Gloriosaol A	12.53	207, 319	C_45_H_30_O_15_	3.3	3.9	809.1485	267.0295 (100), 239.0343 (49), 211.0395 (10), 541.1117 (4), 513.1172 (3)	[55,65]
55.	Trihydroxyoctadecadienoic acid	12.71	-	C_18_H_32_O_5_	2.7	5.1	327.2168	327.2168 (100), 211.1334 (39), 229.1439 (32), 171.1021 (19), 239.1284 (8)	-
56.	Unknown	12.79	215	C_30_H_22_O_9_	3.3	7.3	525.1174	267.0312 (100), 399.0868 (95), 361.0711 (70), 307.0603 (40), 349.0712 (39)	-
57.	Unknown	12.97	-	C_17_H_16_O_6_	2.2	3.9	315.0867	315.0867 (100), 178.9981 (23), 297.0761 (20), 194.0216 (15), 152.0111 (11)	-
58.	Gloriosaol C	13.14	207, 327	C_45_H_30_O_15_	3.0	2.2	809.1488	267.0295 (100), 239.0344 (42), 211.0396 (8), 541.1121 (4), 513.1173 (3)	[13]
59.	Unknown	13.28	-	C_18_H_20_O_6_	1.6	16.8	331.1182	285.1127 (100)	-
60.	Gloriosaol isomer III	13.63	-	C_45_H_30_O_15_	3.2	38.4	809.1486	267.0301 (100), 239.0349 (36), 508.0790 (12), 365.0655 (11), 463.0835 (10)	-
61.	Trihydroxyoctadecenoic acid	13.82	-	C_18_H_34_O_5_	2.1	7.1	329.2327	329.2326 (100), 211.1334 (35), 229.1440 (26), 171.1022 (13), 139.1126 (3)	-
62.	Unknown	14.14	-	C_45_H_30_O_14_	2.3	28.1	793.1545	269.0452 (100), 267.0297 (99), 399.0872 (68), 125.0239 (37), 639.1304 (19)	-
63.	Unknown	14.14	-	C_30_H_18_O_11_	2.0	21.9	553.0765	375.0508 (100), 267.0304 (74), 525.0813 (69), 509.0871 (60), 457.0916 (59)	-
64.	Unknown	14.80	-	C_17_H_16_O_4_	1.4	3.5	283.0972	283.0972 (100), 162.0320 (16), 134.0372 (2), 268.0743 (2)	-
65.	Unknown	14.89	-	C_15_H_12_O_4_	1.4	1.6	255.0659	255.0659 (100)	-
66.	Unknown	16.68	-	C_17_H_16_O_5_	2.0	2.4	299.0919	299.0919 (100), 178.0267 (18), 150.0320 (2)	-
67.	Unknown	18.82	-	C_34_H_52_O_11_	3.2	47.2	635.3416	101.0241 (100), 589.3345 (11)	-
68.	Unknown	18.93	-	C_18_H_28_O_4_	2.0	24.8	307.1909	223.1334 (100), 137.0970 (63), 265.1806 (30), 307.1913 (20), 185.1183 (17)	-
69.	Unknown	19.59	-	C_34_H_54_O_11_	4.4	10.3	637.3565	161.0450 (100), 113.0244 (68), 591.3534 (16)	-
70.	Unknown	23.78	-	C_34_H_56_O_10_	3.2	46.4	623.3781	577.3724 (100), 159.0298 (63)	-
71.	Unknown	26.68	-	C_28_H_42_O_6_	3.3	7.0	473.2893	175.0395 (100), 473.2893 (23), 297.2427 (13), 160.0168 (11), 193.0499 (10)	-

* Accuracy of mass measurements expressed in parts per million (ppm). ** Isotopic pattern fit factor (mσ).

**Table 2:** ^1^H and ^13^C-NMR data (MeOH-*d_4_*, 500/125 MHz, 30 °C) for compounds **15**, **26**, and **29**.

Position	Type	15		26		29	
		δ_H_ (*J*, Hz)	δ_C_ (ppm)	δ_H_ (*J*, Hz)	δ_C_ (ppm)	δ_H_ (*J*, Hz)	δ_C_ (ppm)
2	CH	4.91 br s	79.4	4.99 d (5.8)	81.6	4.19 d (9.5)	82.7
3	CH	4.46 ddd (4.4, 2.9, 1.6)	65.6	4.05 dt (5.8, 5.5)	68.1	3.85 ddd (9.8, 9.5, 5.8)	67.7
4α	CH_2_	2.90 dd (17.0, 2.9)	29.6	2.68 d (5.5)	26.8	2.39 dd (16.3, 9.8)	30.5
4β		2.96 dd (17.0, 4.4)				2.93 dd (16.3, 5.8)	
5	C		158.4		158.0		157.6
6	CH	6.16 s	91.6	6.14 s	91.5	6.05 s	91.1
7	C		153.8		154.1		152.8
8	C		106.7		105.8		106.1
4a	C		104.8		104.9		105.6
8a	C		153.1		152.1		153.2
1′	C		130.6		131.2		130.1
2′	CH	7.15 d (8.5)	128.7	6.98 d (8.5)	128.2	7.20 d (8.5)	130.4
3′	CH	6.72 d (8.5)	115.8	6.66 d (8.5)	115.9	6.83 d (8.5)	115.7
4′	C		157.7		157.8		158.3
5′	CH	6.72 d (8.5)	115.8	6.66 d (8.5)	115.9	6.83 d (8.5)	115.7
6′	CH	7.15 d (8.5)	128.7	6.98 d (8.5)	128.2	7.20 d (8.5)	130.4
1″	C		177.1		177.1		180.6
2″	C		61.5		61.5		61.9
3″	CH	6.25 s	91.2	6.13 s	90.6	5.72 s	94.5
4″	C		105.7		105.8		106.1
5″	C		156.1		156.2		155.1
6″	CH	5.81 d (2.0)	96.7	5.91 d (2.0)	96.7	5.78 d (2.0)	96.5
7″	C		161.3		161.4		161.0
8″	CH	5.98 d (2.0)	90.6	5.94 d (2.0)	90.8	5.66 d (2.0)	90.7
9″	C		164.8		164.6		163.8
10″	C		128.3		128.1		128.1
11″	CH	7.06 d (8.5)	128.5	7.03 d (8.5)	128.4	7.07 d (8.5)	128.4
12″	CH	6.68 d (8.5)	115.8	6.68 d (8.5)	115.8	6.60 d (8.5)	115.5
13″	C		158.6		158.6		158.3
14″	CH	6.68 d (8.5)	115.8	6.68 d (8.5)	115.8	6.60 d (8.5)	115.5
15″	CH	7.06 d (8.5)	128.5	7.03 d (8.5)	128.4	7.07 d (8.5)	128.4

**Table 3:** Experimental ^1^*J*_CH_, ^2^*J*_CH_, and ^3^*J*_CH_ (*J* in Hz) couplings of C-2 and C-3 for (+)-catechin (*R*,*S*), (−)-epicatechin (*R*,*R*), and compounds **15**, **26**, and **29**.

*J*-Type	(+)-Catechin	(−)-Epicatechin	15	26	29
^1^ *J*_C2-H2_	146.1	142.9	144.0	148.9	144.7
^1^ *J*_C3-H3_	145.3	146.2	146.2	146.0	144.8
^2^ *J*_C3-H2_	−4.0	+1.0	+1.4	−4.7	−2.5
^2^ *J*_C2-H3_	−1.6	+1.9	+1.5	0.0	−3.3
^2^ *J*_C4-H3_	−1.0	+2.0	−2.2	−1.0	−0.9
^3^ *J*_C4-H2_	+3.2	+1.8	+2.1	+3.5	+3.0

**Table 4:** Calculated mean average error (MAE), corrected MAE (CMAE), and correlation coefficients of the pair of possible diastereomers for each compound.

Parameters	15		26		29	
	2″*R*,3″S,2*S*,3*R*	2″*R*,3″*S*,2*R*,3*R*	2″*R*,3″*S*,2*R*,3*S*	2″*R*,3″*S*,2*S*,3*S*	2″*R*,3″*R*,2*S*,3*R*	2″*R*,3″*R*,2*R*,3*S*
^1^H-MAE	3.5	3.40	3.8	3.53	0.38	0.4
^13^C-MAE	5.6	5.40	5.5	5.18	5.23	4.95
Total MAE	4.85	4.44	4.92	4.60	3.54	3.37
^1^H-CMAE	4.16	3.69	3.29	3.9	0.83	0.7
^13^C-CMAE	10.92	10.74	9.53	10.45	10.65	9.85
Total CMAE	8.57	8.03	8.40	8.17	7.23	6.67
*r* H	0.9703	0.9724	0.9772	0.9826	0.98460	0.93860
*r* C	0.9966	0.9977	0.9983	0.9986	0.99900	0.99770
Total *r*	0.98995	0.99203	0.99377	0.99506	0.99608	0.98812

**Table 5:** Inhibition (% ± S.D.) and IC_50_ values of compounds **13**, **26**, and **29** screened against acetylcholinesterase (AChE) and butyrylcholinesterase (BChE).

Compound	Inhibition (% ± S.D.^a^) at 1000 µM^b^	
	AChE	BChE
**13**	19.33 ± 3.03	25.21 ± 2.58
**26**	80.53 ± 1.22 (IC_50_ = 294.18 ± 5.26 µM)	48.46 ± 2.29
**29**	52.55 ± 2.60 (IC_50_ = 655.18 ± 6.35 µM)	33.41 ± 1.37
**Reference^c^**	97.92 ± 0.01 (IC_50_ = 2.29 ± 0.33 µM)	91.52 ± 1.63 (IC_50_ = 124.03 ± 4.05 µM)

^a^ Standard deviation; ^b^ Final concentration; ^c^ Galantamine HBr.

## Supplementary Materials

Supplementary Materials are available online.
