# Supplementary material for: Yuccalechins A–C from the Yucca schidigera Roezl ex Ortgies Bark: Elucidation of the Relative and Absolute Configurations of Three New Spirobiflavonoids and Their Cholinesterase Inhibitory Activities
Source: Molecules. 2019 Nov 16;24(22):4162. doi: 10.3390/molecules24224162 (PMC6891570; doi:10.3390/molecules24224162)
Supplement: Supplementary file 1 [file molecules-24-04162-s001.zip › CSEARCH Evaluation Report_Yuccalechin_A.pdf]

**Automatic Evaluation Report from CSEARCH**
**created on 2019-10-23 at 15:34:25**
**based on 340,554 reference spectra**

| | |
|---|---|
| **Did you know ?** | **"Structure representation by reference data over "x" shells on average" is the last line in the prediction table.<br>The value of "x" gives you some feeling how good your query structure is represented by the database.<br>In case the value of "x" is below 2.0, it is strongly recommend to contribute your own measurements !** |

**Request from: lpecio@iung.pulawy.pl**

**Compound: Yuccalechin_A**

**Project: YS**

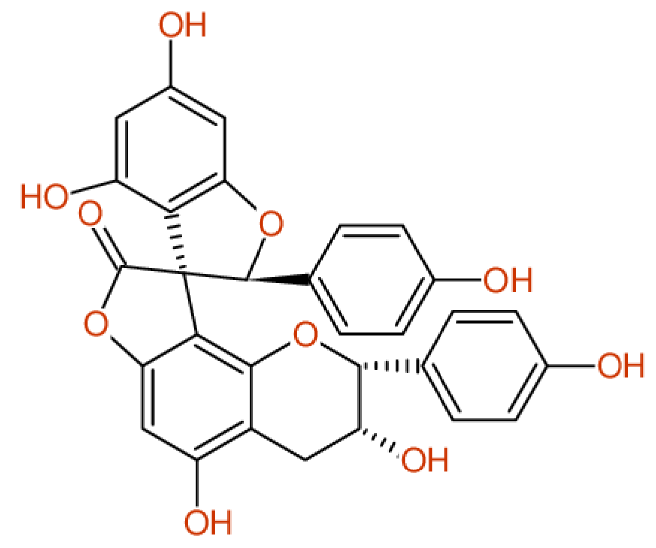


| Database | Number of Entries | Owner of Database |
|---|---|---|
| | | |
| | | |

Please cite the CSEARCH-Robot-Referee as:

N. Haider, W. Robien; http://nmrpredict.orc.univie.ac.at/c13robot/robot.php

| | | |
|---|---|---|
| CSEARCH | 74,997 (A) | CSEARCH-Data / Wolfgang Robien |
| CSEARCH | 56,549 (B) | CSEARCH-Data / Wolfgang Robien |
| CSEARCH | 28,196 (C) | CSEARCH-Data / Wolfgang Robien |
| CSEARCH | 33,587 (D) | CSEARCH-Data / Wolfgang Robien |
| CSEARCH | 39,132 (E) | CSEARCH-Data / Wolfgang Robien + NMR-Database University of Mainz |
| CSEARCH | 26,196 (F) | CSEARCH-Data / Wolfgang Robien |
| CSEARCH | 50,594 (I) | Upcoming CSEARCH-Data / Wolfgang Robien |
| CSEARCH | 31,307 (L) | NMRShiftDB-Data / Version February 2012 |

Permanent URL

This page can be verified by a digital signature

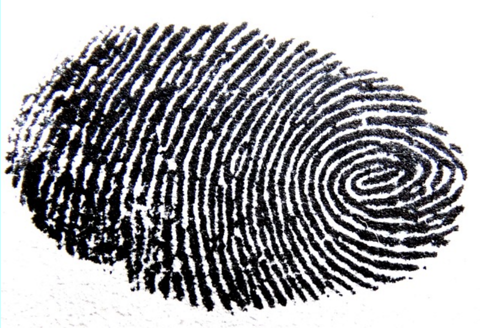

CSEARCH-Version: 9.4.0
Robot-Referee: 2017:06:10

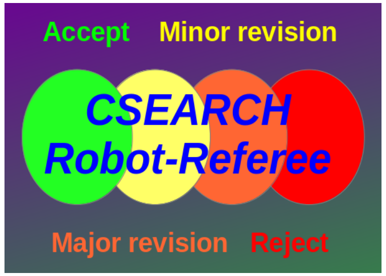


**Request from: lpecio@iung.pulawy.pl**

**Compound: Yuccalechin_A**

**Project: YS**

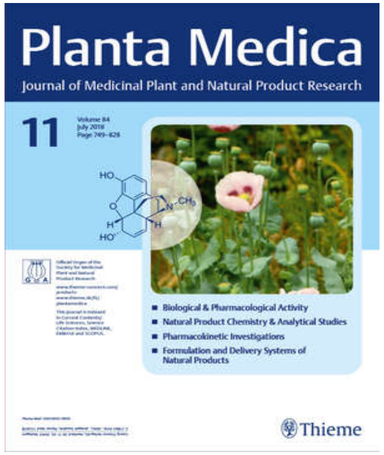


**Recommendation given here**

**Details of Prediction given here**

## Summary of Supplied Data

Understanding the Color Coding Scheme

**Structure Proposal**

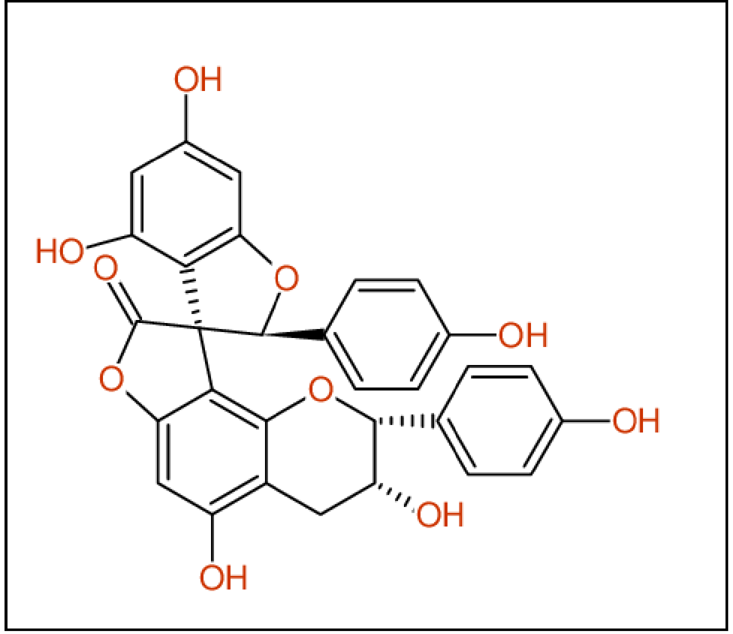


**Molecular formula is: $C_{30}H_{22}O_{10}$** **Molecular weight is: 542.51 amu**

**INCHIKEY is: RNDNBGULZNCSNB-JDEUKJACBJ**

**Numbering Scheme derived from the drawing sequence used during the calculation**

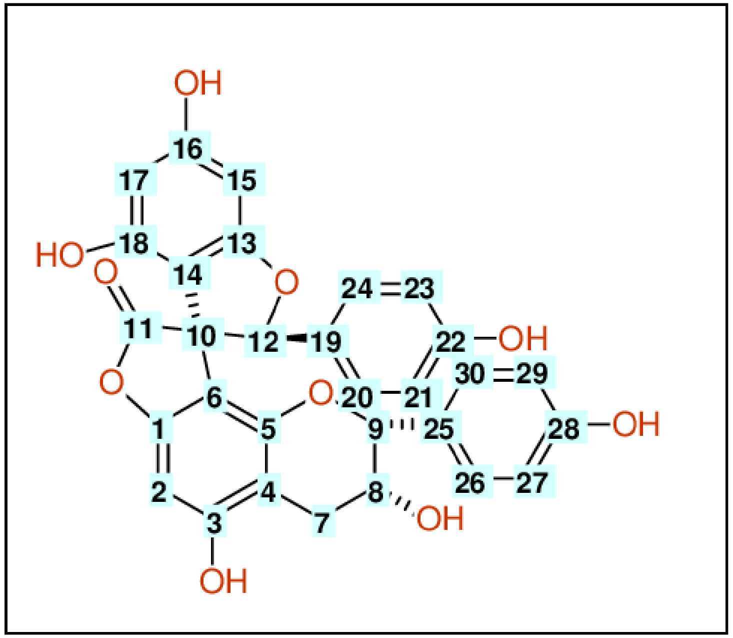

The marked carbons have been fully assigned

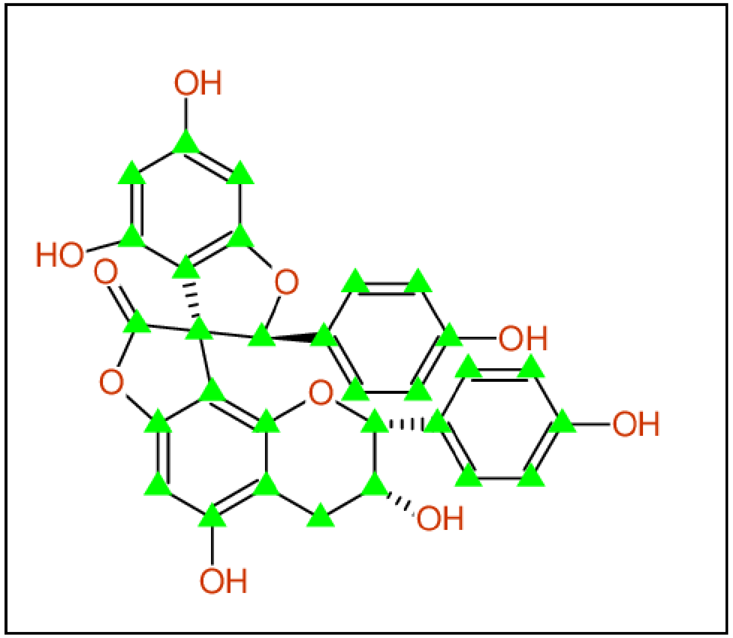


| Carbon number | Chemical Shift Value | Multiplicity from Structure | Multiplicity from Experiment |
|---|---|---|---|
| | | | |
| 1 | 153.80 | S | S |
| 2 | 91.60 | D | D |
| 3 | 158.40 | S | S |
| 4 | 104.80 | S | S |
| 5 | 153.10 | S | S |
| 6 | 106.70 | S | S |
| 7 | 29.60 | T | T |
| 8 | 65.60 | D | D |
| 9 | 79.40 | D | D |
| 10 | 61.50 | S | S |
| 11 | 177.10 | S | S |
| 12 | 91.20 | D | D |
| 13 | 164.80 | S | S |
| 14 | 105.70 | S | S |
| 15 | 90.60 | D | D |
| 16 | 161.30 | S | S |
| 17 | 96.70 | D | D |
| 18 | 156.10 | S | S |

| | | | |
|---|---|---|---|
| 19 | 128.30 | S | S |
| 20 | 128.50 | D | D |
| 21 | 115.80 | D | D |
| 22 | 158.60 | S | S |
| 23 | 115.80 | D | D |
| 24 | 128.50 | D | D |
| 25 | 130.60 | S | S |
| 26 | 128.70 | D | D |
| 27 | 115.80 | D | D |
| 28 | 157.70 | S | S |
| 29 | 115.80 | D | D |
| 30 | 128.70 | D | D |

**The marked carbons have been fully assigned**

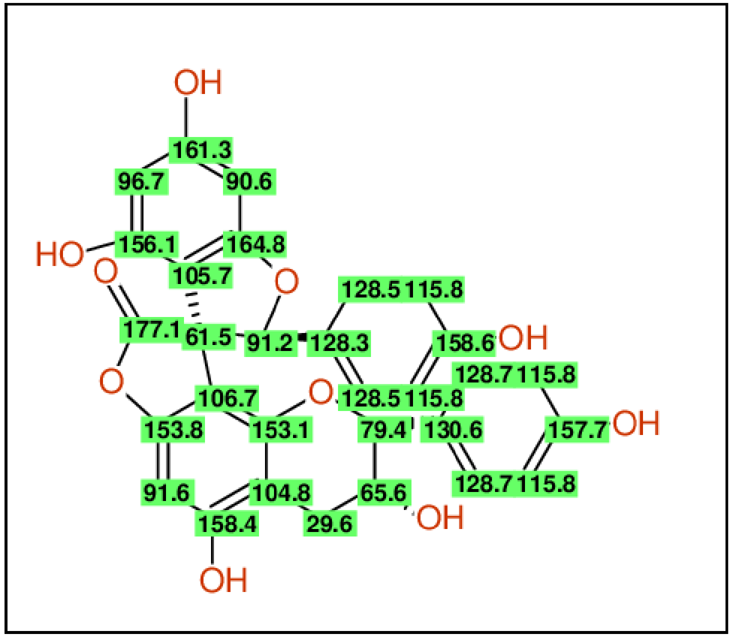


**Graphical summary of the Chemical Shift Data**

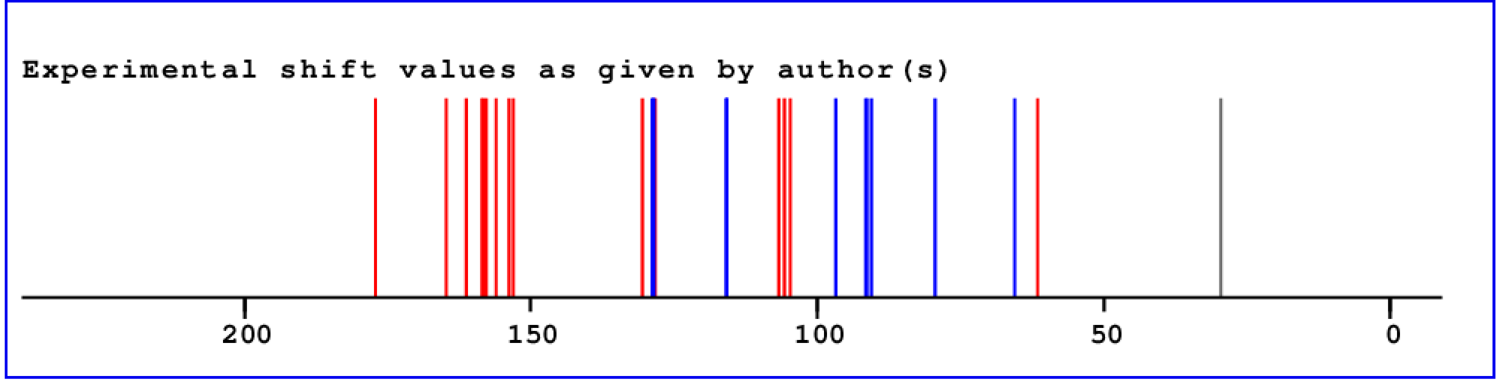


## Searching external databases

Recall this Compound from PUBCHEM ( Skeleton-Match from searching 121,500,754 compounds )
Recall this Compound from PUBCHEM ( Skeleton-Match from searching 121,500,754 compounds )
Recall this Compound from PUBCHEM ( Skeleton-Match from searching 121,500,754 compounds )
Recall this Compound from PUBCHEM ( Skeleton-Match from searching 121,500,754 compounds )
Recall this Compound from PUBCHEM ( Skeleton-Match from searching 121,500,754 compounds )
Recall this Compound from PUBCHEM ( Skeleton-Match from searching 121,500,754 compounds )
Recall this Compound from PUBCHEM ( Skeleton-Match from searching 121,500,754 compounds )

4,400,967 Compounds searched in Emolecules - nothing found

Search the Internet for this compound ( Skeleton only )
Search the Internet for this compound ( Skeleton + Stereochemistry )

Search CHEMSPIDER for this compound ( Skeleton only )
Search CHEMSPIDER for this compound ( Skeleton + Stereochemistry )

Search the Internet for the molecular formula $C_{30}H_{22}O_{10}$

Search CHEMSPIDER for the molecular formula $C_{30}H_{22}O_{10}$

(Description)

## Performing Symmetry Analysis

### Eventually Symmetry Error: Same shiftvalue - Different environment

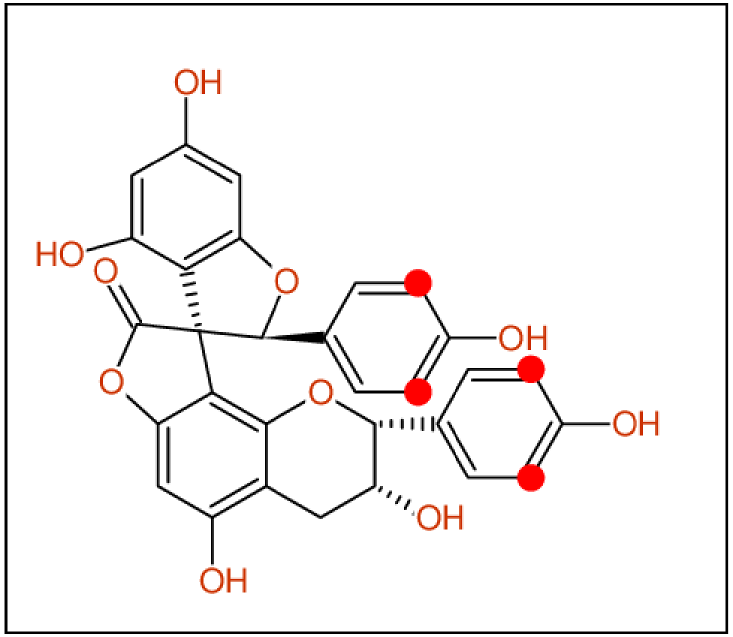


## Basic Evaluation: Checking Multiplicities

| Checking lines & multiplicity | Carbons/Lines | Singlet | Dublet | Triplet | Quartet | Odd | Even | None |
|---|---|---|---|---|---|---|---|---|
| | | | | | | | | |
| From structure | 30 | 15 | 14 | 1 | 0 | 16 | 14 | 0 |
| From spectrum | 30 | 15 | 14 | 1 | 0 | 16 | 14 | 0 |

[Overall impression on compatibility of multiplicity from structure and experiment](#)

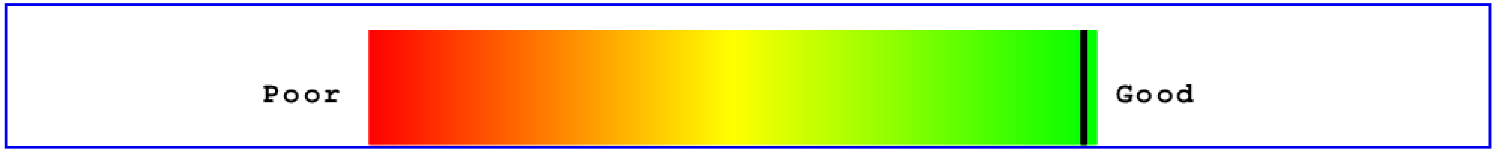


## Evaluation based on Spectrum Prediction

[Numbering Scheme](#)

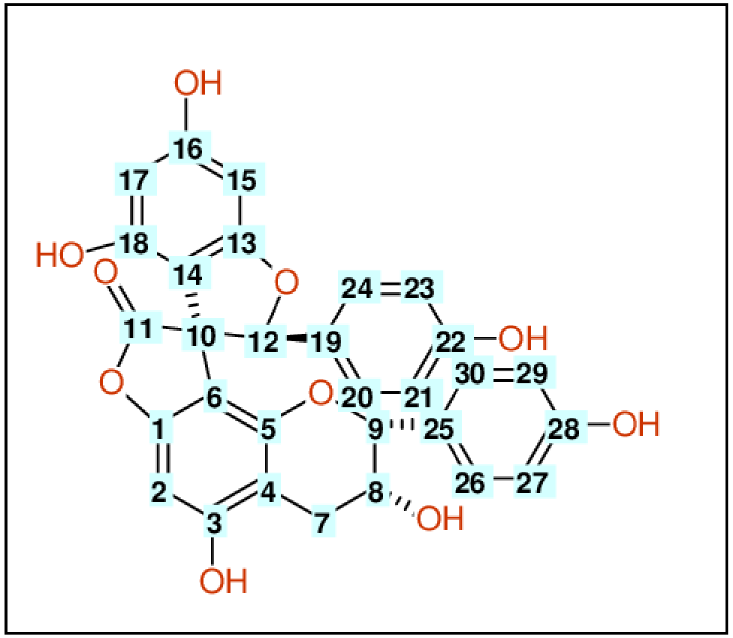


| Carbon Number ▲ ▼ | Neural Network Prediction ▲ ▼ | HOSE-Code Prediction ▲ ▼ | Preferred Value from both Predictions ▲ ▼ | Experimental values ▲ ▼ | Difference (Exp-Pred/ppm) ▲ ▼ | Assignment | Prediction Quality |
|---|---|---|---|---|---|---|---|
| 1 | 154.4 | 153.9 | 154.1 | 153.8 | 0.3 | Assigned by author | |

| Carbon Number ▲ ▼ | Neural Network Prediction ▲ ▼ | HOSE-Code Prediction ▲ ▼ | Preferred Value from both Predictions ▲ ▼ | Experimental values ▲ ▼ | Difference (Exp-Pred/ppm) ▲ ▼ | Assignment | Prediction Quality |
|---|---|---|---|---|---|---|---|
| 2 | 93.8 | 95.9 | 94.9 | 91.6 | 3.3 | Assigned by author | Only reference material with low similarity |
| 3 | 162.5 | 156.1 | 159.3 | 158.4 | 0.9 | Assigned by author<br>Check assignment - maybe 158.60 ? | Large Difference between NET & HOSE |
| 4 | 100.5 | 103.5 | 102.0 | 104.8 | 2.8 | Assigned by author<br>Check assignment - maybe 105.70 ? | Only reference material with low similarity |
| 5 | 148.0 | 152.2 | 148.0 | 153.1 | 5.1 | Assigned by author | Only reference material with low similarity |
| 6 | 104.0 | 133.8 | 104.0 | 106.7 | 2.7 | Assigned by author<br>Check assignment - maybe 104.80 ? | Large Difference between NET & HOSE<br>Only reference material with low similarity |
| 7 | 29.4 | 29.5 | 29.5 | 29.6 | 0.1 | Assigned by author | |
| 8 | 67.4 | 66.9 | 67.0 | 65.6 | 1.4 | Assigned by author | |
| 9 | 79.6 | 80.5 | 80.2 | 79.4 | 0.8 | Assigned by author | |
| 10 | 61.6 | 65.0 | 61.6 | 61.5 | 0.1 | Assigned by author | Only reference material with low similarity<br>Only very few similar structures |
| 11 | 172.8 | 175.6 | 172.8 | 177.1 | 4.3 | Assigned by author | Only reference material with low similarity |
| 12 | 88.9 | 79.7 | 88.9 | 91.2 | 2.3 | Assigned by author | Large Difference between NET & HOSE<br>Only reference material with low similarity |
| 13 | 162.0 | 149.6 | 162.0 | 164.8 | 2.8 | Assigned by author<br>Check assignment - maybe 161.30 ? | Large Difference between NET & HOSE<br>Only reference material with low similarity |
| 14 | 98.1 | 128.4 | 98.1 | 105.7 | 7.6 | Assigned by author<br>Check assignment - maybe 106.70 ? | Large Difference between NET & HOSE<br>Only reference material with low similarity |
| 15 | 93.4 | 90.7 | 90.7 | 90.6 | 0.1 | Assigned by author | |
| 16 | 162.8 | 162.1 | 162.1 | 161.3 | 0.8 | Assigned by author<br>Check assignment - maybe 164.80 ? | |
| 17 | 97.8 | 97.1 | 97.1 | 96.7 | 0.4 | Assigned by author | |
| 18 | 159.7 | 156.1 | 156.9 | 156.1 | 0.8 | Assigned by author | Only very few similar structures |
| 19 | 128.7 | 135.4 | 128.7 | 128.3 | 0.4 | Assigned by author<br>Check assignment - maybe 128.70 ? | Large Difference between NET & HOSE<br>Only reference material with low similarity |
| 20 | 128.5 | 127.8 | 127.8 | 128.5 | 0.7 | Assigned by author<br>Check assignment - maybe 128.30 ? | |
| 21 | 115.3 | 115.7 | 115.7 | 115.8 | 0.2 | Assigned by author | |
| 22 | 160.2 | 158.3 | 158.3 | 158.6 | 0.3 | Assigned by author<br>Check assignment - maybe 158.40 ? | |
| 23 | 115.3 | 115.7 | 115.7 | 115.8 | 0.2 | Assigned by author | |
| 24 | 128.5 | 127.8 | 127.8 | 128.5 | 0.7 | Assigned by author<br>Check assignment - maybe 128.30 ? | |
| 25 | 128.5 | 131.1 | 130.5 | 130.6 | 0.1 | Assigned by author | |
| 26 | 129.7 | 129.5 | 129.5 | 128.7 | 0.8 | Assigned by author | |
| 27 | 116.0 | 115.6 | 115.6 | 115.8 | 0.2 | Assigned by author | |
| 28 | 158.1 | 157.8 | 157.8 | 157.7 | 0.1 | Assigned by author | |
| 29 | 116.0 | 115.6 | 115.6 | 115.8 | 0.2 | Assigned by author | |
| 30 | 129.7 | 129.5 | 129.5 | 128.7 | 0.8 | Assigned by author | |

| Carbon Number ▲ ▼ | Neural Network Prediction ▲ ▼ | HOSE-Code Prediction ▲ ▼ | Preferred Value from both Predictions ▲ ▼ | Experimental values ▲ ▼ | Difference (Exp-Pred/ppm) ▲ ▼ | Assignment | Prediction Quality |
|---|---|---|---|---|---|---|---|
| Absolute<br>Signed | 1.83ppm (30)<br>0.33ppm (30) | 3.53ppm (30)<br>-1.19ppm (30) | 1.36ppm (30)<br>0.64ppm (30) | | | 1.32ppm (30)<br>0.64ppm (30) | Average deviation to experimental values<br>( Number of shift pairs used ) |
| Structure representation by reference data over 3.3 shells on average | | | | | | | |

Visualization of the differences between predicted and experimental values

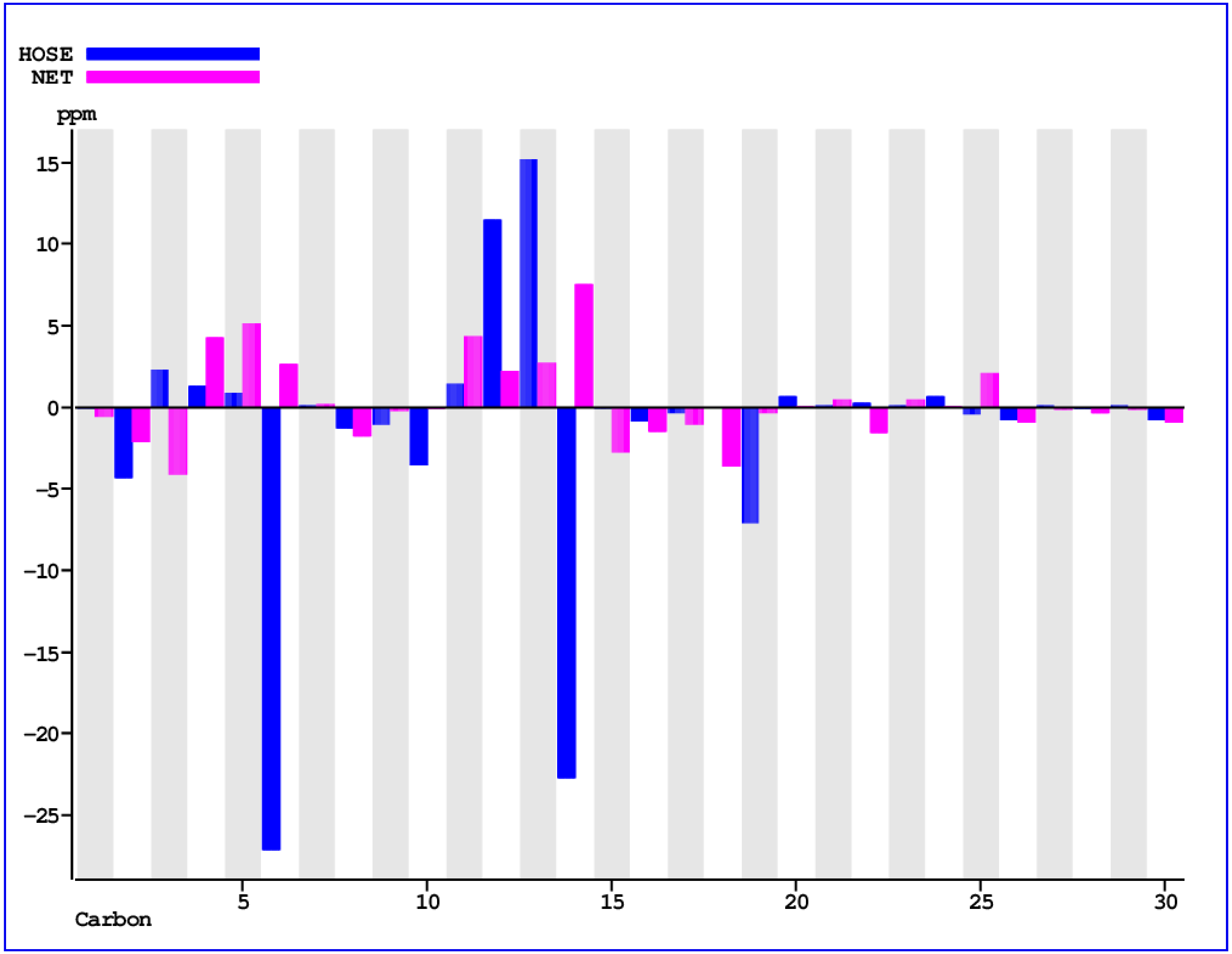

## Quality of the Spectrum Prediction

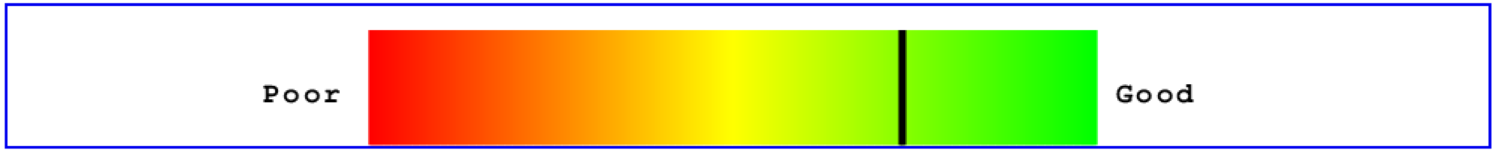


## Experimental Chemical Shift Values as given

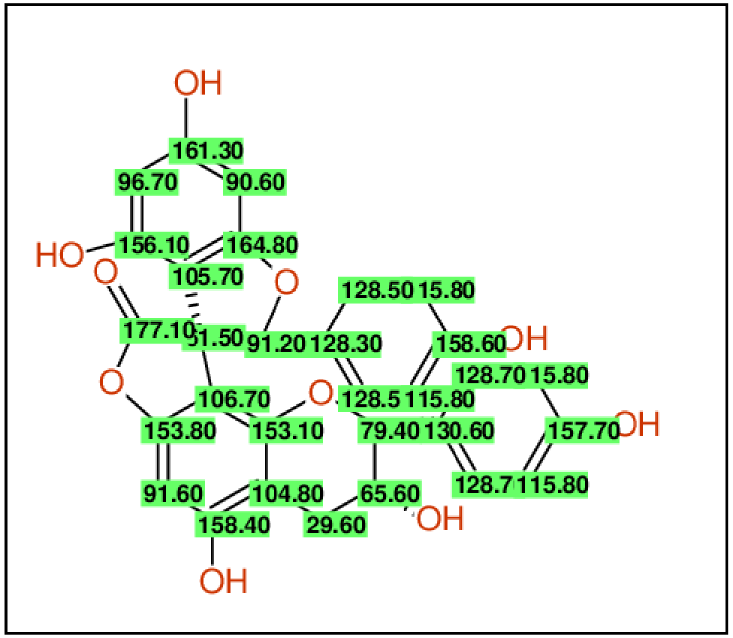


## Experimental Chemical Shift Values using Symmetry

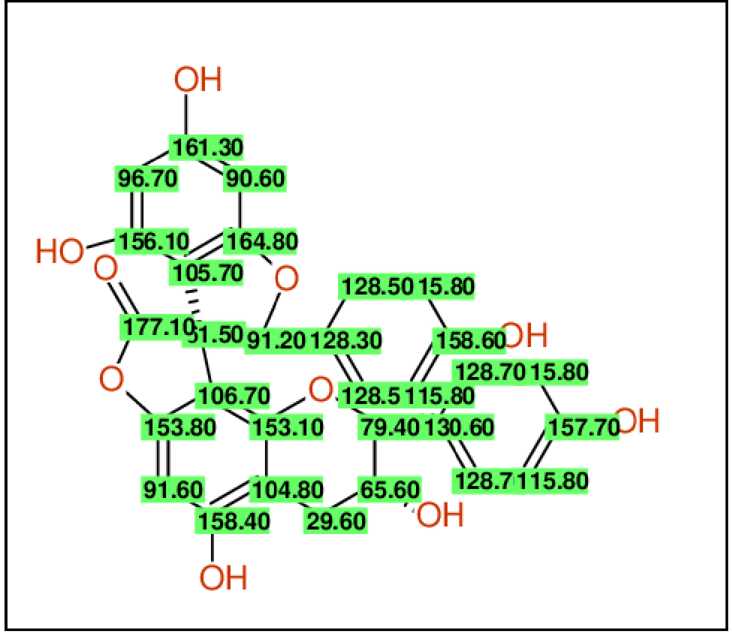


[Preferred Chemical Shift Values from both predictions]

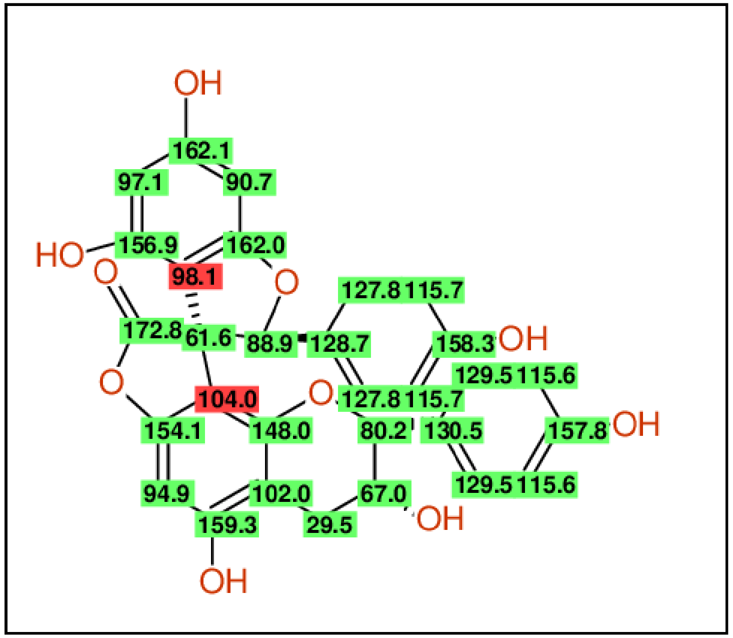


[Carbons with massive contribution from stereochemistry]

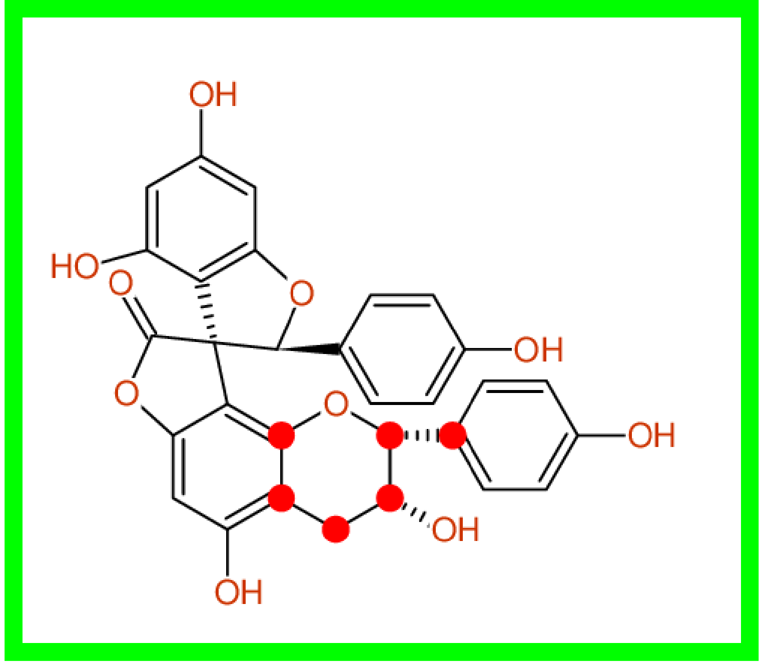


Comparison of Prediction Techniques

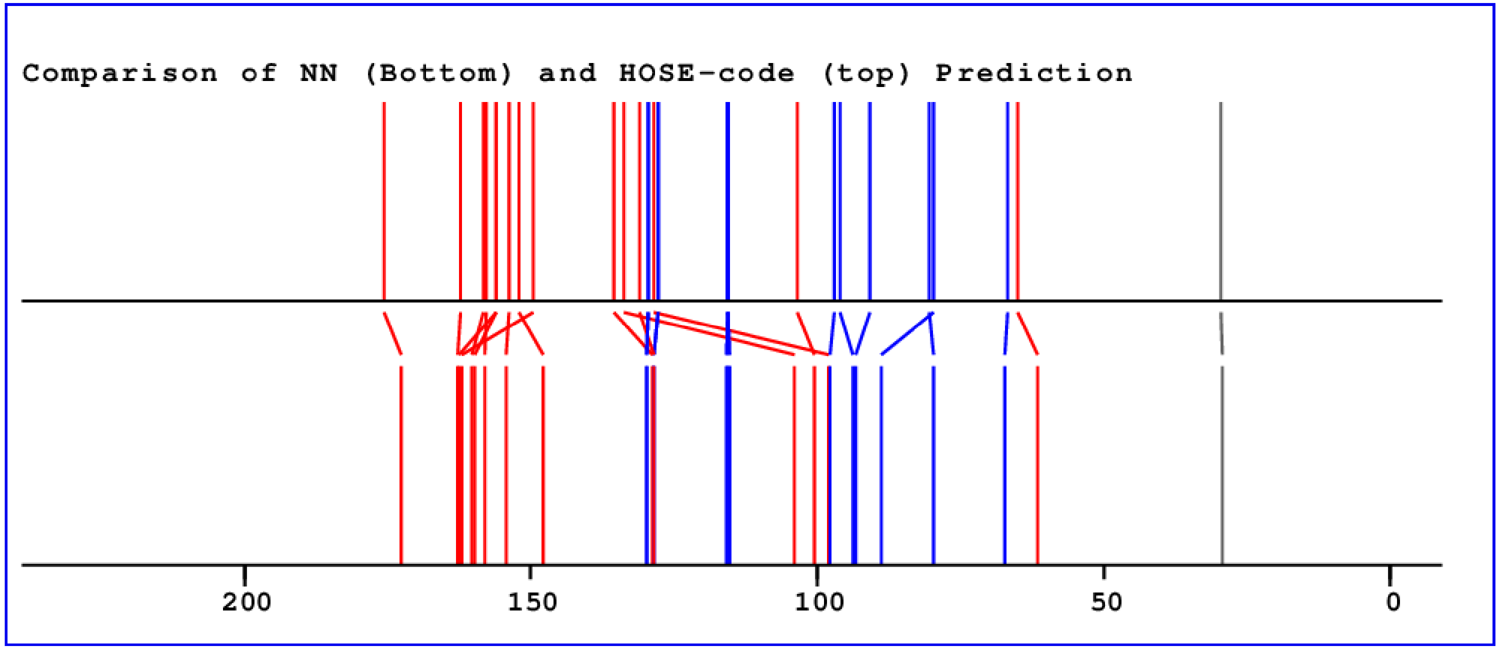


Contribution of the methods

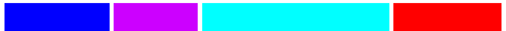

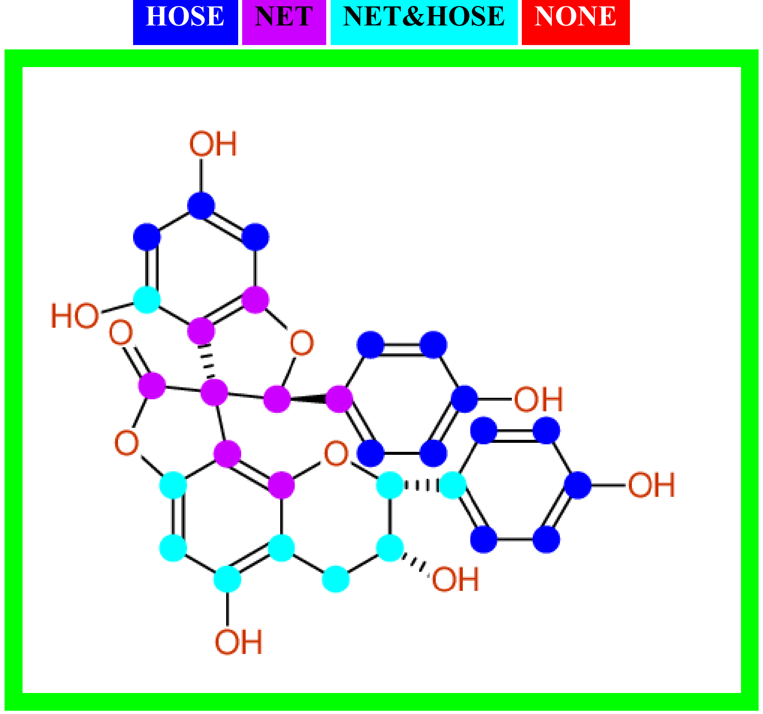


**Similarity between predicted and experimental data based on positions**

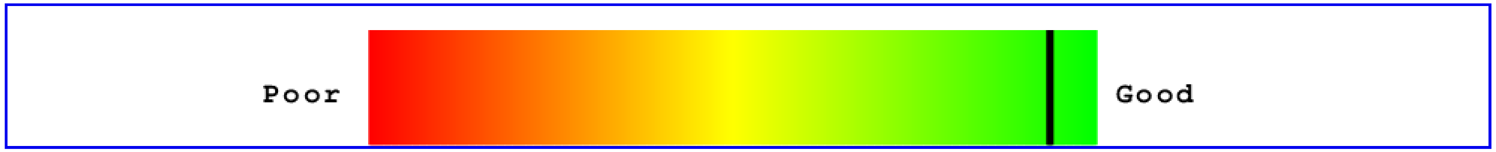


**Matching map of predicted versus experimental data**

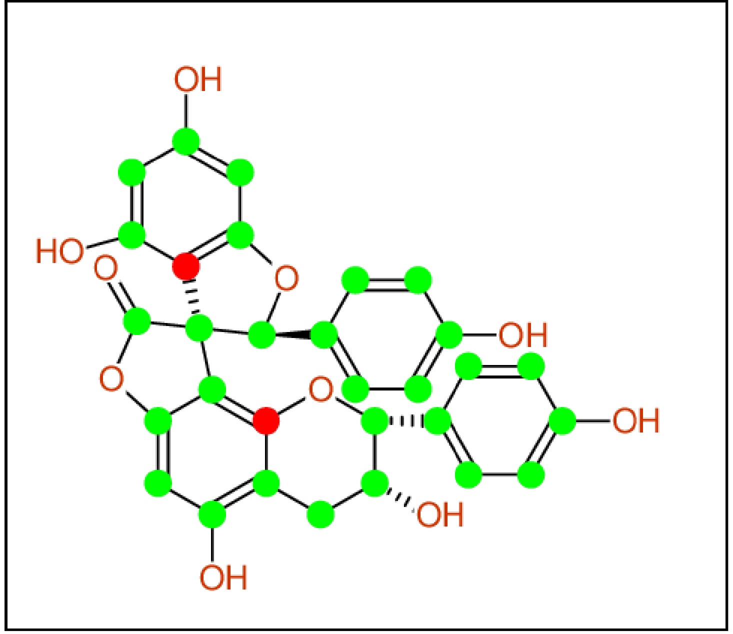


**Differences between predicted and experimental data in ppm**

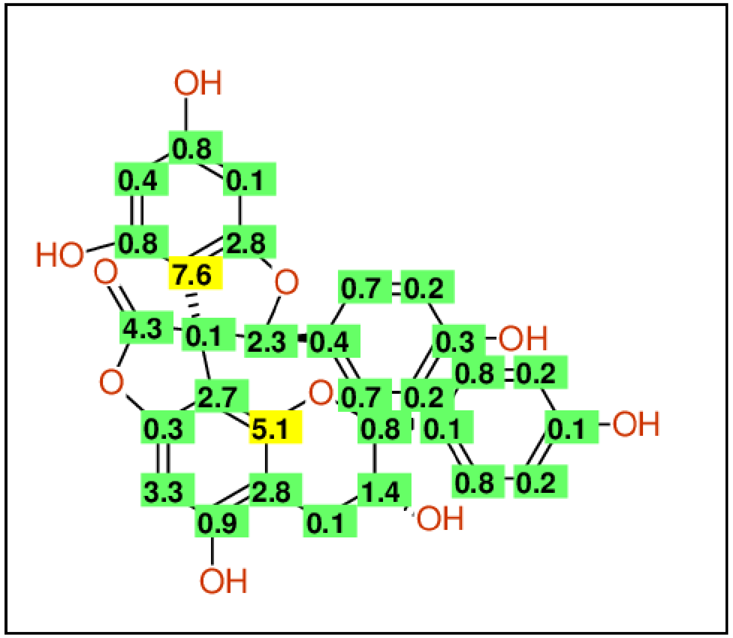


**Comparison of Experimental versus Predicted Chemical Shift Values**

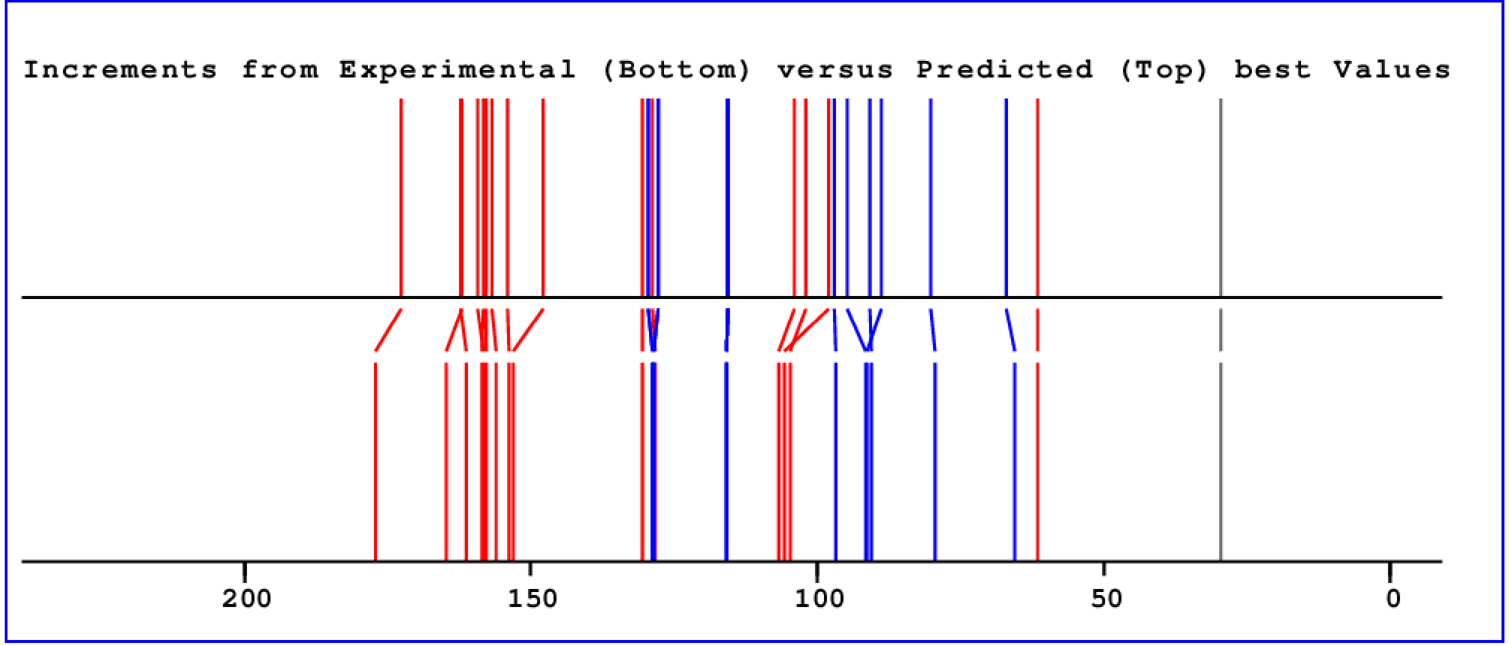


**Overall deviation between predicted and experimental data is 1.4ppm**

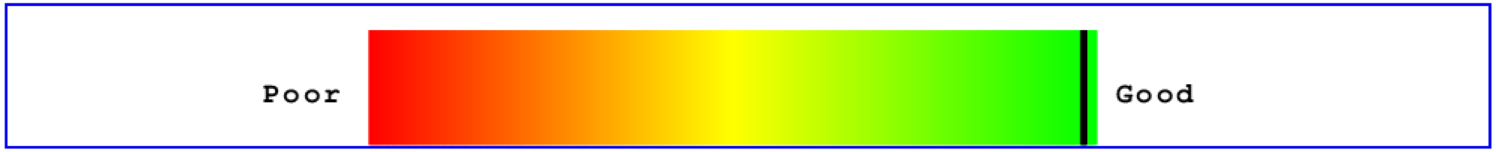


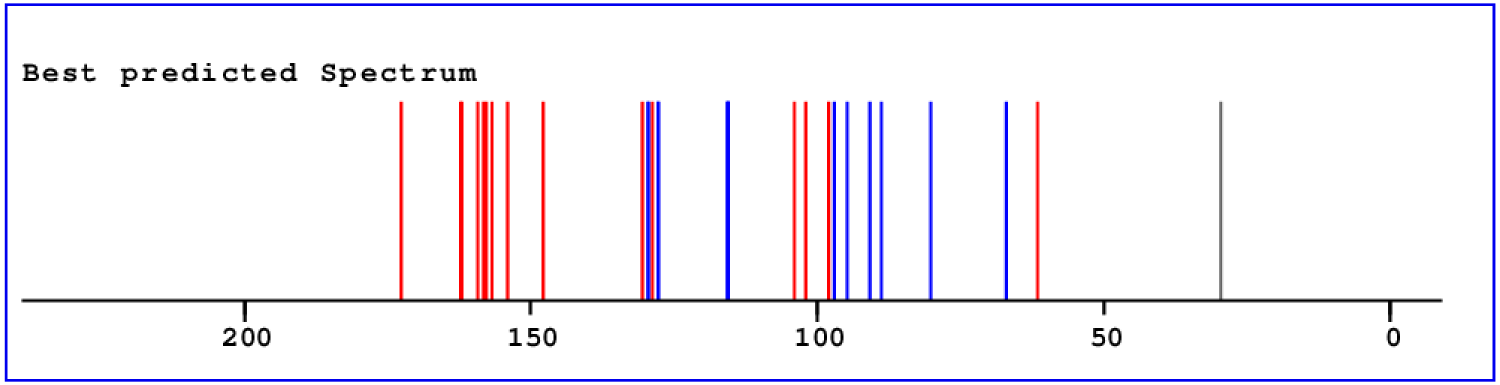

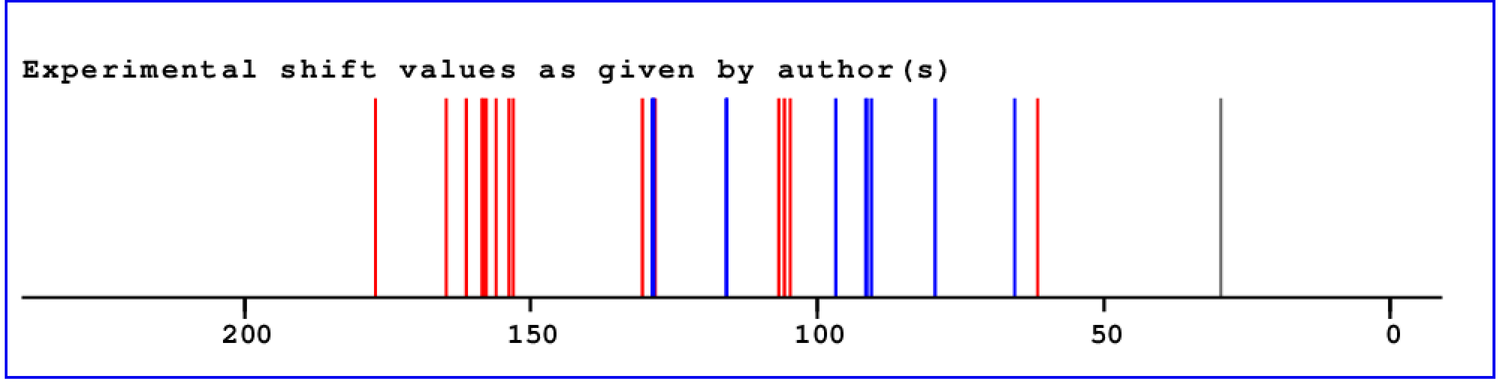


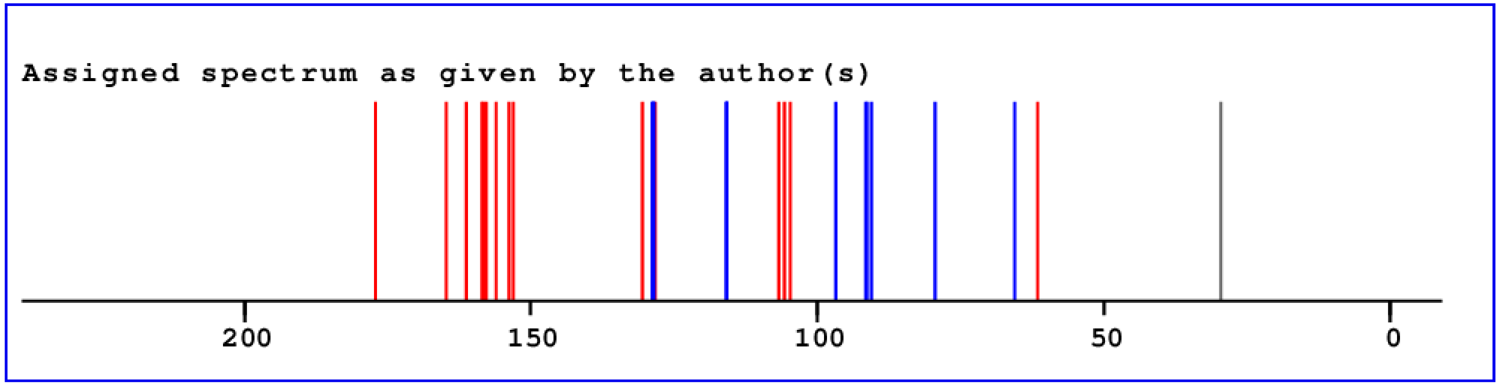


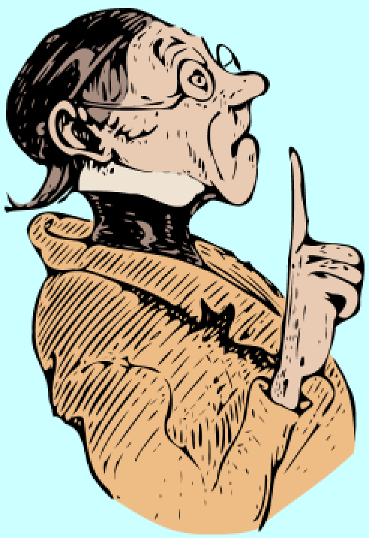

| | Your assignment | Difference to predicted values |
|---|---|---|
| | 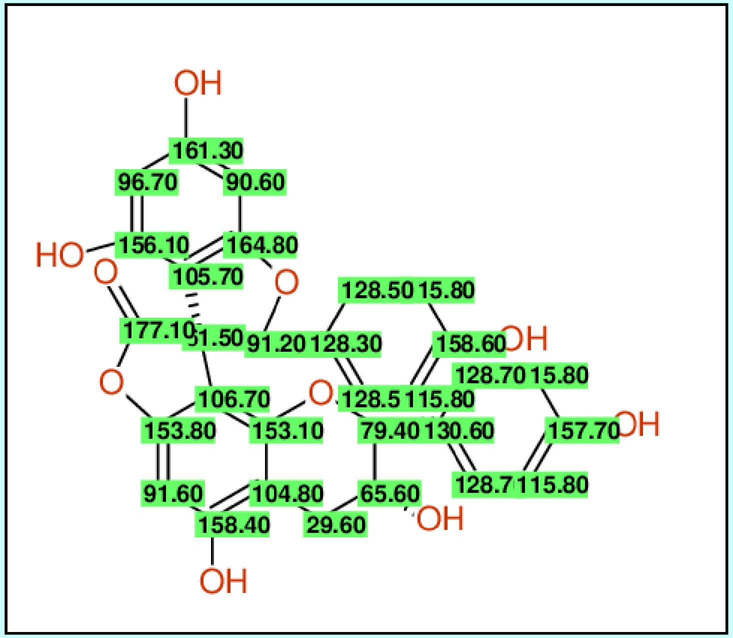 | 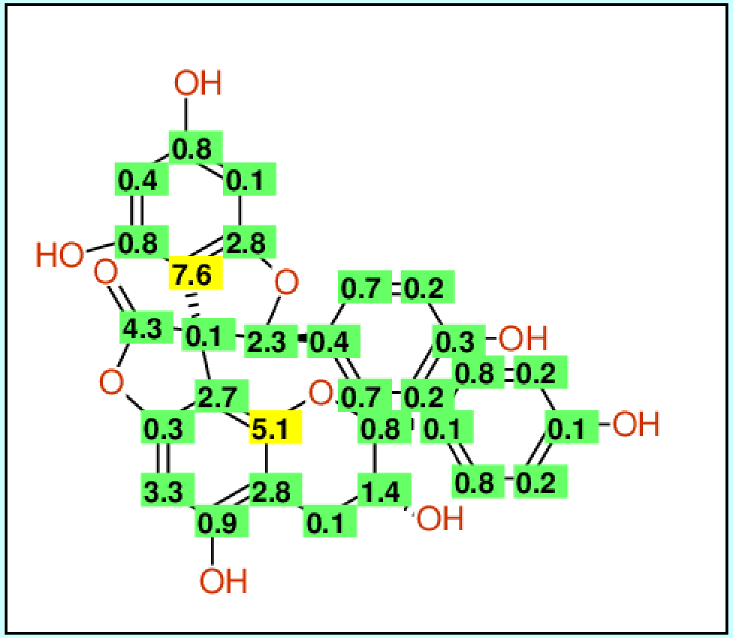 |

### Nothing found when searching CSEARCH for identical structures

(Description)

### No alternative structure found when searching CSEARCH for identical spectra

(Description)

### Overall Impression

Poor Good

**Minor revision might be necessary - please check**

Your evaluation was classified either as "Major revision" or "Reject",
therefore a similarity search using your peaklist over
74,435,185 predicted CNMR-Spectra for the PUBCHEM-Structures
has been automatically launched

[Recall Result from Spectral Similarity Search]

Compound: Yuccalechin_A

Project: YS

## The CSEARCH Robot Referee recommends: Minor revision might be necessary - please check

[Check integrity of page via electronic fingerprint]

- NN-Prediction and HOSE-Code prediction differs significantly at 6 carbon positions
- Assignment can be probably improved at 10 positions
- 2 Carbon positions ( out of 30 ) have a severe assignment problem
- Spectrum prediction - minor inconsistencies found
- 4 Carbon(s) might have a symmetry-problem

| Experimental values |
| --- |
| |

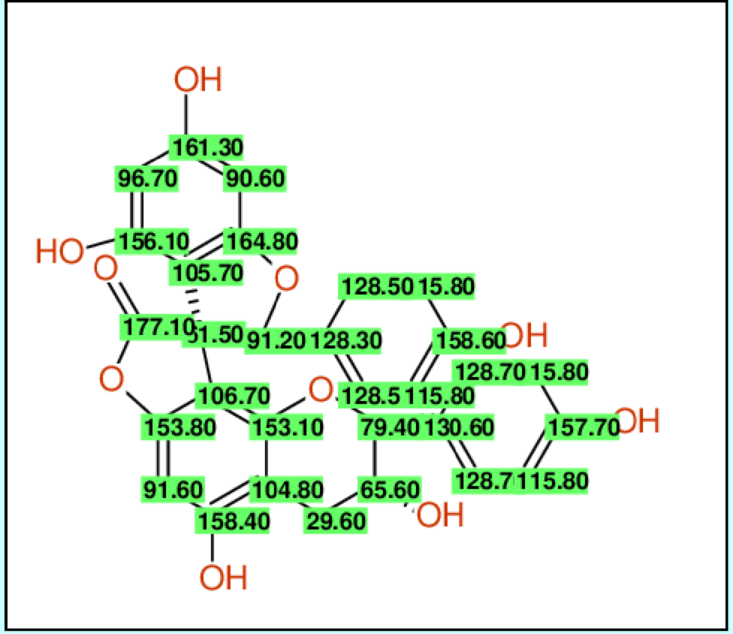


**Predicted values**

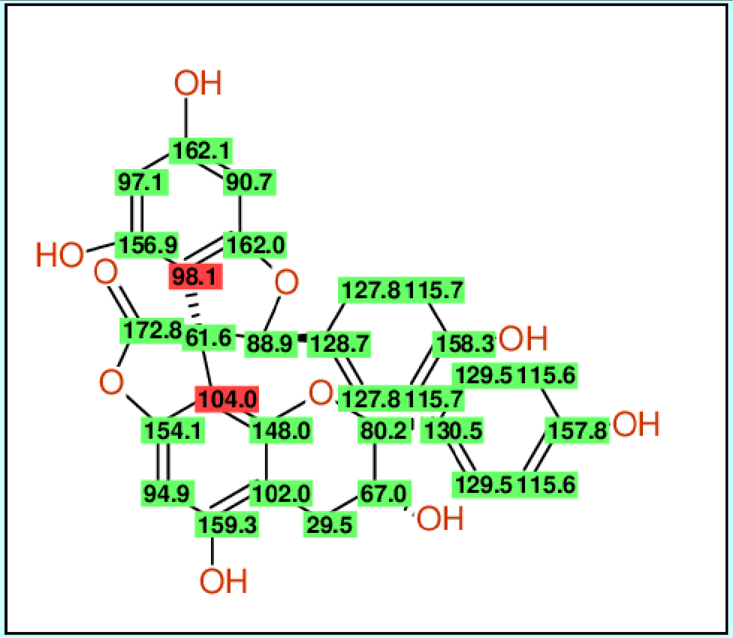


**Matching map**

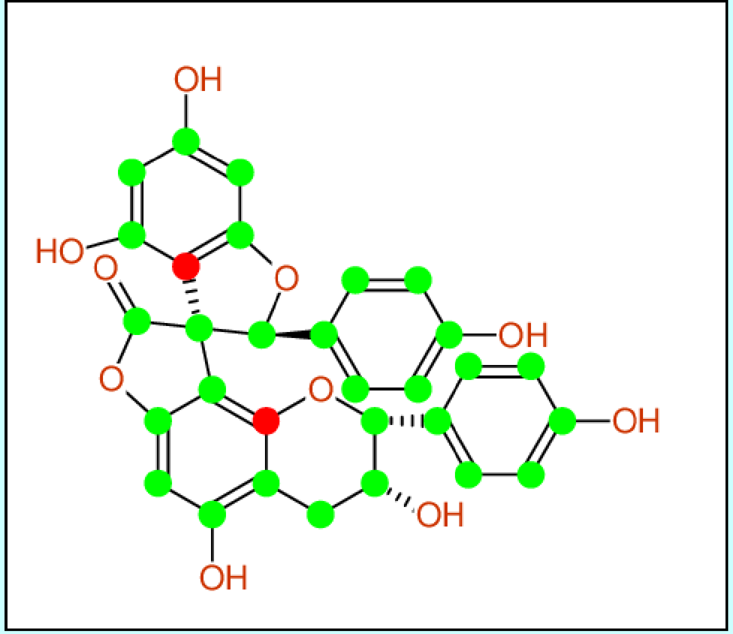


**Deviation per position ( Average is 1.4ppm )**

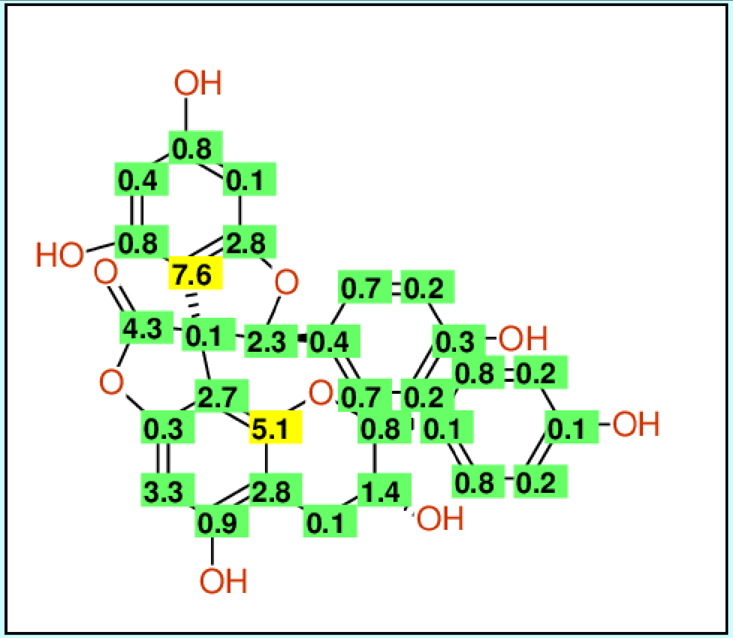


**Carbons having strong stereochemical effects**

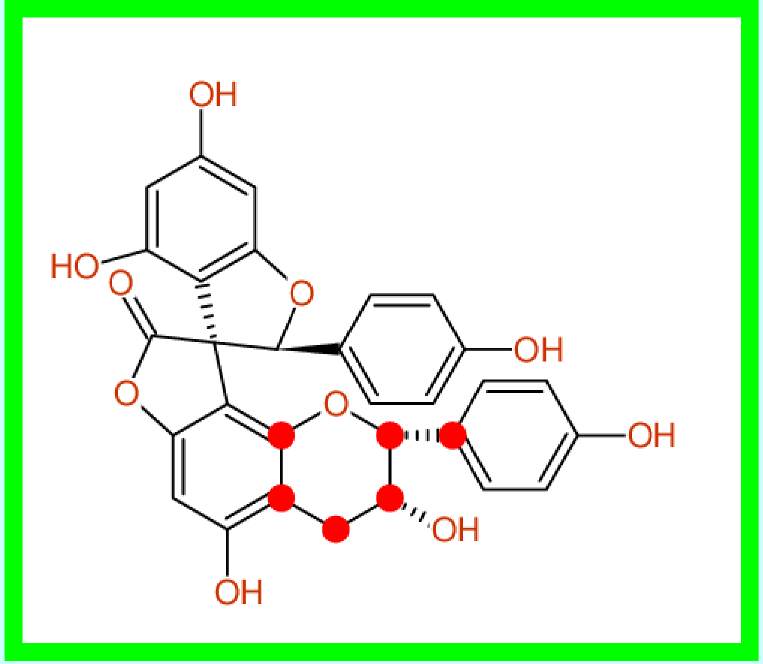


## Contribution of methods

HOSE | NET | NET&HOSE | NONE

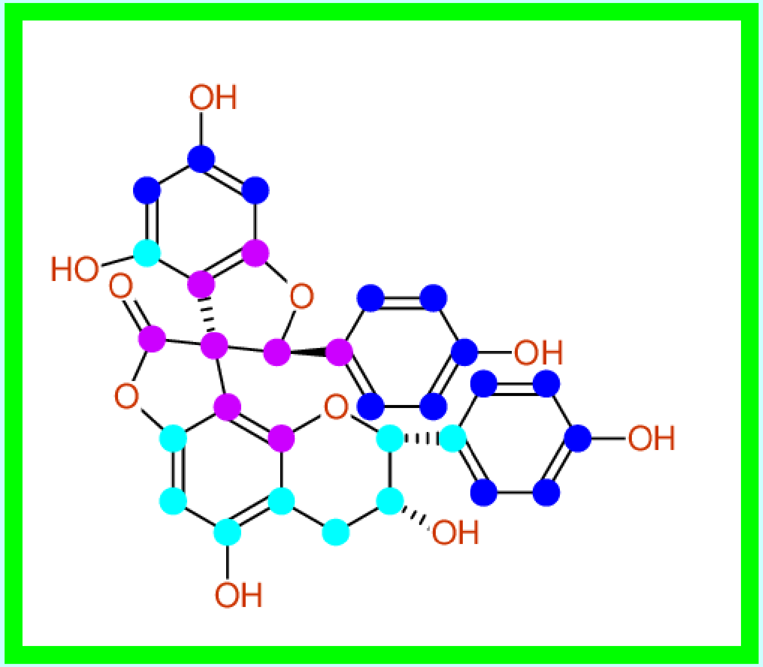


## Overall Similarity Index is 1.6

**0.0 is a "perfect match", up to approximately 3.0 it is "reasonable", above 5.0 it is more or less "unbelievable"**

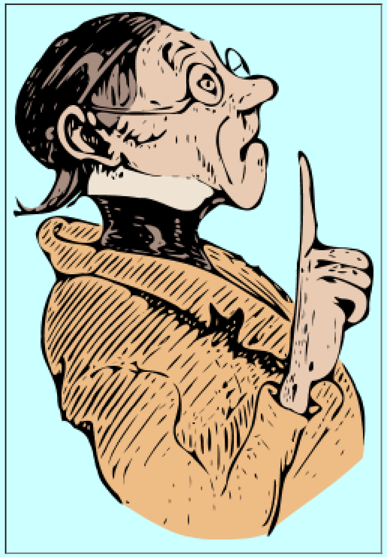

| Your assignment | Difference to predicted values |
|---|---|

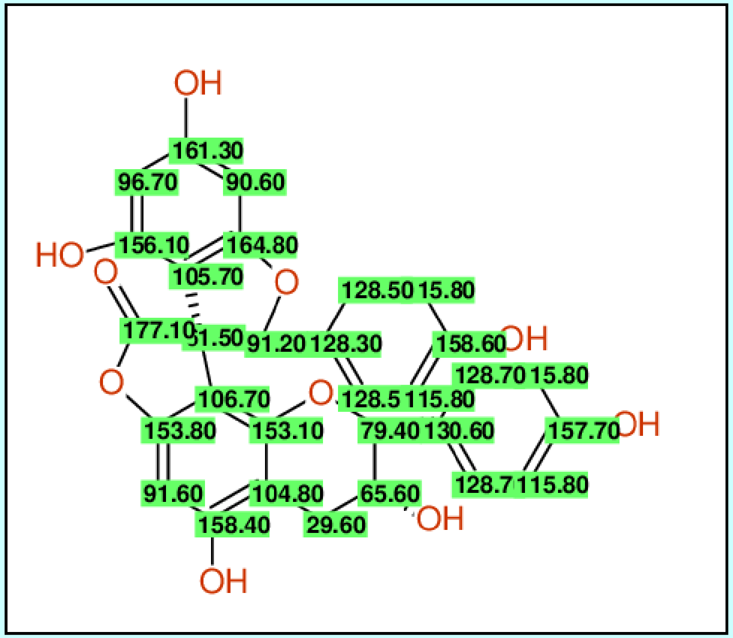



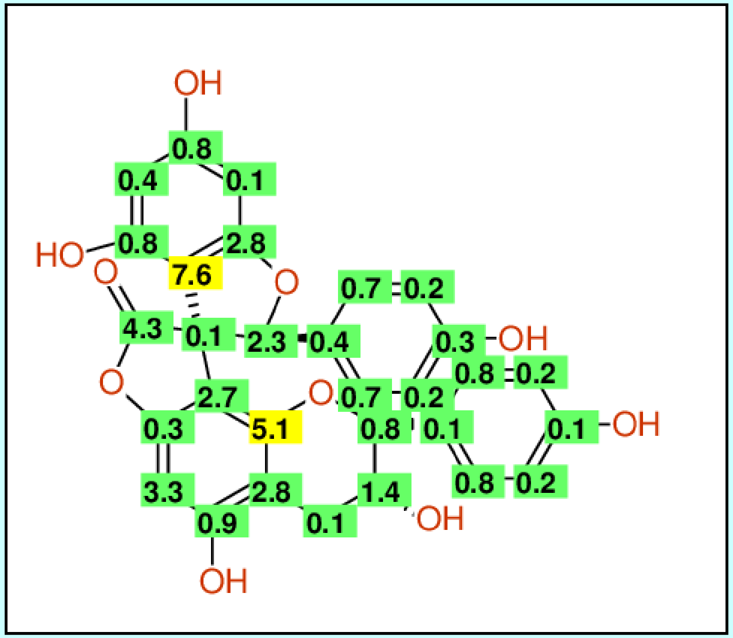



Recall this Compound from PUBCHEM ( Skeleton-Match from searching 121,500,754 compounds )
Recall this Compound from PUBCHEM ( Skeleton-Match from searching 121,500,754 compounds )
Recall this Compound from PUBCHEM ( Skeleton-Match from searching 121,500,754 compounds )
Recall this Compound from PUBCHEM ( Skeleton-Match from searching 121,500,754 compounds )
Recall this Compound from PUBCHEM ( Skeleton-Match from searching 121,500,754 compounds )
Recall this Compound from PUBCHEM ( Skeleton-Match from searching 121,500,754 compounds )
Recall this Compound from PUBCHEM ( Skeleton-Match from searching 121,500,754 compounds )

4,400,967 Compounds searched in Emolecules - nothing found

Search the Internet for this compound ( Skeleton only )
Search the Internet for this compound ( Skeleton + Stereochemistry )

Search CHEMSPIDER for this compound ( Skeleton only )
Search CHEMSPIDER for this compound ( Skeleton + Stereochemistry )

**Search the Internet for the molecular formula $C_{30}H_{22}O_{10}$**

**Search CHEMSPIDER for the molecular formula $C_{30}H_{22}O_{10}$**

(Description)

Top

**Page has been automatically written by CSEARCH**
**CPU-Usage: Evaluation needed 9.017 seconds**
**Wolfgang.Robien(at)univie.ac.at**
