# Supplementary material for: Yuccalechins A–C from the Yucca schidigera Roezl ex Ortgies Bark: Elucidation of the Relative and Absolute Configurations of Three New Spirobiflavonoids and Their Cholinesterase Inhibitory Activities
Source: Molecules. 2019 Nov 16;24(22):4162. doi: 10.3390/molecules24224162 (PMC6891570; doi:10.3390/molecules24224162)
Supplement: Supplementary file 1 [file molecules-24-04162-s001.zip › CSEARCH Evaluation Report_Yuccalechin_B.pdf]

Automatic Evaluation Report from CSEARCH
created on 2019-10-23 at 15:45:27
based on 340,554 reference spectra

Did you know ?

Whenever using your private database, your request will be evaluated twice.
The first evaluation will be based on the CSEARCH-data and your private data,
the second evaluation will be based only on the CSEARCH-data.

Request from: lpecio@iung.pulawy.pl

Compound: Yuccalechin_B

Project: YS

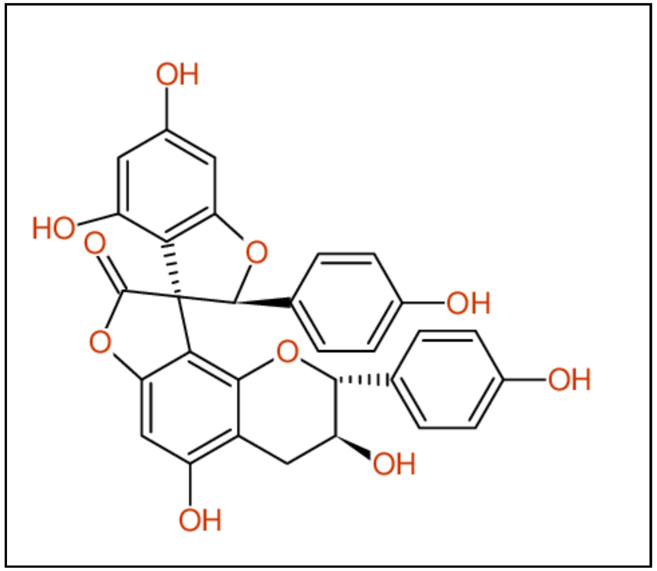

Database	Number of Entries	Owner of Database
Please cite the CSEARCH-Robot-Referee as: N. Haider, W. Robien; http://nmrpredict.orc.univie.ac.at/c13robot/robot.php		
	74,997 (A)	CSEARCH-Data / Wolfgang Robien

CSEARCH		
CSEARCH	56,549 (B)	CSEARCH-Data / Wolfgang Robien
CSEARCH	28,196 (C)	CSEARCH-Data / Wolfgang Robien
CSEARCH	33,587 (D)	CSEARCH-Data / Wolfgang Robien
CSEARCH	39,132 (E)	CSEARCH-Data / Wolfgang Robien + NMR-Database University of Mainz
CSEARCH	26,196 (F)	CSEARCH-Data / Wolfgang Robien
CSEARCH	50,594 (I)	Upcoming CSEARCH-Data / Wolfgang Robien
CSEARCH	31,307 (L)	NMRShiftDB-Data / Version February 2012

Permanent URL

This page can be verified by a digital signature

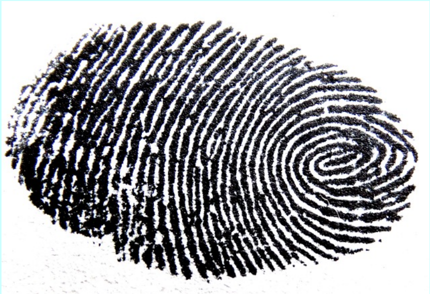

CSEARCH-Version: 9.4.0
Robot-Referee: 2017:06:10

Request from: lpecio@iung.pulawy.pl

Compound: Yuccalechin_B

Accept Minor revision CSEARCH Robot-Referee Major revision Reject

Project: YS

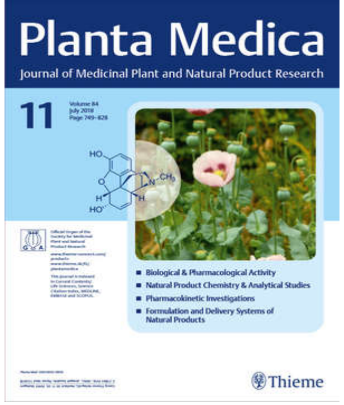

Recommendation given here

Details of Prediction given here

Summary of Supplied Data

Understanding the Color Coding Scheme

Structure Proposal

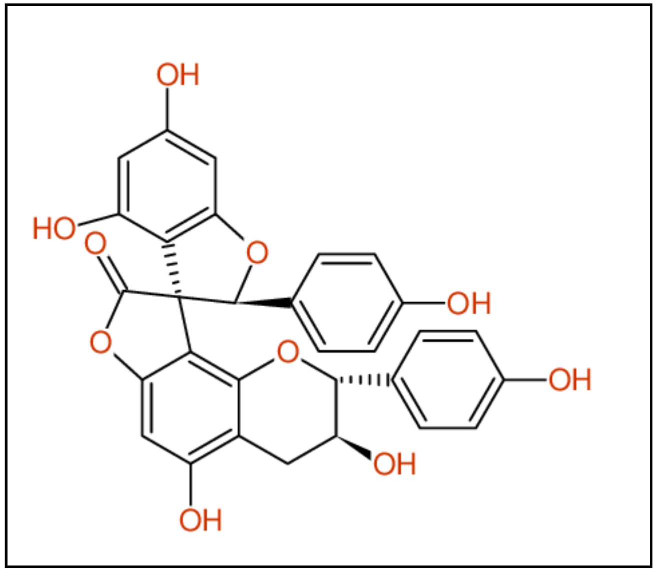

Molecular formula is: $C_{30}H_{22}O_{10}$ Molecular weight is: 542.51 amu

INCHIKEY is: RNDNBGULZNCSNB-PXRMNYBDBC

Numbering Scheme derived from the drawing sequence used during the calculation

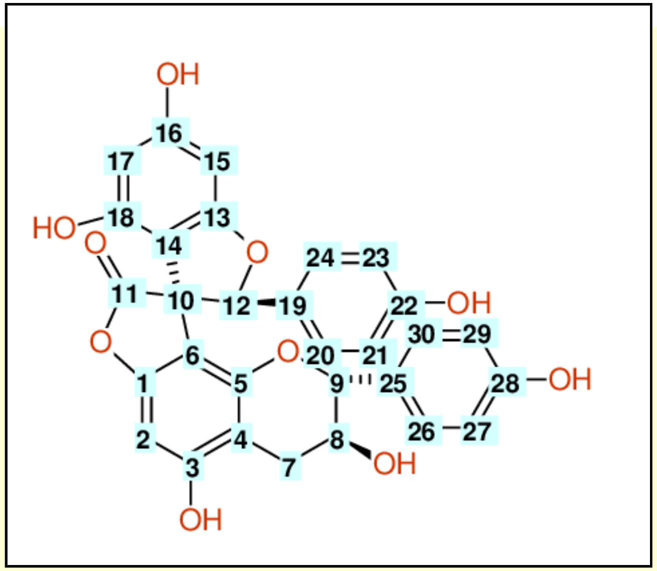

The marked carbons have been fully assigned

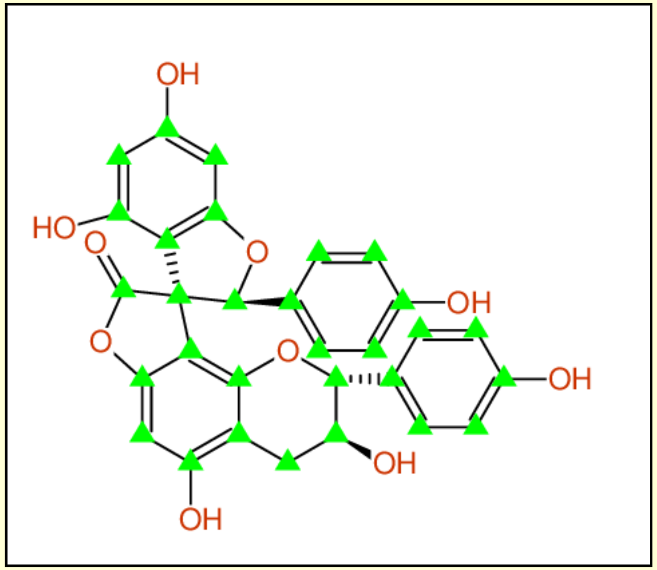

Carbon number	Chemical Shift Value	Multiplicity from Structure	Multiplicity from Experiment

1	154.10	S	S
2	91.50	D	D
3	158.00	S	S
4	104.90	S	S
5	152.10	S	S
6	105.80	S	S
7	26.80	T	T
8	68.10	D	D
9	81.60	D	D
10	61.50	S	S
11	177.10	S	S
12	90.60	D	D
13	164.60	S	S
14	105.80	S	S
15	90.80	D	D
16	161.40	S	S
17	96.70	D	D
18	156.20	S	S
19	128.10	S	S
20	128.40	D	D
21	115.80	D	D
22	158.60	S	S
23	115.80	D	D
24	128.40	D	D
25	131.20	S	S
26	128.20	D	D
27	115.90	D	D
28	157.80	S	S
29	115.90	D	D
30	128.20	D	D

The marked carbons have been fully assigned

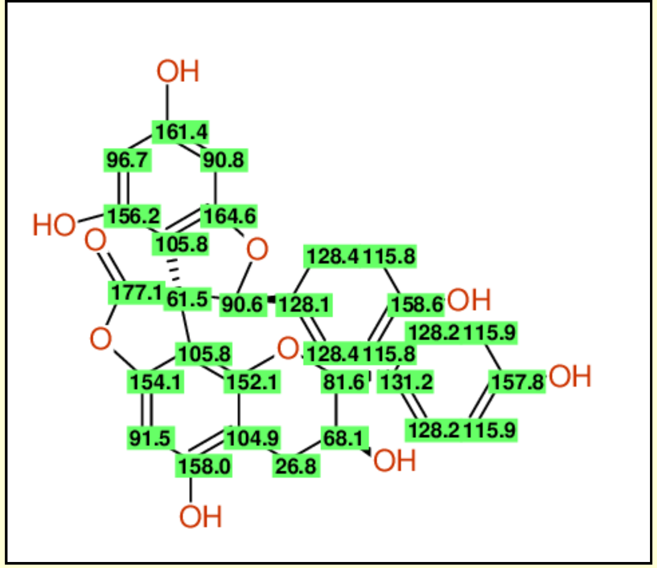

Graphical summary of the Chemical Shift Data

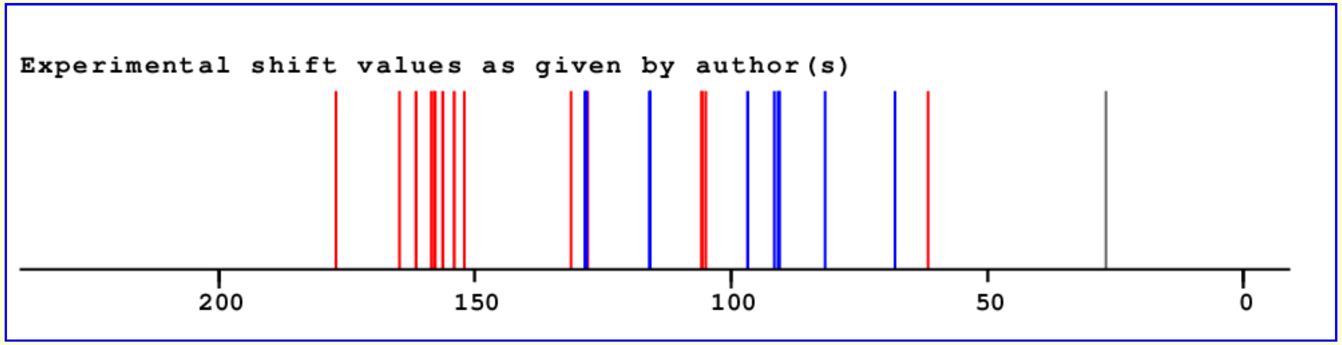

Searching external databases

Recall this Compound from PUBCHEM (Stereo-Match from searching 121,500,754 compounds)

4,400,967 Compounds searched in Emolecules - nothing found

Search the Internet for this compound (Skeleton only)
Search the Internet for this compound (Skeleton + Stereochemistry)

Search CHEMSPIDER for this compound (Skeleton only)
Search CHEMSPIDER for this compound (Skeleton + Stereochemistry)

Search the Internet for the molecular formula $C_{30}H_{22}O_{10}$

Search CHEMSPIDER for the molecular formula $C_{30}H_{22}O_{10}$

(Description)

Performing Symmetry Analysis

Eventually Symmetry Error: Same shiftvalue - Different environment

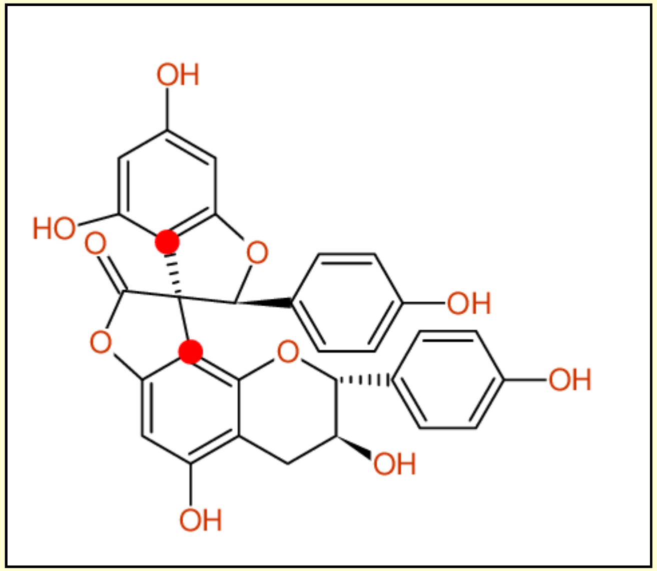

Basic Evaluation: Checking Multiplicities

Checking lines & multiplicity	Carbons/Lines	Singlet	Dublet	Triplet	Quartet	Odd	Even	None
From structure	30	15	14	1	0	16	14	0
From spectrum	30	15	14	1	0	16	14	0

[Overall impression on compatibility of multiplicity from structure and experiment]

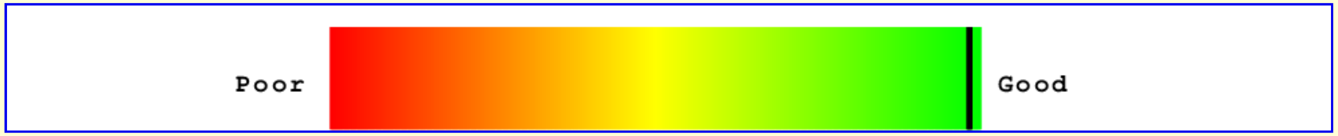

Evaluation based on Spectrum Prediction

[Numbering Scheme]

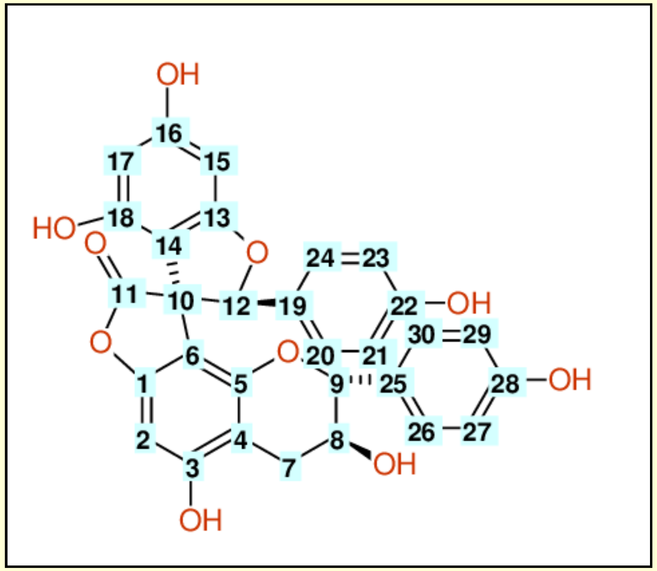

Carbon Number ▲ ▼	Neural Network Prediction ▲ ▼	HOSE-Code Prediction ▲ ▼	Preferred Value from both Predictions ▲ ▼	Experimental values ▲ ▼	Difference (Exp-Pred/ppm) ▲ ▼	Assignment	Prediction Quality

Carbon Number ▲ ▼	Neural Network Prediction ▲ ▼	HOSE-Code Prediction ▲ ▼	Preferred Value from both Predictions ▲ ▼	Experimental values ▲ ▼	Difference (Exp-Pred/ppm) ▲ ▼	Assignment	Prediction Quality
1	154.4	153.9	154.1	154.1	0.0	Assigned by author	
2	93.8	95.9	94.9	91.5	3.4	Assigned by author	Only reference material with low similarity
3	162.5	156.1	159.3	158.0	1.3	Assigned by author	Large Difference between NET & HOSE
4	102.4	103.5	103.0	104.9	1.9	Assigned by author<br>Check assignment - maybe 105.80 ?	
5	149.0	154.2	149.0	152.1	3.1	Assigned by author	Large Difference between NET & HOSE<br>Only reference material with low similarity
6	104.0	133.8	104.0	105.8	1.8	Assigned by author<br>Check assignment - maybe 104.90 ?	Large Difference between NET & HOSE<br>Only reference material with low similarity
7	28.5	28.5	28.5	26.8	1.7	Assigned by author	
8	69.5	68.1	68.4	68.1	0.3	Assigned by author	
9	83.1	83.4	83.4	81.6	1.8	Assigned by author	
10	61.6	65.0	61.6	61.5	0.1	Assigned by author	Only reference material with low similarity<br>Only very few similar structures
11	172.8	175.6	172.8	177.1	4.3	Assigned by author	Only reference material with low similarity
12	88.9	79.7	88.9	90.6	1.7	Assigned by author<br>Check assignment - maybe 90.80 ?	Large Difference between NET & HOSE<br>Only reference material with low similarity
13	162.0	149.6	162.0	164.6	2.6	Assigned by author<br>Check assignment - maybe 161.40 ?	Large Difference between NET & HOSE<br>Only reference material with low similarity
14	98.1	128.4	98.1	105.8	7.7	Assigned by author	Large Difference between NET & HOSE<br>Only reference material with low similarity
15	93.4	90.7	90.7	90.8	0.1	Assigned by author<br>Check assignment - maybe 90.60 ?	
16	162.8	162.1	162.1	161.4	0.8	Assigned by author<br>Check assignment - maybe 164.60 ?	
17	97.8	97.1	97.1	96.7	0.4	Assigned by author	
18	159.7	156.1	156.9	156.2	0.7	Assigned by author	Only very few similar structures
19	128.7	135.4	128.7	128.1	0.6	Assigned by author<br>Check assignment - maybe 128.40 ?	Large Difference between NET & HOSE<br>Only reference material with low similarity
20	128.5	127.8	127.8	128.4	0.6	Assigned by author<br>Check assignment - maybe 128.10 ?	
21	115.3	115.7	115.7	115.8	0.2	Assigned by author	
22	160.2	158.3	158.3	158.6	0.3	Assigned by author	
23	115.3	115.7	115.7	115.8	0.2	Assigned by author	
24	128.5	127.8	127.8	128.4	0.6	Assigned by author<br>Check assignment - maybe 128.10 ?	
25	131.1	130.5	130.6	131.2	0.6	Assigned by author	
26	129.7	129.5	129.5	128.2	1.3	Assigned by author<br>Check assignment - maybe 128.40 ?	
27	116.0	115.6	115.6	115.9	0.3	Assigned by author	
28	158.1	157.8	157.8	157.8	0.0	Assigned by author	
29	116.0	115.6	115.6	115.9	0.3	Assigned by author	
30	129.7	129.5	129.5	128.2	1.3	Assigned by author<br>Check assignment - maybe 128.40 ?	
Absolute<br>Signed	1.69ppm (30)<br>-0.05ppm (30)	3.65ppm (30)<br>-1.37ppm (30)	1.32ppm (30)<br>0.42ppm (30)			1.27ppm (30)<br>0.42ppm (30)	Average deviation to experimental values<br>(Number of shift pairs used)

Carbon Number ▲ ▼	Neural Network Prediction ▲ ▼	HOSE-Code Prediction ▲ ▼	Preferred Value from both Predictions ▲ ▼	Experimental values ▲ ▼	Difference (Exp-Pred/ppm) ▲ ▼	Assignment	Prediction Quality
Structure representation by reference data over 3.3 shells on average							

Visualization of the differences between predicted and experimental values

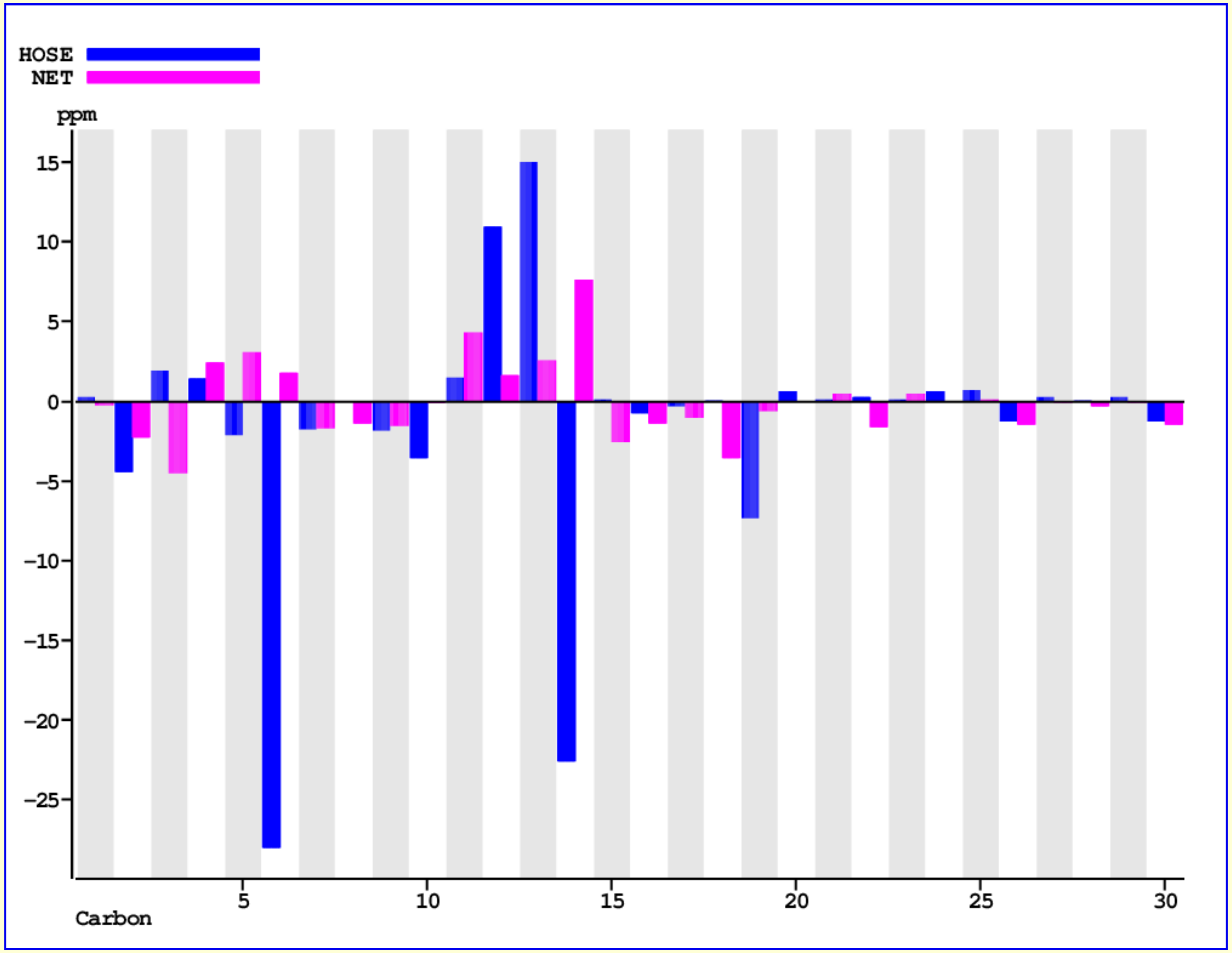

Quality of the Spectrum Prediction

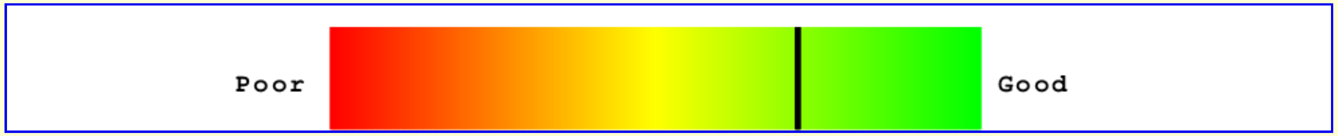

Experimental Chemical Shift Values as given

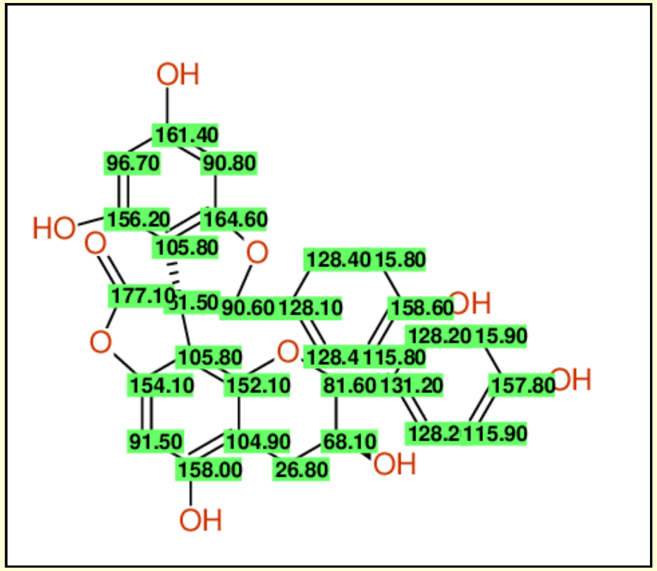

Experimental Chemical Shift Values using Symmetry

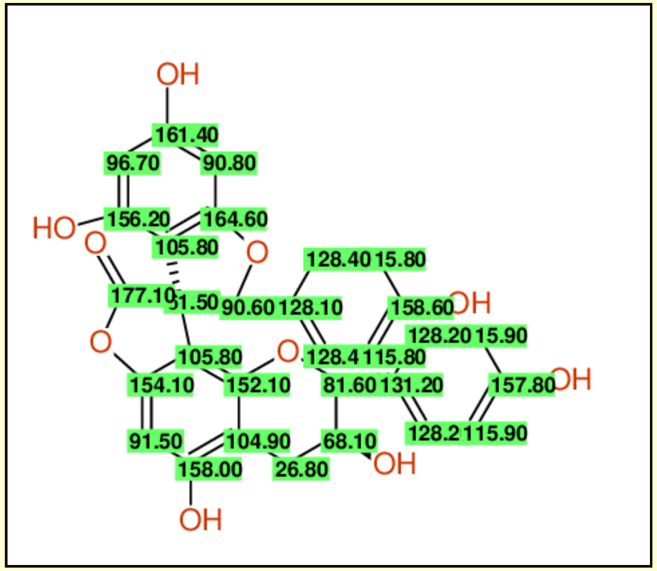

Preferred Chemical Shift Values from both predictions

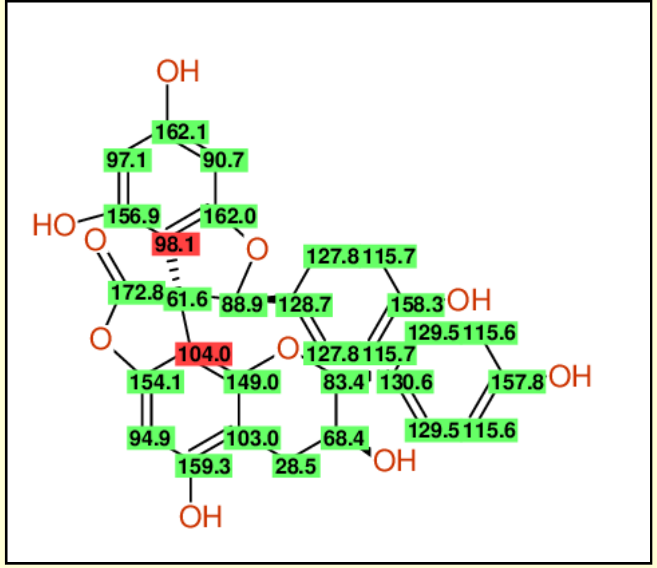

[Carbons with massive contribution from stereochemistry]

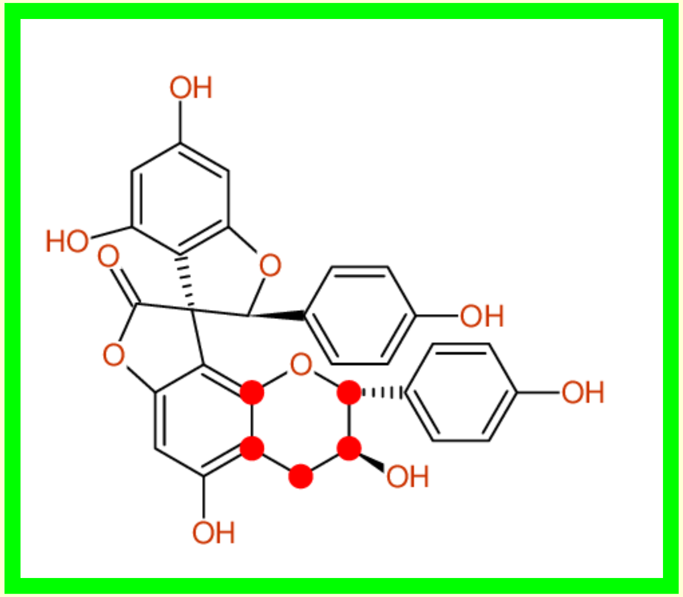

[Comparison of Prediction Techniques]

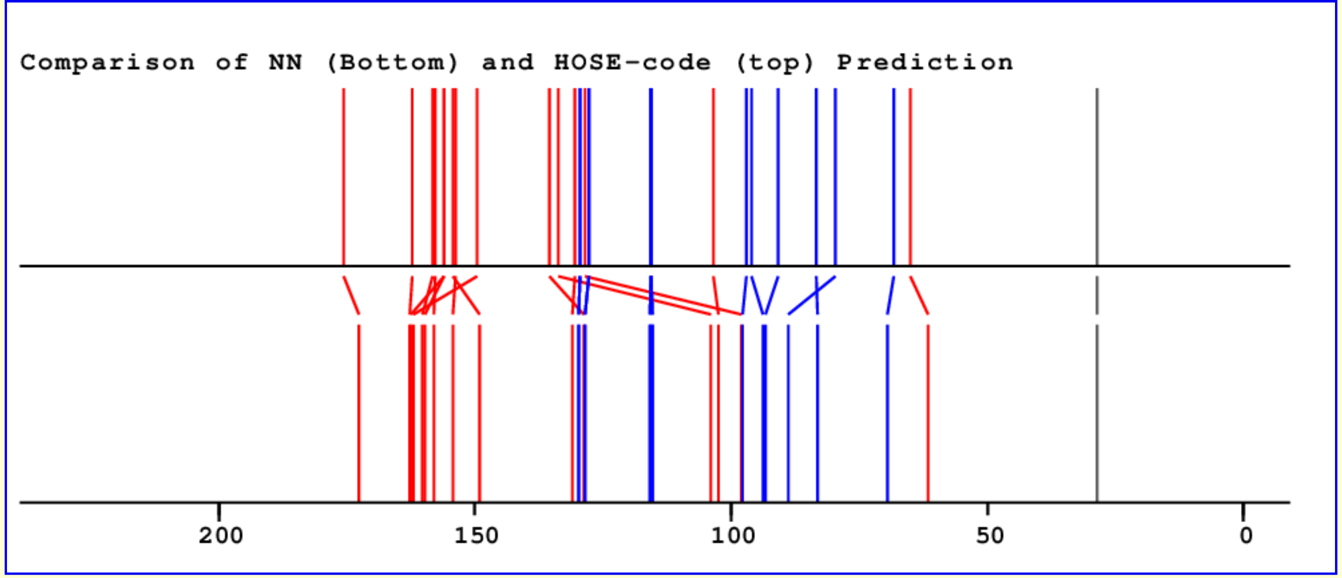

Contribution of the methods

HOSE NET NET&HOSE NONE

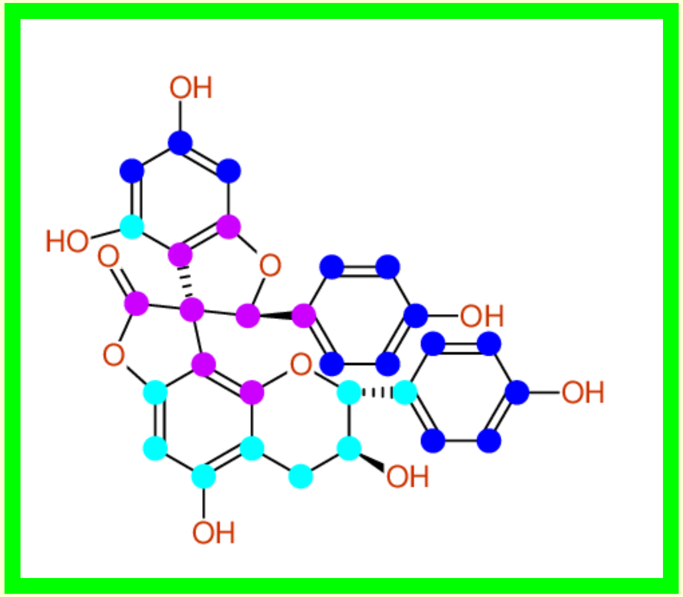

Similarity between predicted and experimental data based on positions

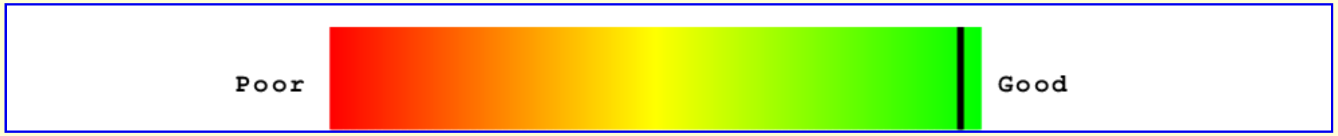

[Matching map of predicted versus experimental data]

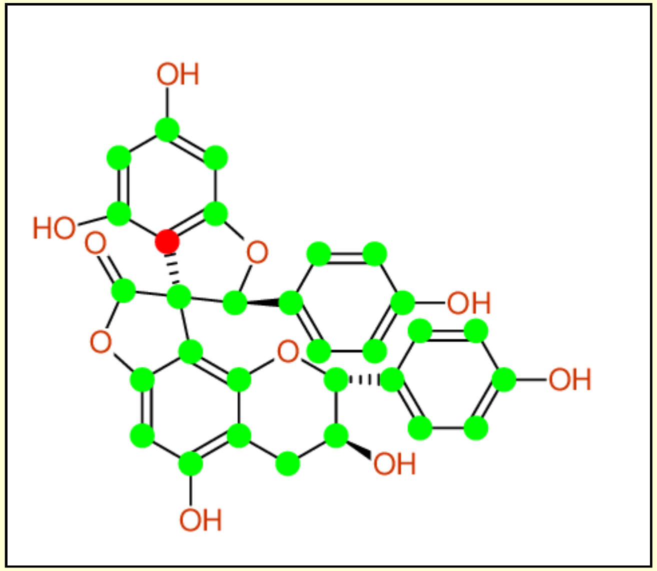

[Differences between predicted and experimental data in ppm]

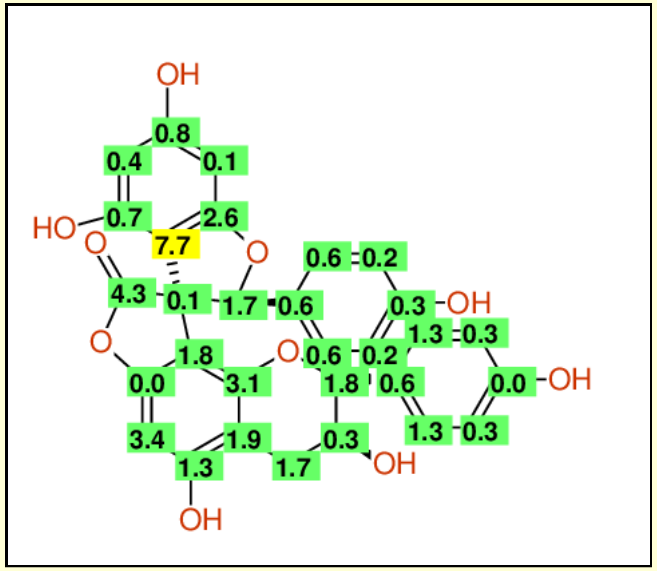

[Comparison of Experimental versus Predicted Chemical Shift Values]

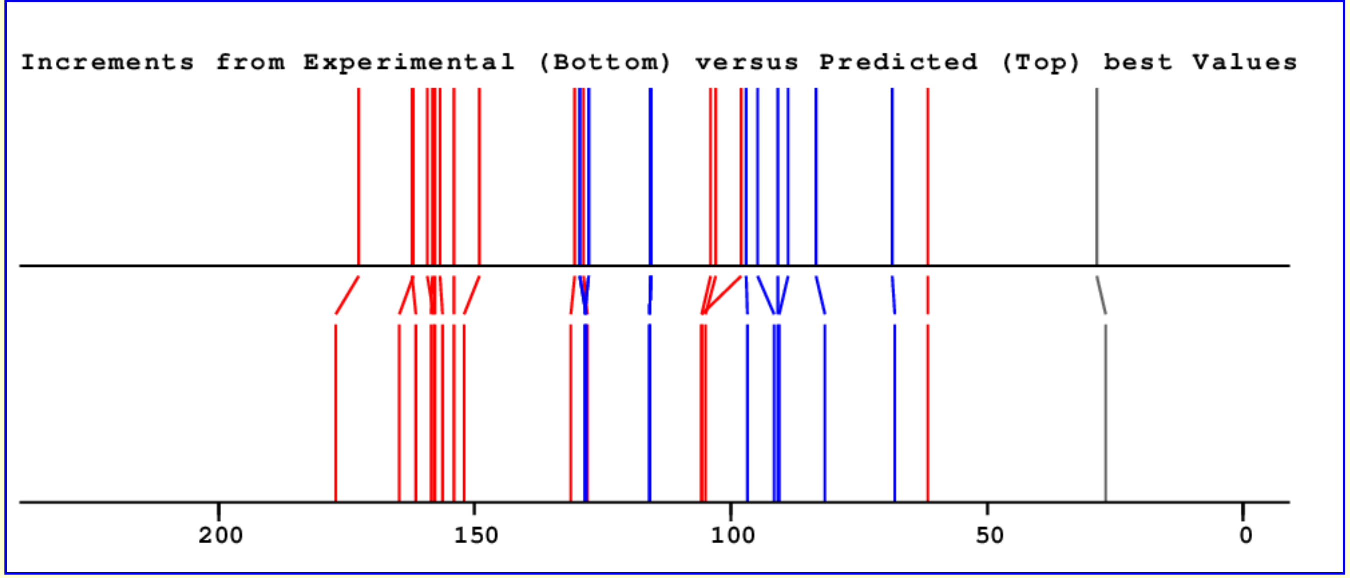

Overall deviation between predicted and experimental data is 1.3ppm

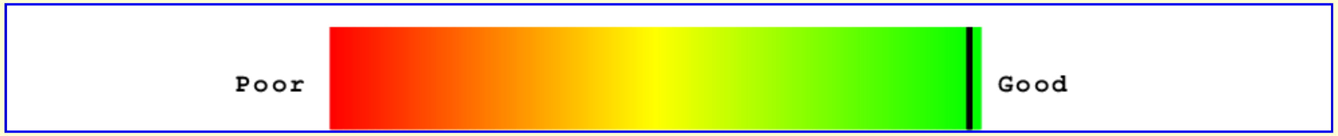

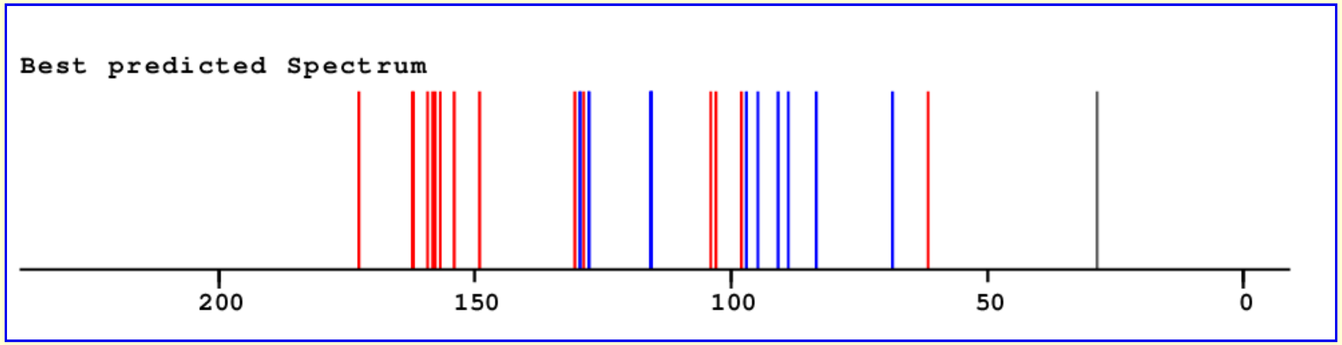

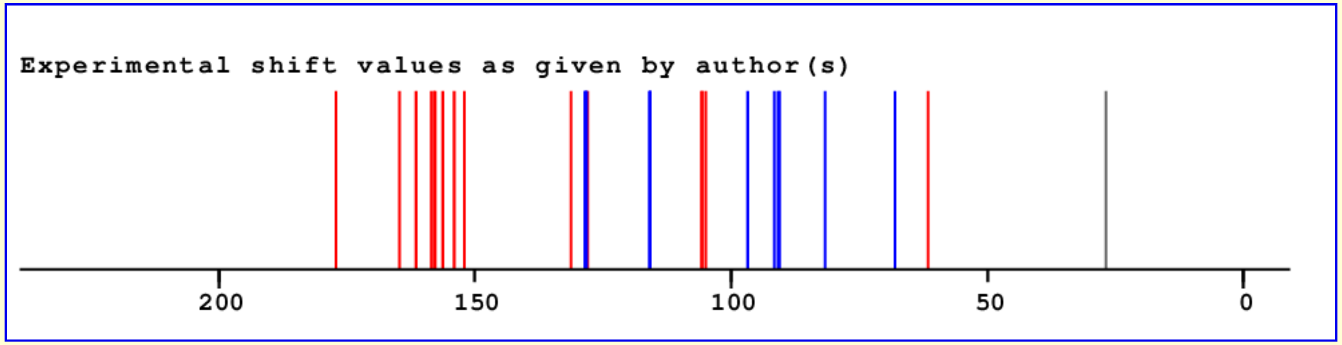

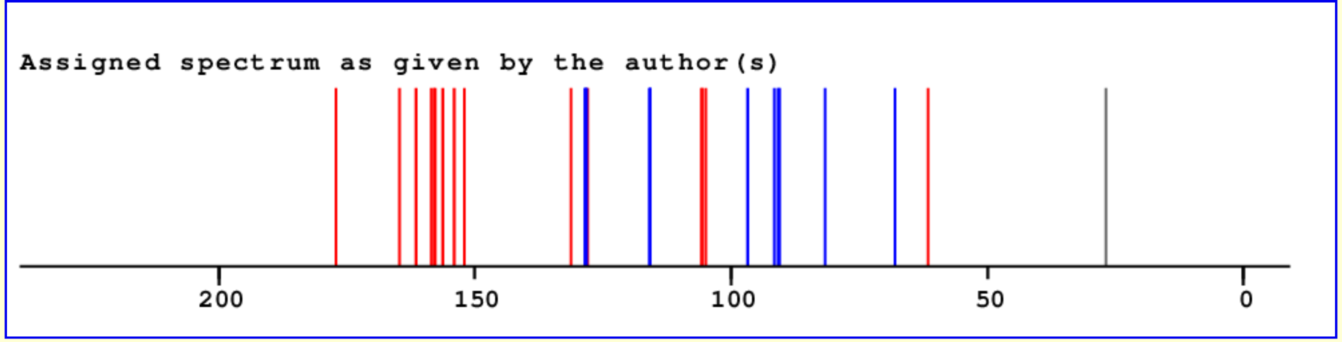

	Your assignment	Difference to predicted values
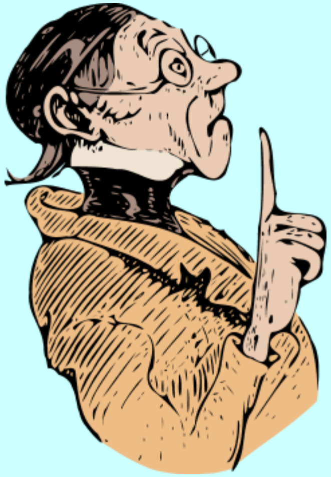	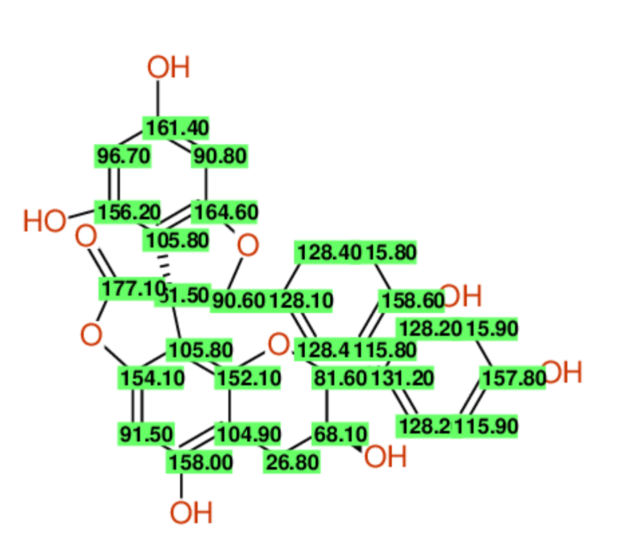	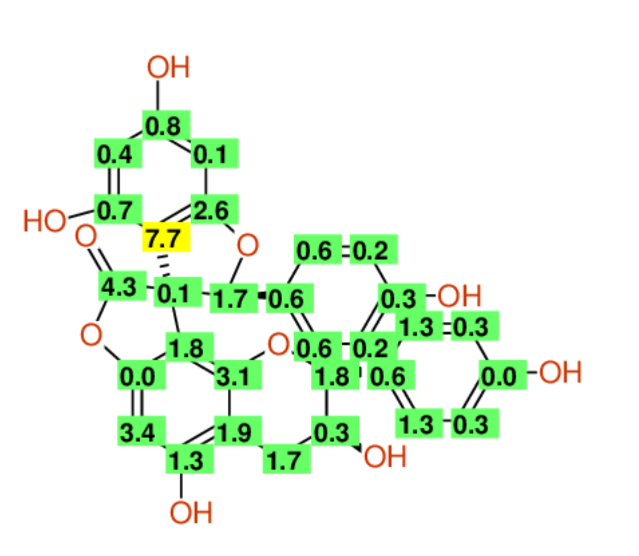

Nothing found when searching CSEARCH for identical structures

(Description)

No alternative structure found when searching CSEARCH for identical spectra

(Description)

Overall Impression

Poor Good

Minor revision might be necessary - please check

Your evaluation was classified either as "Major revision" or "Reject",
therefore a similarity search using your peaklist over
74,435,185 predicted CNMR-Spectra for the PUBCHEM-Structures
has been automatically launched

Recall Result from Spectral Similarity Search

Compound: Yuccalechin_B

Project: YS

The CSEARCH Robot Referee recommends: Minor revision might be necessary - please check

Check integrity of page via electronic fingerprint

- NN-Prediction and HOSE-Code prediction differs significantly at 7 carbon positions
- Assignment can be probably improved at 11 positions
- 1 Carbon position (out of 30) has a severe assignment problem
- Spectrum prediction - minor inconsistencies found
- 2 Carbon(s) might have a symmetry-problem

Experimental values
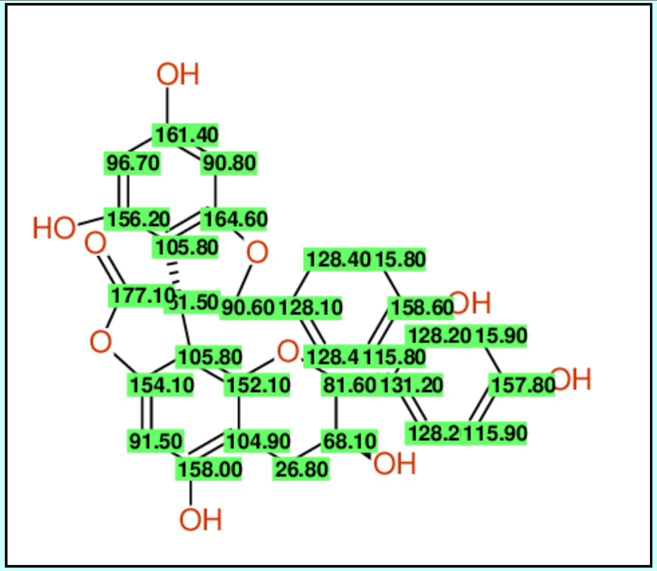
Predicted values

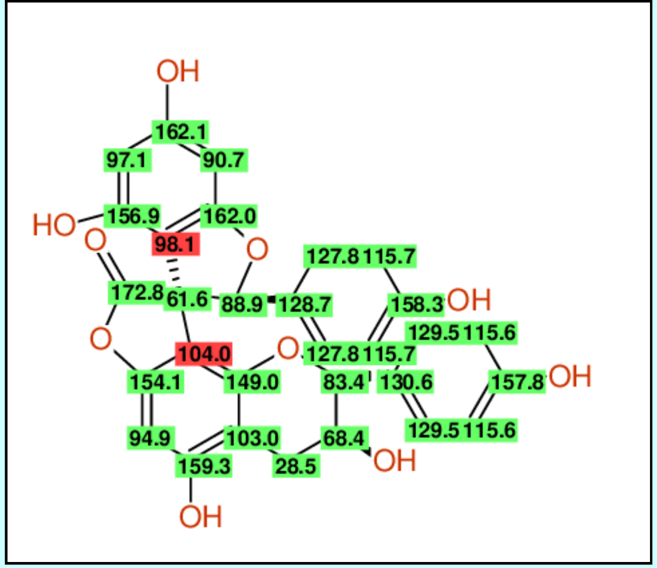

Matching map

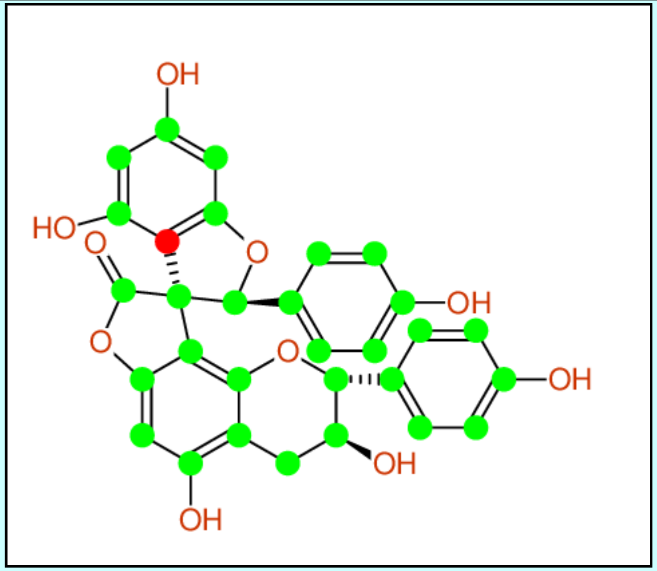

Deviation per position (Average is 1.3ppm)

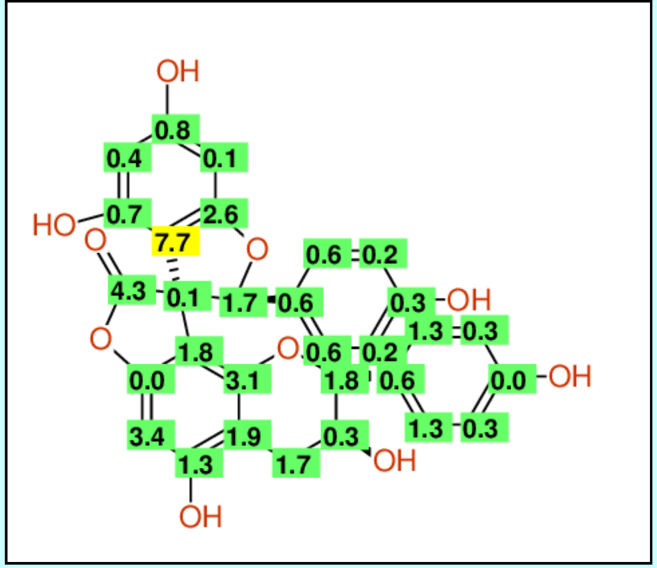

Carbons having strong stereochemical effects

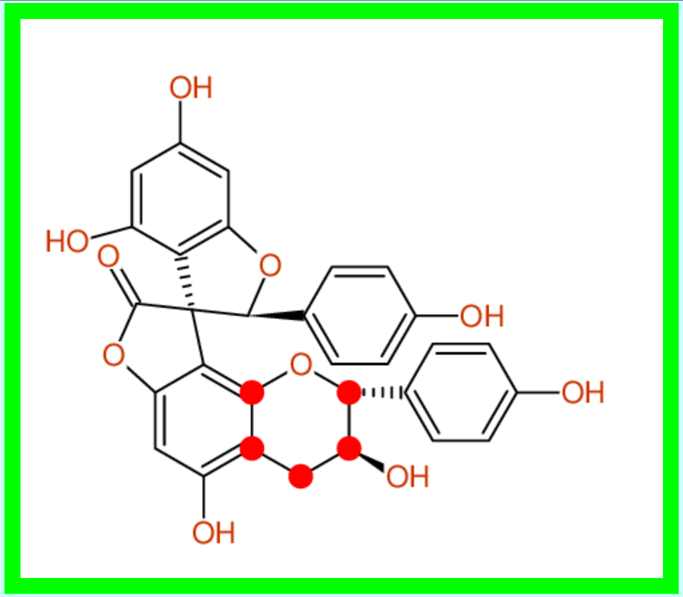

Contribution of methods

HOSE NET NET&HOSE NONE

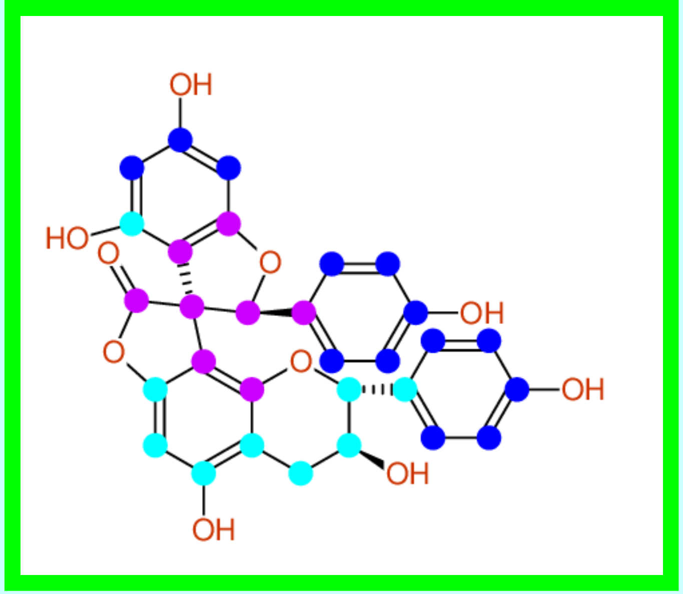

Overall Similarity Index is 1.5
0.0 is a "perfect match", up to approximately 3.0 it is "reasonable",
above 5.0 it is more or less "unbelievable"

	Your assignment	Difference to predicted values
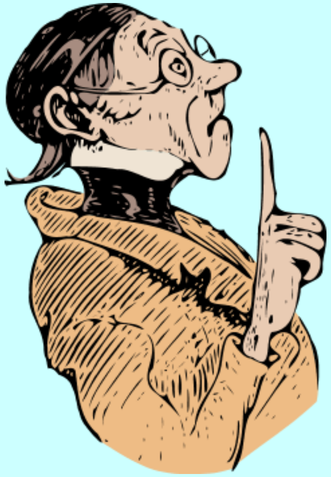	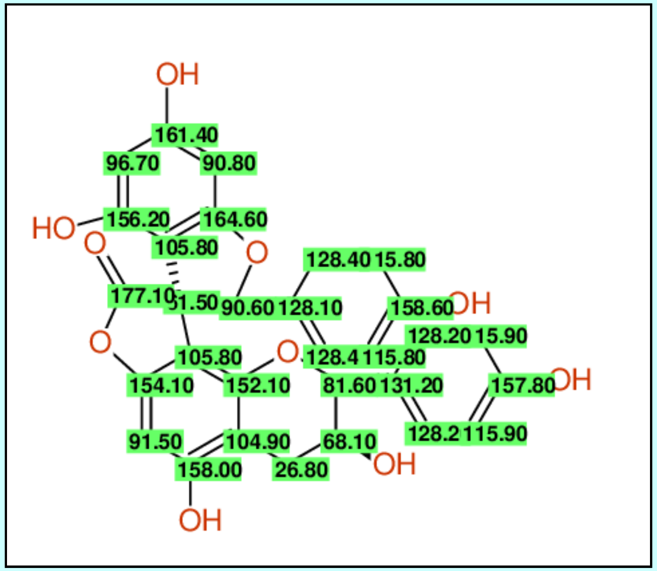	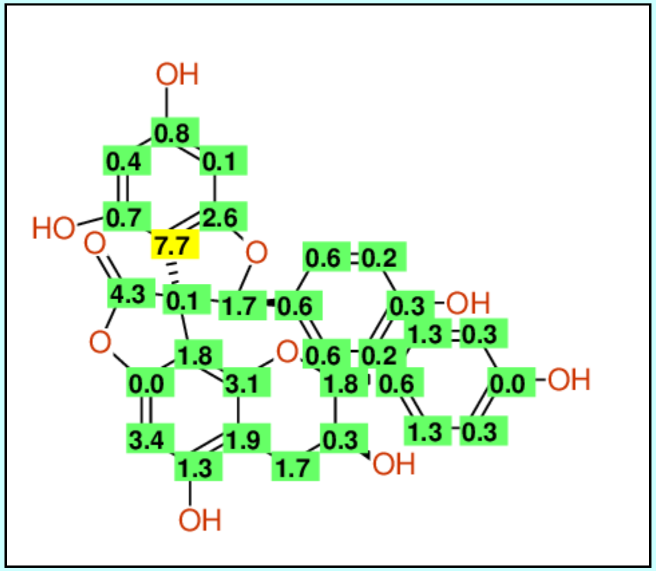

Recall this Compound from PUBCHEM (Stereo-Match from searching 121,500,754 compounds)

4,400,967 Compounds searched in Emolecules - nothing found

Search the Internet for this compound (Skeleton only)
Search the Internet for this compound (Skeleton + Stereochemistry)

Search CHEMSPIDER for this compound (Skeleton only)
Search CHEMSPIDER for this compound (Skeleton + Stereochemistry)

Search the Internet for the molecular formula $C_{30}H_{22}O_{10}$

Search CHEMSPIDER for the molecular formula $C_{30}H_{22}O_{10}$

(Description)

History of your requests for this compound

Date/Time	Result	Method	Assigned Lines	Unassigned Lines	Stereoisomer	Permanent URL	Remark	Comparison of experimental and predicted data (Evaluation only)
2019-10-23 15:34:25	Minor	Evaluation	30	0	YES			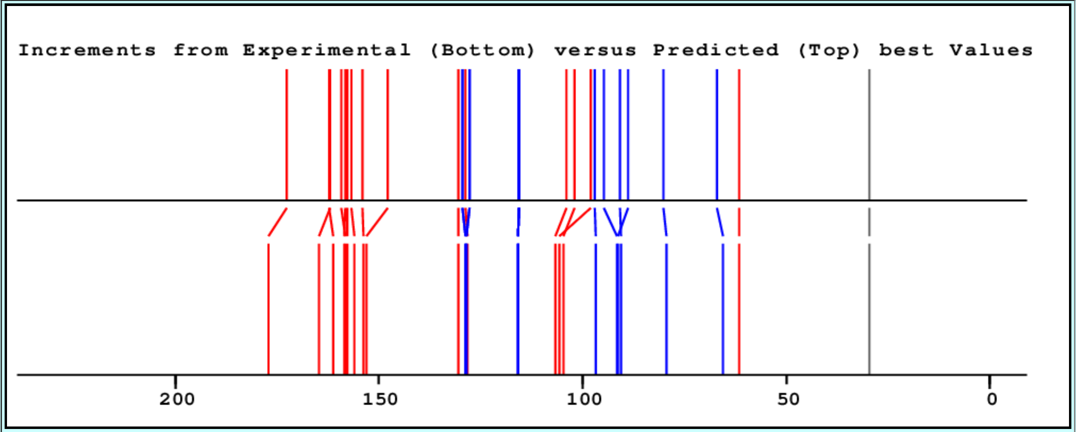
This request	Minor	Evaluation	30	0				

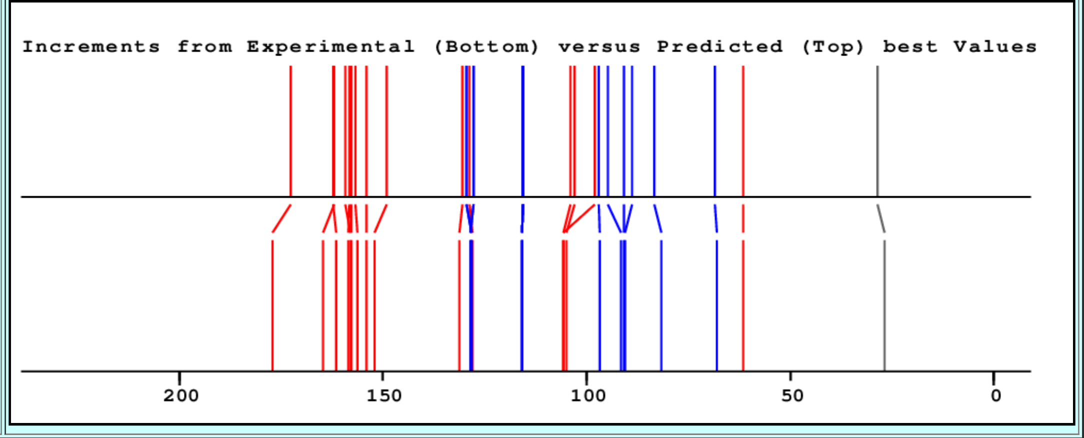

Your Total Usage of the CSEARCH-Robot-Referee

2 Requests have been launched by lpecio@iung.pulawy.pl					
**Year**	**Accept**	**Minor Revision**	**Major Revision**	**Reject**	**Only Prediction**
**2017**		1			
**2019**		1			

Top

Page has been automatically written by CSEARCH
CPU-Usage: Evaluation needed 13.480 seconds
Wolfgang.Robien(at)univie.ac.at
