# Supplementary material for: Yuccalechins A–C from the Yucca schidigera Roezl ex Ortgies Bark: Elucidation of the Relative and Absolute Configurations of Three New Spirobiflavonoids and Their Cholinesterase Inhibitory Activities
Source: Molecules. 2019 Nov 16;24(22):4162. doi: 10.3390/molecules24224162 (PMC6891570; doi:10.3390/molecules24224162)
Supplement: Supplementary file 1 [file molecules-24-04162-s001.zip › CSEARCH Evaluation Report_Yuccalechin_C.pdf]

Automatic Evaluation Report from CSEARCH
created on 2019-10-23 at 15:49:26
based on 340,554 reference spectra

Did you know ?

You can transfer a resulting webpage
to your mobile device using QR-codes.
QR-codes are given in the table below
and point to a permanent URL.

Request from: lpecio@iung.pulawy.pl

Compound: Yuccalechin_C

Project: YS

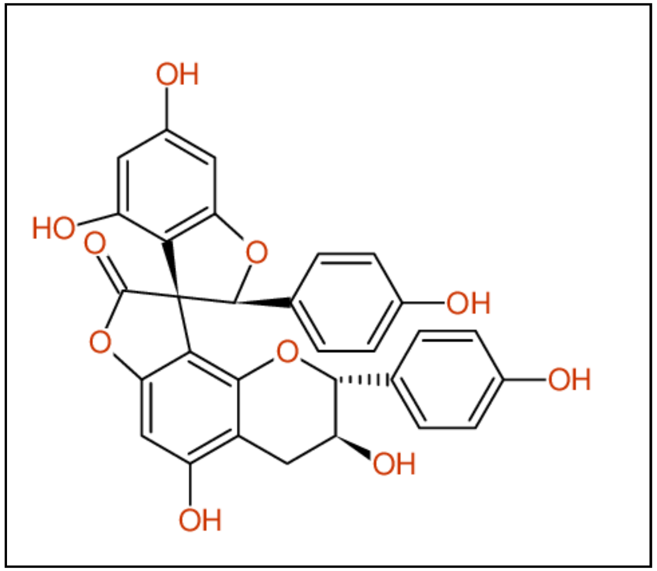

Database	Number of Entries	Owner of Database
Please cite the CSEARCH-Robot-Referee as:<br>N. Haider, W. Robien; http://nmrpredict.orc.univie.ac.at/c13robot/robot.php		

CSEARCH	74,997 (A)	CSEARCH-Data / Wolfgang Robien
CSEARCH	56,549 (B)	CSEARCH-Data / Wolfgang Robien
CSEARCH	28,196 (C)	CSEARCH-Data / Wolfgang Robien
CSEARCH	33,587 (D)	CSEARCH-Data / Wolfgang Robien
CSEARCH	39,132 (E)	CSEARCH-Data / Wolfgang Robien + NMR-Database University of Mainz
CSEARCH	26,196 (F)	CSEARCH-Data / Wolfgang Robien
CSEARCH	50,594 (I)	Upcoming CSEARCH-Data / Wolfgang Robien
CSEARCH	31,307 (L)	NMRShiftDB-Data / Version February 2012

Permanent URL	This page can be verified by a digital signature
	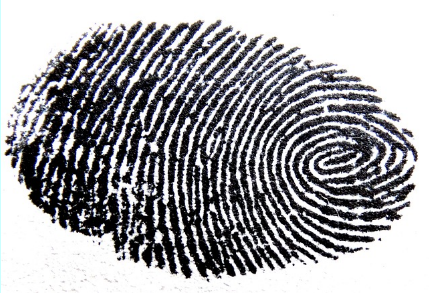

CSEARCH-Version: 9.4.0
Robot-Referee: 2017:06:10

Request from: lpecio@iung.pulawy.pl

Compound: Yuccalechin_C

Project: YS

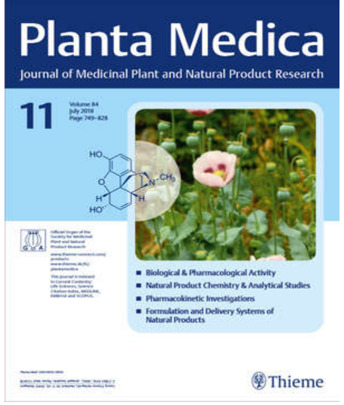

Recommendation given here

Details of Prediction given here

Summary of Supplied Data

Understanding the Color Coding Scheme

Structure Proposal

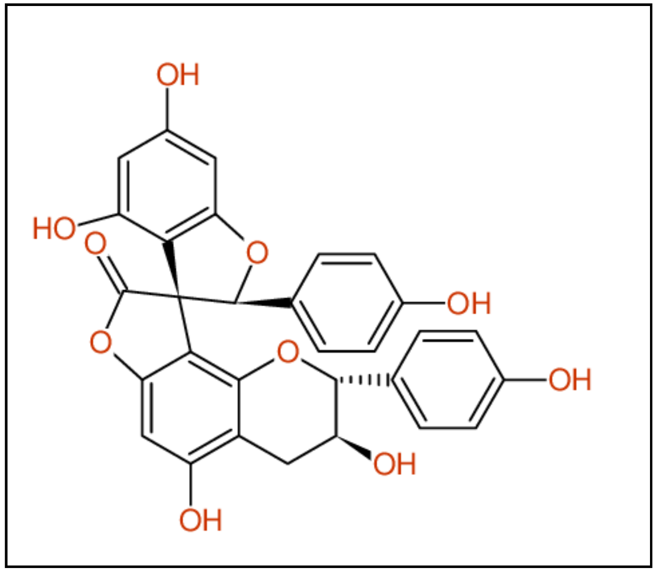

Molecular formula is: $C_{30}H_{22}O_{10}$ Molecular weight is: 542.51 amu

INCHIKEY is: RNDNBGULZNCSNB-RXCSHGEXBY

Numbering Scheme derived from the drawing sequence used during the calculation

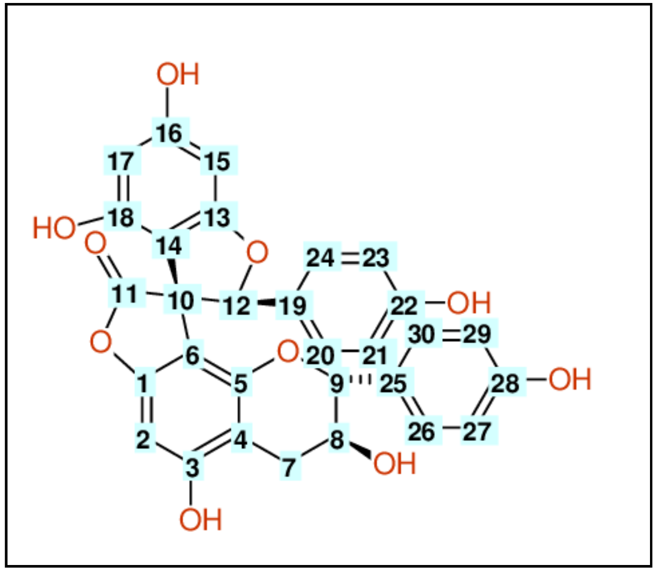

The marked carbons have been fully assigned

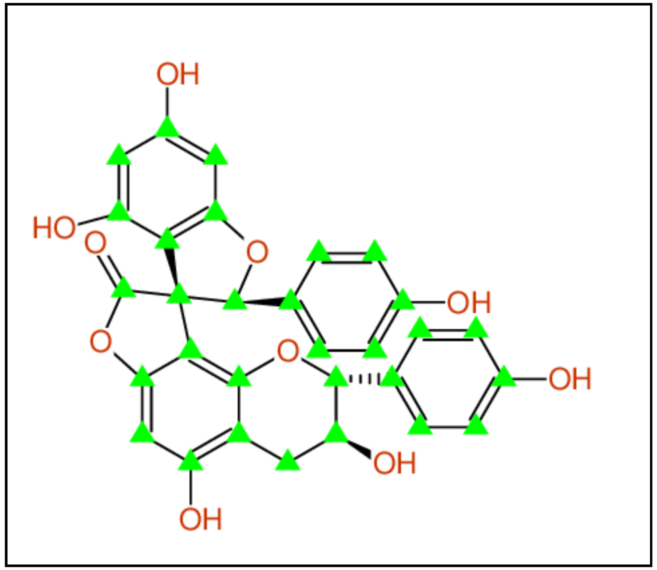

Carbon number	Chemical Shift Value	Multiplicity from Structure	Multiplicity from Experiment

1	152.80	S	S
2	91.10	D	D
3	157.60	S	S
4	105.60	S	S
5	153.20	S	S
6	106.10	S	S
7	30.50	T	T
8	67.70	D	D
9	82.70	D	D
10	61.90	S	S
11	180.60	S	S
12	94.50	D	D
13	163.80	S	S
14	106.10	S	S
15	90.70	D	D
16	161.00	S	S
17	96.50	D	D
18	155.10	S	S
19	128.10	S	S
20	128.40	D	D
21	115.50	D	D
22	158.30	S	S
23	115.50	D	D
24	128.40	D	D
25	130.10	S	S
26	130.40	D	D
27	115.70	D	D
28	158.30	S	S
29	115.70	D	D
30	130.40	D	D

The marked carbons have been fully assigned

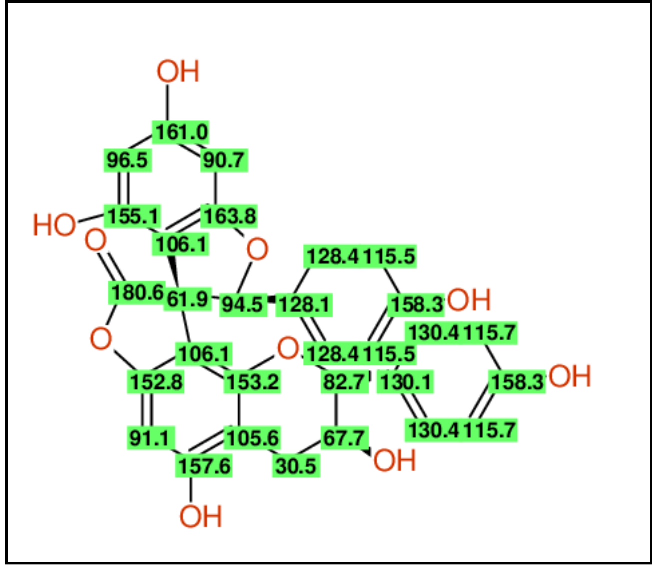

Graphical summary of the Chemical Shift Data

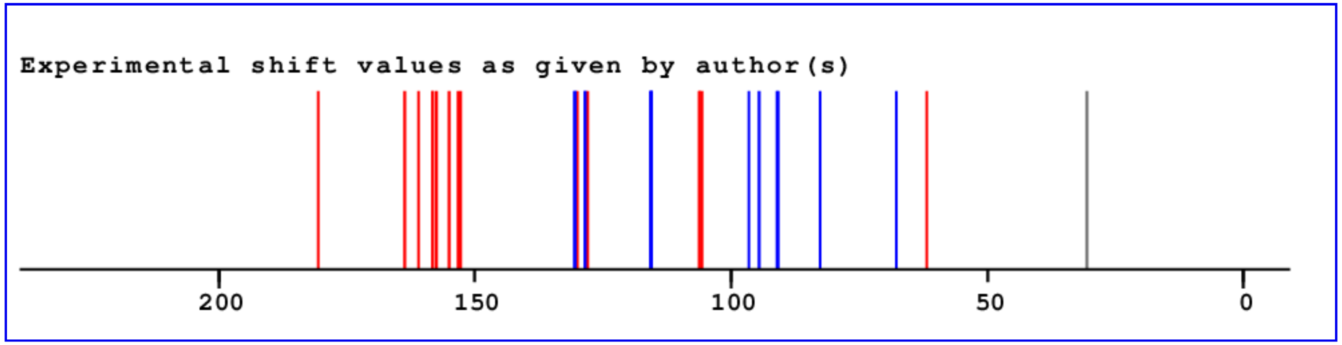

Searching external databases

Recall this Compound from PUBCHEM (Skeleton-Match from searching 121,500,754 compounds)
Recall this Compound from PUBCHEM (Skeleton-Match from searching 121,500,754 compounds)
Recall this Compound from PUBCHEM (Skeleton-Match from searching 121,500,754 compounds)
Recall this Compound from PUBCHEM (Skeleton-Match from searching 121,500,754 compounds)
Recall this Compound from PUBCHEM (Skeleton-Match from searching 121,500,754 compounds)
Recall this Compound from PUBCHEM (Skeleton-Match from searching 121,500,754 compounds)
Recall this Compound from PUBCHEM (Skeleton-Match from searching 121,500,754 compounds)

4,400,967 Compounds searched in Emolecules - nothing found

Search the Internet for this compound (Skeleton only)
Search the Internet for this compound (Skeleton + Stereochemistry)

Search CHEMSPIDER for this compound (Skeleton only)
Search CHEMSPIDER for this compound (Skeleton + Stereochemistry)

Search the Internet for the molecular formula $C_{30}H_{22}O_{10}$

Search CHEMSPIDER for the molecular formula $C_{30}H_{22}O_{10}$

(Description)

Performing Symmetry Analysis

Eventually Symmetry Error: Same shiftvalue - Different environment

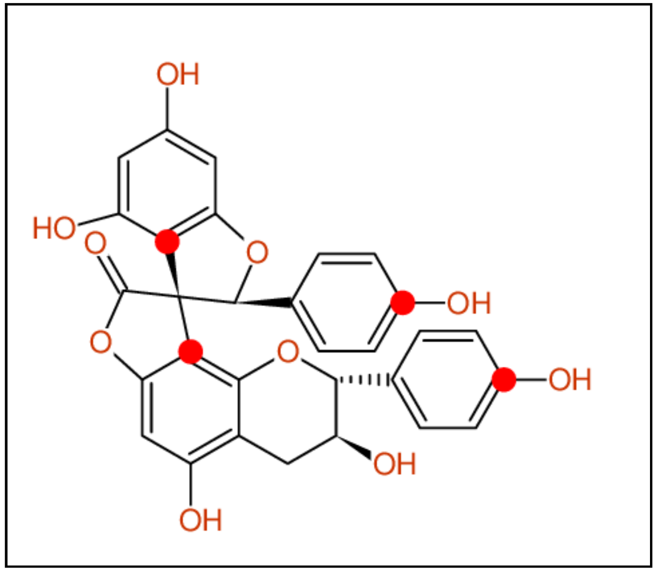

Basic Evaluation: Checking Multiplicities

Checking lines & multiplicity	Carbons/Lines	Singlet	Dublet	Triplet	Quartet	Odd	Even	None
From structure	30	15	14	1	0	16	14	0
From spectrum	30	15	14	1	0	16	14	0

Overall impression on compatibility of multiplicity from structure and experiment

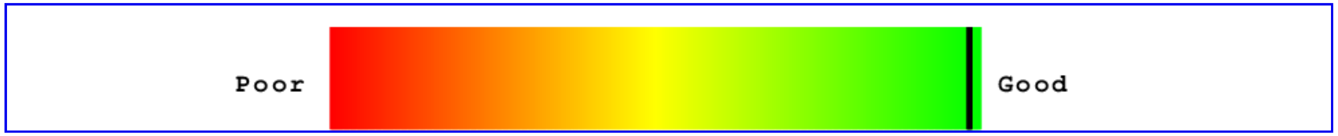

Evaluation based on Spectrum Prediction

Numbering Scheme

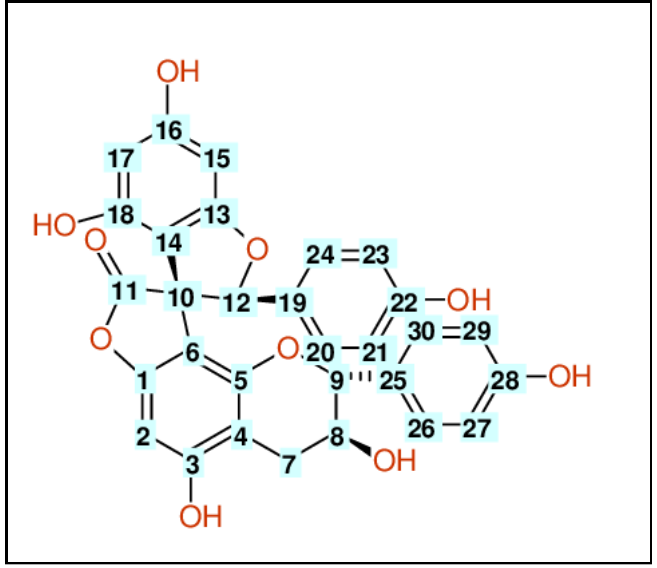

Carbon Number	Neural Network Prediction	HOSE-Code Prediction	Preferred Value from both Predictions	Experimental values	Difference (Exp-Pred/ppm)	Assignment	Prediction Quality
1	154.4	153.9	154.1	152.8	1.3	Assigned by author<br>Check assignment - maybe 153.20 ?	
2	93.8	95.9	94.9	91.1	3.8	Assigned by author<br>Check assignment - maybe 94.50 ?	Only reference material with low similarity
3	162.5	156.1	159.3	157.6	1.7	Assigned by author<br>Check assignment - maybe 158.30 ?	Large Difference between NET & HOSE
4	102.4	103.5	103.0	105.6	2.6	Assigned by author<br>Check assignment - maybe 106.10 ?	Only reference material with low similarity
5	149.0	154.2	149.0	153.2	4.2	Assigned by author<br>Check assignment - maybe 152.80 ?	Large Difference between NET & HOSE<br>Only reference material with low similarity
6	104.0	133.8	104.0	106.1	2.1	Assigned by author<br>Check assignment - maybe 105.60 ?	Large Difference between NET & HOSE<br>Only reference material with low similarity
7	28.5	28.5	28.5	30.5	2.0	Assigned by author	
8	69.5	68.1	68.4	67.7	0.7	Assigned by author	
9	83.1	83.4	83.4	82.7	0.7	Assigned by author	
10	61.6	65.0	61.6	61.9	0.3	Assigned by author	Only reference material with low similarity<br>Only very few similar structures
11	172.8	175.6	172.8	180.6	7.8	Assigned by author	Only reference material with low similarity
12	88.9	79.7	88.9	94.5	5.6	Assigned by author<br>Check assignment - maybe 91.10 ?	Large Difference between NET & HOSE<br>Only reference material with low similarity
13	162.0	149.6	162.0	163.8	1.8	Assigned by author<br>Check assignment - maybe 161.00 ?	Large Difference between NET & HOSE<br>Only reference material with low similarity
14	98.1	128.4	98.1	106.1	8.0	Assigned by author	Large Difference between NET & HOSE<br>Only reference material with low similarity
15	93.4	90.7	90.7	90.7	0.0	Assigned by author	
16	162.8	162.1	162.1	161.0	1.1	Assigned by author<br>Check assignment - maybe 163.80 ?	
17	97.8	97.1	97.1	96.5	0.6	Assigned by author	

Carbon Number ▲ ▼	Neural Network Prediction ▲ ▼	HOSE-Code Prediction ▲ ▼	Preferred Value from both Predictions ▲ ▼	Experimental values ▲ ▼	Difference (Exp-Pred/ppm) ▲ ▼	Assignment	Prediction Quality
18	159.7	156.1	156.9	155.1	1.8	Assigned by author	Only very few similar structures
19	128.7	135.4	128.7	128.1	0.6	Assigned by author<br>Check assignment - maybe 128.40 ?	Large Difference between NET & HOSE<br>Only reference material with low similarity
20	128.5	127.8	127.8	128.4	0.6	Assigned by author<br>Check assignment - maybe 128.10 ?	
21	115.3	115.7	115.7	115.5	0.2	Assigned by author<br>Check assignment - maybe 115.70 ?	
22	160.2	158.3	158.3	158.3	0.0	Assigned by author	
23	115.3	115.7	115.7	115.5	0.2	Assigned by author<br>Check assignment - maybe 115.70 ?	
24	128.5	127.8	127.8	128.4	0.6	Assigned by author<br>Check assignment - maybe 128.10 ?	
25	131.1	130.5	130.6	130.1	0.5	Assigned by author<br>Check assignment - maybe 130.40 ?	
26	129.7	129.5	129.5	130.4	0.9	Assigned by author<br>Check assignment - maybe 130.10 ?	
27	116.0	115.6	115.6	115.7	0.1	Assigned by author	
28	158.1	157.8	157.8	158.3	0.5	Assigned by author<br>Check assignment - maybe 157.60 ?	
29	116.0	115.6	115.6	115.7	0.1	Assigned by author	
30	129.7	129.5	129.5	130.4	0.9	Assigned by author<br>Check assignment - maybe 130.10 ?	
Absolute<br>Signed	2.09ppm (30)<br>0.37ppm (30)	3.84ppm (30)<br>-0.96ppm (30)	1.70ppm (30)<br>0.83ppm (30)			1.34ppm (30)<br>0.83ppm (30)	Average deviation to experimental values<br>(Number of shift pairs used)
Structure representation by reference data over 3.3 shells on average							

Visualization of the differences between predicted and experimental values

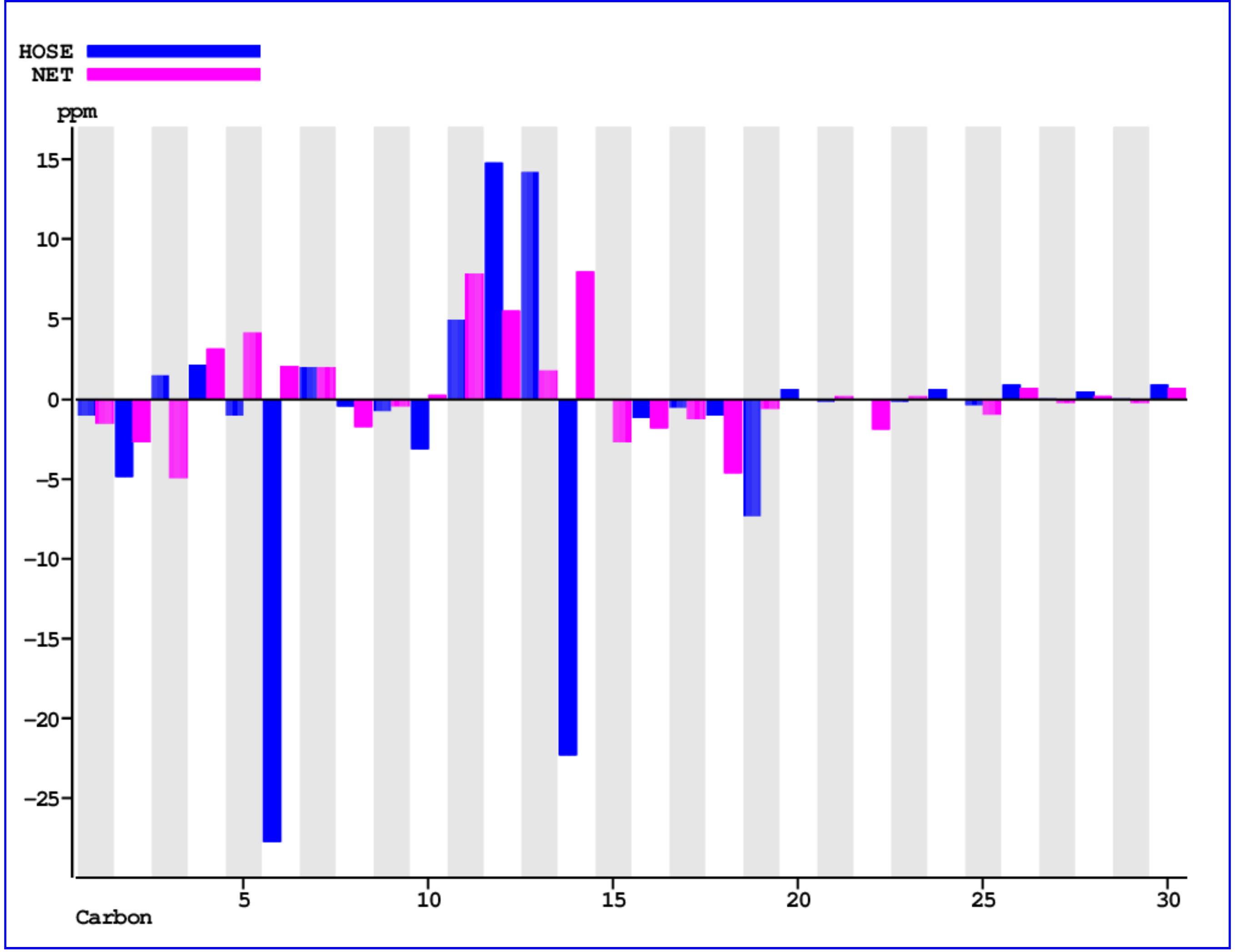

Quality of the Spectrum Prediction

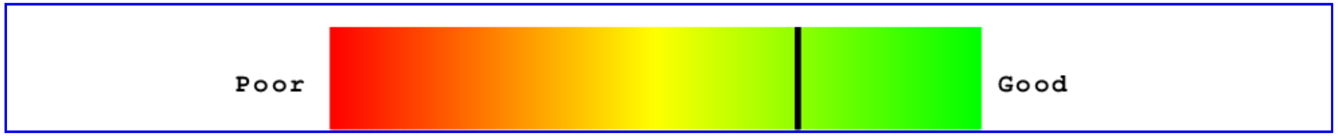

Experimental Chemical Shift Values as given

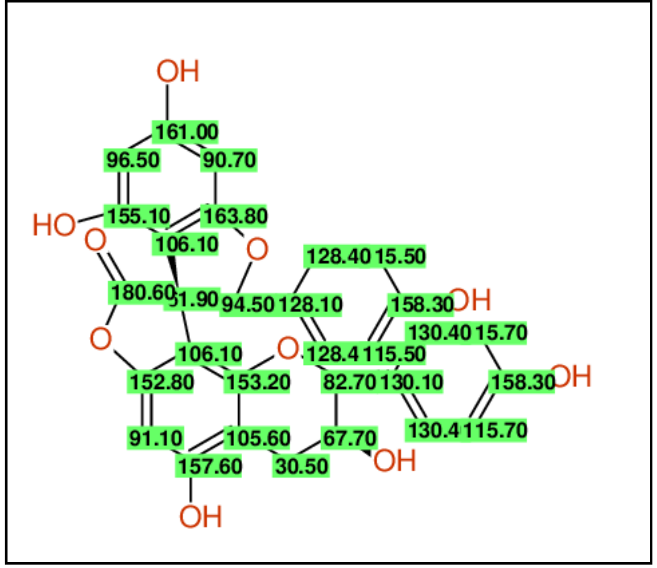

Experimental Chemical Shift Values using Symmetry

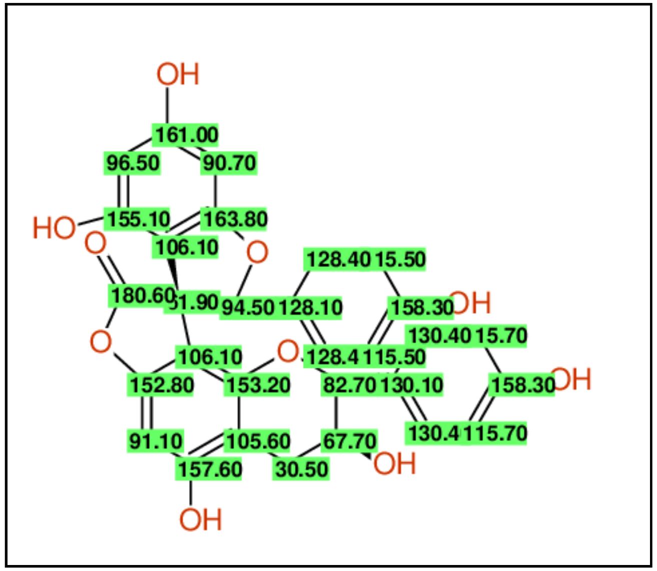

Preferred Chemical Shift Values from both predictions

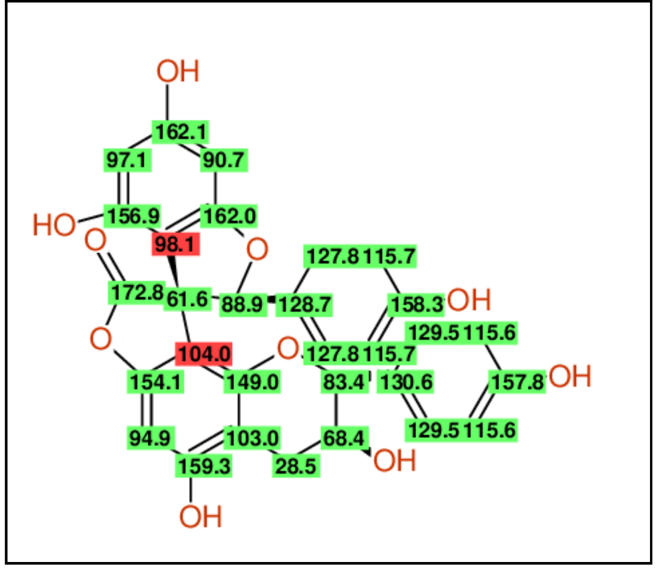

Carbons with massive contribution from stereochemistry

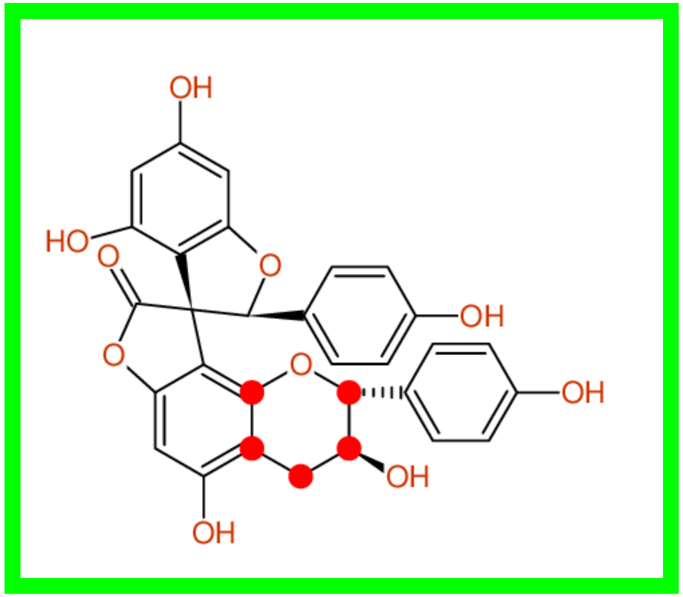

Comparison of Prediction Techniques

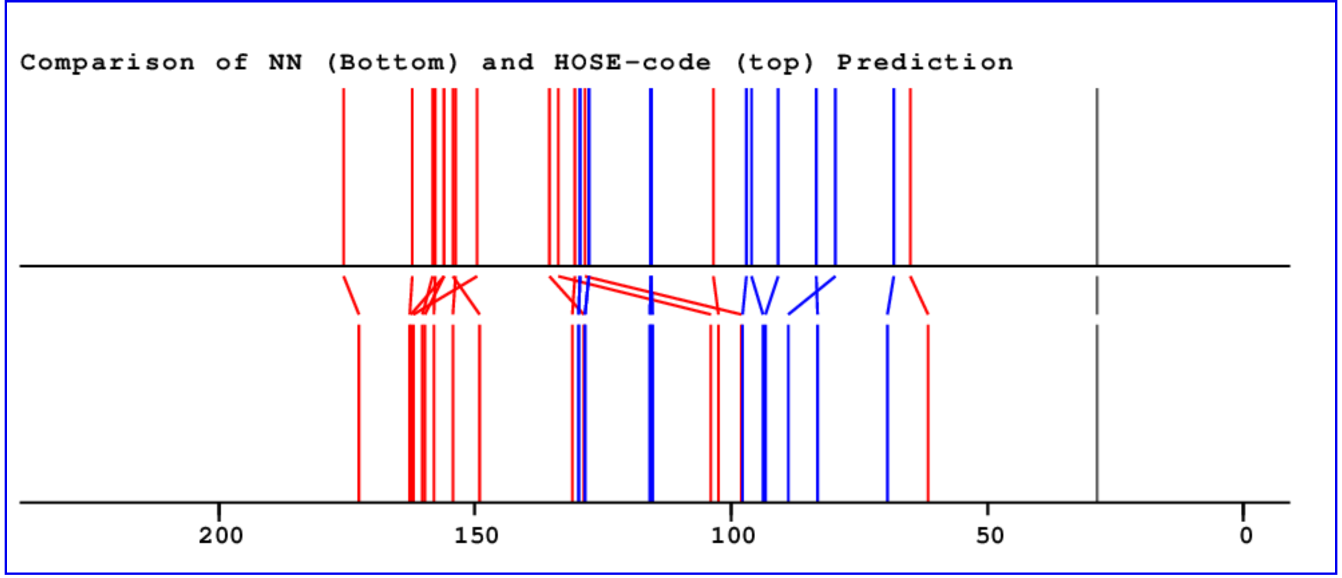

Contribution of the methods

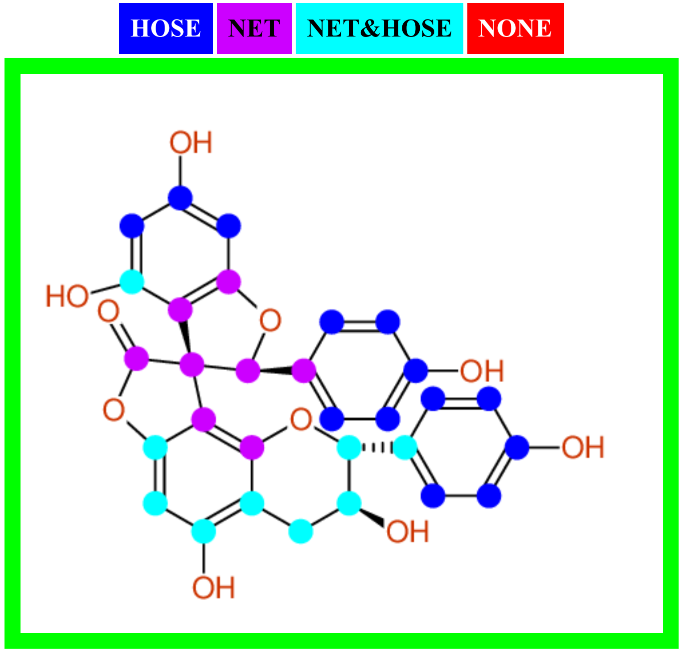

Similarity between predicted and experimental data based on positions

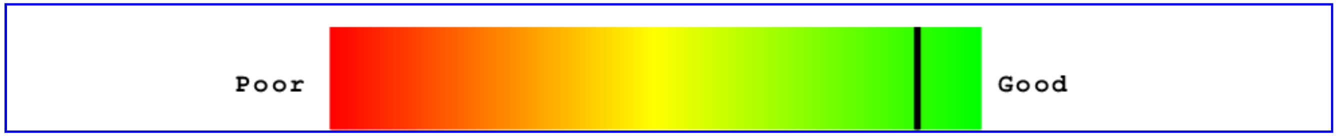

Matching map of predicted versus experimental data

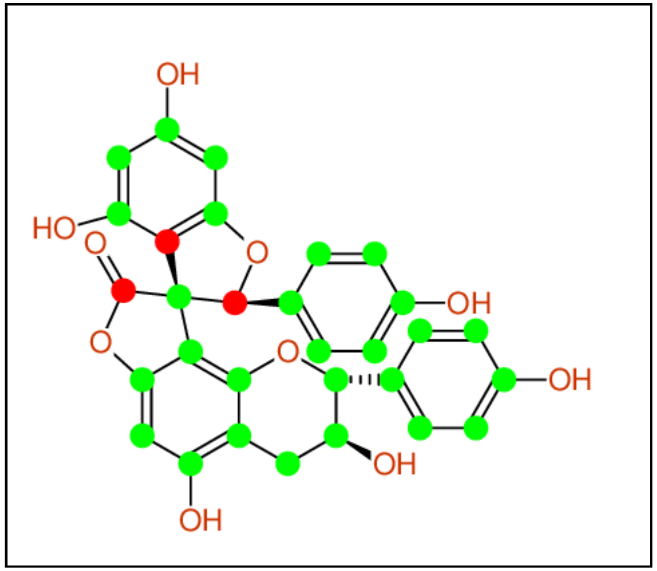

Differences between predicted and experimental data in ppm

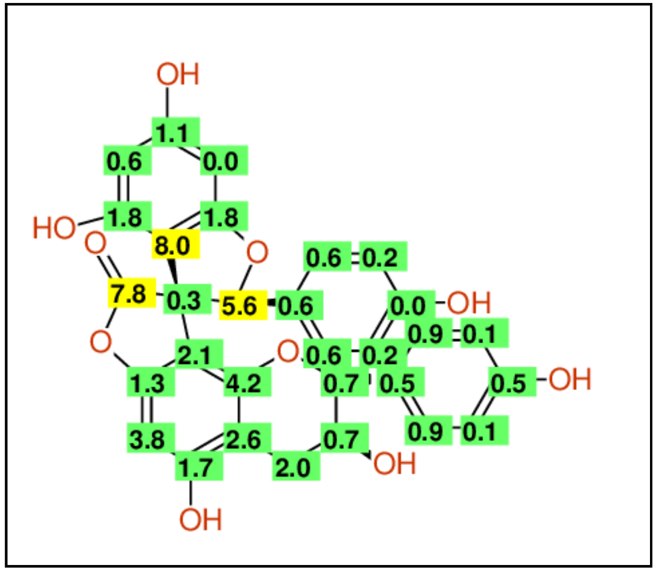

Comparison of Experimental versus Predicted Chemical Shift Values

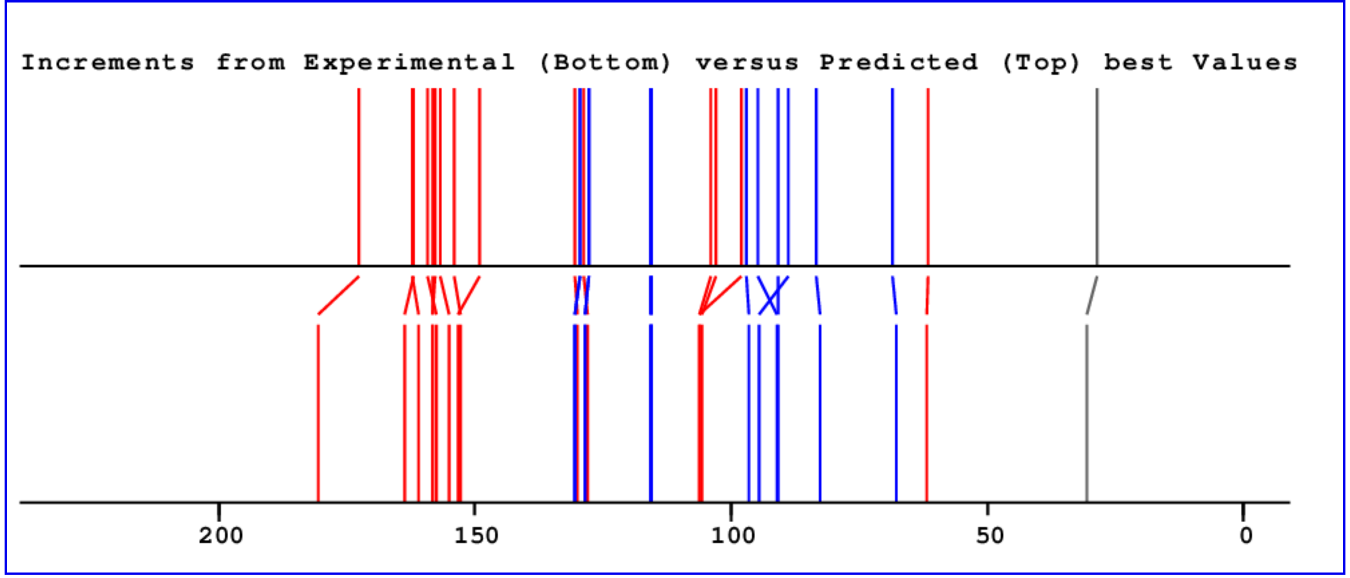

Overall deviation between predicted and experimental data is 1.7ppm

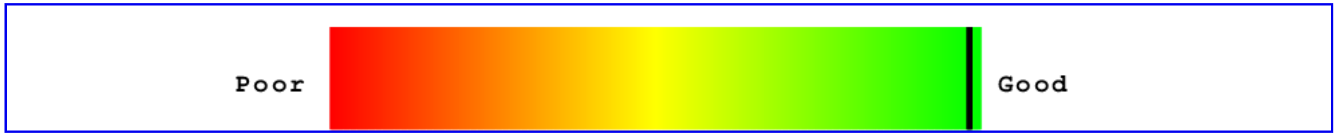

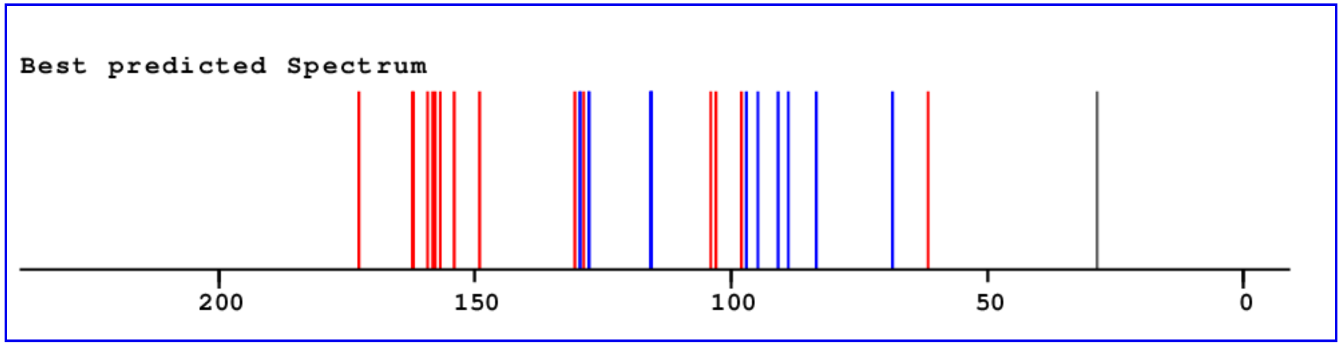

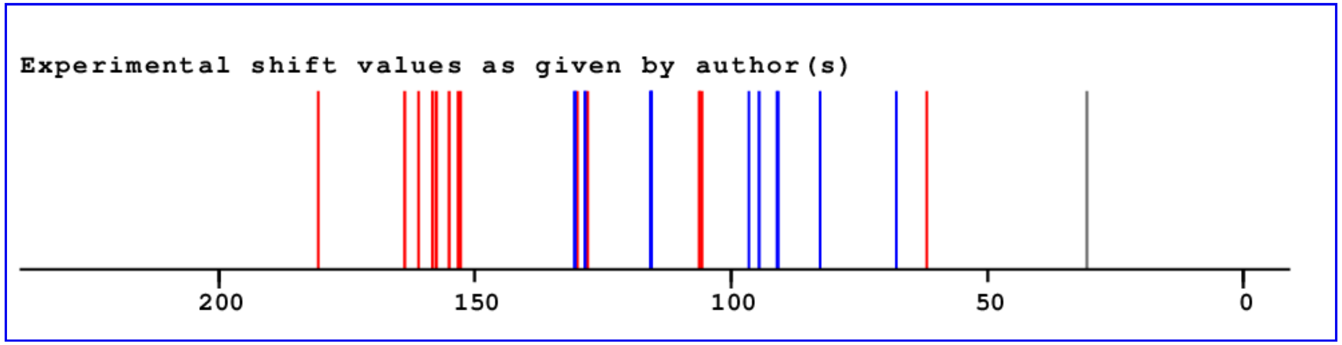

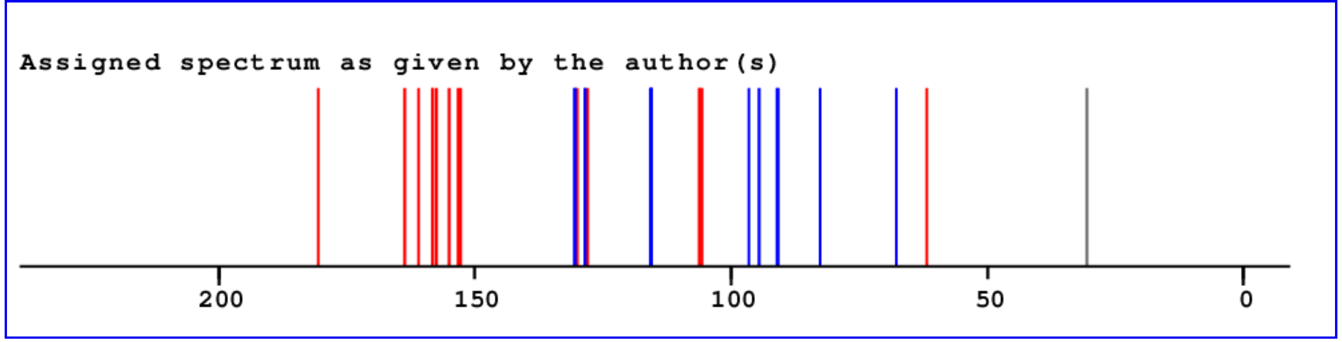

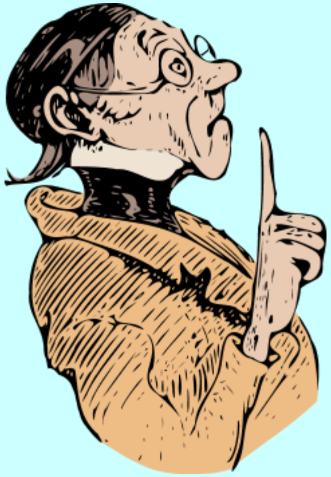

Your assignment	Difference to predicted values
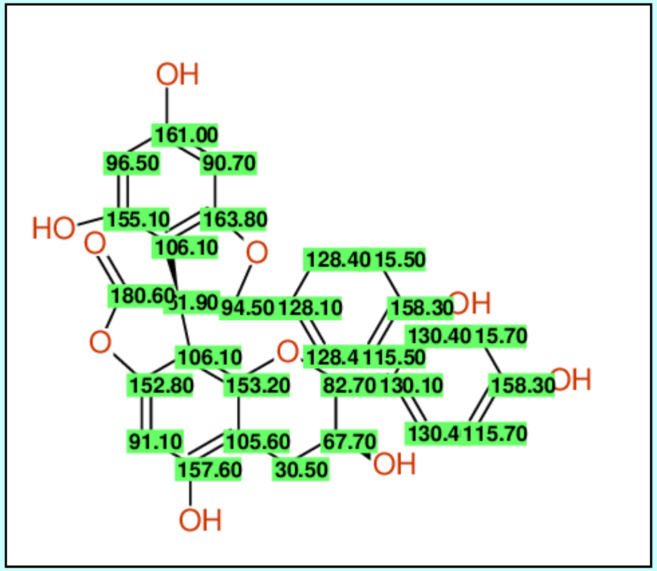	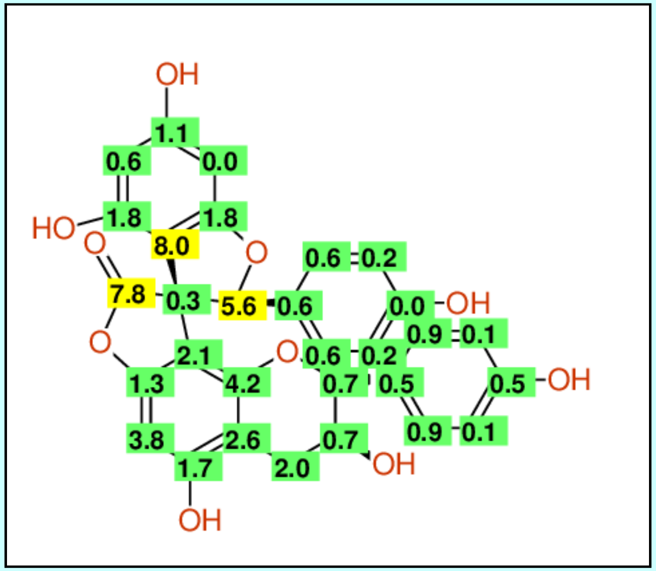

Nothing found when searching CSEARCH for identical structures

(Description)

No alternative structure found when searching CSEARCH for identical spectra

(Description)

Overall Impression

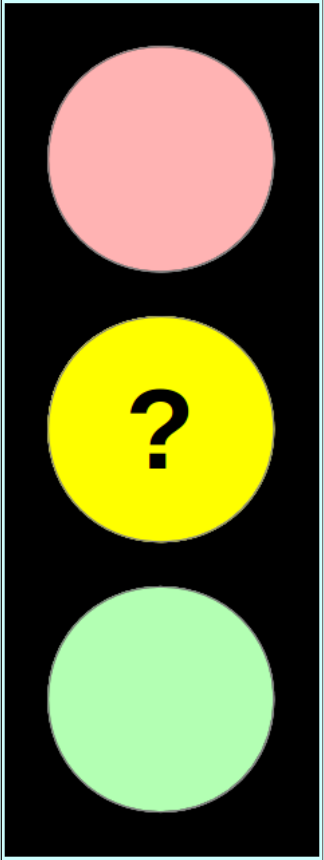

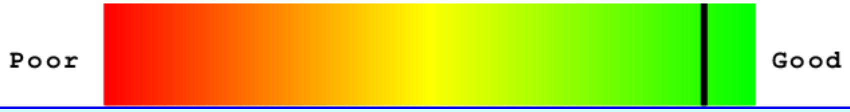

Minor revision might be necessary - please check

Your evaluation was classified either as "Major revision" or "Reject", therefore a similarity search using your peaklist over 74,435,185 predicted CNMR-Spectra for the PUBCHEM-Structures has been automatically launched

Recall Result from Spectral Similarity Search

Compound: Yuccalechin_C

Project: YS

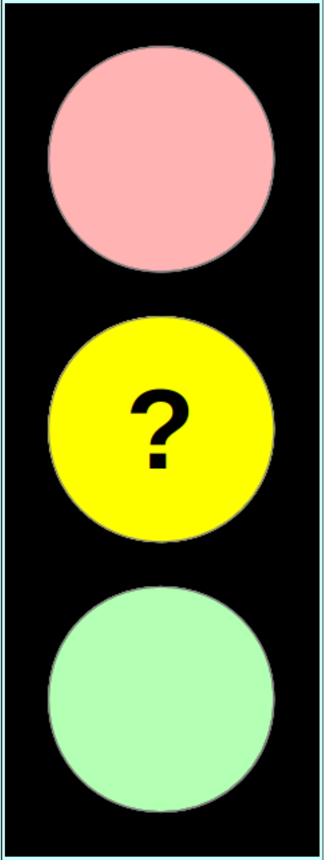

The CSEARCH Robot Referee recommends: Minor revision might be necessary - please check

Check integrity of page via electronic fingerprint

- NN-Prediction and HOSE-Code prediction differs significantly at 7 carbon positions
- Assignment can be probably improved at 18 positions
- 3 Carbon positions (out of 30) have a severe assignment problem
- Spectrum prediction - minor inconsistencies found
- 4 Carbon(s) might have a symmetry-problem

Experimental values
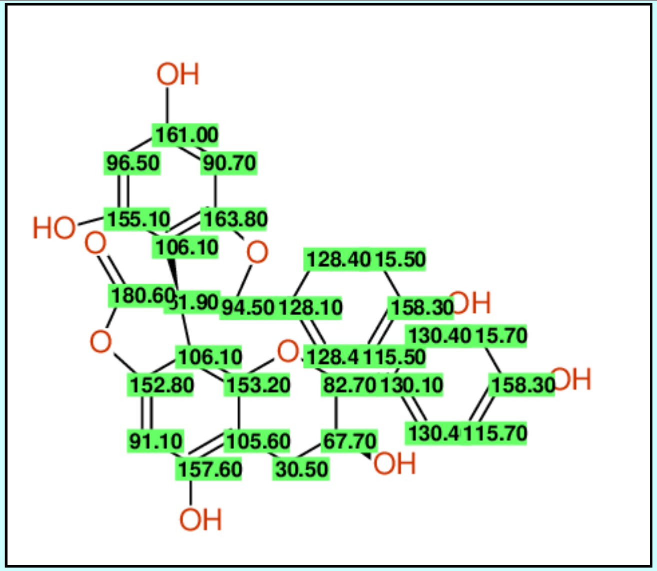
**Predicted values**

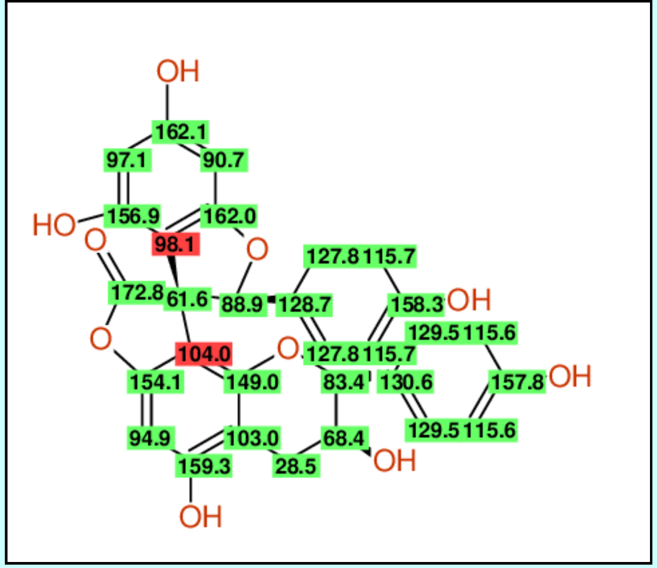

Matching map

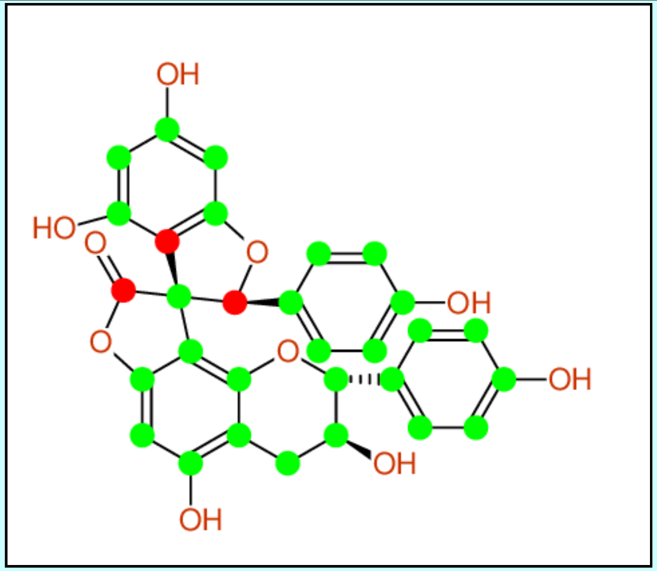

Deviation per position (Average is 1.7ppm)

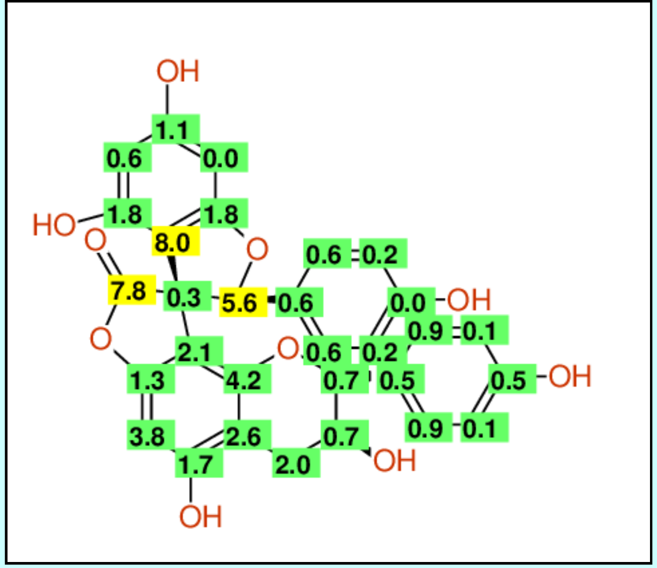

Carbons having strong stereochemical effects

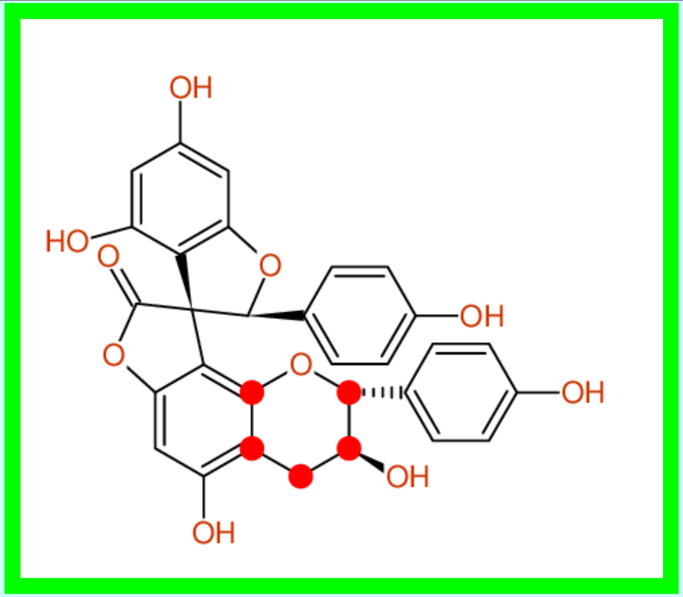

Contribution of methods

HOSE NET NET&HOSE NONE

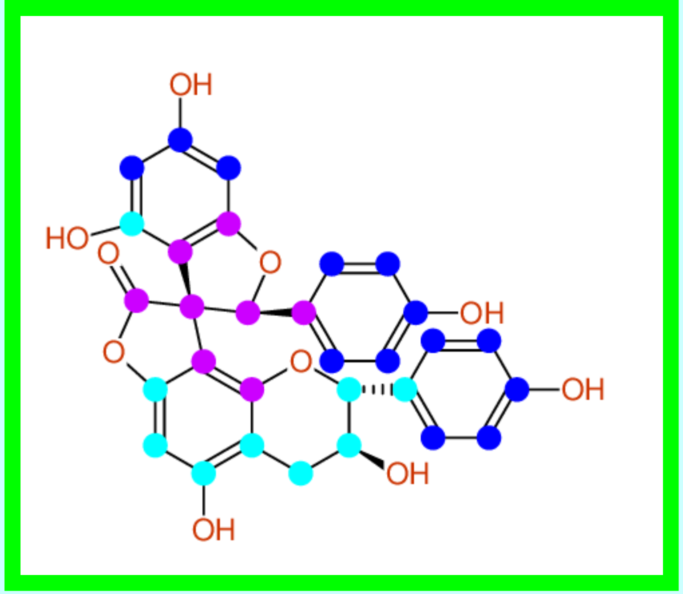

Overall Similarity Index is 1.9
0.0 is a "perfect match", up to approximately 3.0 it is "reasonable",
above 5.0 it is more or less "unbelievable"

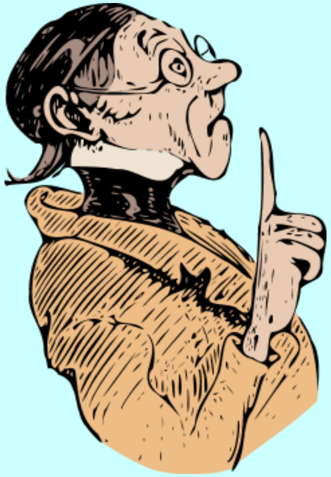

Your assignment	Difference to predicted values
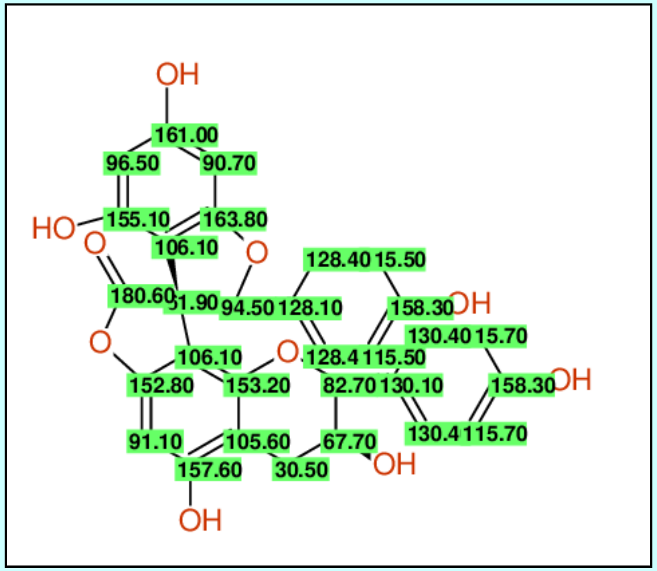	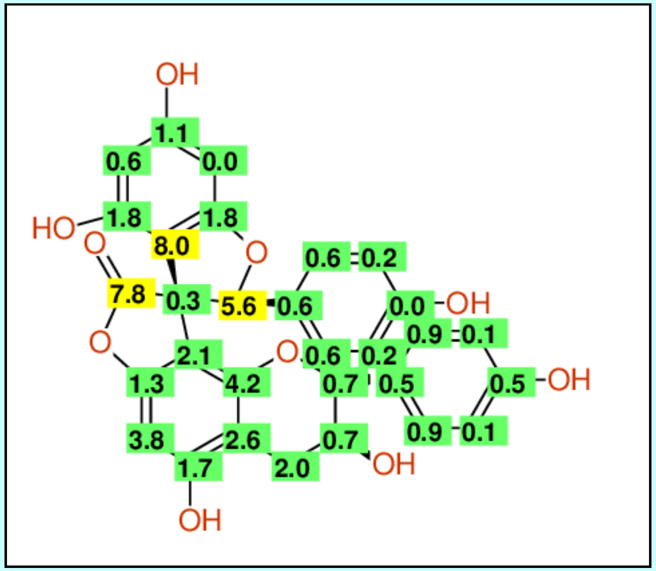

Recall this Compound from PUBCHEM (Skeleton-Match from searching 121,500,754 compounds)
Recall this Compound from PUBCHEM (Skeleton-Match from searching 121,500,754 compounds)
Recall this Compound from PUBCHEM (Skeleton-Match from searching 121,500,754 compounds)
Recall this Compound from PUBCHEM (Skeleton-Match from searching 121,500,754 compounds)

Recall this Compound from PUBCHEM (Skeleton-Match from searching 121,500,754 compounds)
Recall this Compound from PUBCHEM (Skeleton-Match from searching 121,500,754 compounds)
Recall this Compound from PUBCHEM (Skeleton-Match from searching 121,500,754 compounds)

4,400,967 Compounds searched in Emolecules - nothing found

Search the Internet for this compound (Skeleton only)
Search the Internet for this compound (Skeleton + Stereochemistry)

Search CHEMSPIDER for this compound (Skeleton only)
Search CHEMSPIDER for this compound (Skeleton + Stereochemistry)

Search the Internet for the molecular formula $C_{30}H_{22}O_{10}$

Search CHEMSPIDER for the molecular formula $C_{30}H_{22}O_{10}$

(Description)

History of your requests for this compound

Date/Time	Result	Method	Assigned Lines	Unassigned Lines	Stereoisomer	Permanent URL	Remark	Comparison of experimental and predicted data (Evaluation only)
2019-10-23 15:34:25	Minor	Evaluation	30	0	YES			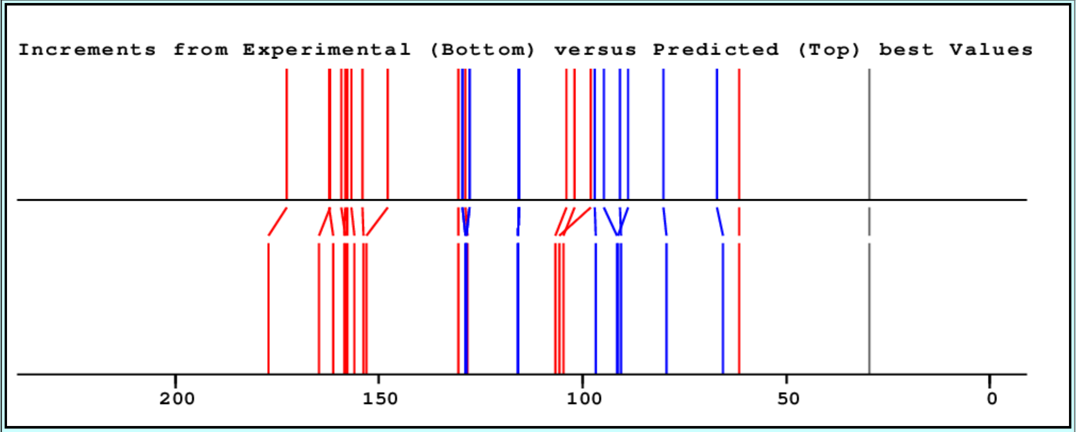
2019-10-23 15:45:27	Minor	Evaluation	30	0	YES			

								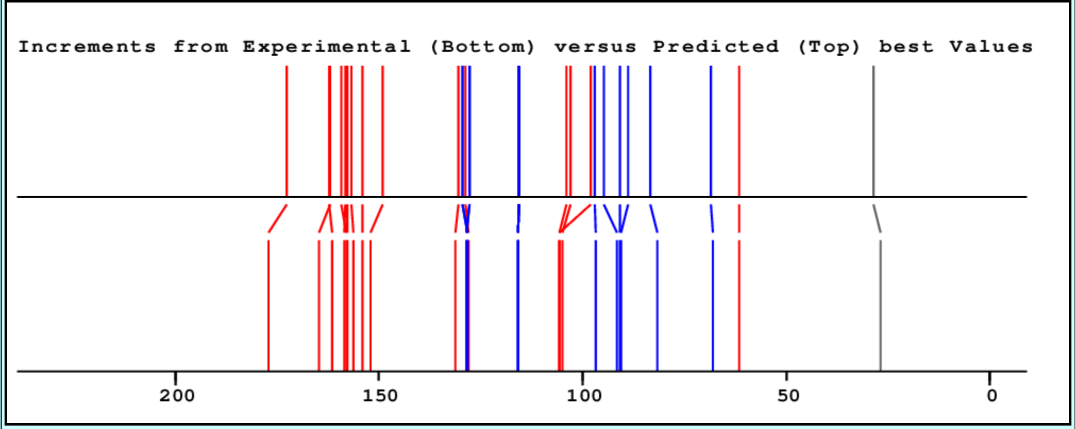
This request	Minor	Evaluation	30	0				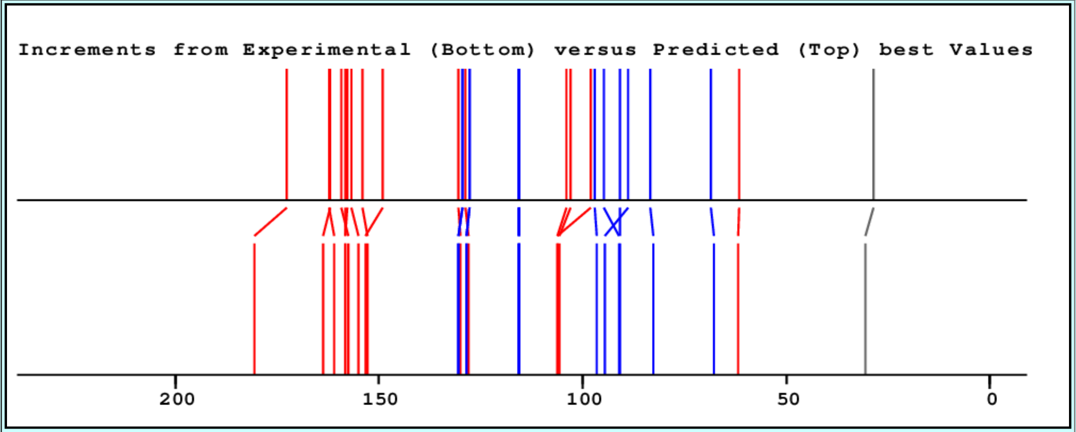

Your Total Usage of the CSEARCH-Robot-Referee

3 Requests have been launched by lpecio@iung.pulawy.pl					
Year	Accept	Minor Revision	Major Revision	Reject	Only Prediction
2017		1			
2019		2			

Top

Page has been automatically written by CSEARCH
CPU-Usage: Evaluation needed 12.458 seconds
Wolfgang.Robien(at)univie.ac.at
