# Supplementary material for: Yuccalechins A–C from the Yucca schidigera Roezl ex Ortgies Bark: Elucidation of the Relative and Absolute Configurations of Three New Spirobiflavonoids and Their Cholinesterase Inhibitory Activities
Source: Molecules. 2019 Nov 16;24(22):4162. doi: 10.3390/molecules24224162 (PMC6891570; doi:10.3390/molecules24224162)

Supplementary Materials

Yuccalechins A-C from the *Yucca schidigera* Roezl ex Ortgies bark: elucidation of relative and absolute configurations of three new spirobiflavonoids and their cholinesterase inhibitory activity.

Łukasz Pecio [1†,*], Mostafa Alilou [2†,*], Solomiia Kozachok [1], Ilkay Erdogan Orhan [3], Gokcen Eren [4], Fatma Sezer Senol Deniz [3], Hermann Stuppner [2], and Wiesław Oleszek [1]

[1] Department of Biochemistry and Crop Quality, Institute of Soil Science and Plant Cultivation—State Research Institute, Czartoryskich 8, 24-100 Puławy, Poland; lpecio@iung.pulawy.pl (Ł.P.); skozachok@iung.pulawy.pl (S.K.); wieslaw.oleszek@iung.pulawy.pl (W.O.)

[2] Institute of Pharmacy/Pharmacognosy, Center for Molecular Biosciences Innsbruck, University of Innsbruck, Innrain 80/82, Innsbruck 6020, Austria; mostafa.alilou@student.uibk.ac.at (M.A.); hermann.stuppner@uibk.ac.at (H.S.)

[3] Department of Pharmacognosy, Faculty of Pharmacy, Gazi University, 06330 Ankara, Turkey; iorhan@gazi.edu.tr (I.E.O.); fssenol@gazi.edu.tr (F.S.S.D.)

[4] Department of Pharmaceutical Chemistry, Faculty of Pharmacy, Gazi University, 06330 Ankara, Turkey; gokcene@gazi.edu.tr (G.E.)

* Correspondence: lpecio@iung.pulawy.pl (Ł.P.); mostafa.alilou@student.uibk.ac.at (M.A.)

† These authors contributed equally to this work.

Received: date; Accepted: date; Published: date

Abstract: The ethyl acetate fraction of the methanolic extract of *Yucca schidigera* Roezl ex Ortgies bark exhibited moderate acetylcholinesterase (AChE) and butyrylcholinesterase (BChE) inhibitory activity (IC_{50} 47.44 μg mL^{-1} and 47.40 μg mL^{-1} respectively). Gel filtration on Sephadex LH-20 and further RP-C_{18} preparative HPLC of EtOAc fraction afforded 15 known and three new compounds, stereoisomers of larixinol. The structures of the isolated spirobiflavonoids **15**, **26** and **29** were elucidated using 1D and 2D NMR and MS spectroscopic techniques. The relative configuration of isolated compounds was assigned based on coupling constants and ROESY correlations along with applying DP4+ probability method in case of ambiguous chiral centers. Determination of absolute configuration was performed by comparing calculated ECD spectra with experimental ones. Compounds **26** and **29**, obtained in sufficient amounts, were evaluated for activities against AChE and BChE, and showed a weak inhibition only towards AChE (IC_{50} 294.18 μM for **26**, and 655.18 μM for **29**). Furthermore, molecular docking simulations were performed to investigate the possible binding modes of **26** and **29** with AChE.

Keywords: *Yucca schidigera*; Asparagaceae; spirobiflavonoid; absolute configuration; DP4+; ECD; Alzheimer's disease

List of Figures

Figure S1. ^{1}H NMR spectrum of *trans*-3,3',5,5'-tetrahydroxy-4'-methoxystilbene (**13**) (500 MHz, MeOH-d_4, 30 °C). 4

Figure S2. ^{13}C NMR spectrum of *trans*-3,3',5,5'-tetrahydroxy-4'-methoxystilbene (**13**) (125 MHz, MeOH-d_4, 30 °C). 4

Figure S3. ^{1}H NMR spectrum of yuccalechin A (**15**) (500 MHz, MeOH-d_4, 30 °C). 5

Figure S4. ^{13}C NMR spectrum of yuccalechin A (**15**) (125 MHz, MeOH-d_4, 30 °C). 5

Figure S5. ^{1}H-^{1}H COSY NMR spectrum of yuccalechin A (**15**) (500 MHz, MeOH-d_4, 30 °C). 6

Figure S6. ^{1}H-^{1}H ROESY (250 ms) NMR spectrum of yuccalechin A (**15**) (500 MHz, MeOH-d_4, 30 °C). 6

Figure S7. ^{1}H-^{13}C HSQC NMR spectrum of yuccalechin A (**15**) (500/125 MHz, MeOH-d_4, 30 °C). 7

Figure S8. ^{1}H-^{13}C H2BC NMR spectrum of yuccalechin A (**15**) (500/125 MHz, MeOH-d_4, 30 °C). 7

Figure S9. ^{1}H-^{13}C HMBC (8 Hz) NMR spectrum of yuccalechin A (**15**) (500/125 MHz, MeOH-d_4, 30 °C). 8

Figure S10. Optimized conformers of yuccalechin A (**15**) in DFT/B3LYP/6-31G(d,p)/IEFPCM/methanol level of theory. . 8

Figure S11. Calculated DP4+ probabilities of yuccalechin A (**15**) using mpw1pw91/6-111G+(d,p)/ IEFPCM/methanol level of theory. Isomer 1 is 2”*R*,3”*S*,2*R*,3*R* and isomer 2 is 2”*R*,3”S,2*S*,3*R*. 9

Table S1. Calculated and experimental chemical shift values used for DP4+ calculation for (**15**). 9

Figure S12. HRESIMS (Q-TOF) analysis of yuccalechin A (**15**) in negative ion mode. 10

Figure S13. ^{1}H NMR spectrum of aromadendrin (**16**) (500 MHz, MeOH-d_4, 30 °C). 11

Figure S14. ^{13}C NMR spectrum of aromadendrin (**16**) (125 MHz, MeOH-d_4, 30 °C). 11

Figure S15. ^{1}H NMR spectrum of *trans*-resveratrol (**21**) (500 MHz, MeOH-d_4, 30 °C). 12

Figure S16. ^{13}C NMR spectrum of *trans*-resveratrol (**21**) (125 MHz, MeOH-d_4, 30 °C). 12

Figure S17. ^{1}H NMR spectrum of yuccalechin B (**26**) (500 MHz, MeOH-d_4, 30 °C). 13

Figure S18. ^{13}C NMR spectrum of yuccalechin B (**26**) (125 MHz, MeOH-d_4, 30 °C). 13

Figure S19. ^{1}H-^{1}H COSY NMR spectrum of yuccalechin B (**26**) (500 MHz, MeOH-d_4, 30 °C). 14

Figure S20. ^{1}H-^{1}H ROESY (250 ms) NMR spectrum of yuccalechin B (**26**) (500 MHz, MeOH-d_4, 30 °C). 14

Figure S21. ^{1}H-^{13}C HSQC NMR spectrum of yuccalechin B (**26**) (500/125 MHz, MeOH-d_4, 30 °C). 15

Figure S22. ^{1}H-^{13}C H2BC NMR spectrum of yuccalechin B (**26**) (500/125 MHz, MeOH-d_4, 30 °C). 15

Figure S23. ^{1}H-^{13}C HMBC (8Hz) NMR spectrum of yuccalechin B (**26**) (500/125 MHz, MeOH-d_4, 30 °C). 16

Figure S24. Optimized conformers of yuccalechin B (**26**) in DFT/B3LYP/6-31G(d,p)/IEFPCM/MeOH level of theory. ... 17

Figure S25. Calculated DP4+ probabilities of yuccalechin B (**26**) using mpw1pw91/6-111G+(d,p)/IEFPCM/ methanol level of theory. Isomer 1 is 2”*R*,3”*S*,2*S*,3*S* and isomer 2 is 2”*R*,3”*S*,2*R*,3*S*. 17

Table S2. Calculated and experimental chemical shift values used for DP4+ calculation for (**26**). 18

Figure S26. HRESIMS (Q-TOF) analysis of yuccalechin B (**26**) in negative ion mode. 19

Figure S27. ^{1}H NMR spectrum of yuccalechin C (**29**) (500 MHz, MeOH-d_4, 30 °C). 20

Figure S28. ^{13}C NMR spectrum of yuccalechin C (**29**) (125 MHz, MeOH-d_4, 30 °C). 20

Figure S29. ^{1}H-^{1}H COSY NMR spectrum of yuccalechin C (**29**) (500 MHz, MeOH-d_4, 30 °C). 21

Figure S30. ^{1}H-^{1}H ROESY (250 ms) NMR spectrum of yuccalechin C (**29**) (500 MHz, MeOH-d_4, 30 °C). 21

Figure S31. ^{1}H-^{13}C HSQC NMR spectrum of yuccalechin C (**29**) (500/125 MHz, MeOH-d_4, 30 °C). 22

Figure S32. ^{1}H-^{13}C H2BC NMR spectrum of yuccalechin C (**29**) (500/125 MHz, MeOH-d_4, 30 °C). 22

Figure S33. ^{1}H-^{13}C HMBC (8Hz) NMR spectrum of yuccalechin C (**29**) (500/125 MHz, MeOH-d_4, 30 °C). 23

Figure S34. Optimized conformers of yuccalechin C (**29**) and their contribution to Boltzmann averaging at DFT/B3LYP/6-31G(d) level of theory in gas phase. 23

Figure S35. Calculated DP4+ probabilities of yuccalechin C (**29**) using mpw1pw91/6-111G+(d,p)/ CPCM/methanol level of theory. Isomer 1 is 2”*R*,3”*R*,2*R*,3*R* and isomer 2 is 2”*R*,3”*R*,2*R*,3*S* and isomer 3 is 2”*R*,3”*R*,2*S*,3*R*. 24

Table S3. Calculated and experimental chemical shift values used for DP4+ calculation for (**29**). 24

Figure S36. HRESIMS (Q-TOF) analysis of yuccalechin C (**29**) in negative ion mode. 25

Figure S37. ^{1}H NMR spectrum of yuccaol E (**37**) (500 MHz, MeOH-d_4, 30 °C). 26

Figure S38. ^{13}C NMR spectrum of yuccaol E (**37**) (125 MHz, MeOH-d_4, 30 °C). 26

Figure S39. ^{1}H NMR spectrum of naringenin (**38**) (500 MHz, MeOH-d_4, 30 °C). 27

Figure S40. ^{13}C NMR spectrum of naringenin (**38**) (125 MHz, MeOH-d_4, 30 °C). 27

Figure S41. ^{1}H NMR spectrum of yuccaol C (**39**) (500 MHz, MeOH-d_4, 30 °C). 28

Figure S42. ^{13}C NMR spectrum of yuccaol C (**39**) (125 MHz, MeOH-d_4, 30 °C). 28

Figure S43. ^{1}H NMR spectrum of yuccalide A (**40**) (500 MHz, MeOH-d_4, 30 °C). 29

Figure S44. ^{13}C NMR spectrum of yuccalide A (**40**) (125 MHz, MeOH-d_4, 30 °C). 29

Figure S45. ^{1}H NMR spectrum of yuccaol D (**42**) (500 MHz, MeOH-d_4, 30 °C). 30

Figure S46. ^{13}C NMR spectrum of yuccaol D (**42**) (125 MHz, MeOH-d_4, 30 °C). 30

Figure S47. ^{1}H NMR spectrum of kaempferol (**44**) (500 MHz, MeOH-d_4, 30 °C). 31

Figure S48. ^{13}C NMR spectrum of kaempferol (**44**) (125 MHz, MeOH-d_4, 30 °C). 31

Figure S49. ^{1}H NMR spectrum of yuccaol A (**47**) (500 MHz, MeOH-d_4, 30 °C). 32

Figure S50. ^{13}C NMR spectrum of yuccaol A (**47**) (125 MHz, MeOH-d_4, 30 °C). 32

Figure S51. ^{1}H NMR spectrum of yuccaol B (**48**) (500 MHz, MeOH-d_4, 30 °C). 33

Figure S52. ^{13}C NMR spectrum of yuccaol B (**48**) (125 MHz, MeOH-d_4, 30 °C). 33

Figure S53. ^{1}H NMR spectrum of gloriosaol E (**49**) (500 MHz, MeOH-d_4, 30 °C). 34

Figure S54. ^{13}C NMR spectrum of gloriosaol E (**49**) (125 MHz, MeOH-d_4, 30 °C). 34

Figure S55. ^{1}H NMR spectrum of gloriosaol D (**50**) (500 MHz, MeOH-d_4, 30 °C). 35

Figure S56. ^{13}C NMR spectrum of gloriosaol D (**50**) (125 MHz, MeOH-d_4, 30 °C). 35

Figure S57. ^{1}H NMR spectrum of gloriosaol A (**54**) (500 MHz, MeOH-d_4, 30 °C). 36

Figure S58. ^{13}C NMR spectrum of gloriosaol A (**54**) (125 MHz, MeOH-d_4, 30 °C). 36

Figure S59. ^{1}H NMR spectrum of gloriosaol C (**58**) (500 MHz, MeOH-d_4, 30 °C). 37

Figure S60. ^{13}C NMR spectrum of gloriosaol C (**58**) (125 MHz, MeOH-d_4, 30 °C). 37

Figure S1. ^{1}H NMR spectrum of *trans*-3,3′,5,5′-tetrahydroxy-4′-methoxystilbene (**13**) (500 MHz, MeOH-d_4, 30 °C).

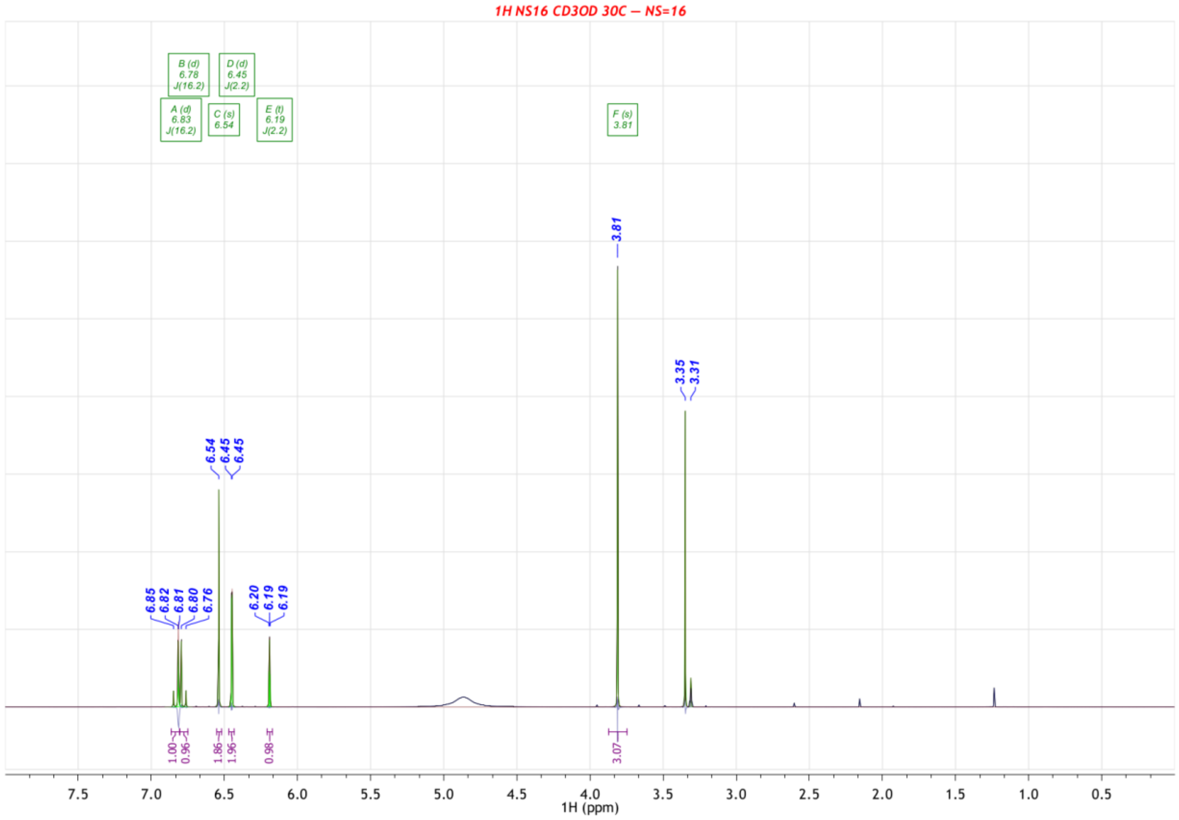

Figure S2. ^{13}C NMR spectrum of *trans*-3,3′,5,5′-tetrahydroxy-4′-methoxystilbene (**13**) (125 MHz, MeOH-d_4, 30 °C).

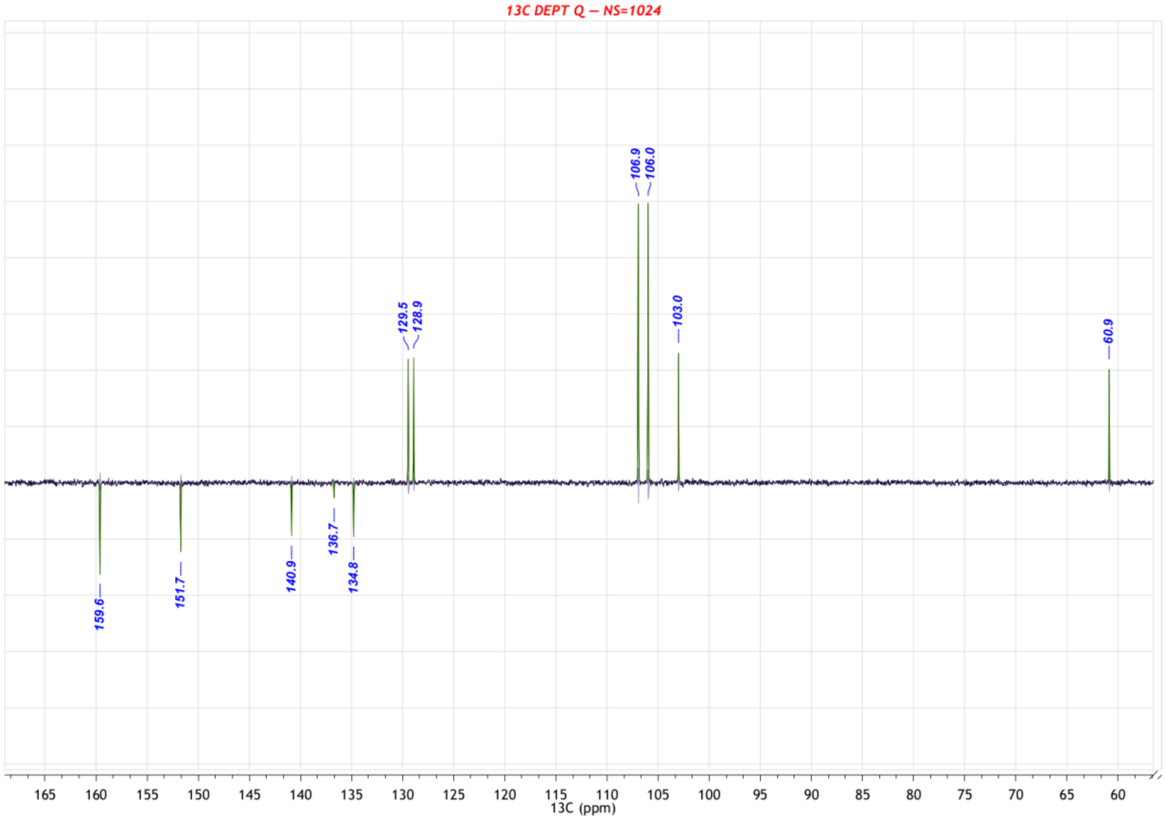

Figure S3. ^{1}H NMR spectrum of yuccalechin A (**15**) (500 MHz, MeOH-d_4, 30 °C).

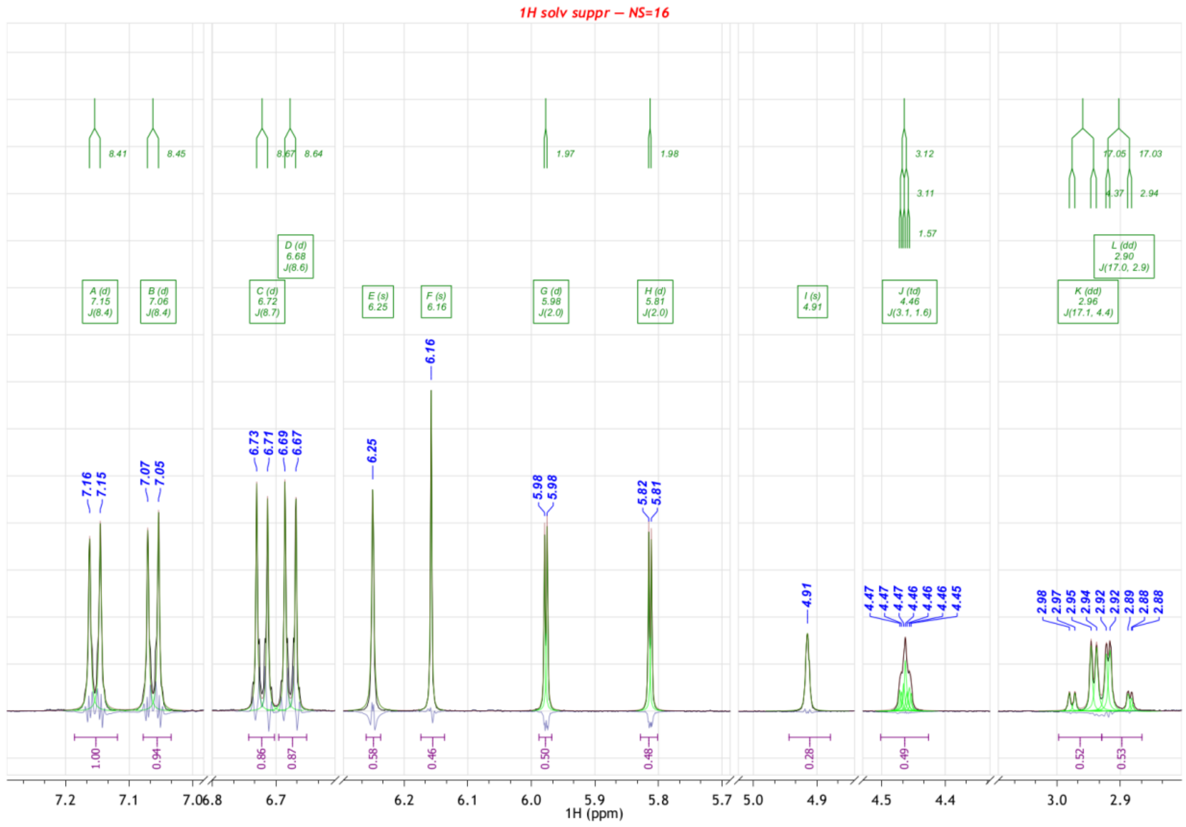

Figure S4. ^{13}C NMR spectrum of yuccalechin A (**15**) (125 MHz, MeOH-d_4, 30 °C).

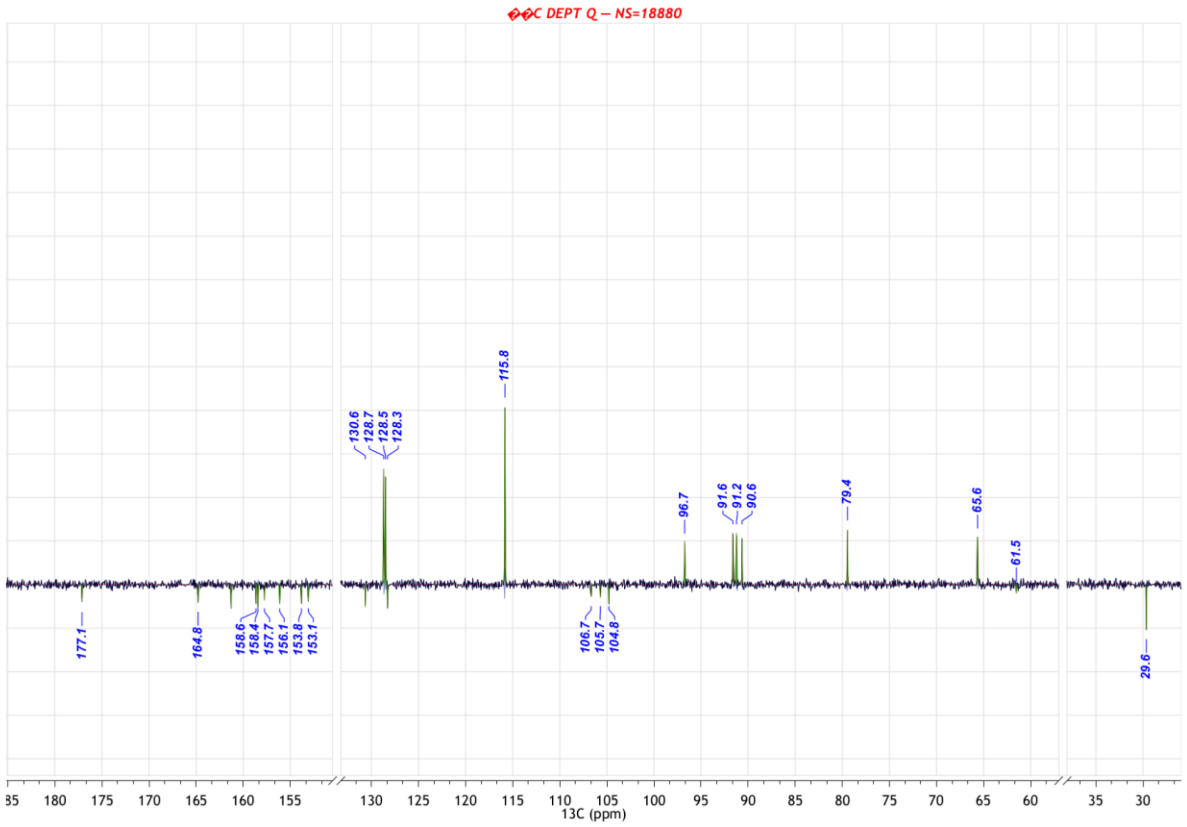

Figure S5. ¹H-¹H COSY NMR spectrum of yuccalechin A (**15**) (500 MHz, MeOH-d_4, 30 °C).

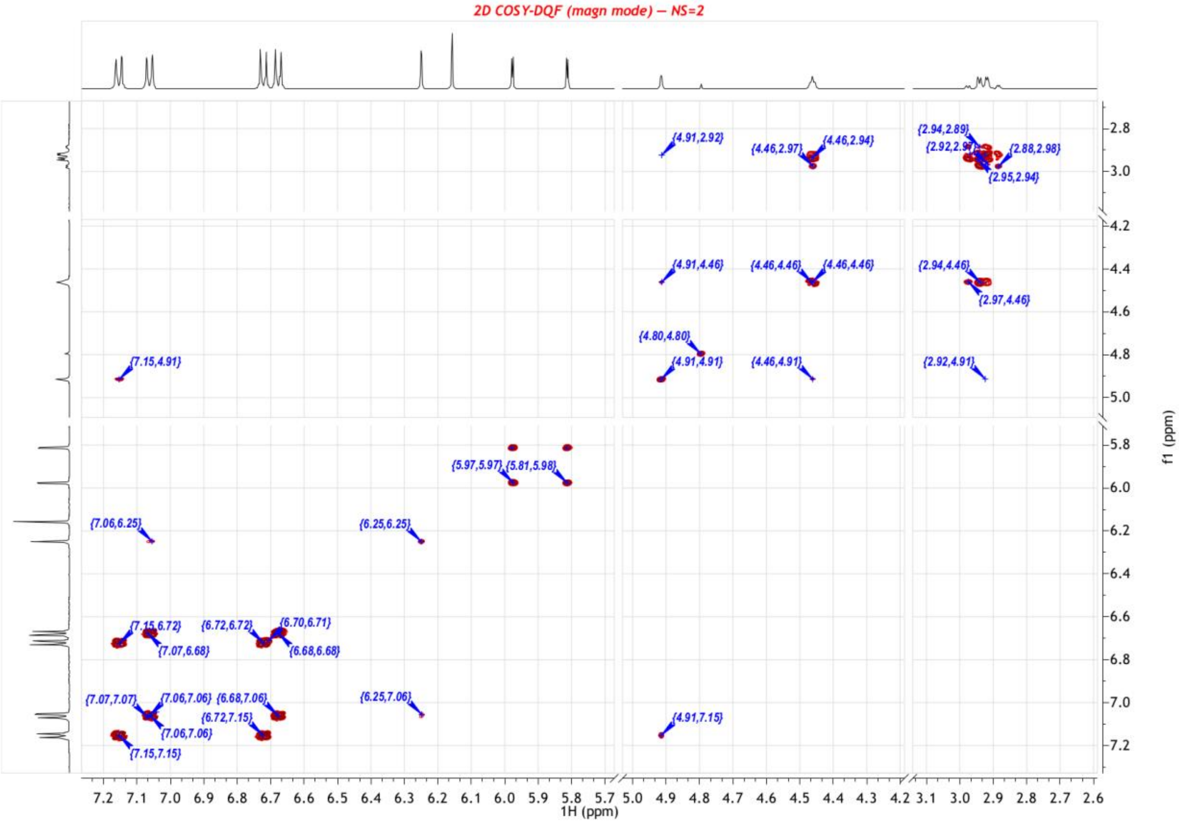

Figure S6. ¹H-¹H ROESY (250 ms) NMR spectrum of yuccalechin A (**15**) (500 MHz, MeOH-d_4, 30 °C).

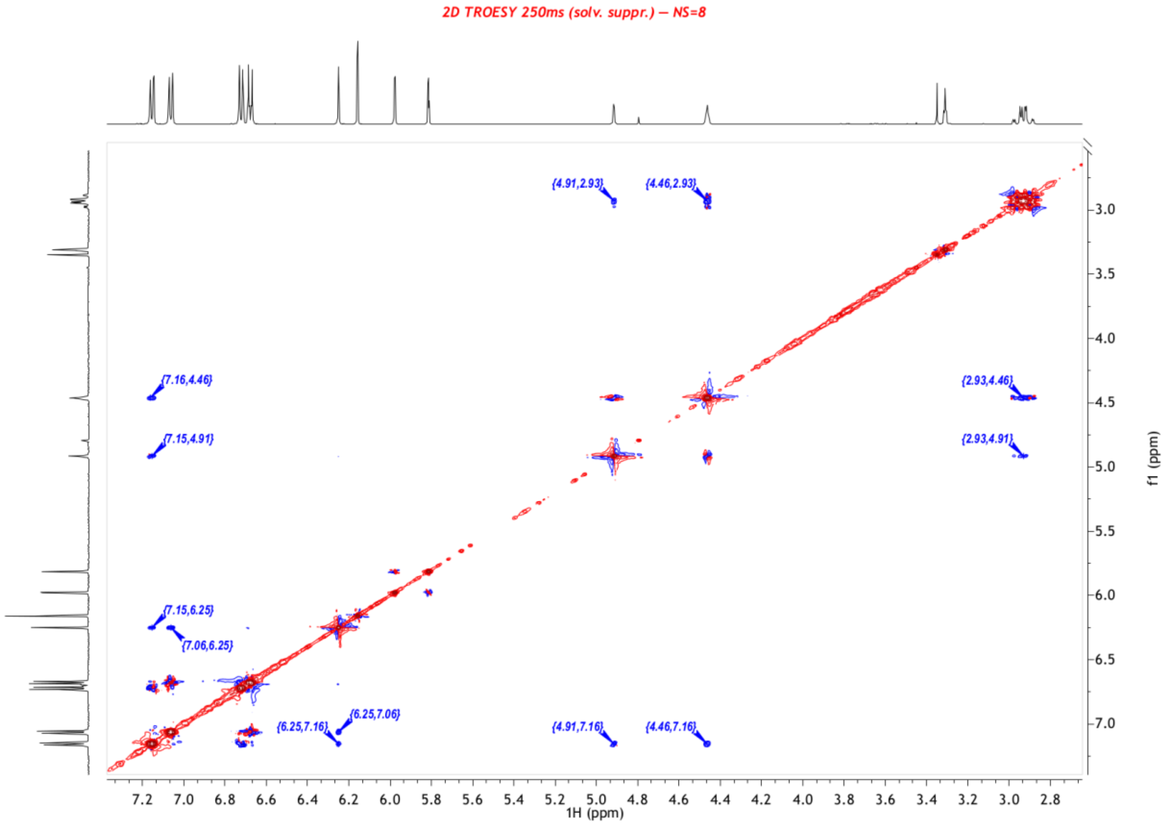

Figure S7. ^{1}H-^{13}C HSQC NMR spectrum of yuccalechin A (**15**) (500/125 MHz, MeOH-d_4, 30 °C).

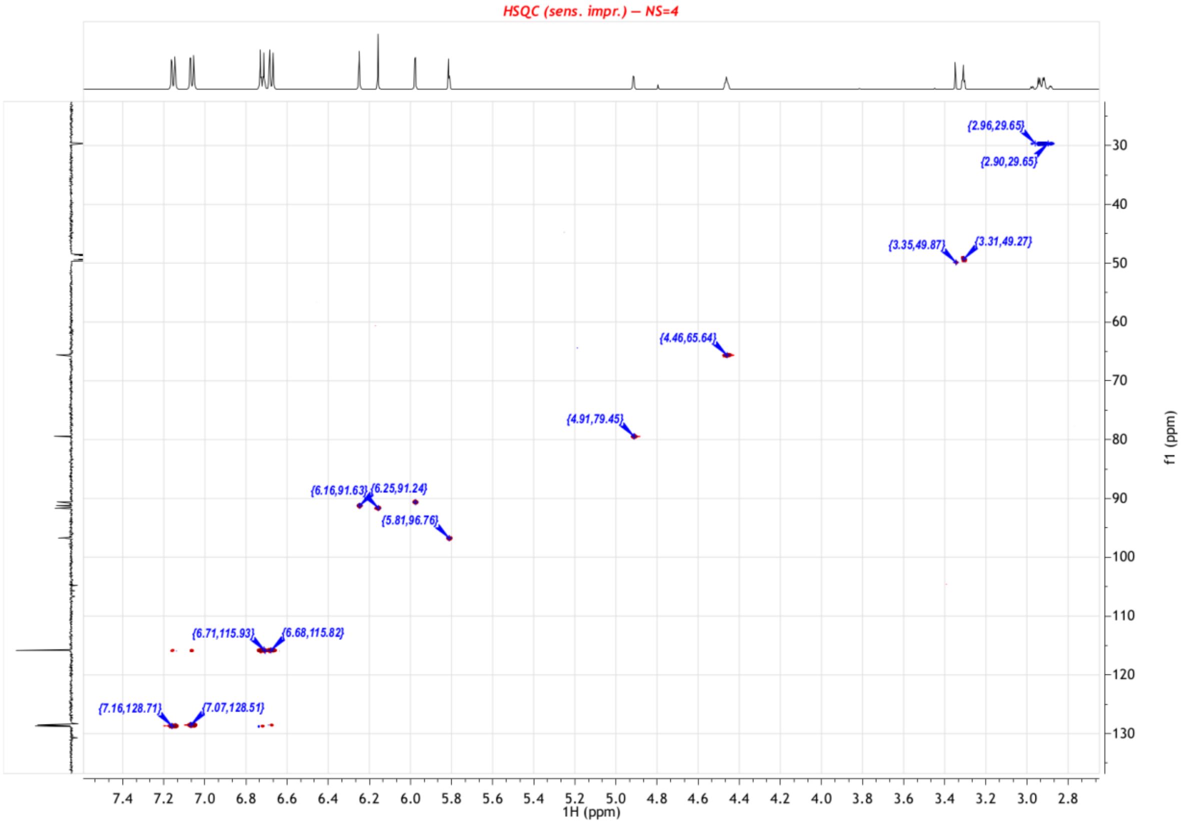

Figure S8. ^{1}H-^{13}C H2BC NMR spectrum of yuccalechin A (**15**) (500/125 MHz, MeOH-d_4, 30 °C).

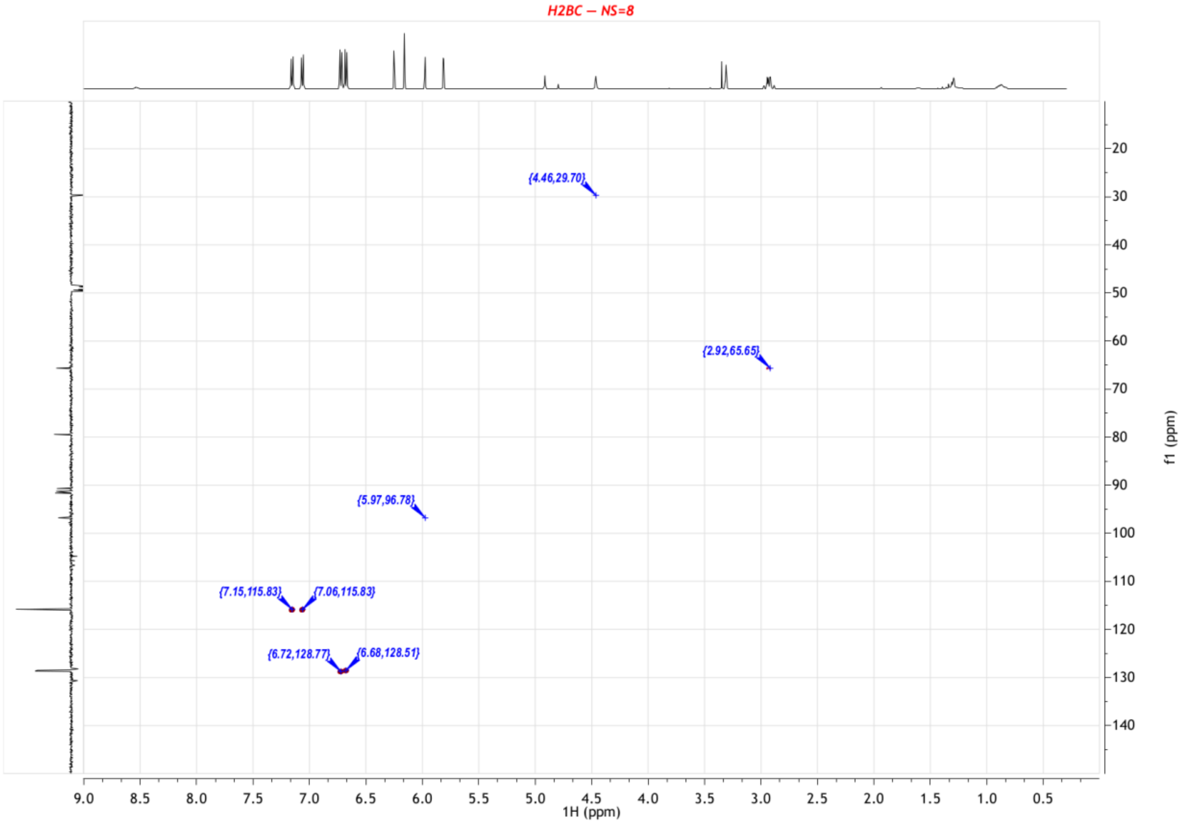

Figure S9. ^{1}H-^{13}C HMBC (8 Hz) NMR spectrum of yuccalechin A (**15**) (500/125 MHz, MeOH-d_4, 30 °C).

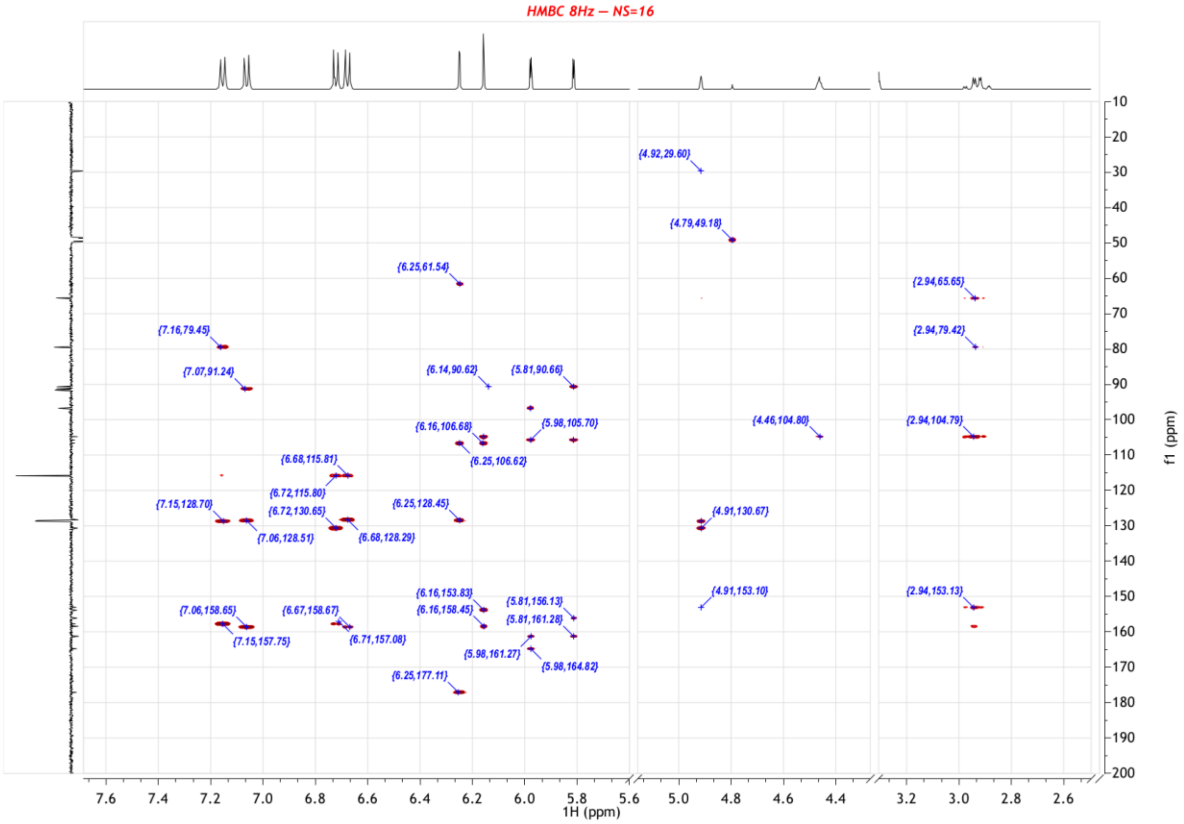

Figure S10. Optimized conformers of yuccalechin A (**15**) in DFT/B3LYP/6-31G(d,p)/IEFPCM/methanol level of theory.

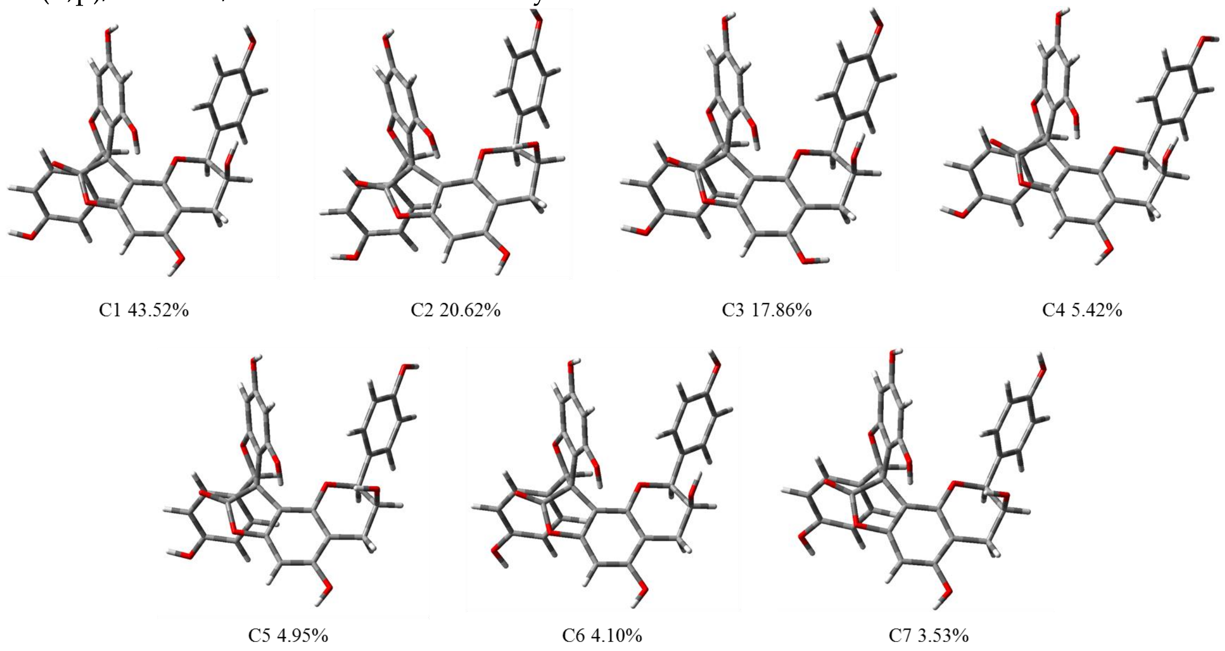

Figure S11. Calculated DP4+ probabilities of yuccalechin A (**15**) using mpw1pw91/6-111G+(d,p)/ IEFPCM/methanol level of theory. Isomer 1 is 2″*R*,3″*S*,2*R*,3*R* and isomer 2 is 2″*R*,3″S,2*S*,3*R*.

	A	B	C	D	E	F	G	H
1	Functional		Solvent?		Basis Set		Type of Data	
2	mPW1PW91		PCM		6-311+G(d,p)		Unscaled Shifts	
3								
4			Isomer 1	Isomer 2	Isomer 3	Isomer 4	Isomer 5	Isomer 6
5	sDP4+ (H data)		12.94%	87.06%	-	-	-	-
6	sDP4+ (C data)		99.97%	0.03%	-	-	-	-
7	sDP4+ (all data)		99.77%	0.23%	-	-	-	-
8	uDP4+ (H data)		99.70%	0.30%	-	-	-	-
9	uDP4+ (C data)		97.47%	2.53%	-	-	-	-
10	uDP4+ (all data)		99.99%	0.01%	-	-	-	-
11	DP4+ (H data)		98.03%	1.97%	-	-	-	-
12	DP4+ (C data)		100.00%	0.00%	-	-	-	-
13	DP4+ (all data)		100.00%	0.00%	-	-	-	-

Table S1. Calculated and experimental chemical shift values used for DP4+ calculation for (**15**).

Atom	Exp.	Isomer 1	Isomer 2
C	161.3	168.2	167.9
C	96.7	100.2	99.7
C	156.1	161.7	161.4
C	105.7	109.7	109.0
C	164.8	170.4	170.8
C	90.6	96.3	95.3
C	91.2	94.9	95.4
C	61.5	66.9	67.0
C	177.1	185.2	185.1
C	153.8	161.2	161.2
C	106.7	104.6	105.0
C	91.6	96.2	96.5
C	158.4	165.7	165.1
C	104.8	109.8	111.8
C	153.1	160.8	160.6
C	128.3	131.7	131.6
C	128.5	136.5	136.6
C	115.8	120.2	120.1
C	158.6	165.4	165.5
C	115.8	119.6	120.1
C	128.5	136.1	136.3
C	29.6	30.1	31.0
C	65.6	70.4	72.5
C	79.4	84.2	87.6
C	130.6	136.0	135.0
C	128.7	134.5	138.3
C	115.8	120.1	120.3
C	157.7	164.7	165.2

C	115.8	121.2	121.0
C	128.7	136.1	135.3
H	5.81	6.16	5.97
H	5.98	6.34	6.12
H	6.25	6.23	6.40
H	6.16	6.33	6.37
H	7.06	7.94	8.00
H	6.68	7.11	7.11
H	6.68	6.90	7.01
H	7.06	7.05	7.19
H	2.96	3.02	3.33
H	2.9	3.16	2.67
H	4.46	4.08	3.99
H	4.91	5.23	4.61
H	7.15	7.42	7.58
H	6.72	6.95	7.20
H	6.72	7.23	7.13
H	7.15	7.60	7.83

Figure S12. HRESIMS (Q-TOF) analysis of yuccalechin A (**15**) in negative ion mode.

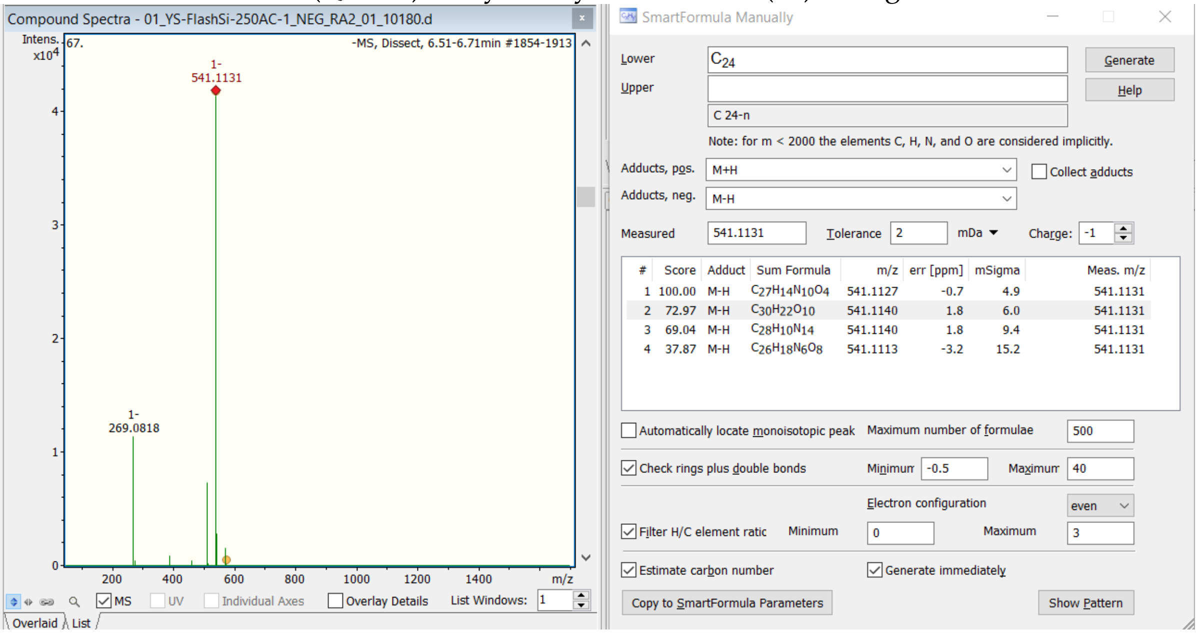

Figure S13. ^{1}H NMR spectrum of aromadendrin (**16**) (500 MHz, MeOH-d_4, 30 °C).

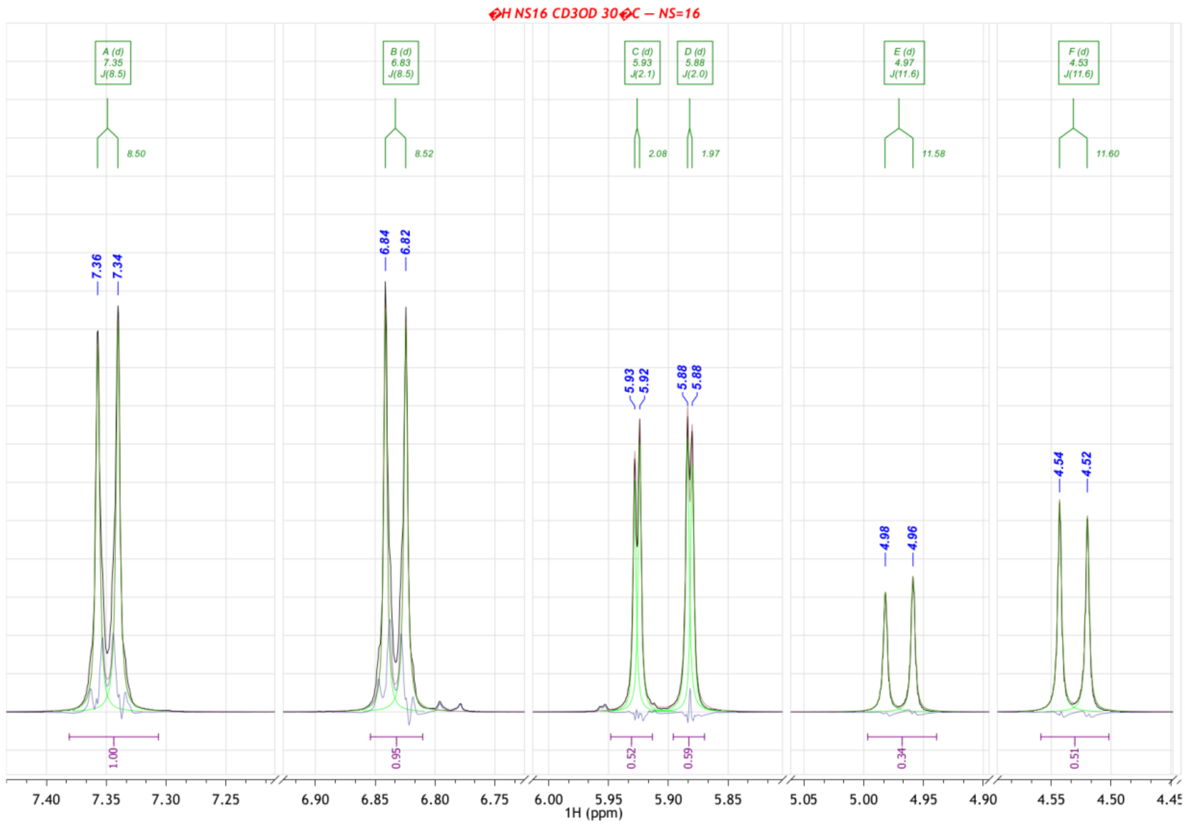

Figure S14. ^{13}C NMR spectrum of aromadendrin (**16**) (125 MHz, MeOH-d_4, 30 °C).

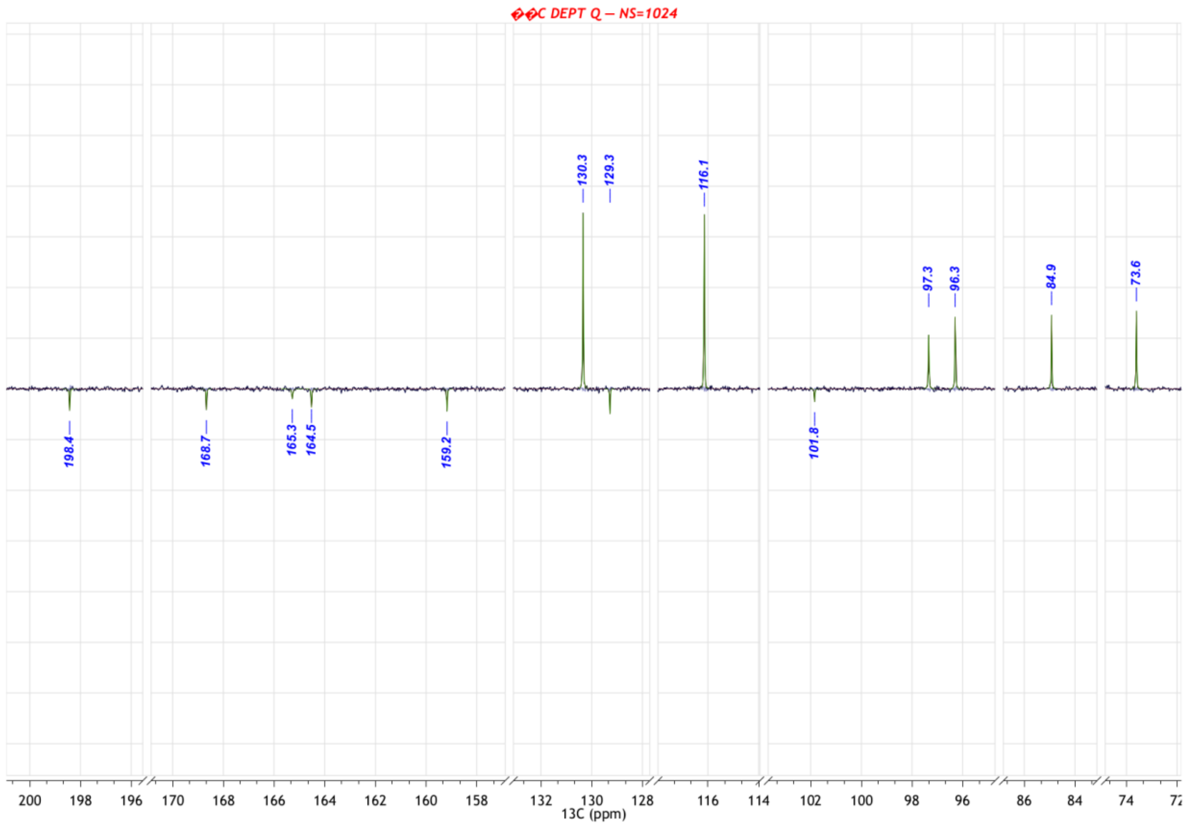

Figure S15. ^{1}H NMR spectrum of *trans*-resveratrol (**21**) (500 MHz, MeOH-d_4, 30 °C).

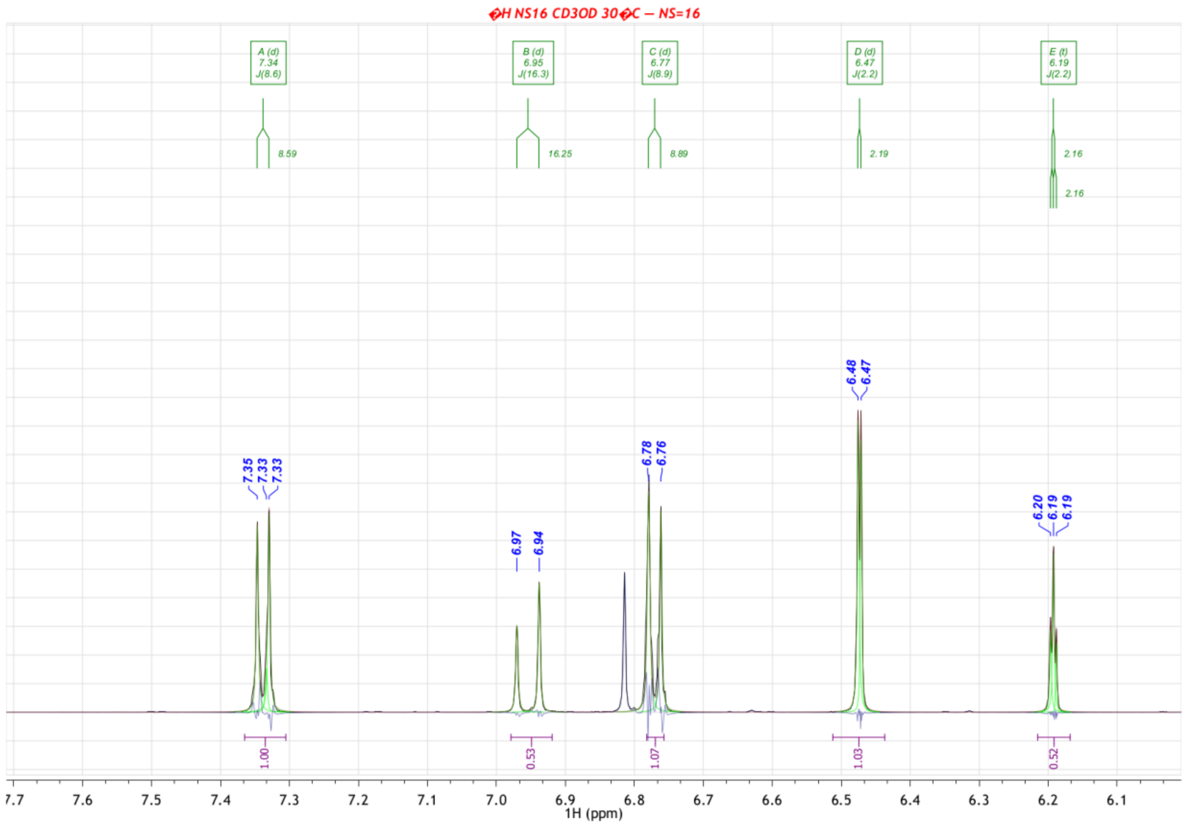

Figure S16. ^{13}C NMR spectrum of *trans*-resveratrol (**21**) (125 MHz, MeOH-d_4, 30 °C).

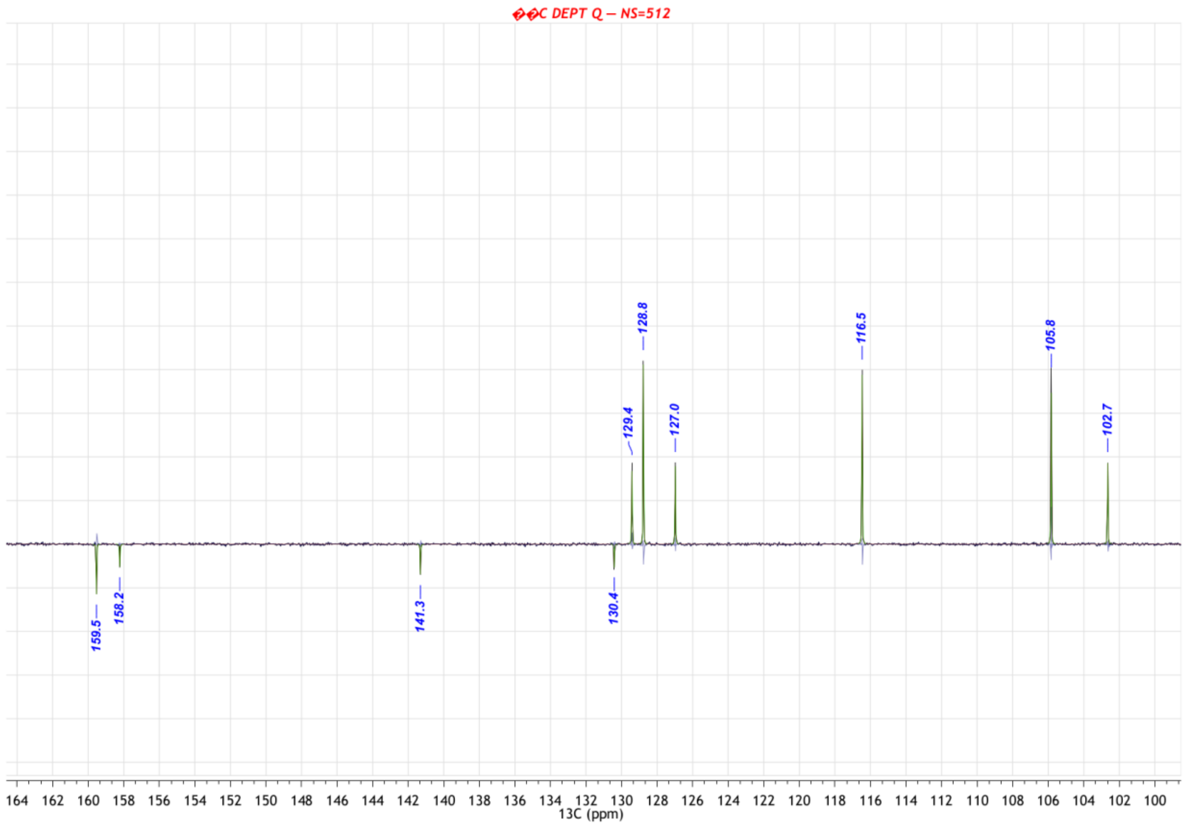

Figure S17. ^{1}H NMR spectrum of yuccalechin B (**26**) (500 MHz, MeOH-d_4, 30 °C).

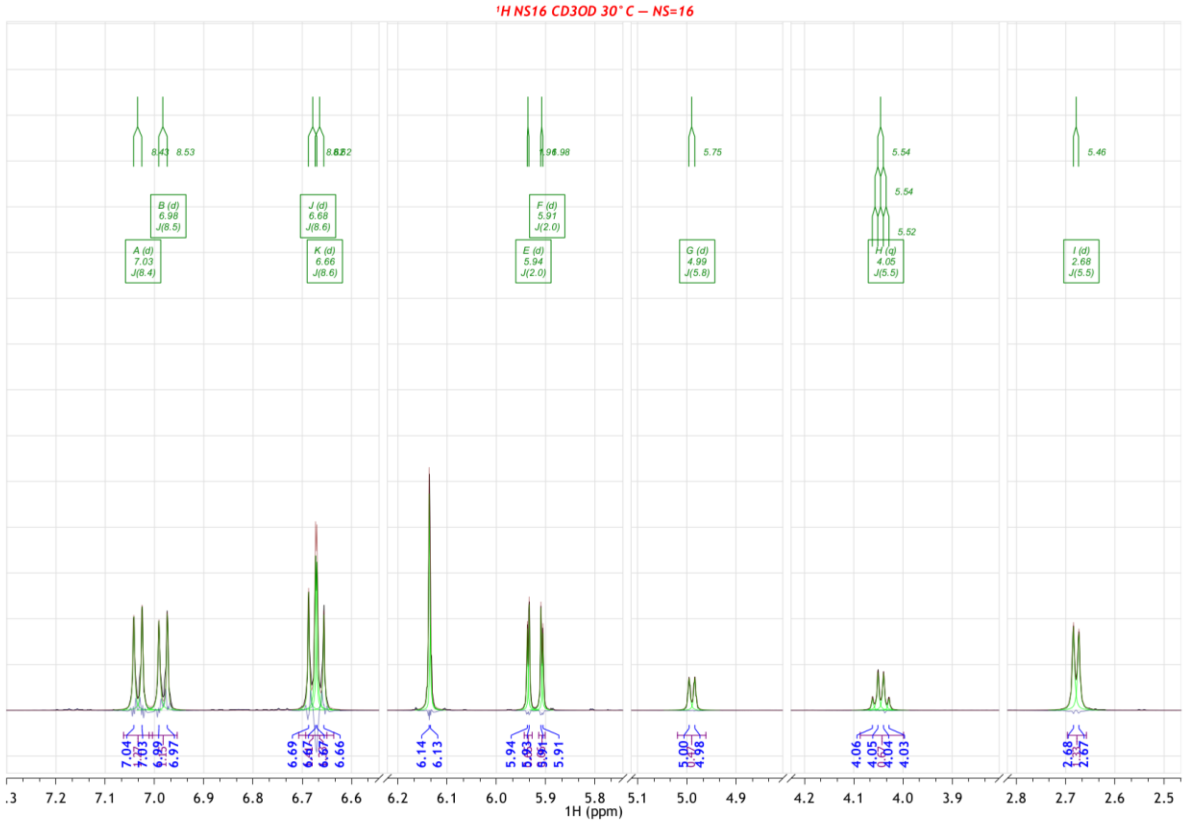

Figure S18. ^{13}C NMR spectrum of yuccalechin B (**26**) (125 MHz, MeOH-d_4, 30 °C).

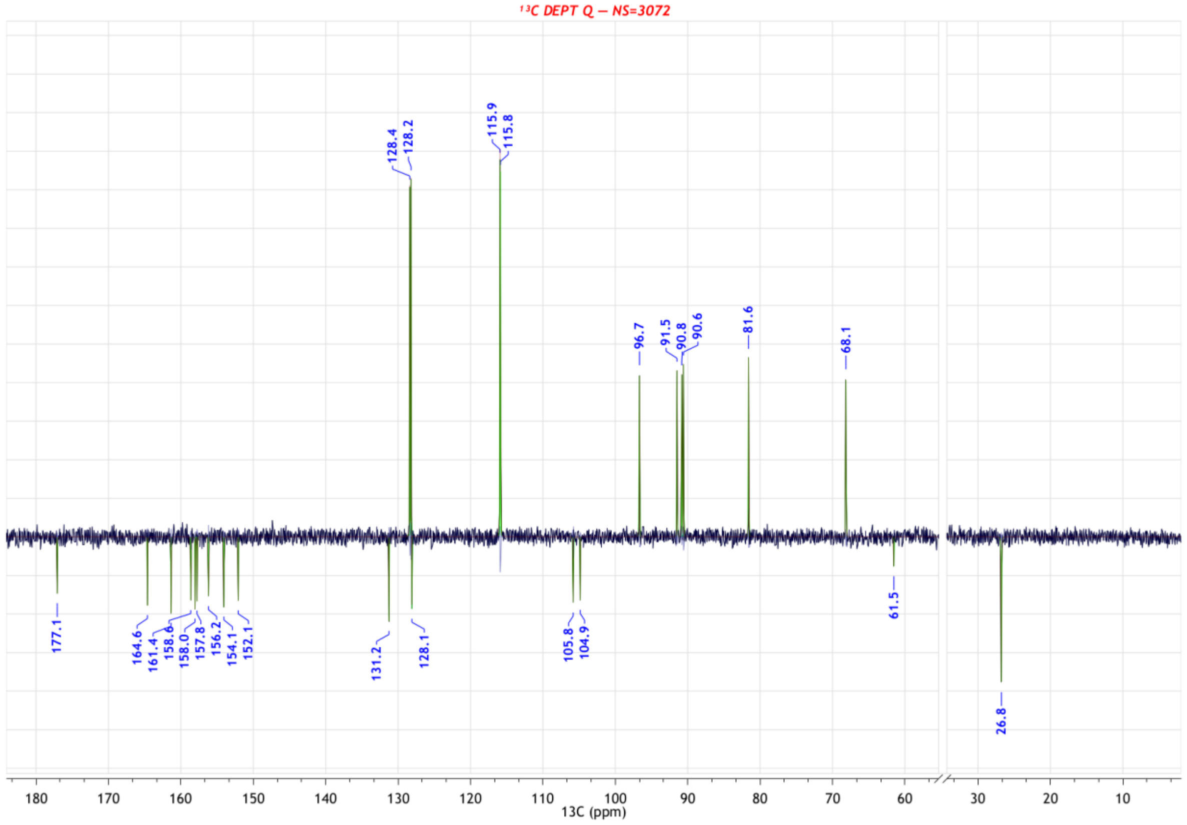

Figure S19. ^{1}H-^{1}H COSY NMR spectrum of yuccalechin B (**26**) (500 MHz, MeOH-d_4, 30 °C).

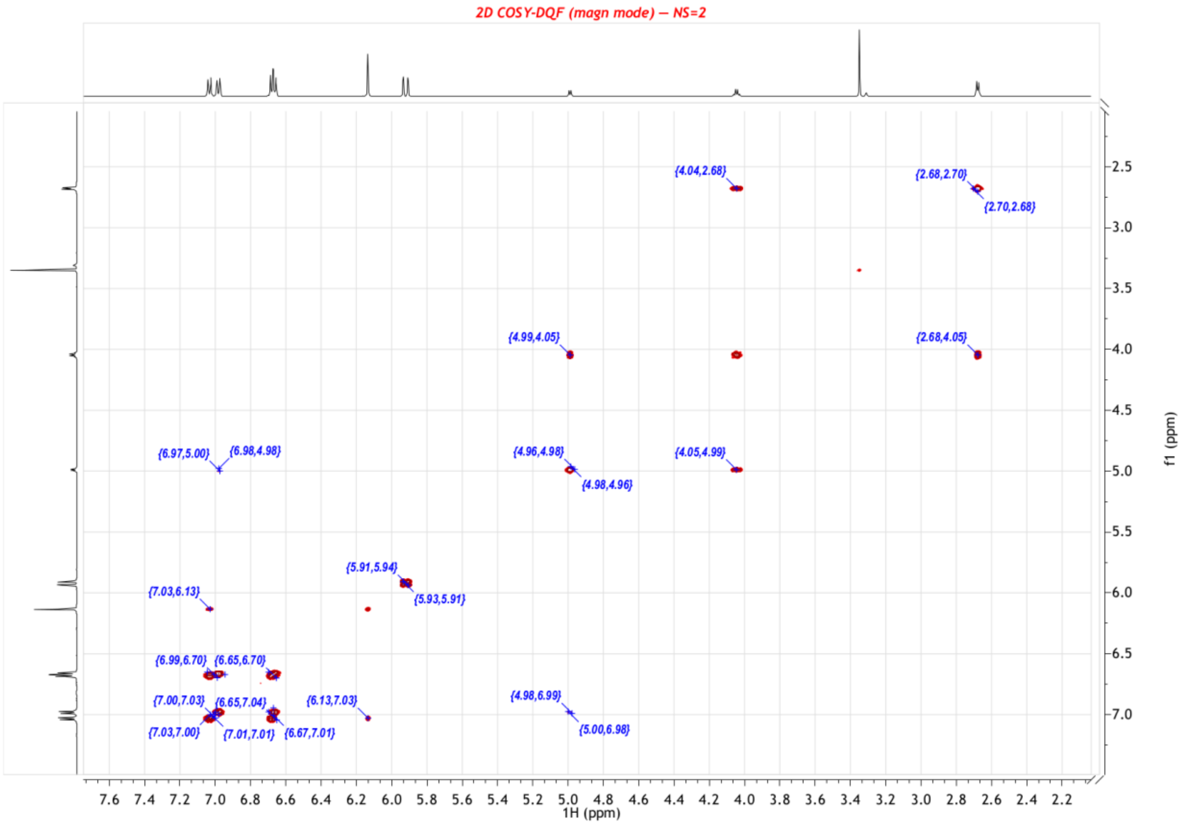

Figure S20. ^{1}H-^{1}H ROESY (250 ms) NMR spectrum of yuccalechin B (**26**) (500 MHz, MeOH-d_4, 30 °C).

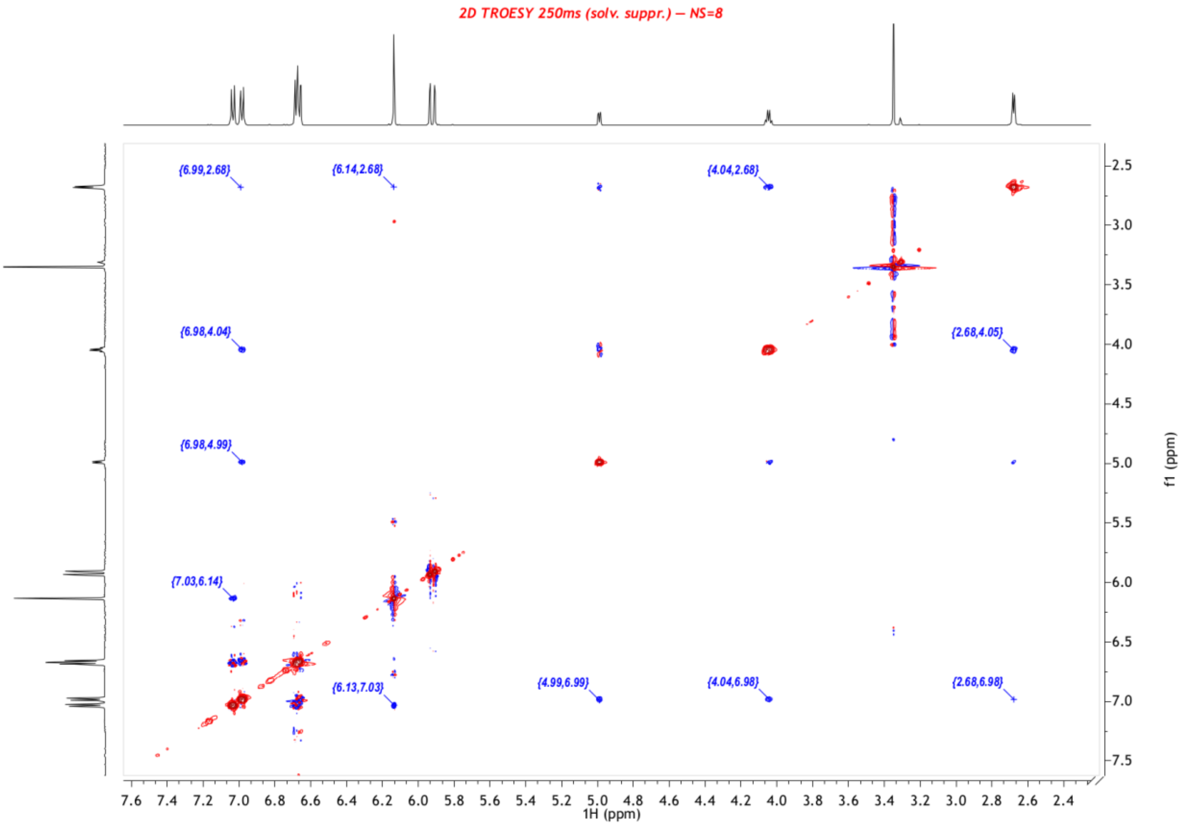

Figure S21. ^1H-^{13}C HSQC NMR spectrum of yuccalechin B (**26**) (500/125 MHz, MeOH-d_4, 30 °C).

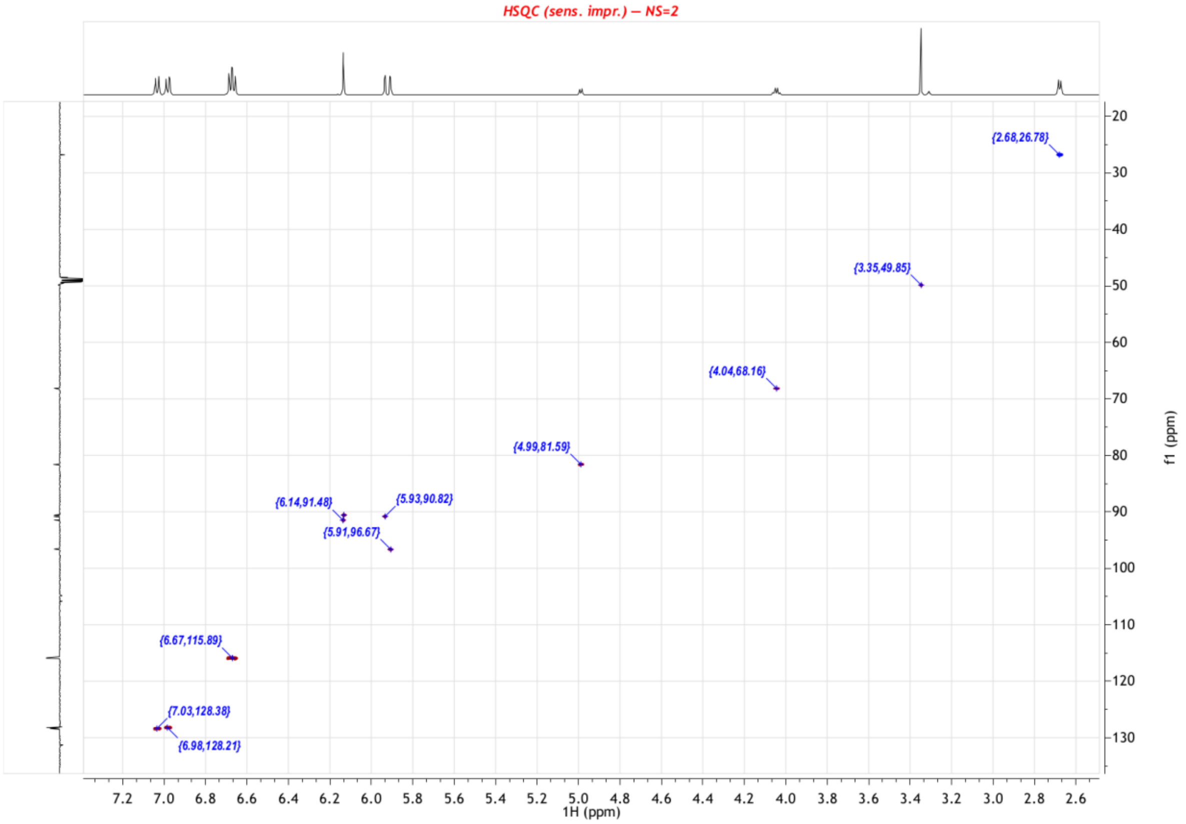

Figure S22. ^1H-^{13}C H2BC NMR spectrum of yuccalechin B (**26**) (500/125 MHz, MeOH-d_4, 30 °C).

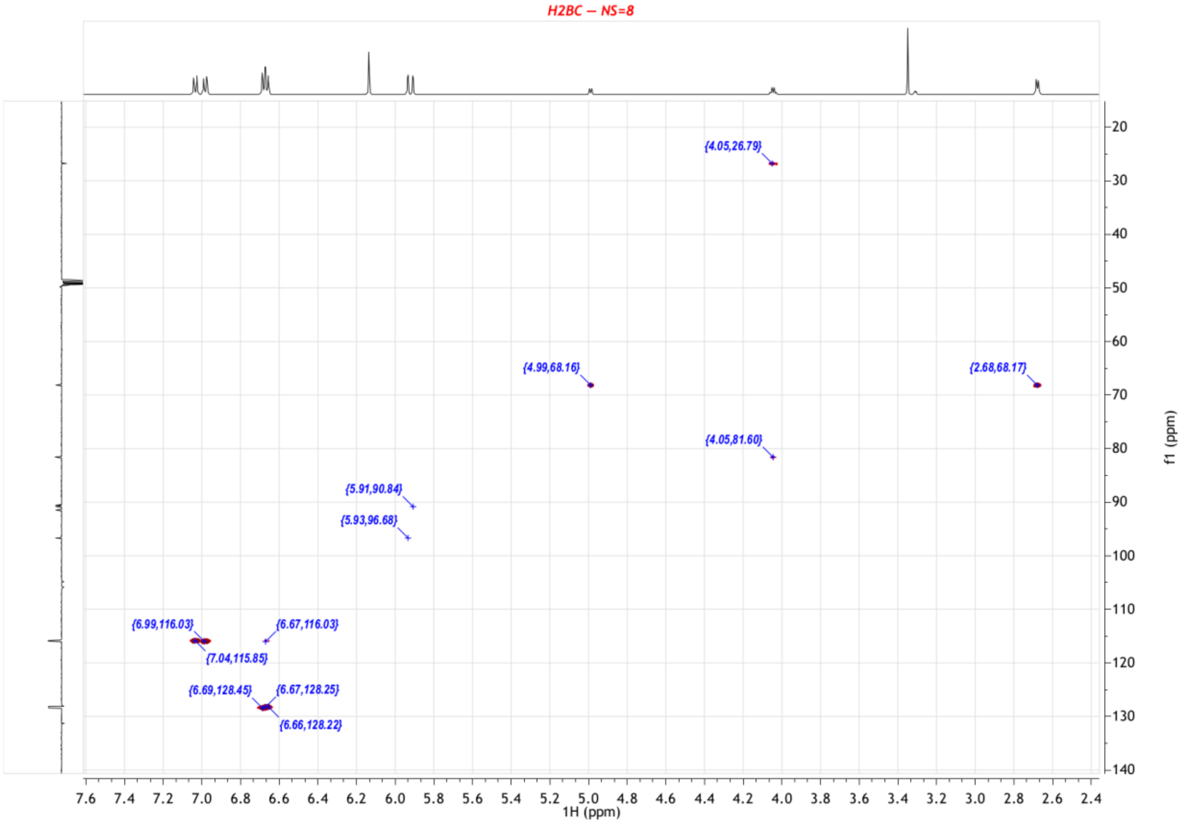

Figure S23. ^{1}H-^{13}C HMBC (8Hz) NMR spectrum of yuccalechin B (**26**) (500/125 MHz, MeOH-d_4, 30 °C).

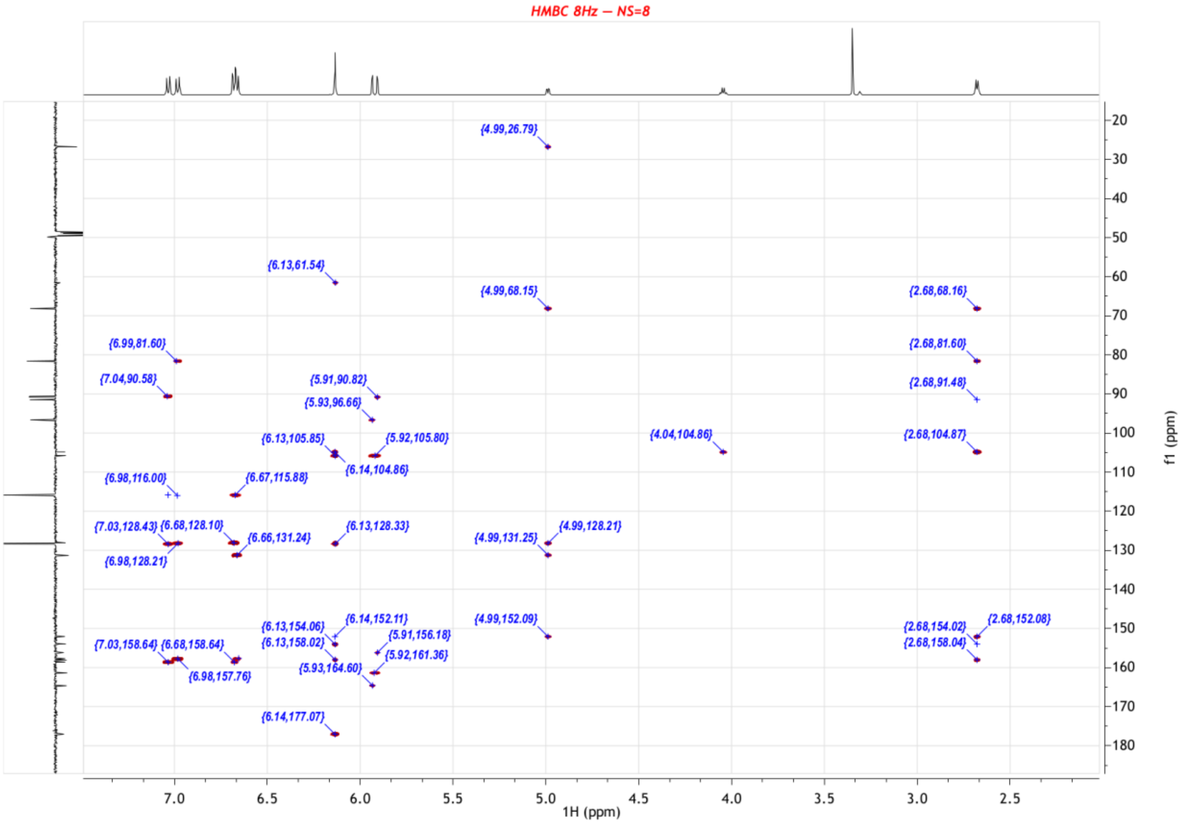

Figure S24. Optimized conformers of yuccalechin B (**26**) in DFT/B3LYP/6-31G(d,p)/IEFPCM/MeOH level of theory.

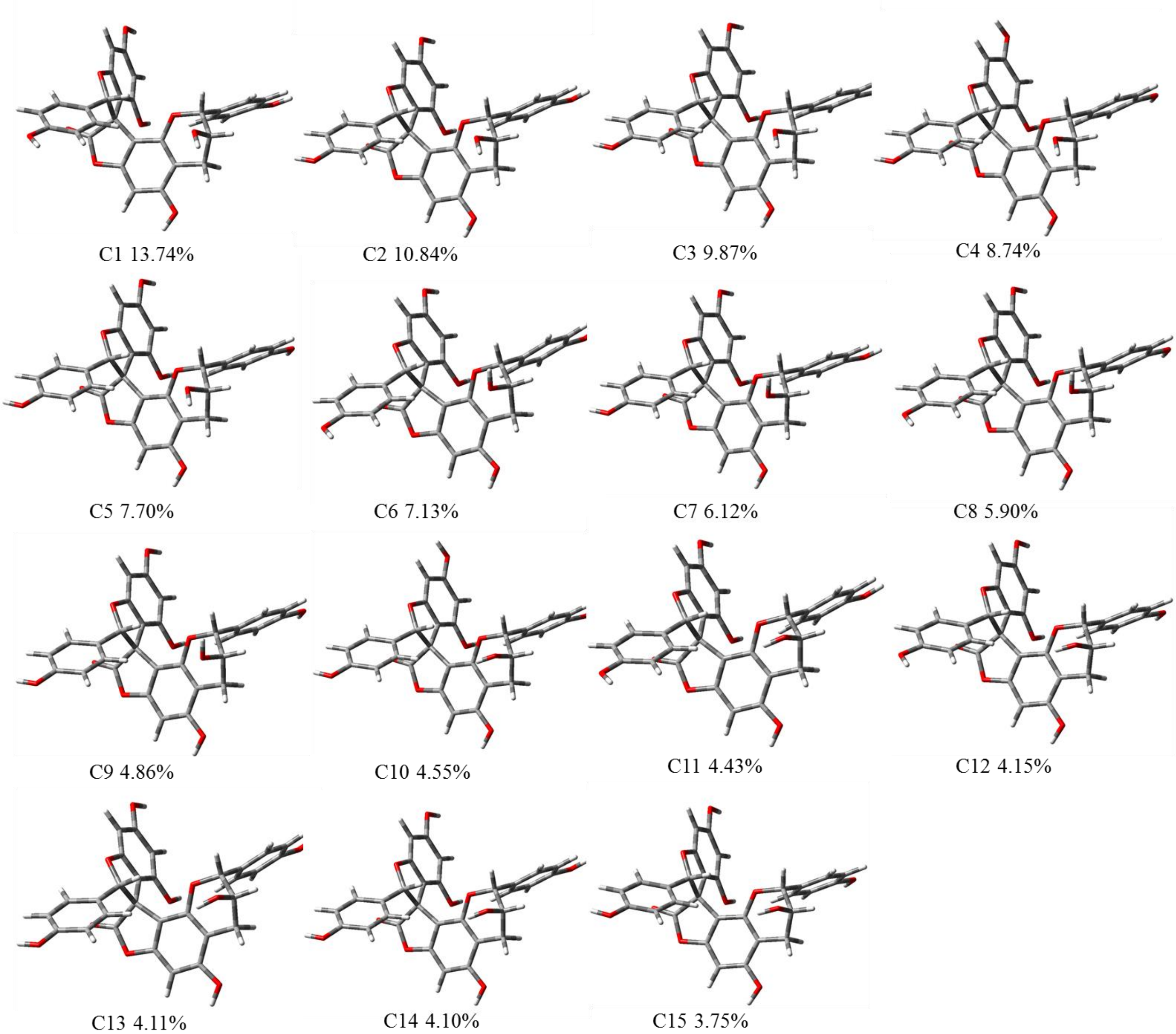

Figure S25. Calculated DP4+ probabilities of yuccalechin B (**26**) using mpw1pw91/6-111G+(d,p)/IEFPCM/ methanol level of theory. Isomer 1 is 2″*R*,3″*S*,2*S*,3*S* and isomer 2 is 2″*R*,3″*S*,2*R*,3*S*.

F12 =Main!G11

	A–B	C	D	E	F	G	H
1	Functional	Solvent?		Basis Set		Type of Data	
2	mPW1PW91	PCM		6-311+G(d,p)		Unscaled Shifts	
3							
4		Isomer 1	Isomer 2	Isomer 3	Isomer 4	Isomer 5	Isomer 6
5	sDP4+ (H data)	2.85%	97.15%	-	-	-	-
6	sDP4+ (C data)	1.35%	98.65%	-	-	-	-
7	sDP4+ (all data)	0.04%	99.96%	-	-	-	-
8	uDP4+ (H data)	11.52%	88.48%	-	-	-	-
9	uDP4+ (C data)	99.36%	0.64%	-	-	-	-
10	uDP4+ (all data)	95.31%	4.69%	-	-	-	-
11	DP4+ (H data)	0.38%	99.62%	-	-	-	-
12	DP4+ (C data)	68.16%	31.84%	-	-	-	-
13	DP4+ (all data)	0.81%	99.19%	-	-	-	-

Table S2. Calculated and experimental chemical shift values used for DP4+ calculation for (**26**).

Atom	Exp.	Isomer 1	Isomer 2
C	161.4	167.6	167.6
C	96.7	99.0	99.1
C	156.2	161.1	160.9
C	105.8	110.3	110.6
C	164.6	171.3	172.3
C	90.8	94.8	95.5
C	90.6	95.3	95.0
C	61.5	67.8	67.8
C	177.1	185.9	186.1
C	154.1	160.7	160.6
C	105.8	110.1	109.3
C	91.5	95.9	95.2
C	158	164.5	164.3
C	104.9	108.7	108.6
C	152.1	160.1	158.8
C	128.1	132.3	132.3
C	128.4	136.6	136.6
C	115.8	120.4	120.4
C	158.6	165.3	165.3
C	115.8	119.5	119.8
C	128.4	136.1	136.4
C	26.8	32.2	25.9
C	68.1	70.7	72.3
C	81.6	84.8	85.5
C	131.2	136.7	137.5
C	128.2	136.4	133.2
C	115.9	120.2	120.5
C	157.8	164.7	163.6
C	115.9	120.3	119.8
C	128.2	136.6	132.9
H	5.91	5.86	5.88
H	5.94	6.05	6.27
H	6.13	6.51	6.46
H	6.14	6.37	6.27
H	7.03	7.68	7.85
H	6.68	7.15	7.18
H	6.68	6.99	7.00
H	7.03	7.51	7.32
H	2.68	3.05	2.26
H	2.68	3.10	2.88
H	4.05	3.99	4.40
H	4.99	4.92	5.50
H	6.98	7.80	7.52

H	6.66	7.15	7.13
H	6.66	7.15	7.01
H	6.98	7.58	7.48

Figure S26. HRESIMS (Q-TOF) analysis of yuccalechin B (**26**) in negative ion mode.

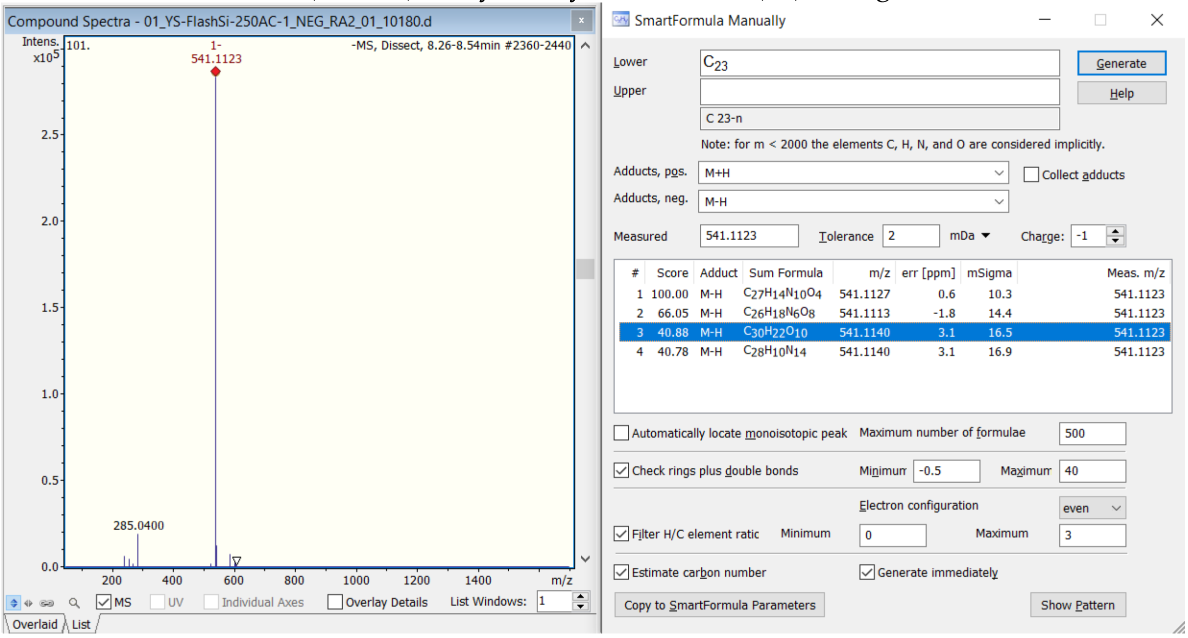

Figure S27. ^{1}H NMR spectrum of yuccalechin C (**29**) (500 MHz, MeOH-d_4, 30 °C).

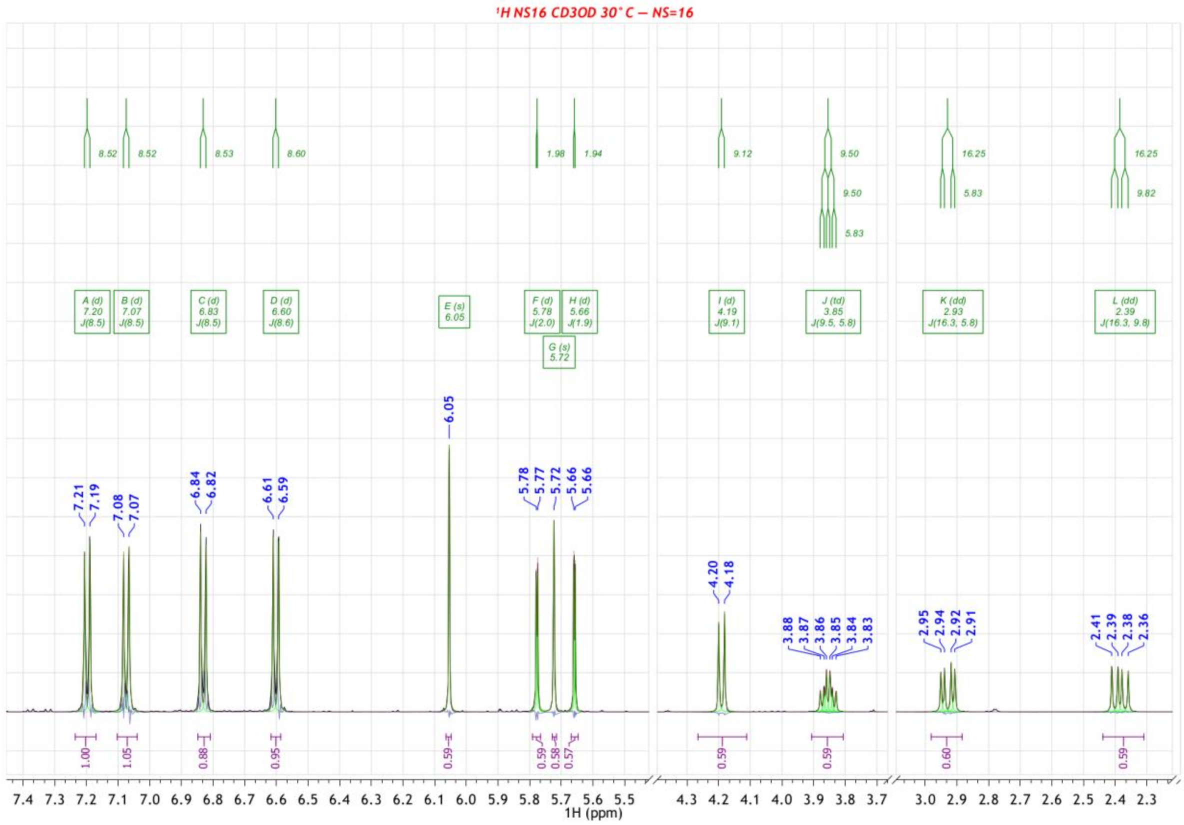

Figure S28. ^{13}C NMR spectrum of yuccalechin C (**29**) (125 MHz, MeOH-d_4, 30 °C).

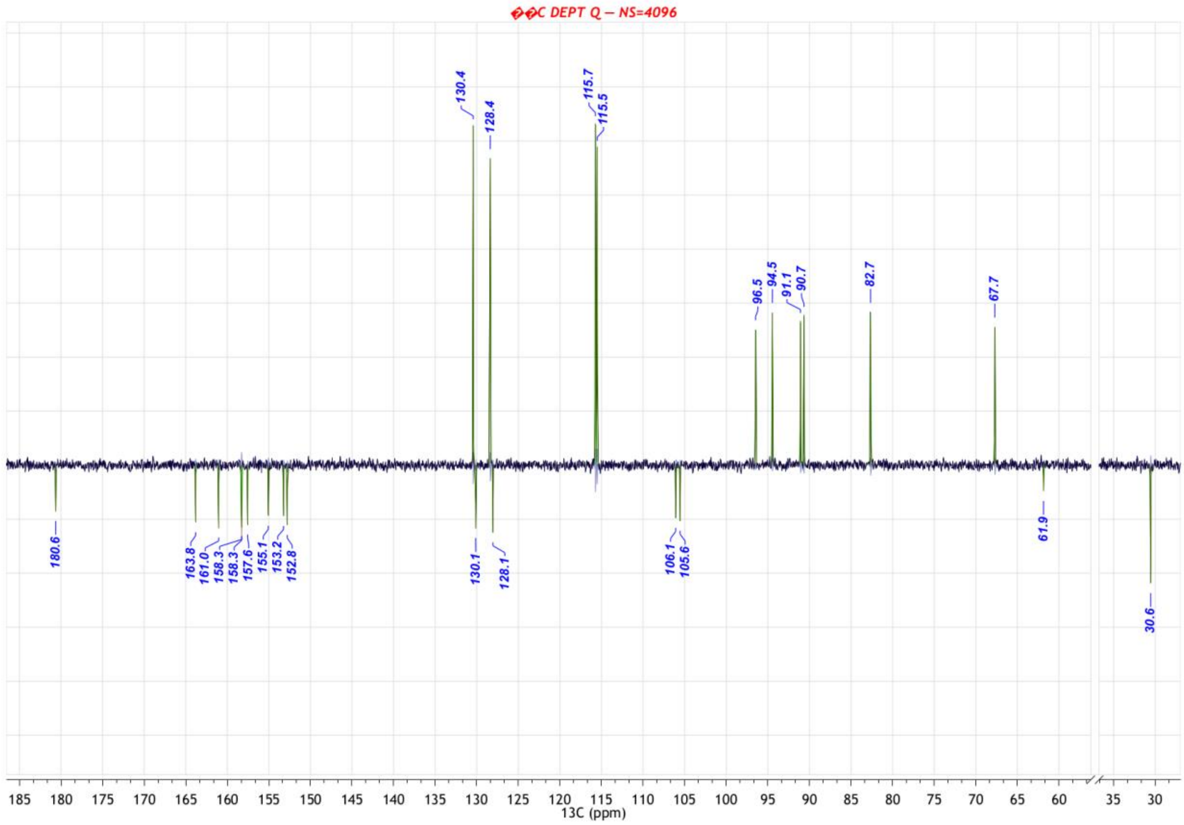

Figure S29. ^{1}H-^{1}H COSY NMR spectrum of yuccalechin C (**29**) (500 MHz, MeOH-d_4, 30 °C).

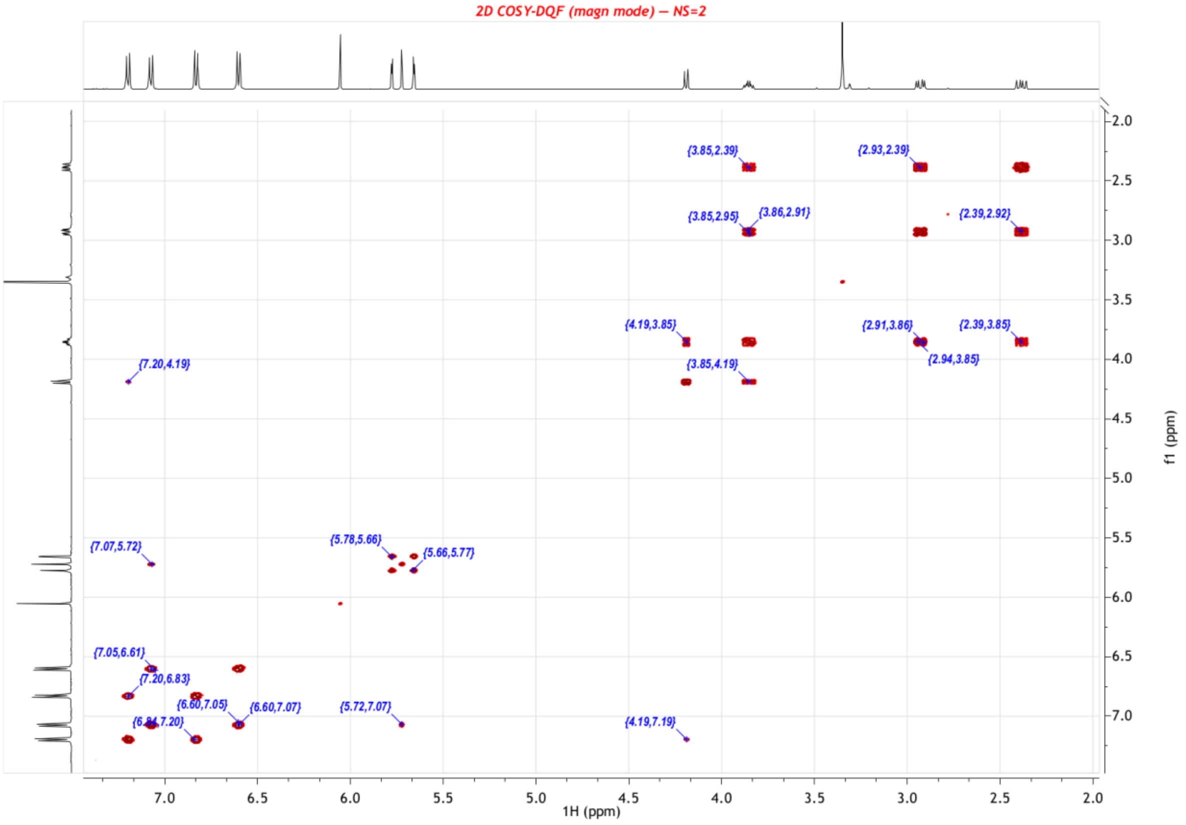

Figure S30. ^{1}H-^{1}H ROESY (250 ms) NMR spectrum of yuccalechin C (**29**) (500 MHz, MeOH-d_4, 30 °C).

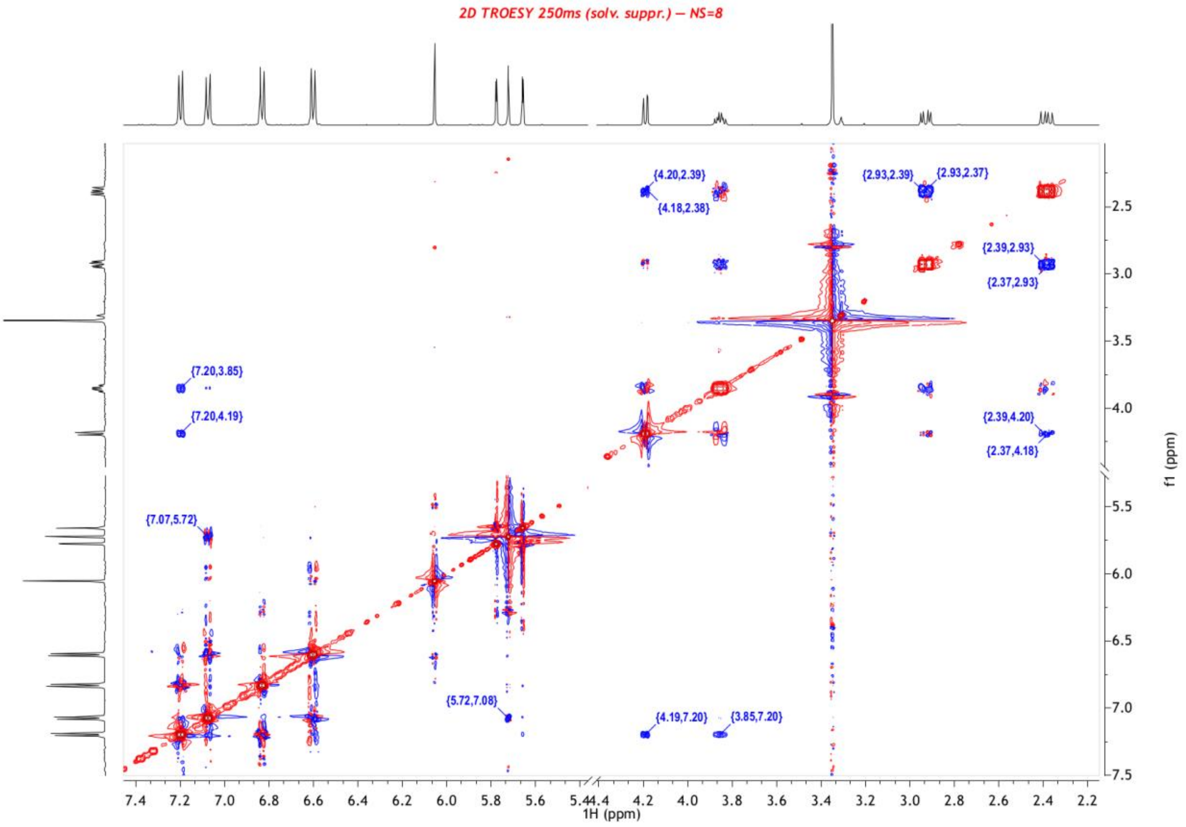

Figure S31. ^{1}H-^{13}C HSQC NMR spectrum of yuccalechin C (**29**) (500/125 MHz, MeOH-*d*4, 30 °C).

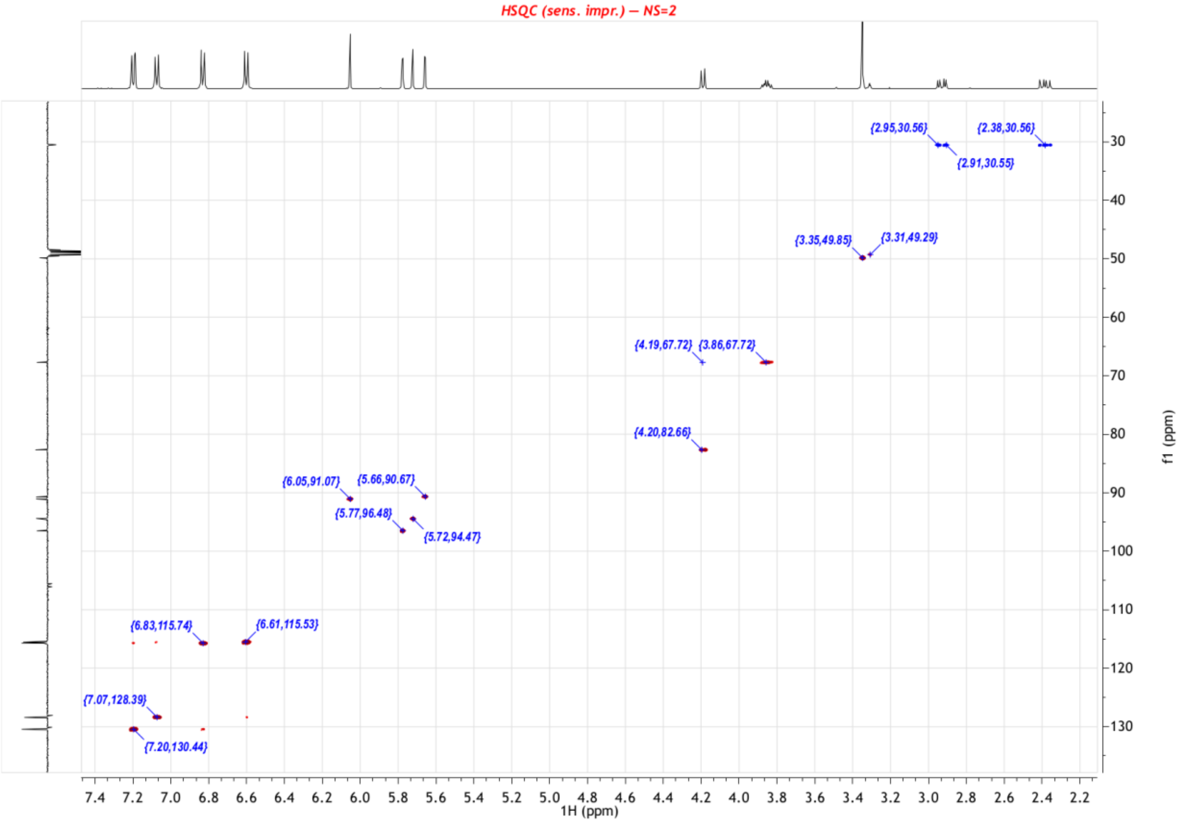

Figure S32. ^{1}H-^{13}C H2BC NMR spectrum of yuccalechin C (**29**) (500/125 MHz, MeOH-*d*4, 30 °C).

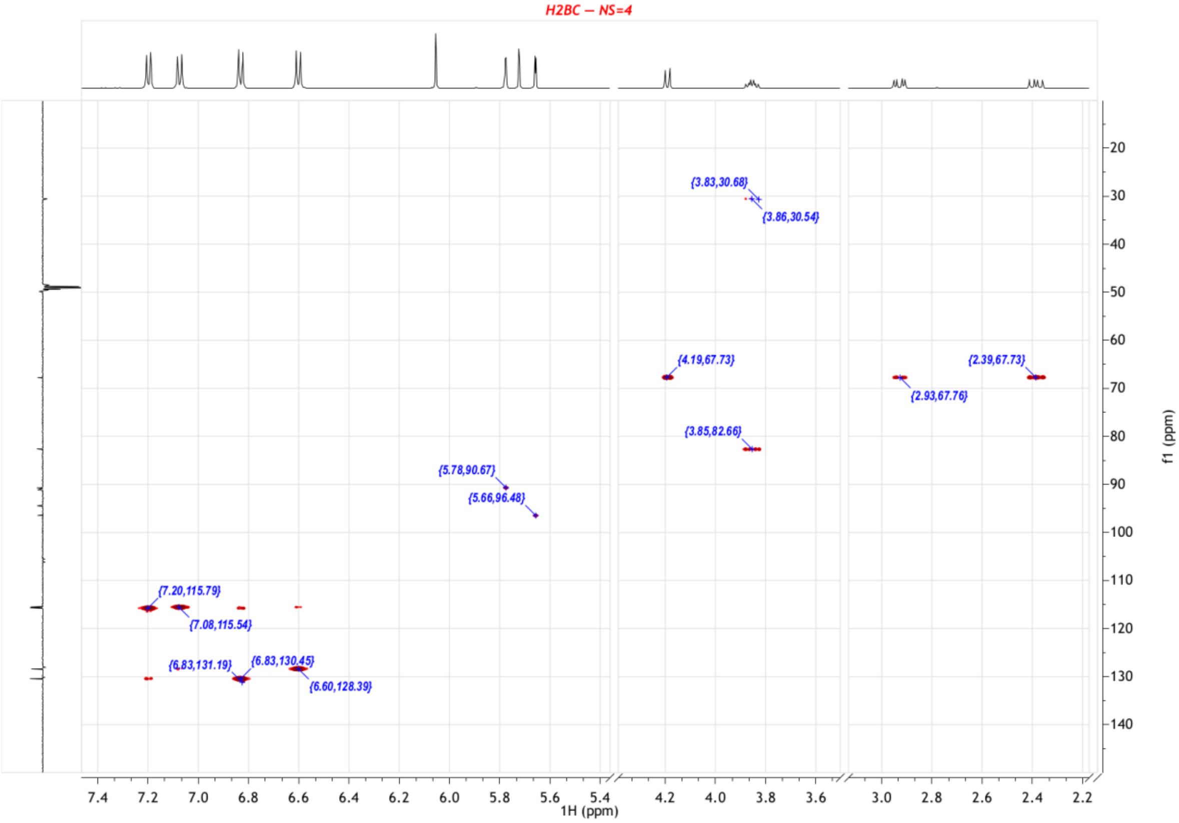

Figure S33. ^1H-^{13}C HMBC (8Hz) NMR spectrum of yuccalechin C (**29**) (500/125 MHz, MeOH-d_4, 30 °C).

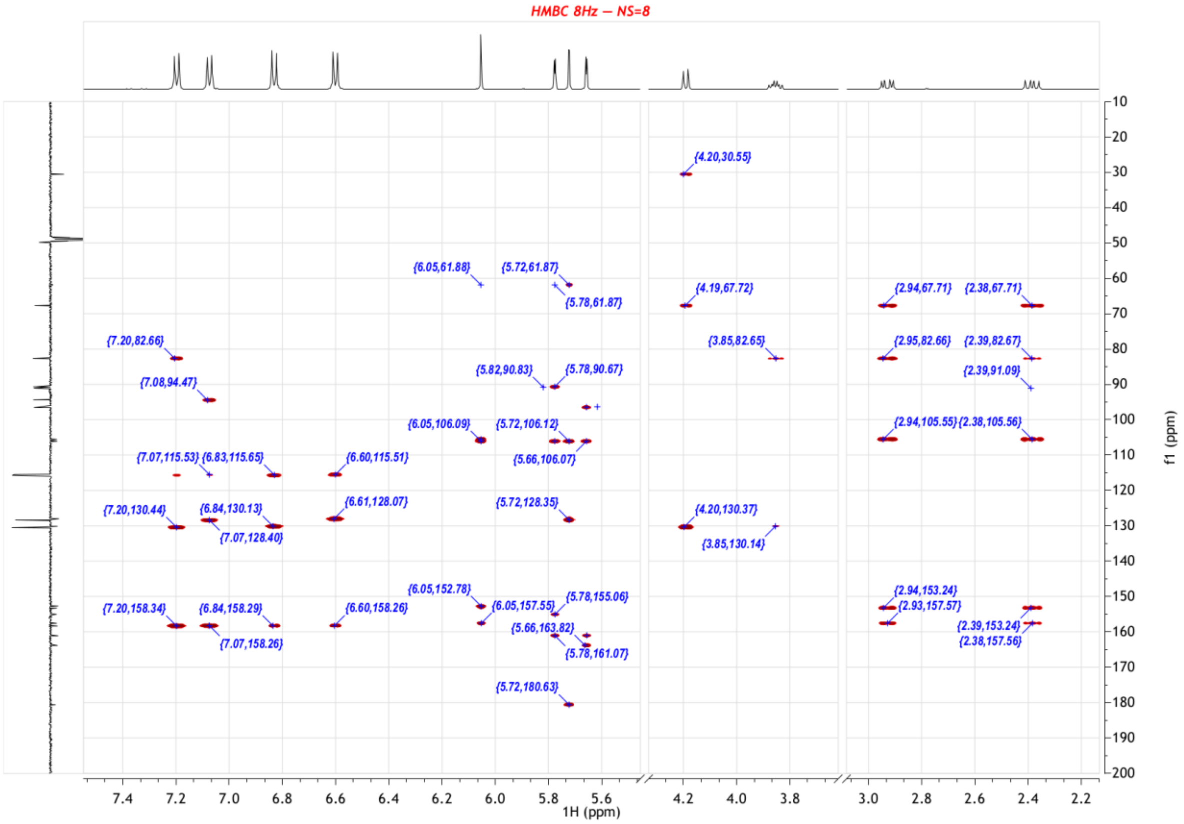

Figure S34. Optimized conformers of yuccalechin C (**29**) and their contribution to Boltzmann averaging at DFT/B3LYP/6-31G(d) level of theory in gas phase.

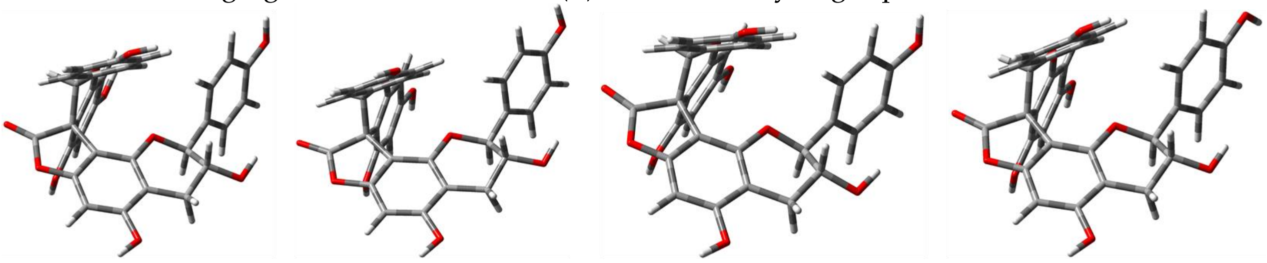

C1 45.79%   C2 20.50%   C3 20.11%   C7 13.60%

Figure S35. Calculated DP4+ probabilities of yuccalechin C (**29**) using mpw1pw91/6-111G+(d,p)/ CPCM/methanol level of theory. Isomer 1 is 2″*R*,3″*R*,2*R*,3*R* and isomer 2 is 2″*R*,3″*R*,2*R*,3*S* and isomer 3 is 2″*R*,3″*R*,2*S*,3*R*.

F12 =Main!G11

Functional	Solvent?	Basis Set	Type of Data
mPW1PW91	PCM	6-311+G(d,p)	Unscaled Shifts

	Isomer 1	Isomer 2	Isomer 3	Isomer 4	Isomer 5	Isomer 6
sDP4+ (H data)	2.06%	97.94%	0.00%	-	-	-
sDP4+ (C data)	96.27%	3.72%	0.00%	-	-	-
sDP4+ (all data)	35.23%	64.77%	0.00%	-	-	-
uDP4+ (H data)	1.31%	98.68%	0.01%	-	-	-
uDP4+ (C data)	0.00%	100.00%	0.00%	-	-	-
uDP4+ (all data)	0.00%	100.00%	0.00%	-	-	-
DP4+ (H data)	0.03%	99.97%	0.00%	-	-	-
DP4+ (C data)	0.00%	100.00%	0.00%	-	-	-
DP4+ (all data)	0.00%	100.00%	0.00%	-	-	-

Table S3. Calculated and experimental chemical shift values used for DP4+ calculation for (**29**).

Atom	Exp.	Isomer 1	Isomer 2	Isomer 3
C	161	160.8	167.5	167.7
C	96.5	95.7	98.6	99.5
C	155.1	154.0	159.4	160.5
C	106.1	105.5	109.6	109.8
C	163.8	163.0	170.5	171.5
C	90.7	91.4	93.5	94.7
C	94.5	95.2	99.7	99.8
C	61.9	64.3	67.3	68.0
C	180.6	181.0	189.5	189.2
C	152.8	151.4	158.6	159.8
C	106.1	105.1	111.7	110.1
C	91.1	91.4	95.3	95.4
C	157.6	157.4	163.4	164.6
C	105.6	105.0	111.4	107.7
C	153.2	154.0	160.4	157.3
C	128.1	128.1	133.7	134.2
C	128.4	129.2	134.1	134.5
C	115.5	114.2	120.1	120.5
C	158.3	157.5	164.8	165.0
C	115.5	114.5	119.5	119.7
C	128.4	128.2	134.1	134.8
C	30.5	29.8	31.7	26.2
C	67.7	67.4	72.2	71.7
C	82.7	80.0	86.7	85.6
C	130.1	130.1	134.5	136.8
C	130	129.2	138.2	132.2
C	115.7	115.4	120.2	119.8

C	158.3	157.2	165.0	163.3
C	115.7	114.6	120.8	119.6
C	130	129.4	136.6	132.0
H	5.78	6.01	5.71	5.97
H	5.66	6.08	5.71	6.22
H	5.72	5.99	6.14	6.20
H	6.05	6.24	6.32	6.30
H	7.07	7.43	7.80	7.33
H	6.6	6.69	7.15	7.06
H	6.6	6.75	7.09	7.04
H	7.07	6.95	7.98	8.05
H	2.39	2.71	2.33	2.03
H	2.93	2.61	2.96	2.58
H	3.85	3.74	3.79	4.14
H	4.19	4.76	3.92	5.39
H	7.2	7.18	7.57	7.09
H	6.83	7.03	7.19	6.98
H	6.83	6.90	7.29	6.94
H	7.2	6.80	8.15	6.97

Figure S36. HRESIMS (Q-TOF) analysis of yuccalechin C (**29**) in negative ion mode.

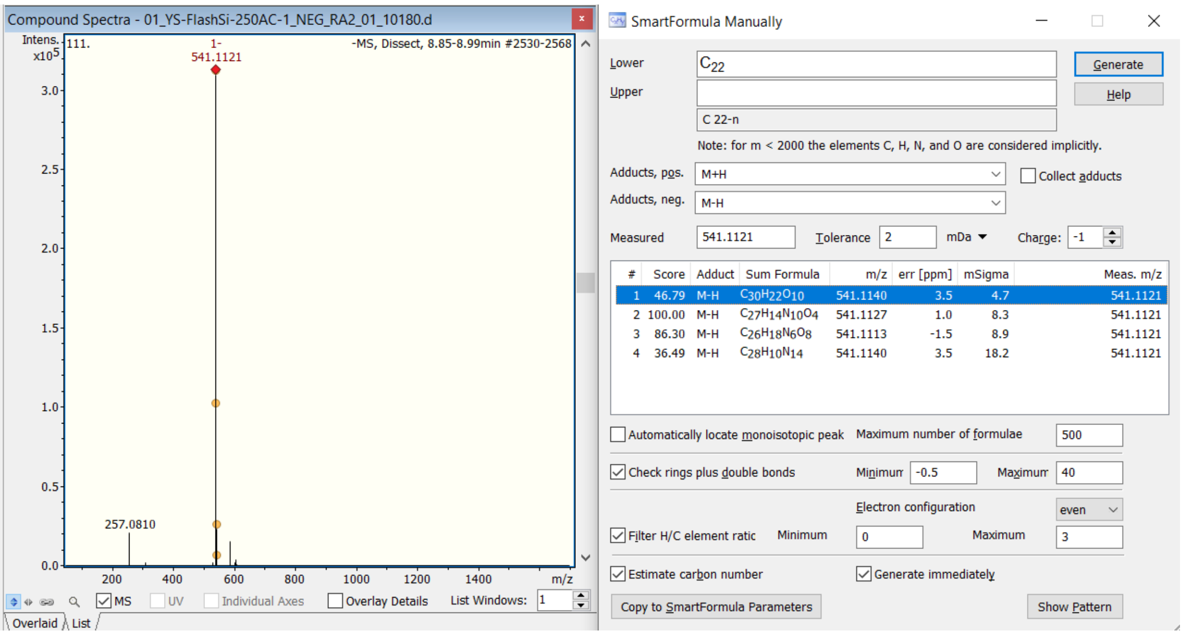

Figure S37. ^{1}H NMR spectrum of yuccaol E (**37**) (500 MHz, MeOH-d_4, 30 °C).

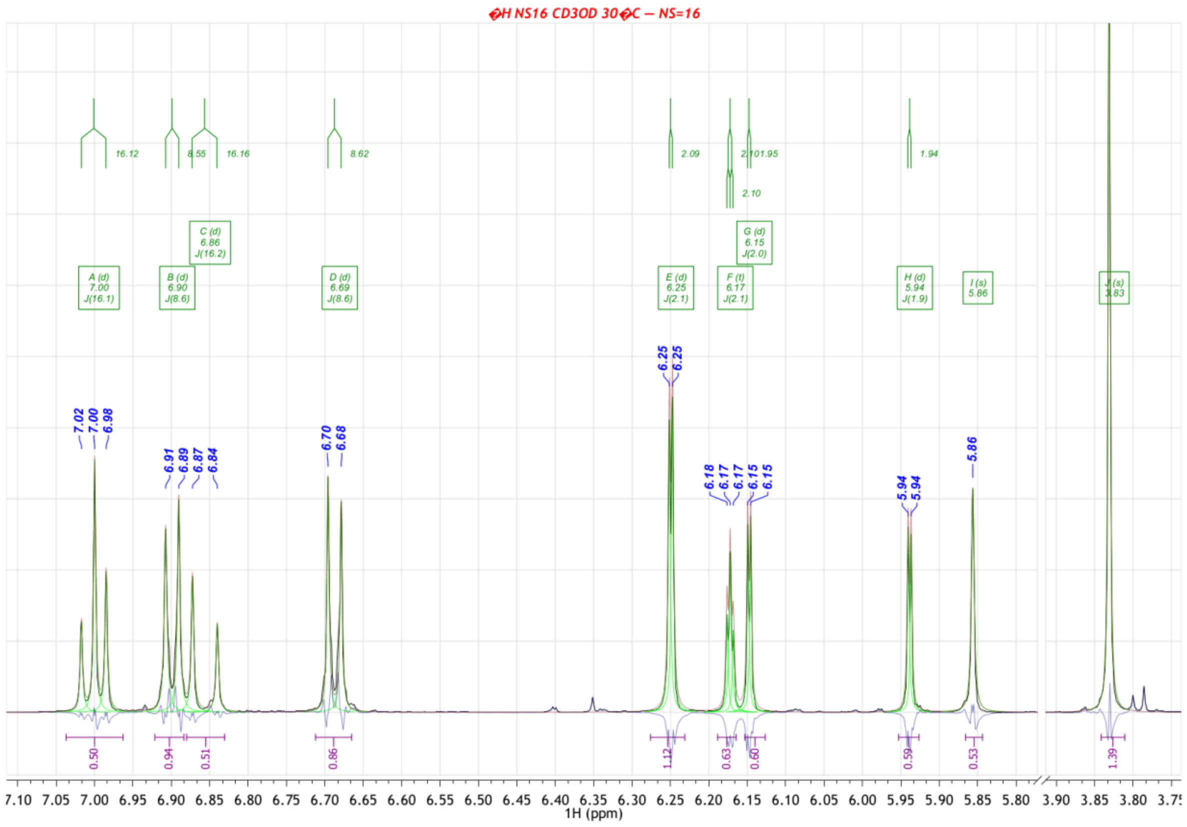

Figure S38. ^{13}C NMR spectrum of yuccaol E (**37**) (125 MHz, MeOH-d_4, 30 °C).

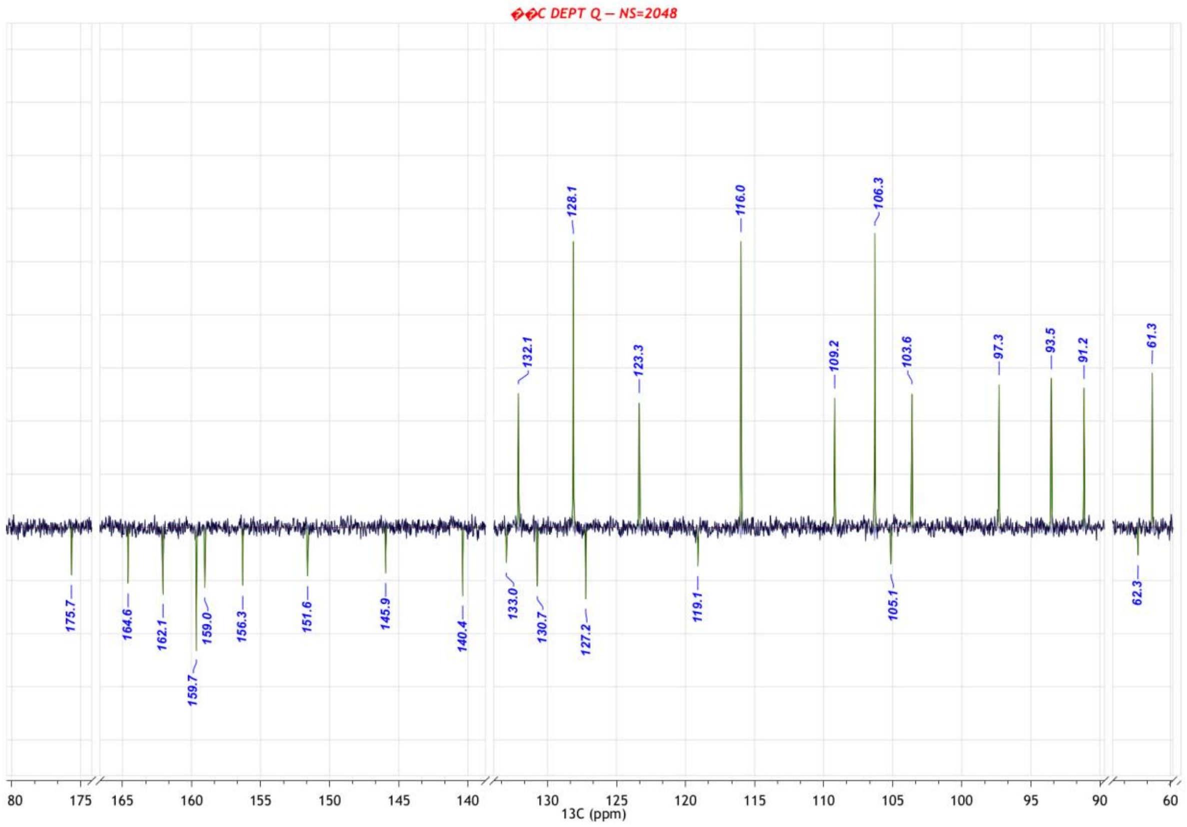

Figure S39. ^{1}H NMR spectrum of naringenin (**38**) (500 MHz, MeOH-d_4, 30 °C).

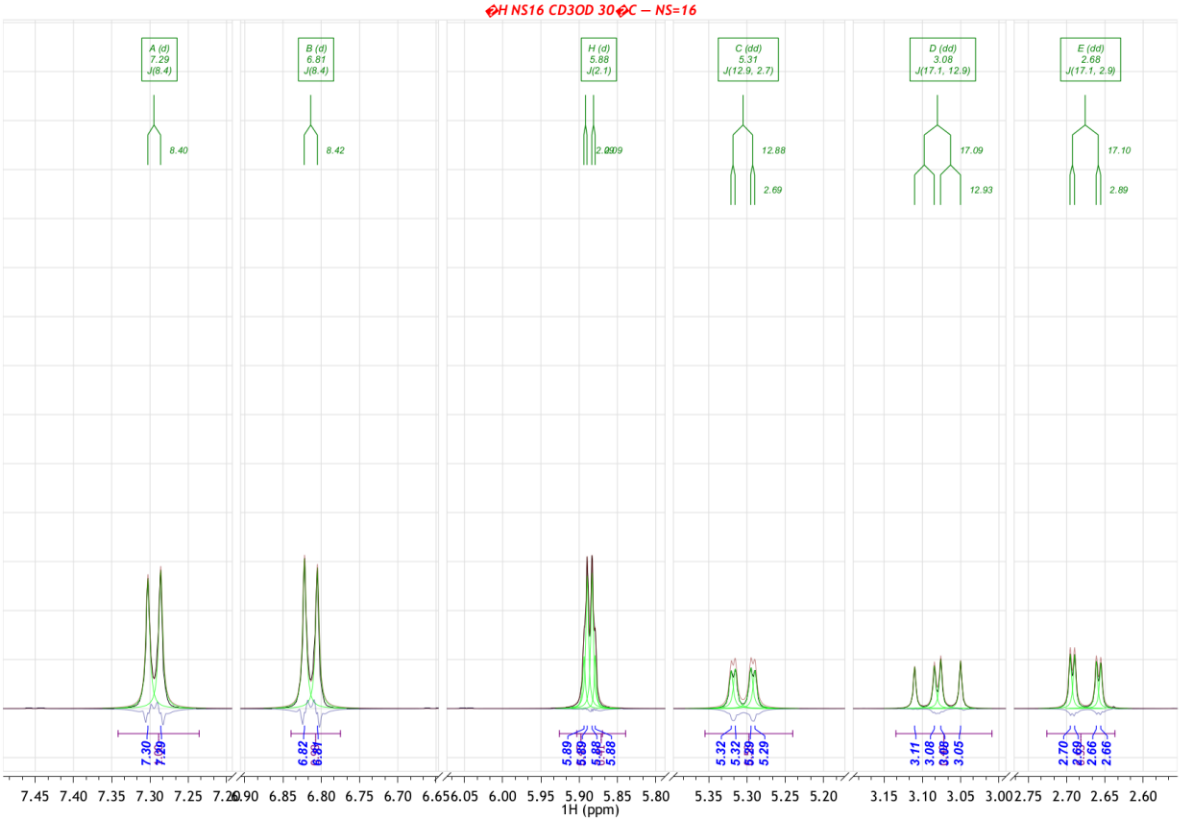

Figure S40. ^{13}C NMR spectrum of naringenin (**38**) (125 MHz, MeOH-d_4, 30 °C).

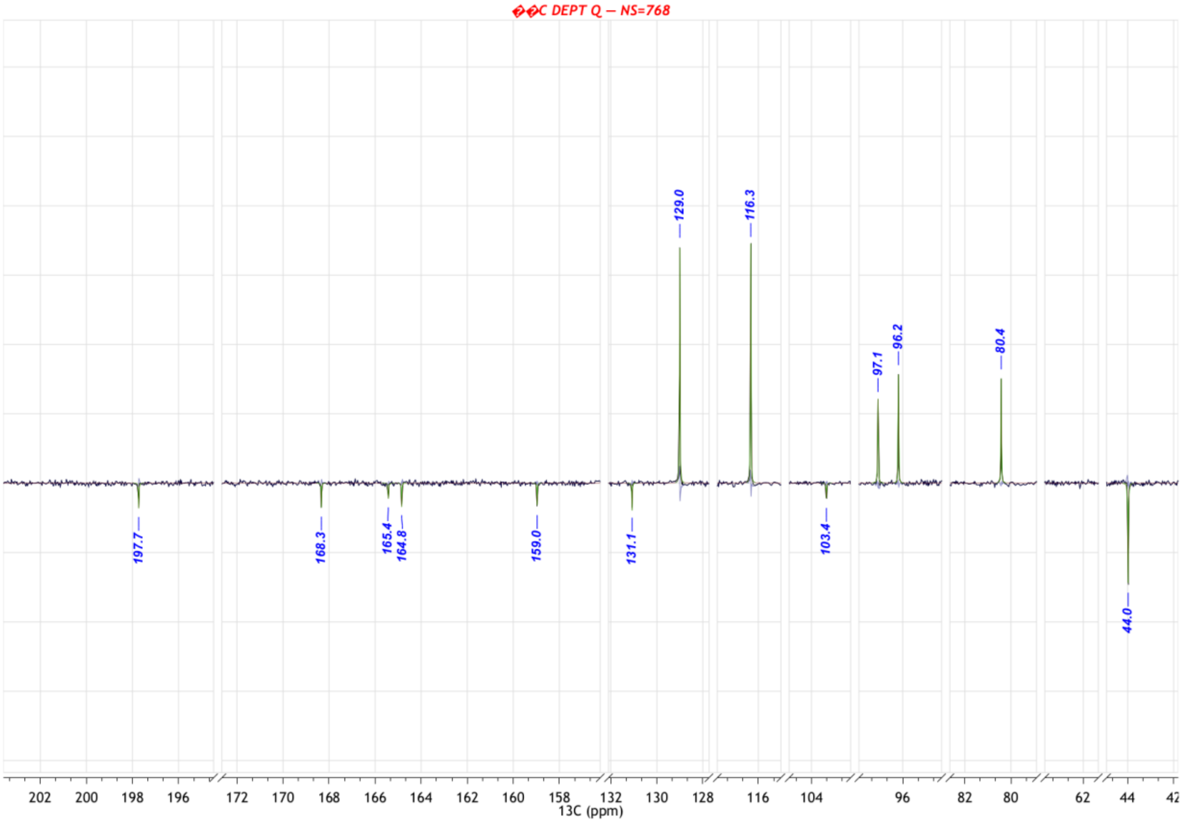

Figure S41. ^{1}H NMR spectrum of yuccaol C (**39**) (500 MHz, MeOH-d_4, 30 °C).

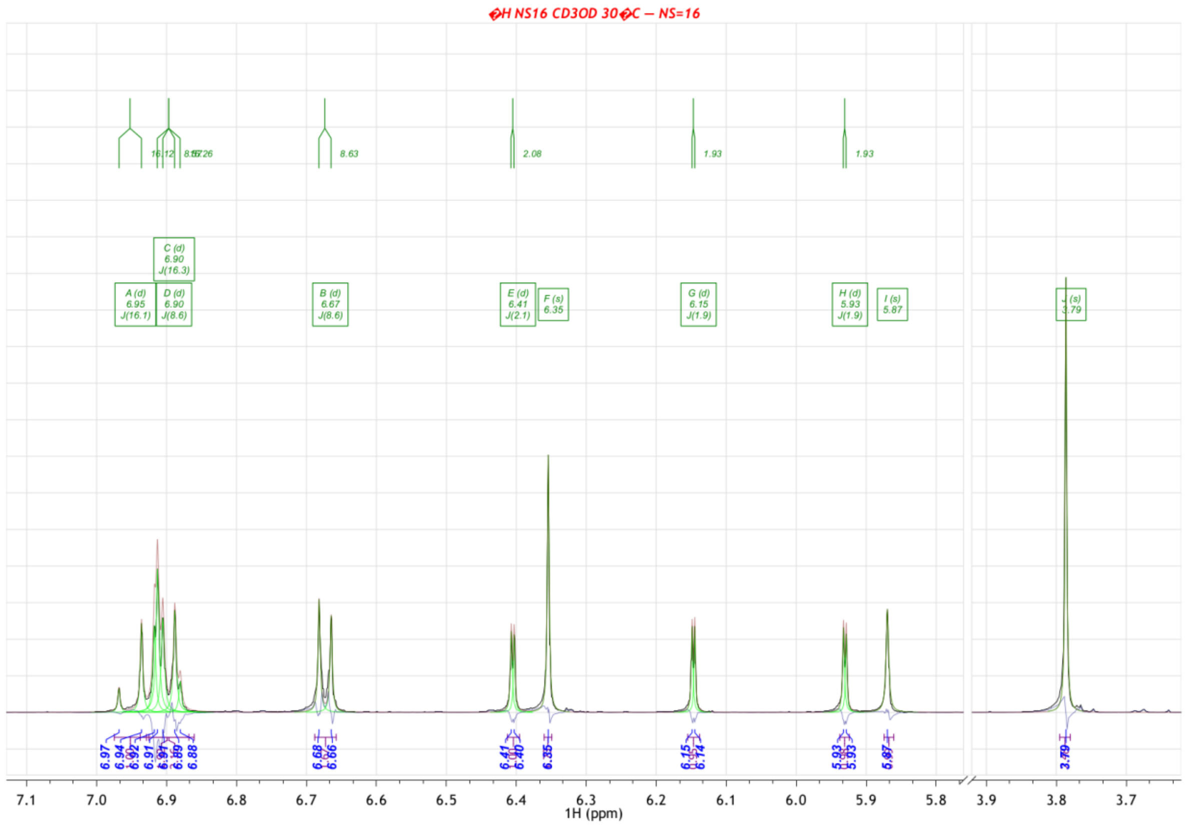

Figure S42. ^{13}C NMR spectrum of yuccaol C (**39**) (125 MHz, MeOH-d_4, 30 °C).

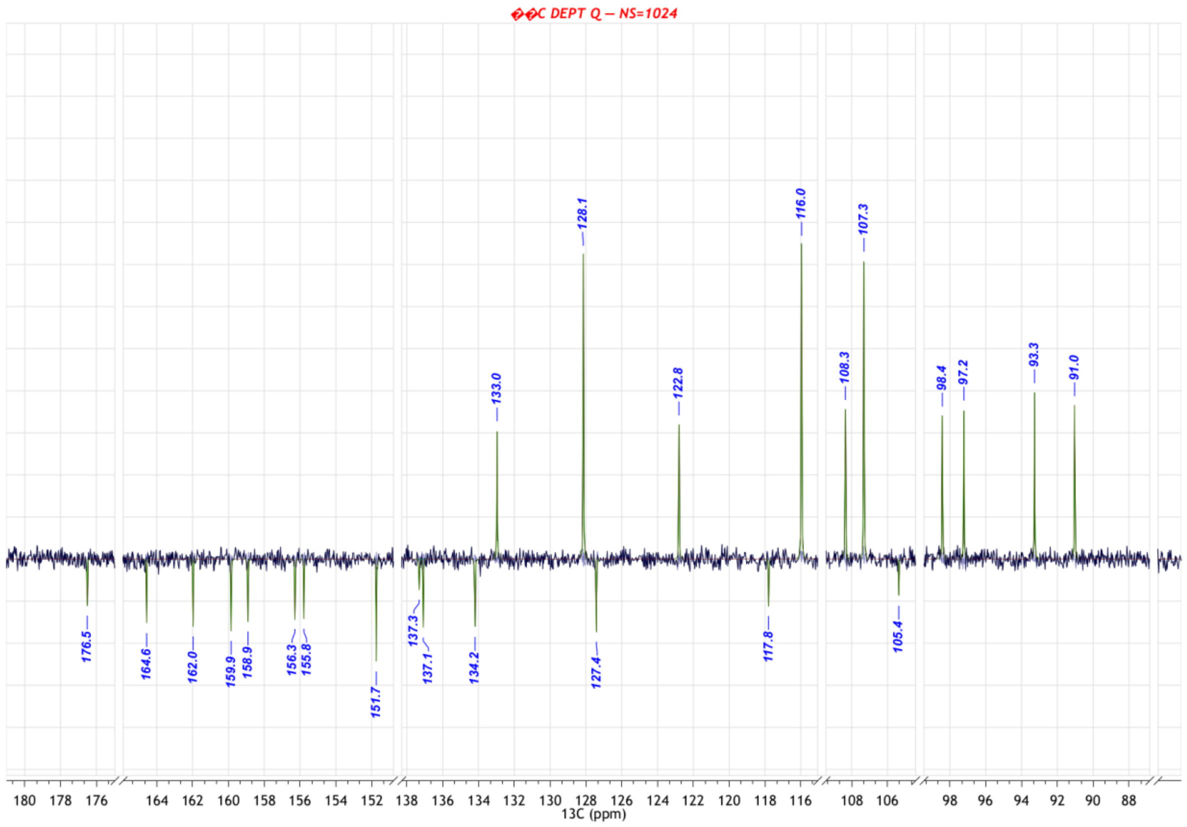

Figure S43. ^{1}H NMR spectrum of yuccalide A (**40**) (500 MHz, MeOH-d_4, 30 °C).

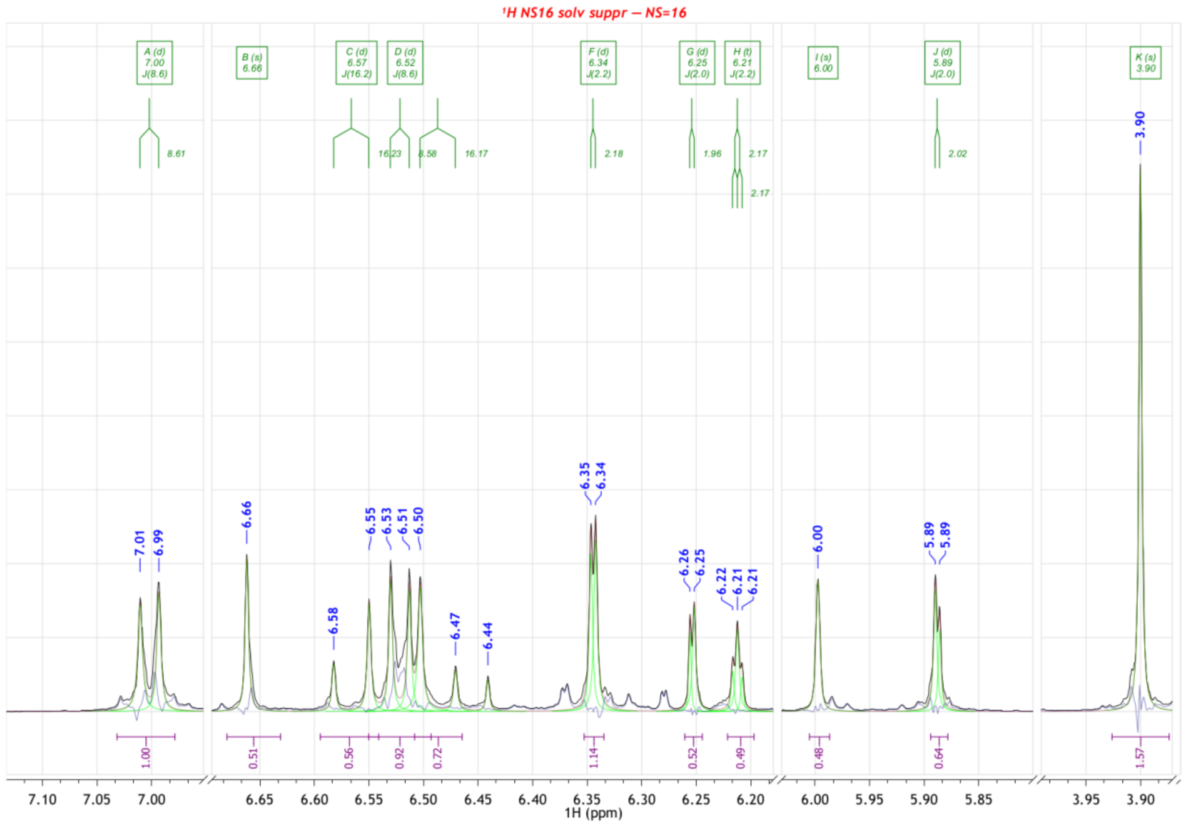

Figure S44. ^{13}C NMR spectrum of yuccalide A (**40**) (125 MHz, MeOH-d_4, 30 °C).

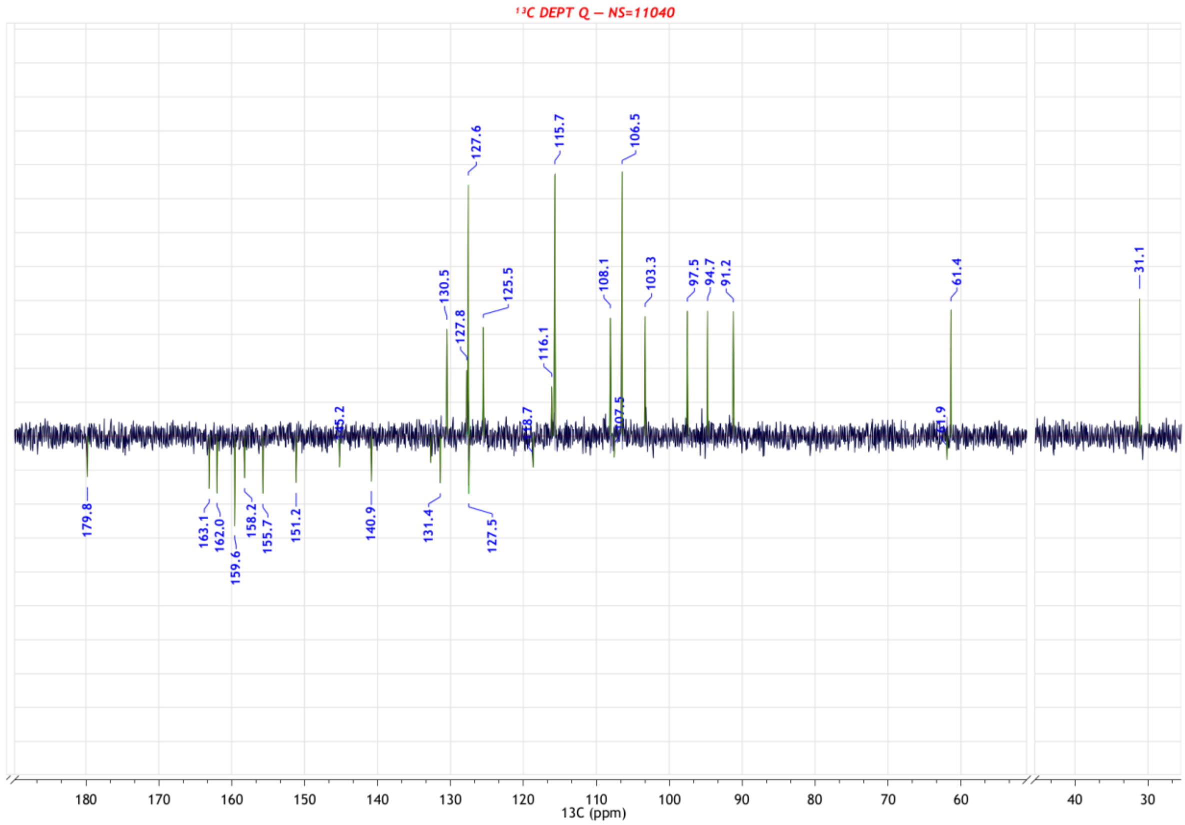

Figure S45. ^{1}H NMR spectrum of yuccaol D (**42**) (500 MHz, MeOH-d_4, 30 °C).

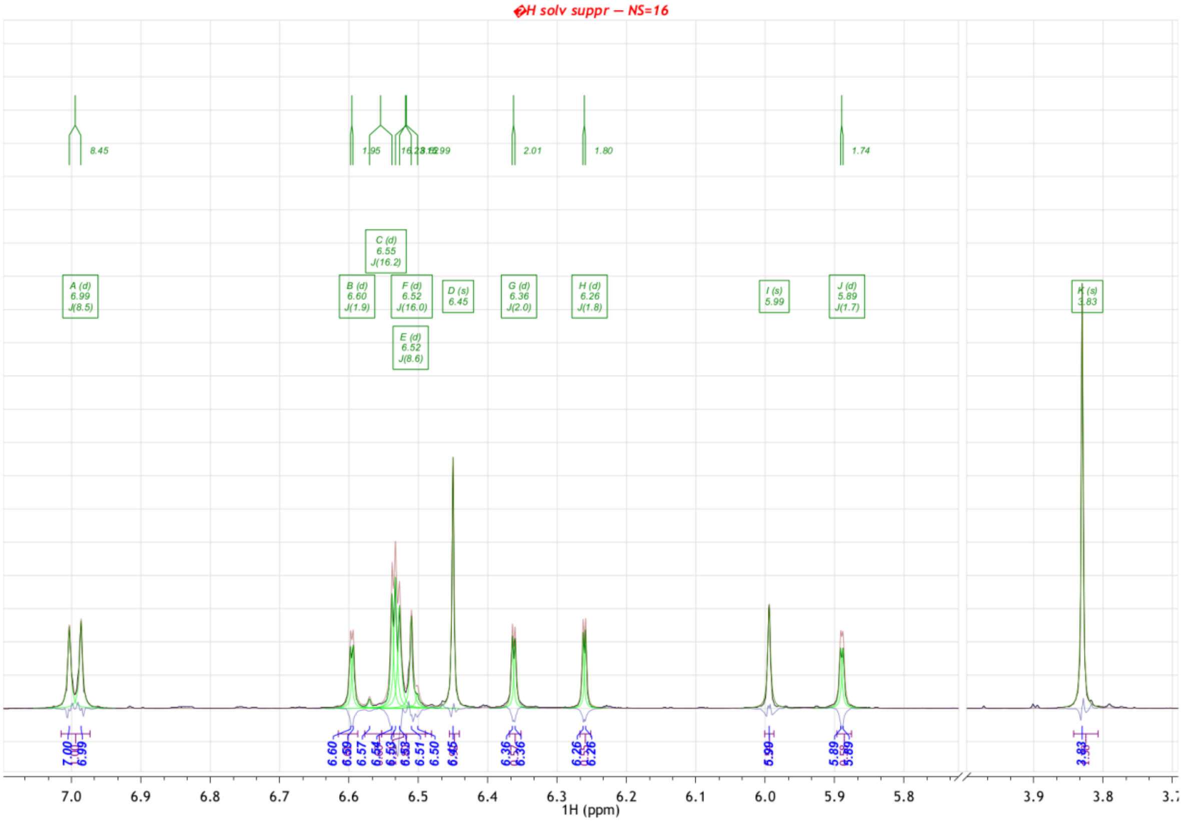

Figure S46. ^{13}C NMR spectrum of yuccaol D (**42**) (125 MHz, MeOH-d_4, 30 °C).

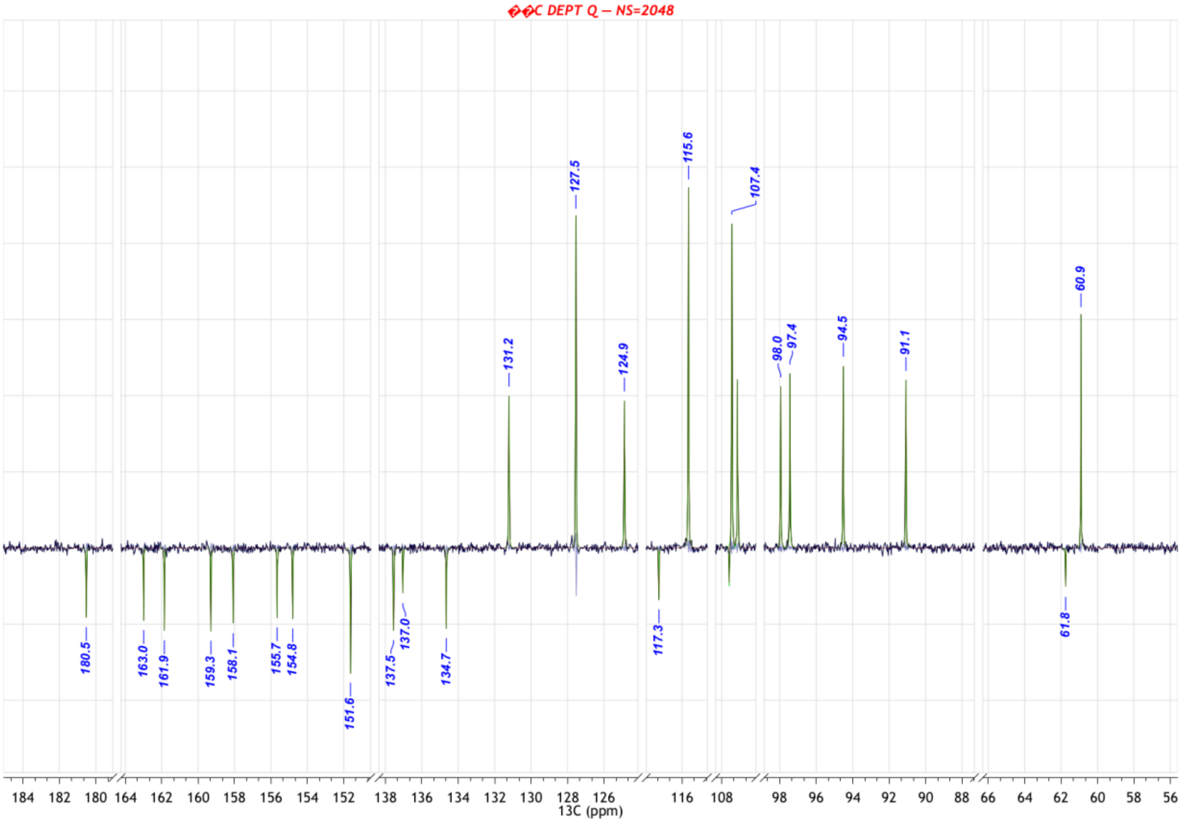

Figure S47. ^{1}H NMR spectrum of kaempferol (**44**) (500 MHz, MeOH-d_4, 30 °C).

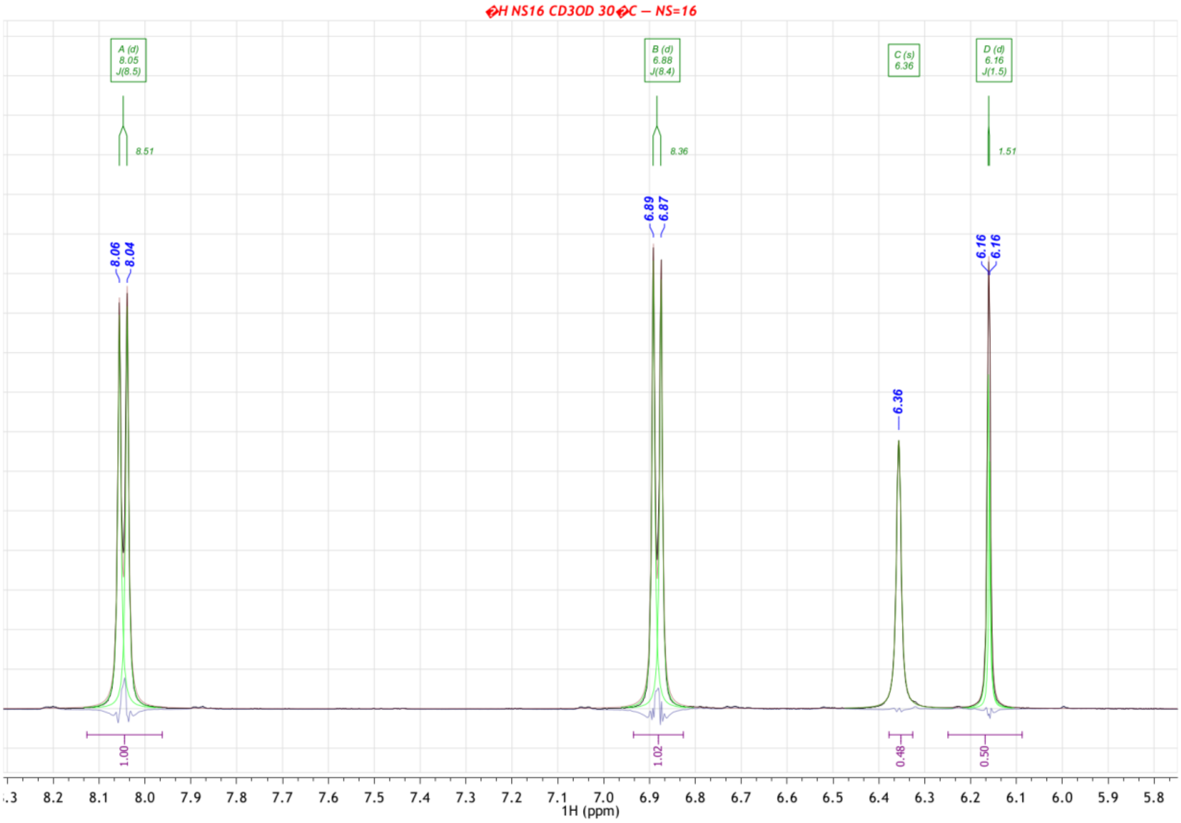

Figure S48. ^{13}C NMR spectrum of kaempferol (**44**) (125 MHz, MeOH-d_4, 30 °C).

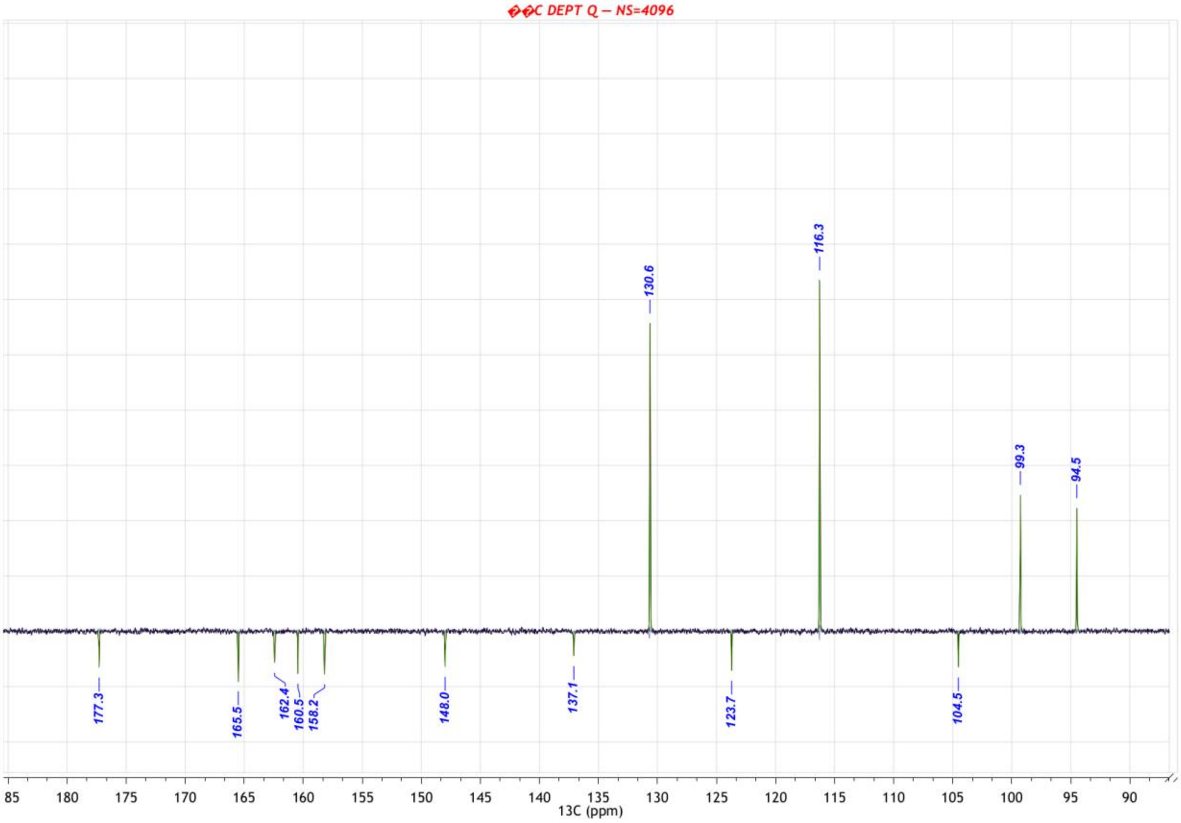

Figure S49. ^{1}H NMR spectrum of yuccaol A (**47**) (500 MHz, MeOH-d_4, 30 °C).

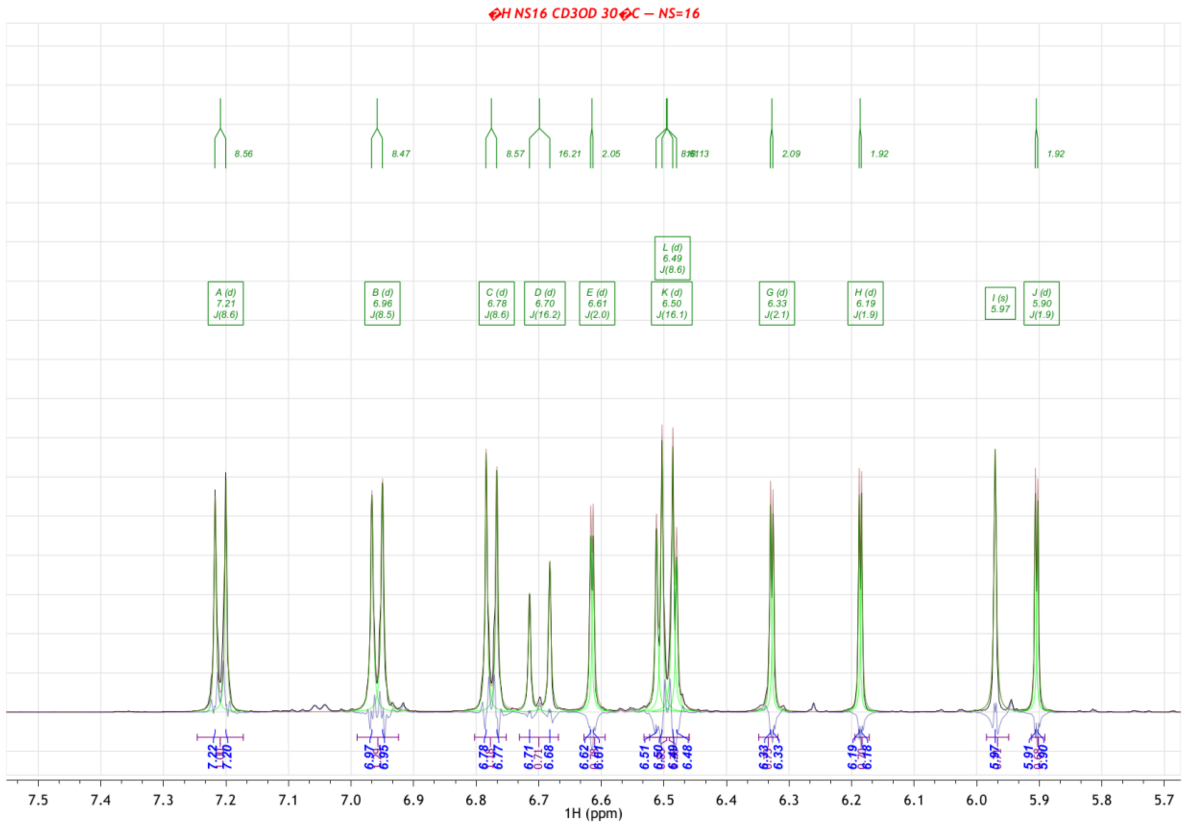

Figure S50. ^{13}C NMR spectrum of yuccaol A (**47**) (125 MHz, MeOH-d_4, 30 °C).

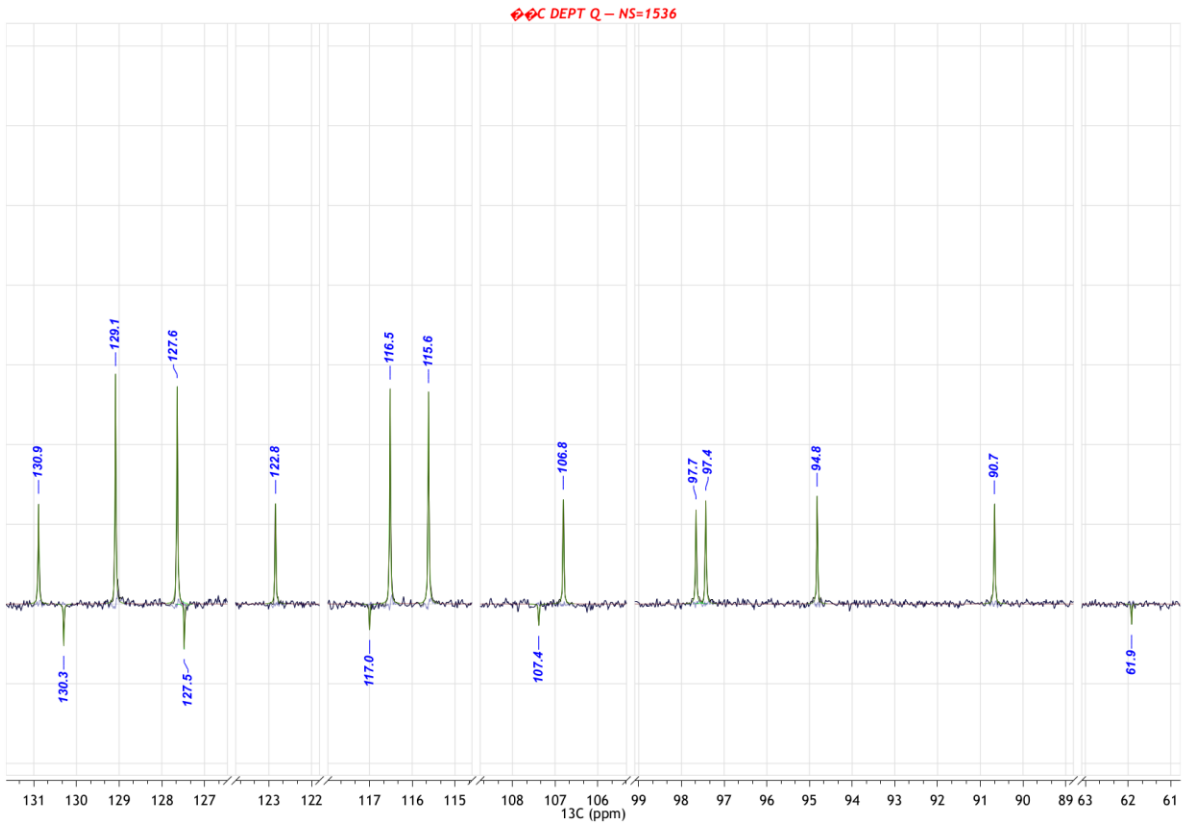

Figure S51. ^{1}H NMR spectrum of yuccaol B (**48**) (500 MHz, MeOH-d_4, 30 °C).

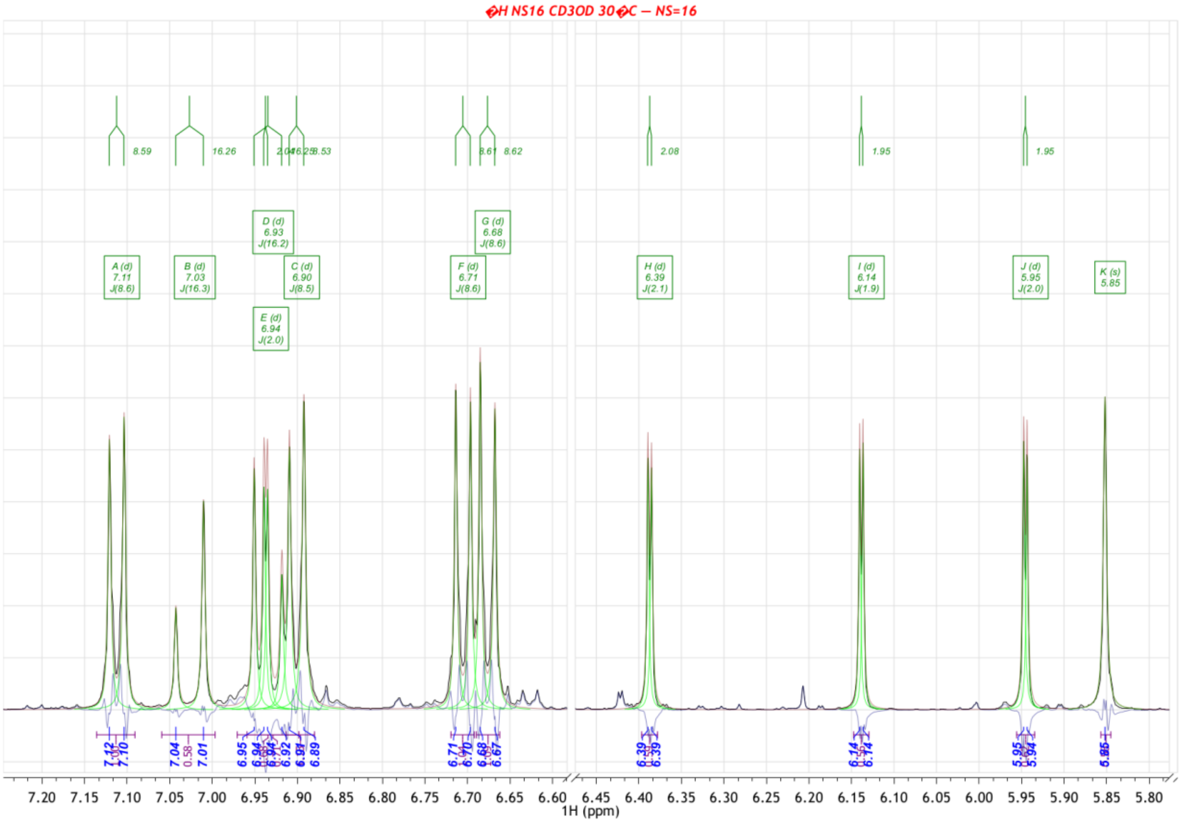

Figure S52. ^{13}C NMR spectrum of yuccaol B (**48**) (125 MHz, MeOH-d_4, 30 °C).

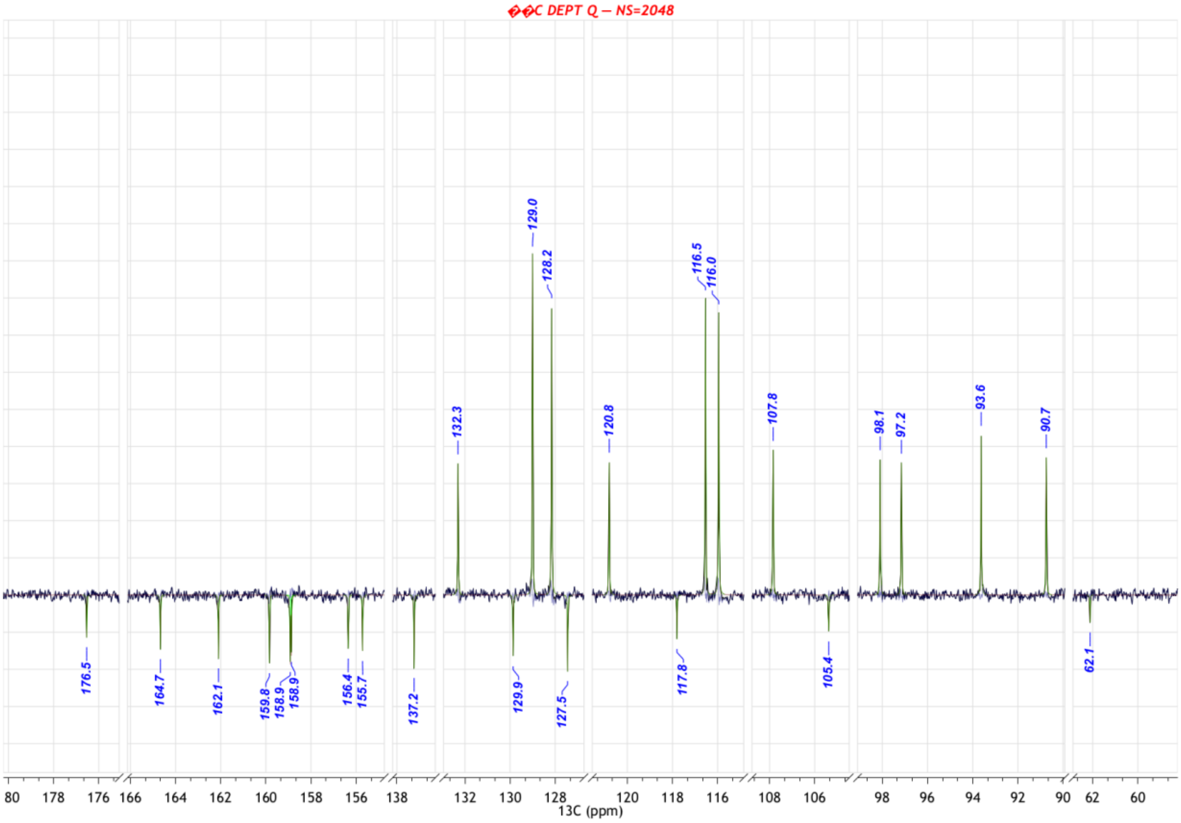

Figure S53. ^{1}H NMR spectrum of gloriosaol E (**49**) (500 MHz, MeOH-d_4, 30 °C).

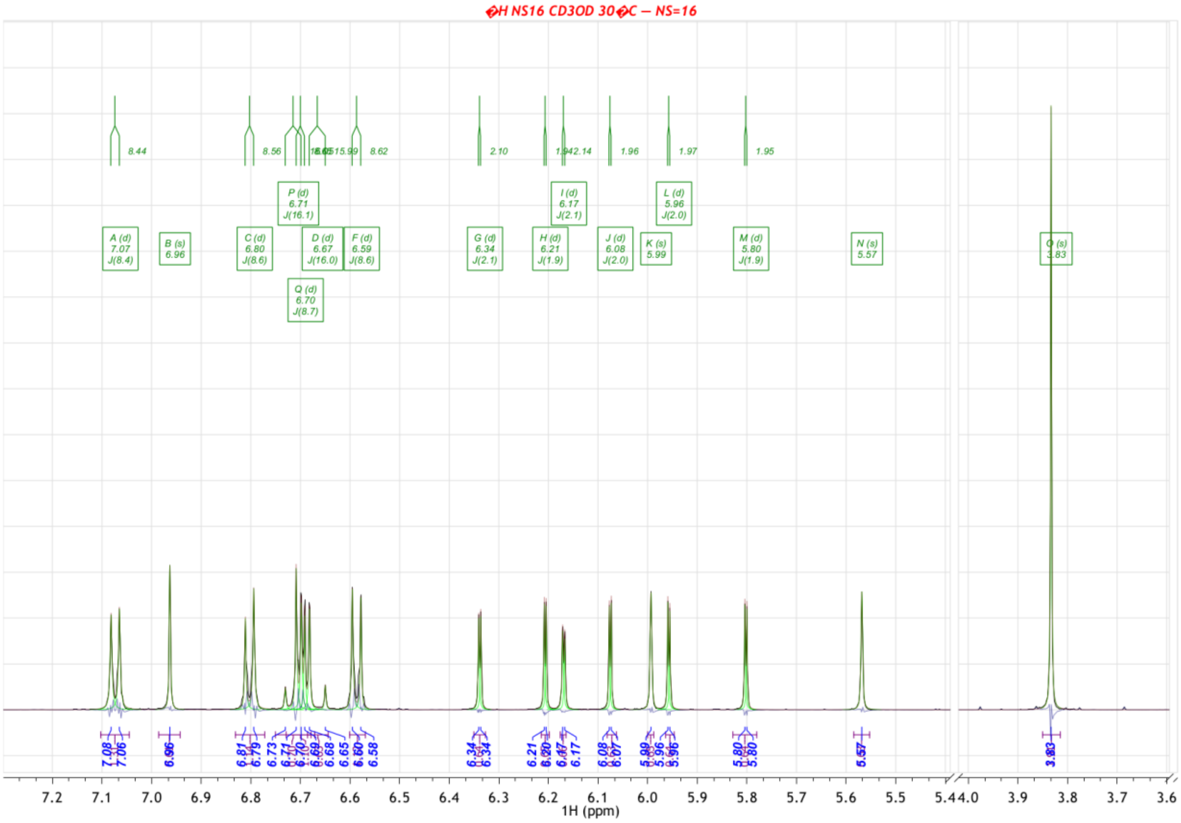

Figure S54. ^{13}C NMR spectrum of gloriosaol E (**49**) (125 MHz, MeOH-d_4, 30 °C).

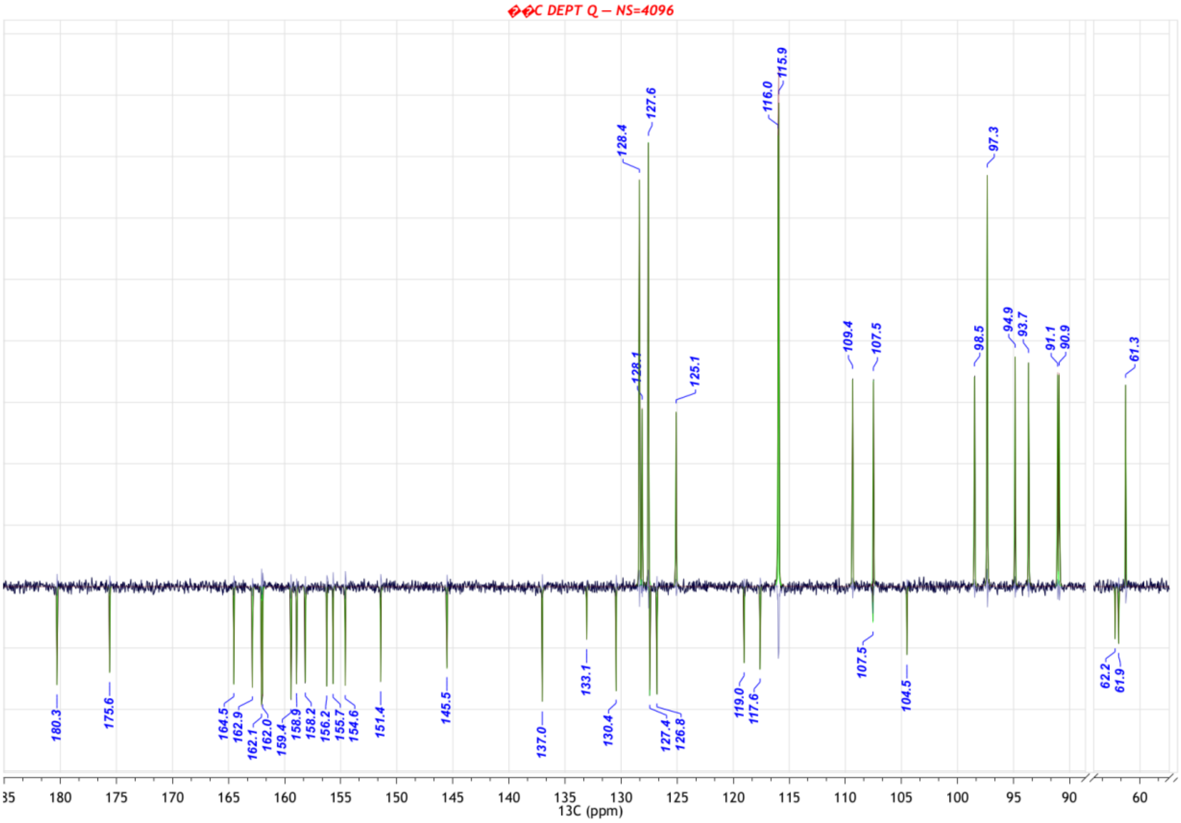

Figure S55. ^{1}H NMR spectrum of gloriosaol D (**50**) (500 MHz, MeOH-d_4, 30 °C).

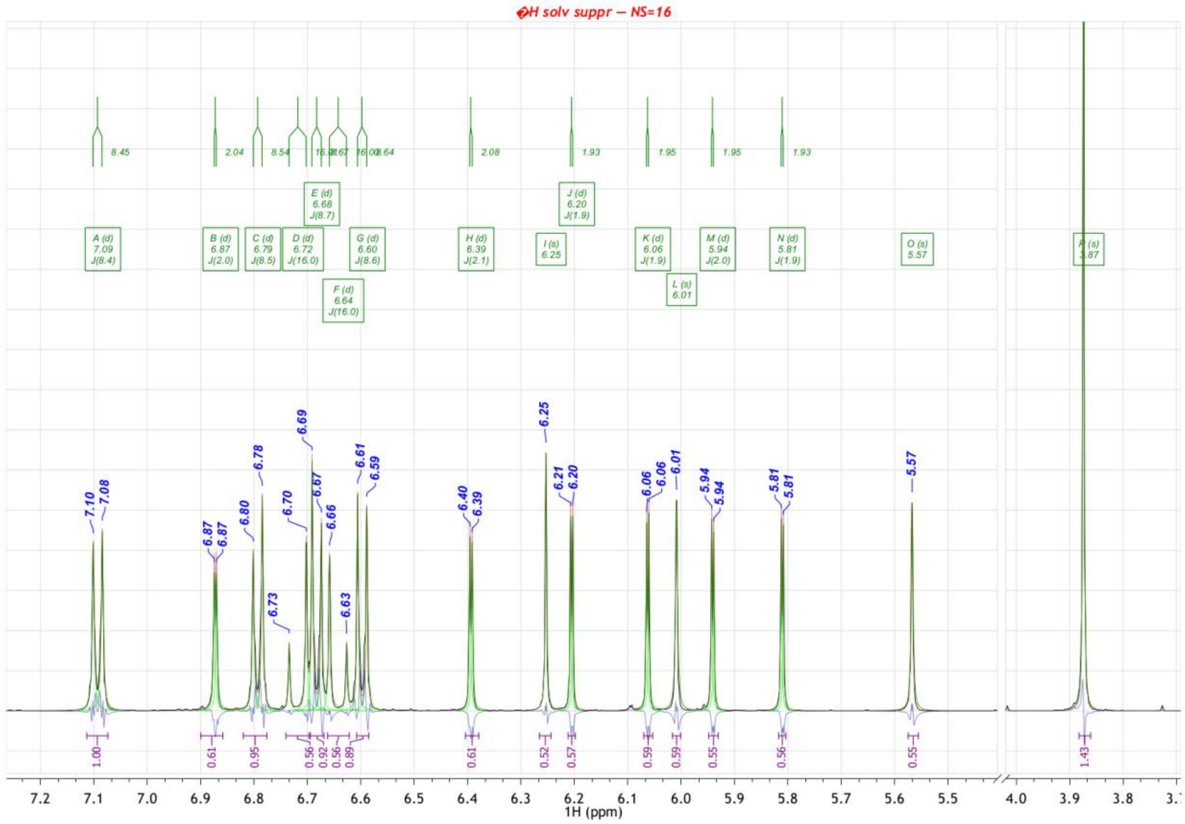

Figure S56. ^{13}C NMR spectrum of gloriosaol D (**50**) (125 MHz, MeOH-d_4, 30 °C).

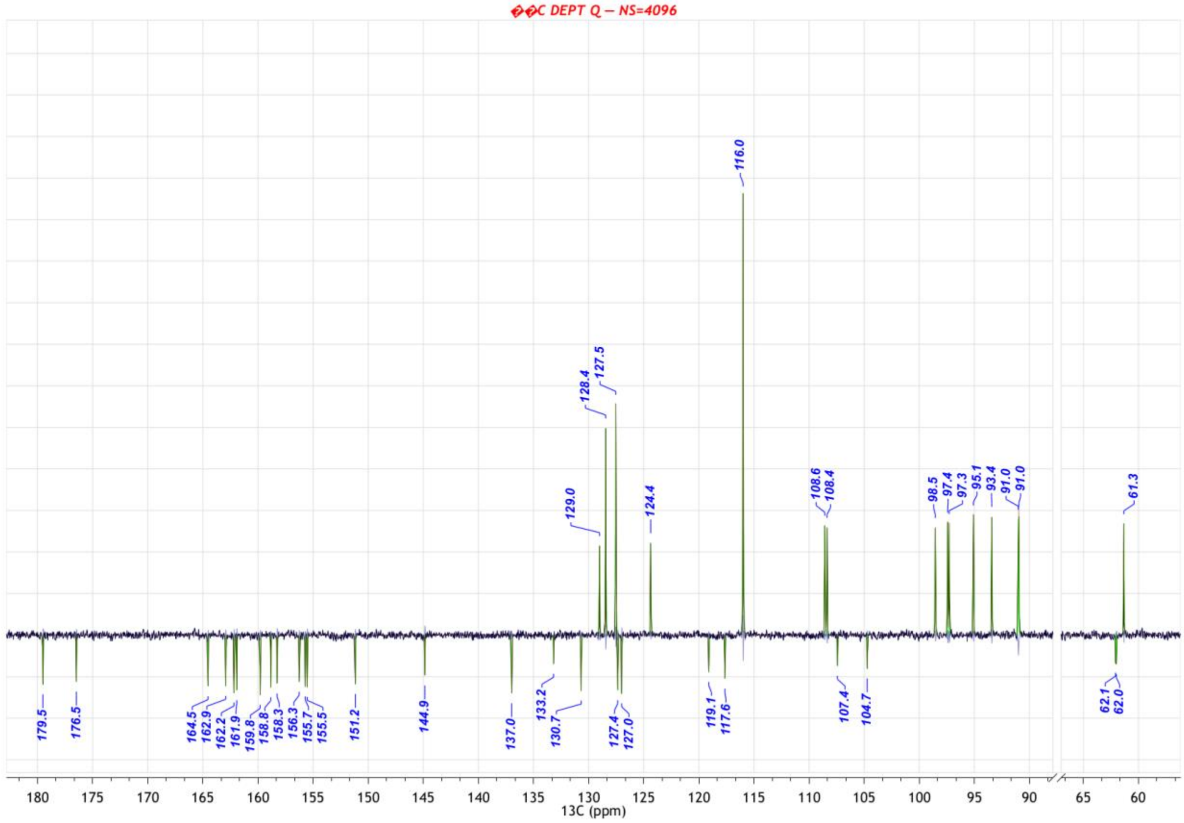

Figure S57. ^{1}H NMR spectrum of gloriosaol A (**54**) (500 MHz, MeOH-d_4, 30 °C).

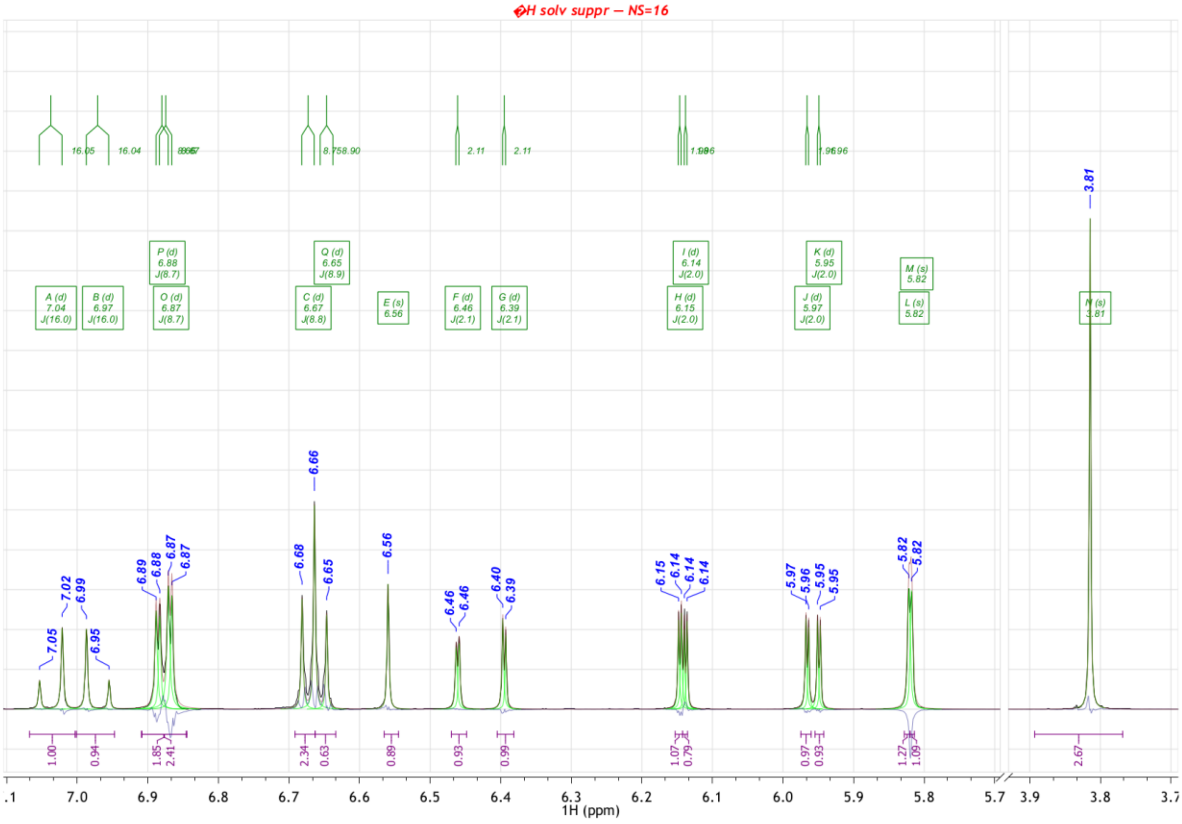

Figure S58. ^{13}C NMR spectrum of gloriosaol A (**54**) (125 MHz, MeOH-d_4, 30 °C).

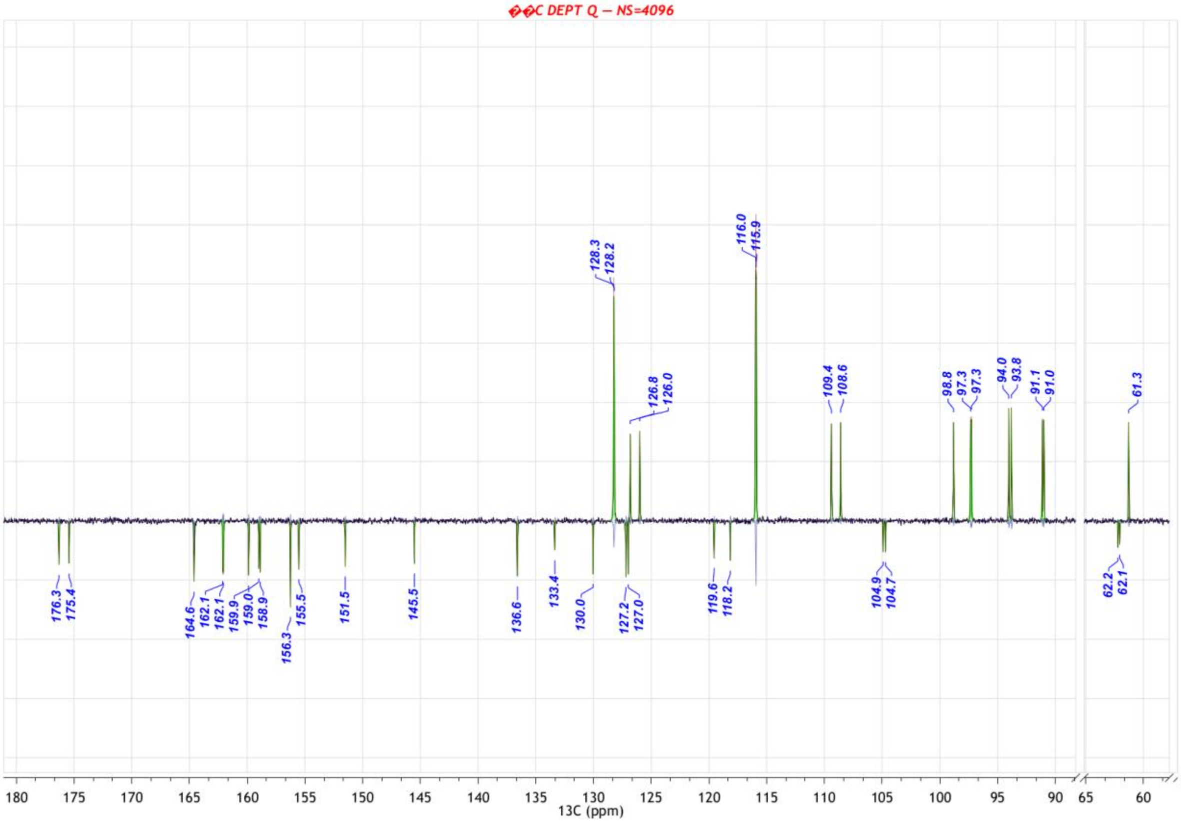

Figure S59. ^{1}H NMR spectrum of gloriosaol C (**58**) (500 MHz, MeOH-d_4, 30 °C).

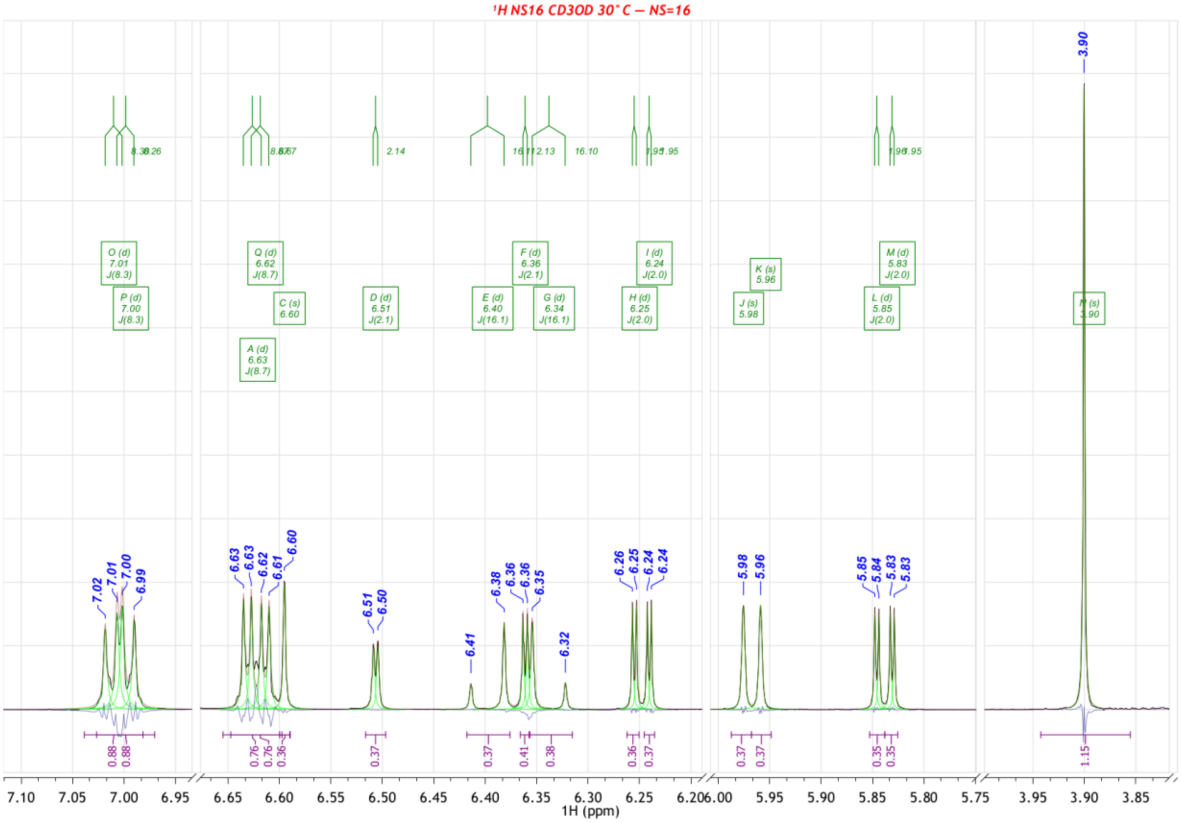

Figure S60. ^{13}C NMR spectrum of gloriosaol C (**58**) (125 MHz, MeOH-d_4, 30 °C).

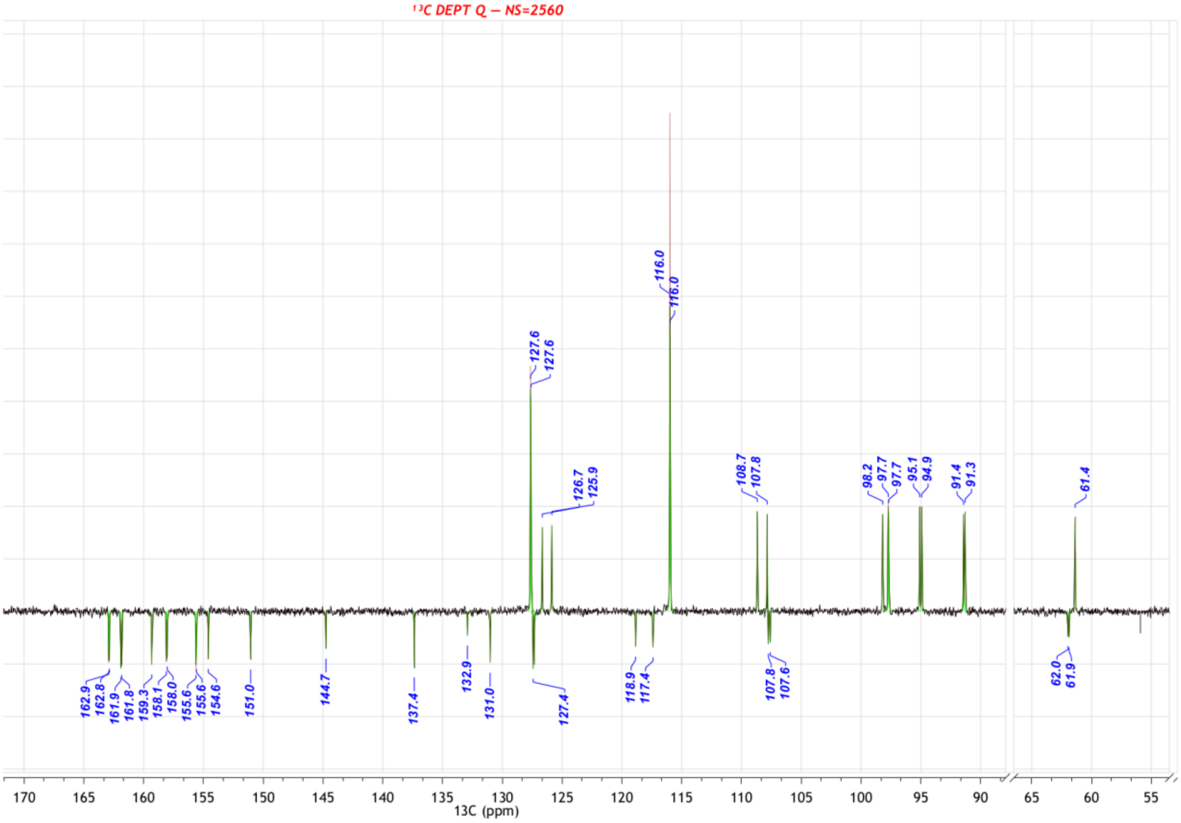

Supplement: Supplementary file 1 [file molecules-24-04162-s001.zip › Supplementary Materials_LPecio_MAlilou_ready.pdf]
