# Supplementary material for: Yuccalechins A–C from the Yucca schidigera Roezl ex Ortgies Bark: Elucidation of the Relative and Absolute Configurations of Three New Spirobiflavonoids and Their Cholinesterase Inhibitory Activities
Source: Molecules. 2019 Nov 16;24(22):4162. doi: 10.3390/molecules24224162 (PMC6891570; doi:10.3390/molecules24224162)

Supplementary Materials

Yuccalechins A-C from the *Yucca schidigera* Roezl ex Ortgies bark: elucidation of relative and absolute configurations of three new spirobiflavonoids and their cholinesterase inhibitory activity.

Łukasz Pecio ^1†,*^, Mostafa Alilou ^2†,*^, Solomiia Kozachok ^1^, Ilkay Erdogan Orhan ^3^, Gokcen Eren ^4^, Fatma Sezer Senol Deniz ^3^, Hermann Stuppner ^2^, and Wiesław Oleszek ^1^

^1^ Department of Biochemistry and Crop Quality, Institute of Soil Science and Plant Cultivation—State Research Institute, Czartoryskich 8, 24-100 Puławy, Poland; [lpecio@iung.pulawy.pl](mailto:lpecio@iung.pulawy.pl) (Ł.P.); [skozachok@iung.pulawy.pl](mailto:skozachok@iung.pulawy.pl) (S.K.); [wieslaw.oleszek@iung.pulawy.pl](mailto:wieslaw.oleszek@iung.pulawy.pl) (W.O.)

^2^ Institute of Pharmacy/Pharmacognosy, Center for Molecular Biosciences Innsbruck, University of Innsbruck, Innrain 80/82, Innsbruck 6020, Austria; mostafa.alilou@student.uibk.ac.at (M.A.); [hermann.stuppner@uibk.ac.at](mailto:hermann.stuppner@uibk.ac.at) (H.S.)

^3^ Department of Pharmacognosy, Faculty of Pharmacy, Gazi University, 06330 Ankara, Turkey; [iorhan@gazi.edu.tr](mailto:iorhan@gazi.edu.tr) (I.E.O.); [fssenol@gazi.edu.tr](mailto:fssenol@gazi.edu.tr) (F.S.S.D.)

^4^ Department of Pharmaceutical Chemistry, Faculty of Pharmacy, Gazi University, 06330 Ankara, Turkey; [gokcene@gazi.edu.tr](mailto:gokcene@gazi.edu.tr) (G.E.)

***** Correspondence: [lpecio@iung.pulawy.pl](mailto:lpecio@iung.pulawy.pl) (Ł.P.); mostafa.alilou@student.uibk.ac.at (M.A.)

^†^ These authors contributed equally to this work.

Received: date; Accepted: date; Published: date

**Abstract:** The ethyl acetate fraction of the methanolic extract of *Yucca schidigera* Roezl ex Ortgies bark exhibited moderate acetylcholinesterase (AChE) and butyrylcholinesterase (BChE) inhibitory activity (IC_50_ 47.44 µg·mL^-1^ and 47.40 µg·mL^-1^, respectively). Gel filtration on Sephadex LH-20 and further RP-C_18_ preparative HPLC of EtOAc fraction afforded 15 known and three new compounds, stereoisomers of larixinol. The structures of the isolated spirobiflavonoids **15**, **26** and **29** were elucidated using 1D and 2D NMR and MS spectroscopic techniques. The relative configuration of isolated compounds was assigned based on coupling constants and ROESY correlations along with applying DP4+ probability method in case of ambiguous chiral centers. Determination of absolute configuration was done by comparison of calculated ECD spectra with experimental ones. Compounds **26** and **29**, obtained in sufficient amounts, were evaluated for activities against AChE and BChE, and showed a weak inhibition only towards AChE (IC_50_ 294.18 µM for **26**, and 655.18 µM for **29**). Furthermore, molecular docking simulations were performed to investigate the possible binding modes of **26** and **29** with AChE.

**Keywords:** *Yucca schidigera*; Asparagaceae; spirobiflavonoid; absolute configuration; DP4+; ECD; Alzheimer’s disease

List of Figures

[Figure S1. ^1^H NMR spectrum of *trans*-3,3′,5,5′-tetrahydroxy-4′-methoxystilbene (**13**) (500 MHz, MeOH-*d_4_*, 30 °C). 4](#_Toc22646600)

[Figure S2. ^13^C NMR spectrum of *trans*-3,3′,5,5′-tetrahydroxy-4′-methoxystilbene (**13**) (125 MHz, MeOH-*d_4_*, 30 °C). 4](#_Toc22646601)

[Figure S3. ^1^H NMR spectrum of yuccalechin A (**15**) (500 MHz, MeOH-*d_4_*, 30 °C). 5](#_Toc22646602)

[Figure S4. ^13^C NMR spectrum of yuccalechin A (**15**) (125 MHz, MeOH-*d_4_*, 30 °C). 5](#_Toc22646603)

[Figure S5. ^1^H-^1^H COSY NMR spectrum of yuccalechin A (**15**) (500 MHz, MeOH-*d_4_*, 30 °C). 6](#_Toc22646604)

[Figure S6. ^1^H-^1^H ROESY (250 ms) NMR spectrum of yuccalechin A (**15**) (500 MHz, MeOH-*d_4_*, 30 °C). 6](#_Toc22646605)

[Figure S7. ^1^H-^13^C HSQC NMR spectrum of yuccalechin A (**15**) (500/125 MHz, MeOH-*d_4_*, 30 °C). 7](#_Toc22646606)

[Figure S8. ^1^H-^13^C H2BC NMR spectrum of yuccalechin A (**15**) (500/125 MHz, MeOH-*d_4_*, 30 °C). 7](#_Toc22646607)

[Figure S9. ^1^H-^13^C HMBC (8 Hz) NMR spectrum of yuccalechin A (**15**) (500/125 MHz, MeOH-*d_4_*, 30 °C). 8](#_Toc22646608)

[Figure S10. Optimized conformers of yuccalechin A (**15**) in DFT/B3LYP/6-31G(d,p)/IEFPCM/methanol level of theory. 8](#_Toc22646609)

[Figure S11. Calculated DP4+ probabilities of yuccalechin A (**15**) using mpw1pw91/6-111G+(d,p)/ IEFPCM/methanol level of theory. Isomer 1 is 2”*R*,3”*S*,2*R*,3*R* and isomer 2 is 2”*R*,3”S,2*S*,3*R*. 9](#_Toc22646610)

[Table S1. Calculated and experimental chemical shift values used for DP4+ calculation for (**15**). 9](#_Toc22646611)

[Figure S12. HRESIMS (Q-TOF) analysis of yuccalechin A (**15**) in negative ion mode. 10](#_Toc22646612)

[Figure S13. ^1^H NMR spectrum of aromadendrin (**16**) (500 MHz, MeOH-*d_4_*, 30 °C). 11](#_Toc22646613)

[Figure S14. ^13^C NMR spectrum of aromadendrin (**16**) (125 MHz, MeOH-*d_4_*, 30 °C). 11](#_Toc22646614)

[Figure S15. ^1^H NMR spectrum of *trans*-resveratrol (**21**) (500 MHz, MeOH-*d_4_*, 30 °C). 12](#_Toc22646615)

[Figure S16. ^13^C NMR spectrum of *trans*-resveratrol (**21**) (125 MHz, MeOH-*d_4_*, 30 °C). 12](#_Toc22646616)

[Figure S17. ^1^H NMR spectrum of yuccalechin B (**26**) (500 MHz, MeOH-*d_4_*, 30 °C). 13](#_Toc22646617)

[Figure S18. ^13^C NMR spectrum of yuccalechin B (**26**) (125 MHz, MeOH-*d_4_*, 30 °C). 13](#_Toc22646618)

[Figure S19. ^1^H-^1^H COSY NMR spectrum of yuccalechin B (**26**) (500 MHz, MeOH-*d_4_*, 30 °C). 14](#_Toc22646619)

[Figure S20. ^1^H-^1^H ROESY (250 ms) NMR spectrum of yuccalechin B (**26**) (500 MHz, MeOH-*d_4_*, 30 °C). 14](#_Toc22646620)

[Figure S21. ^1^H-^13^C HSQC NMR spectrum of yuccalechin B (**26**) (500/125 MHz, MeOH-*d_4_*, 30 °C). 15](#_Toc22646621)

[Figure S22. ^1^H-^13^C H2BC NMR spectrum of yuccalechin B (**26**) (500/125 MHz, MeOH-*d_4_*, 30 °C). 15](#_Toc22646622)

[Figure S23. ^1^H-^13^C HMBC (8Hz) NMR spectrum of yuccalechin B (**26**) (500/125 MHz, MeOH-*d_4_*, 30 °C). 16](#_Toc22646623)

[Figure S24. Optimized conformers of yuccalechin B (**26**) in DFT/B3LYP/6-31G(d,p)/IEFPCM/MeOH level of theory. 17](#_Toc22646624)

[Figure S25. Calculated DP4+ probabilities of yuccalechin B (**26**) using mpw1pw91/6-111G+(d,p)/IEFPCM/ methanol level of theory. Isomer 1 is 2”*R*,3”*S*,2*S*,3*S* and isomer 2 is 2”*R*,3”*S*,2*R*,3*S*. 17](#_Toc22646625)

[Table S2. Calculated and experimental chemical shift values used for DP4+ calculation for (**26**). 18](#_Toc22646626)

[Figure S26. HRESIMS (Q-TOF) analysis of yuccalechin B (**26**) in negative ion mode. 19](#_Toc22646627)

[Figure S27. ^1^H NMR spectrum of yuccalechin C (**29**) (500 MHz, MeOH-*d_4_*, 30 °C). 20](#_Toc22646628)

[Figure S28. ^13^C NMR spectrum of yuccalechin C (**29**) (125 MHz, MeOH-*d_4_*, 30 °C). 20](#_Toc22646629)

[Figure S29. ^1^H-^1^H COSY NMR spectrum of yuccalechin C (**29**) (500 MHz, MeOH-*d_4_*, 30 °C). 21](#_Toc22646630)

[Figure S30. ^1^H-^1^H ROESY (250 ms) NMR spectrum of yuccalechin C (**29**) (500 MHz, MeOH-*d_4_*, 30 °C). 21](#_Toc22646631)

[Figure S31. ^1^H-^13^C HSQC NMR spectrum of yuccalechin C (**29**) (500/125 MHz, MeOH-*d_4_*, 30 °C). 22](#_Toc22646632)

[Figure S32. ^1^H-^13^C H2BC NMR spectrum of yuccalechin C (**29**) (500/125 MHz, MeOH-*d_4_*, 30 °C). 22](#_Toc22646633)

[Figure S33. ^1^H-^13^C HMBC (8Hz) NMR spectrum of yuccalechin C (**29**) (500/125 MHz, MeOH-*d_4_*, 30 °C). 23](#_Toc22646634)

[Figure S34. Optimized conformers of yuccalechin C (**29**) and their contribution to Boltzmann averaging at DFT/B3LYP/6-31G(d) level of theory in gas phase. 23](#_Toc22646635)

[Figure S35. Calculated DP4+ probabilities of yuccalechin C (**29**) using mpw1pw91/6-111G+(d,p)/ CPCM/methanol level of theory. Isomer 1 is 2”*R*,3”*R*,2*R*,3*R* and isomer 2 is 2”*R*,3”*R*,2*R*,3*S* and isomer 3 is 2”*R*,3”*R*,2*S*,3*R*. 24](#_Toc22646636)

[Table S3. Calculated and experimental chemical shift values used for DP4+ calculation for (**29**). 24](#_Toc22646637)

[Figure S36. HRESIMS (Q-TOF) analysis of yuccalechin C (**29**) in negative ion mode. 25](#_Toc22646638)

[Figure S37. ^1^H NMR spectrum of yuccaol E (**37**) (500 MHz, MeOH-*d_4_*, 30 °C). 26](#_Toc22646639)

[Figure S38. ^13^C NMR spectrum of yuccaol E (**37**) (125 MHz, MeOH-*d_4_*, 30 °C). 26](#_Toc22646640)

[Figure S39. ^1^H NMR spectrum of naringenin (**38**) (500 MHz, MeOH-*d_4_*, 30 °C). 27](#_Toc22646641)

[Figure S40. ^13^C NMR spectrum of naringenin (**38**) (125 MHz, MeOH-*d_4_*, 30 °C). 27](#_Toc22646642)

[Figure S41. ^1^H NMR spectrum of yuccaol C (**39**) (500 MHz, MeOH-*d_4_*, 30 °C). 28](#_Toc22646643)

[Figure S42. ^13^C NMR spectrum of yuccaol C (**39**) (125 MHz, MeOH-*d_4_*, 30 °C). 28](#_Toc22646644)

[Figure S43. ^1^H NMR spectrum of yuccalide A (**40**) (500 MHz, MeOH-*d_4_*, 30 °C). 29](#_Toc22646645)

[Figure S44. ^13^C NMR spectrum of yuccalide A (**40**) (125 MHz, MeOH-*d_4_*, 30 °C). 29](#_Toc22646646)

[Figure S45. ^1^H NMR spectrum of yuccaol D (**42**) (500 MHz, MeOH-*d_4_*, 30 °C). 30](#_Toc22646647)

[Figure S46. ^13^C NMR spectrum of yuccaol D (**42**) (125 MHz, MeOH-*d_4_*, 30 °C). 30](#_Toc22646648)

[Figure S47. ^1^H NMR spectrum of kaempferol (**44**) (500 MHz, MeOH-*d_4_*, 30 °C). 31](#_Toc22646649)

[Figure S48. ^13^C NMR spectrum of kaempferol (**44**) (125 MHz, MeOH-*d_4_*, 30 °C). 31](#_Toc22646650)

[Figure S49. ^1^H NMR spectrum of yuccaol A (**47**) (500 MHz, MeOH-*d_4_*, 30 °C). 32](#_Toc22646651)

[Figure S50. ^13^C NMR spectrum of yuccaol A (**47**) (125 MHz, MeOH-*d_4_*, 30 °C). 32](#_Toc22646652)

[Figure S51. ^1^H NMR spectrum of yuccaol B (**48**) (500 MHz, MeOH-*d_4_*, 30 °C). 33](#_Toc22646653)

[Figure S52. ^13^C NMR spectrum of yuccaol B (**48**) (125 MHz, MeOH-*d_4_*, 30 °C). 33](#_Toc22646654)

[Figure S53. ^1^H NMR spectrum of gloriosaol E (**49**) (500 MHz, MeOH-*d_4_*, 30 °C). 34](#_Toc22646655)

[Figure S54. ^13^C NMR spectrum of gloriosaol E (**49**) (125 MHz, MeOH-*d_4_*, 30 °C). 34](#_Toc22646656)

[Figure S55. ^1^H NMR spectrum of gloriosaol D (**50**) (500 MHz, MeOH-*d_4_*, 30 °C). 35](#_Toc22646657)

[Figure S56. ^13^C NMR spectrum of gloriosaol D (**50**) (125 MHz, MeOH-*d_4_*, 30 °C). 35](#_Toc22646658)

[Figure S57. ^1^H NMR spectrum of gloriosaol A (**54**) (500 MHz, MeOH-*d_4_*, 30 °C). 36](#_Toc22646659)

[Figure S58. ^13^C NMR spectrum of gloriosaol A (**54**) (125 MHz, MeOH-*d_4_*, 30 °C). 36](#_Toc22646660)

[Figure S59. ^1^H NMR spectrum of gloriosaol C (**58**) (500 MHz, MeOH-*d_4_*, 30 °C). 37](#_Toc22646661)

[Figure S60. ^13^C NMR spectrum of gloriosaol C (**58**) (125 MHz, MeOH-*d_4_*, 30 °C). 37](#_Toc22646662)

1. ^1^H NMR spectrum of *trans*-3,3′,5,5′-tetrahydroxy-4′-methoxystilbene (**13**) (500 MHz, MeOH-*d_4_*, 30 °C).


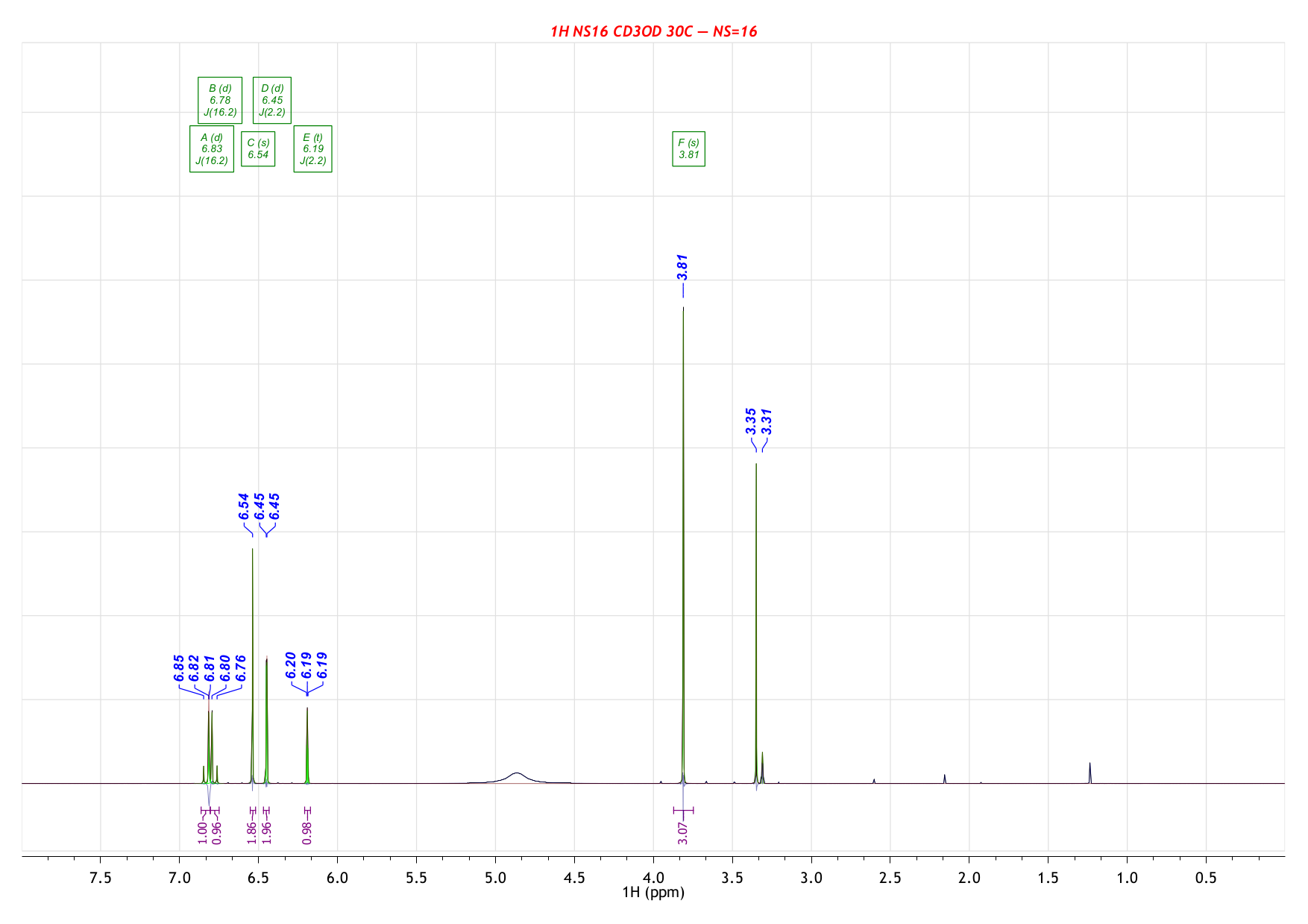


1. ^13^C NMR spectrum of *trans*-3,3′,5,5′-tetrahydroxy-4′-methoxystilbene (**13**) (125 MHz, MeOH-*d_4_*, 30 °C).


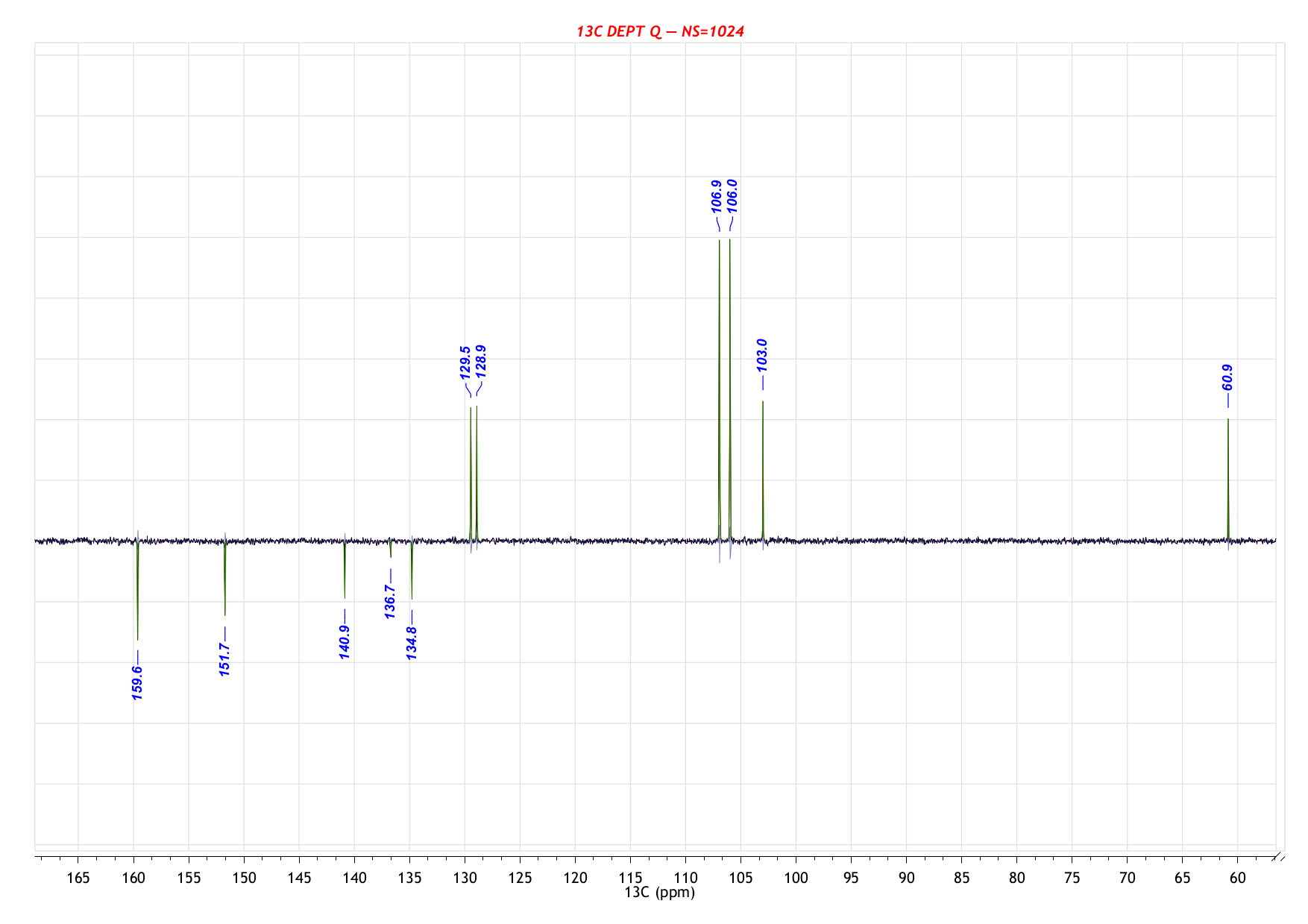


1. ^1^H NMR spectrum of yuccalechin A (**15**) (500 MHz, MeOH-*d_4_*, 30 °C).


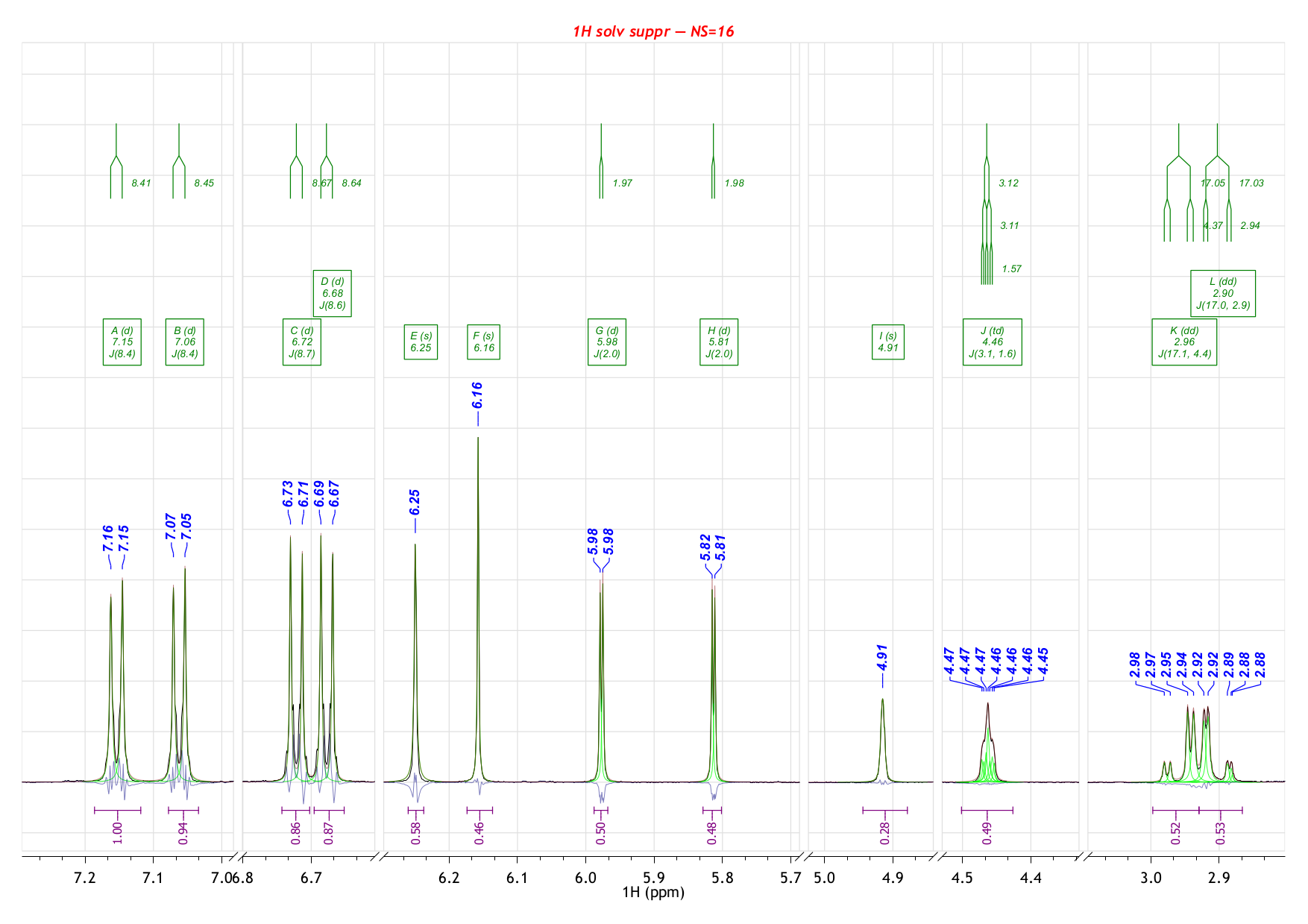


1. ^13^C NMR spectrum of yuccalechin A (**15**) (125 MHz, MeOH-*d_4_*, 30 °C).


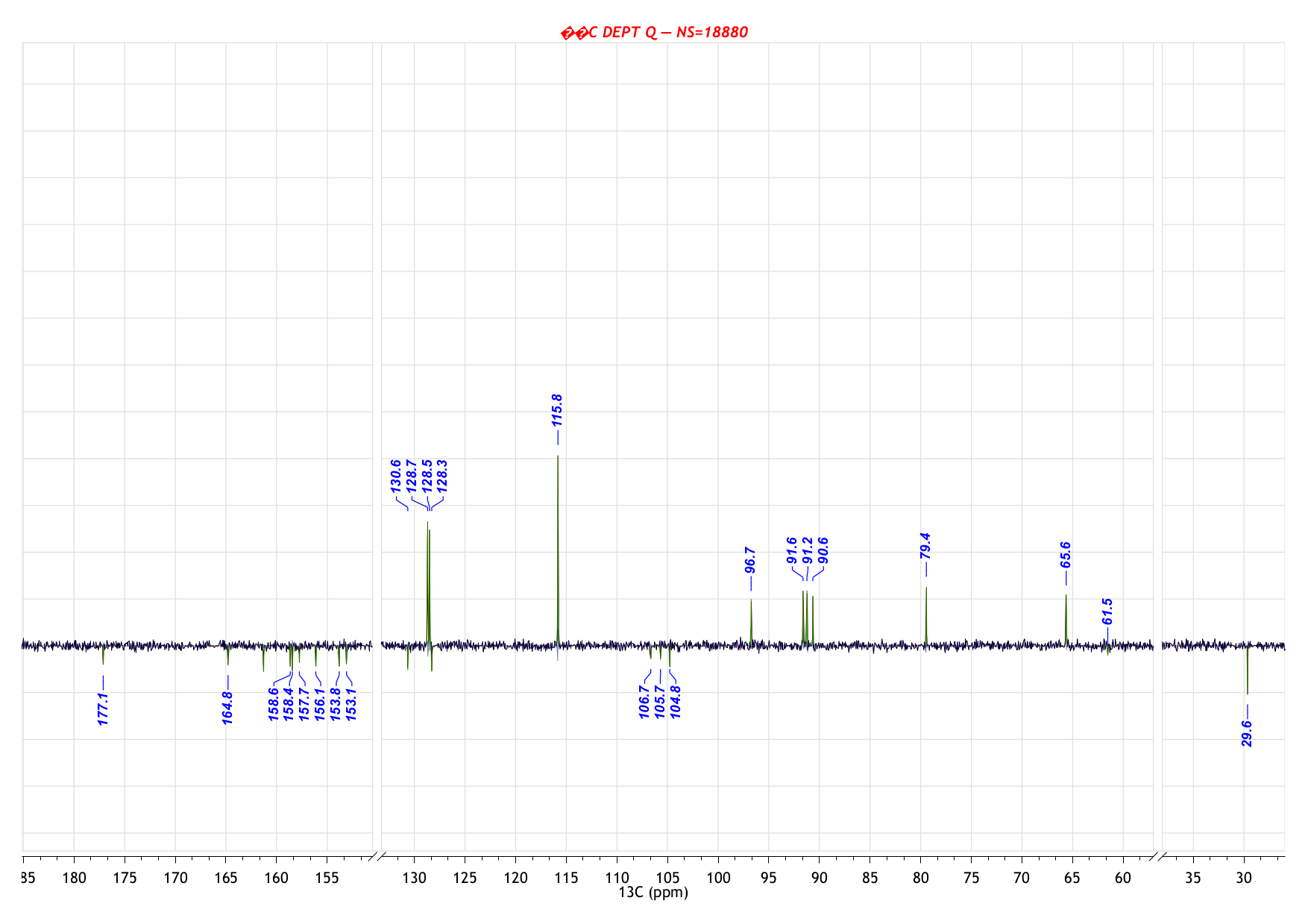


1. ^1^H-^1^H COSY NMR spectrum of yuccalechin A (**15**) (500 MHz, MeOH-*d_4_*, 30 °C).


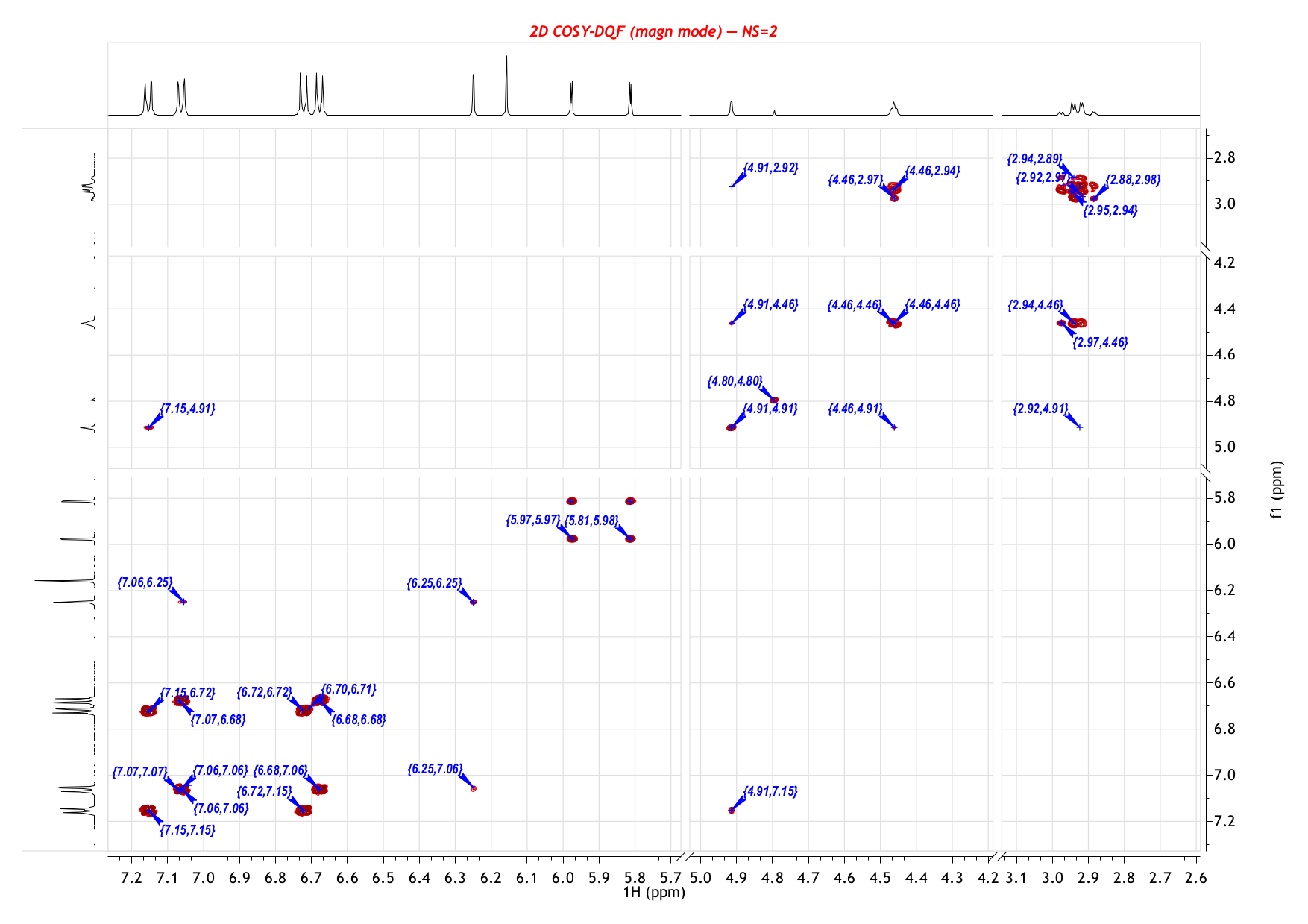


1. ^1^H-^1^H ROESY (250 ms) NMR spectrum of yuccalechin A (**15**) (500 MHz, MeOH-*d_4_*, 30 °C).


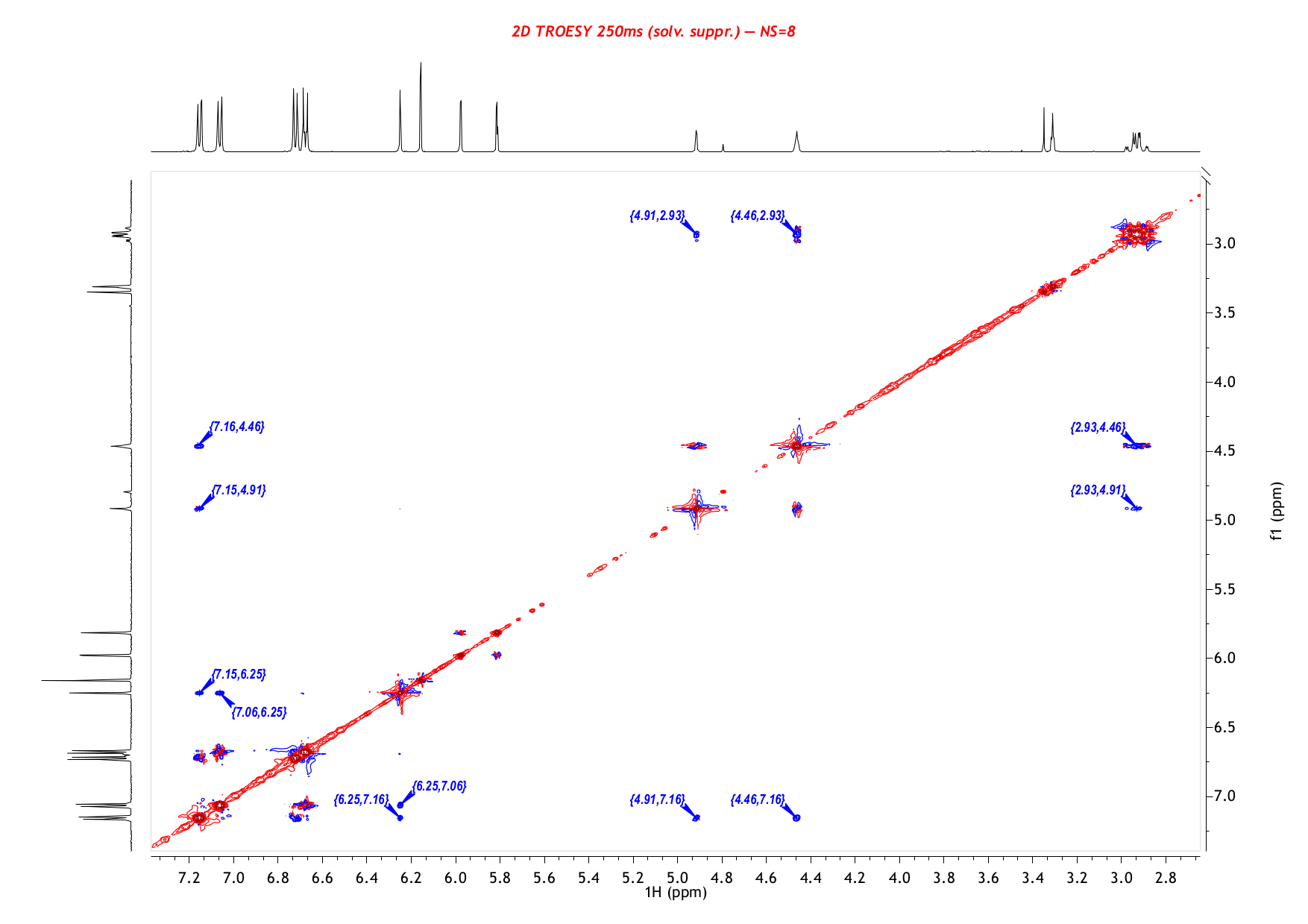


1. ^1^H-^13^C HSQC NMR spectrum of yuccalechin A (**15**) (500/125 MHz, MeOH-*d_4_*, 30 °C).


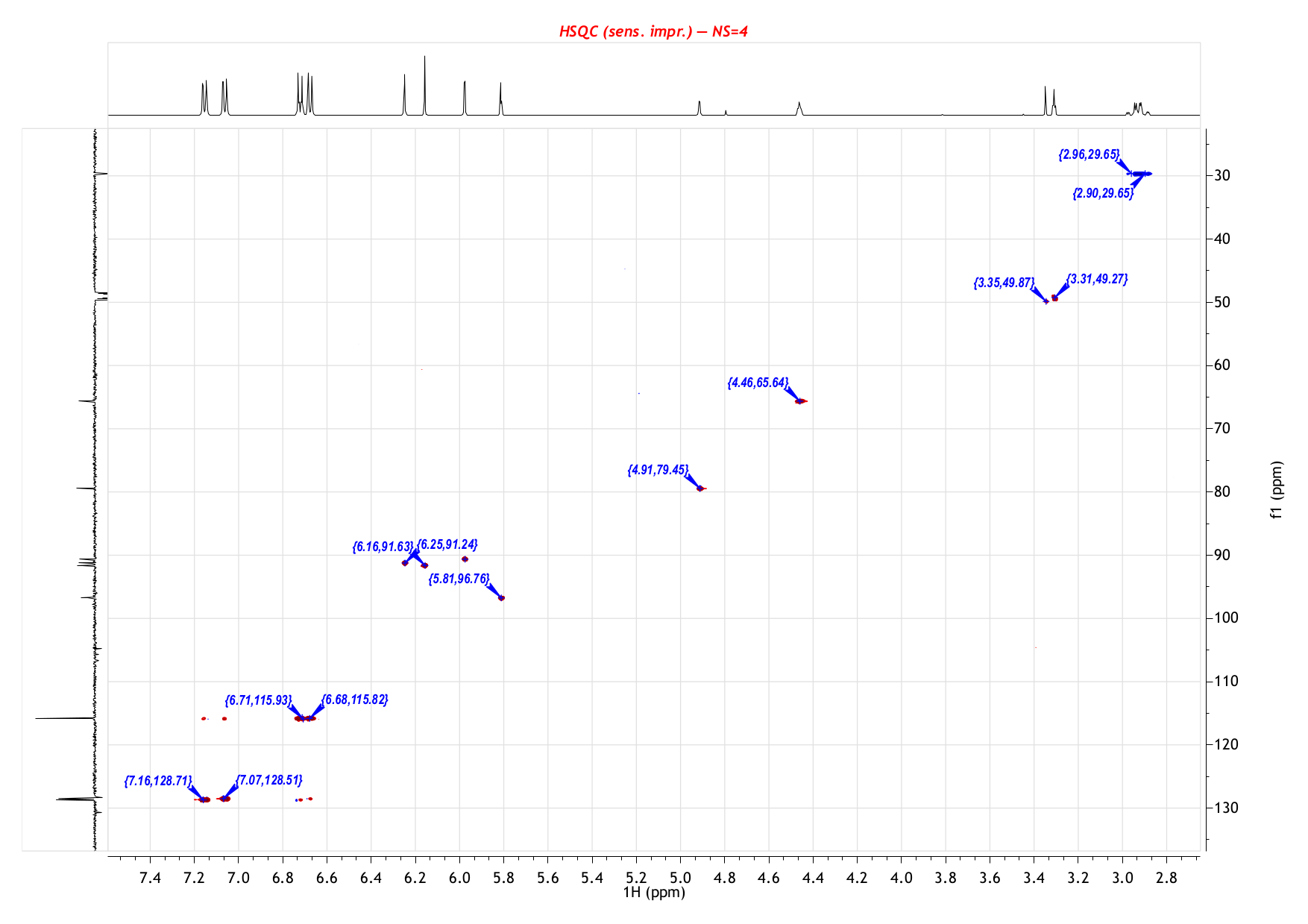


1. ^1^H-^13^C H2BC NMR spectrum of yuccalechin A (**15**) (500/125 MHz, MeOH-*d_4_*, 30 °C).


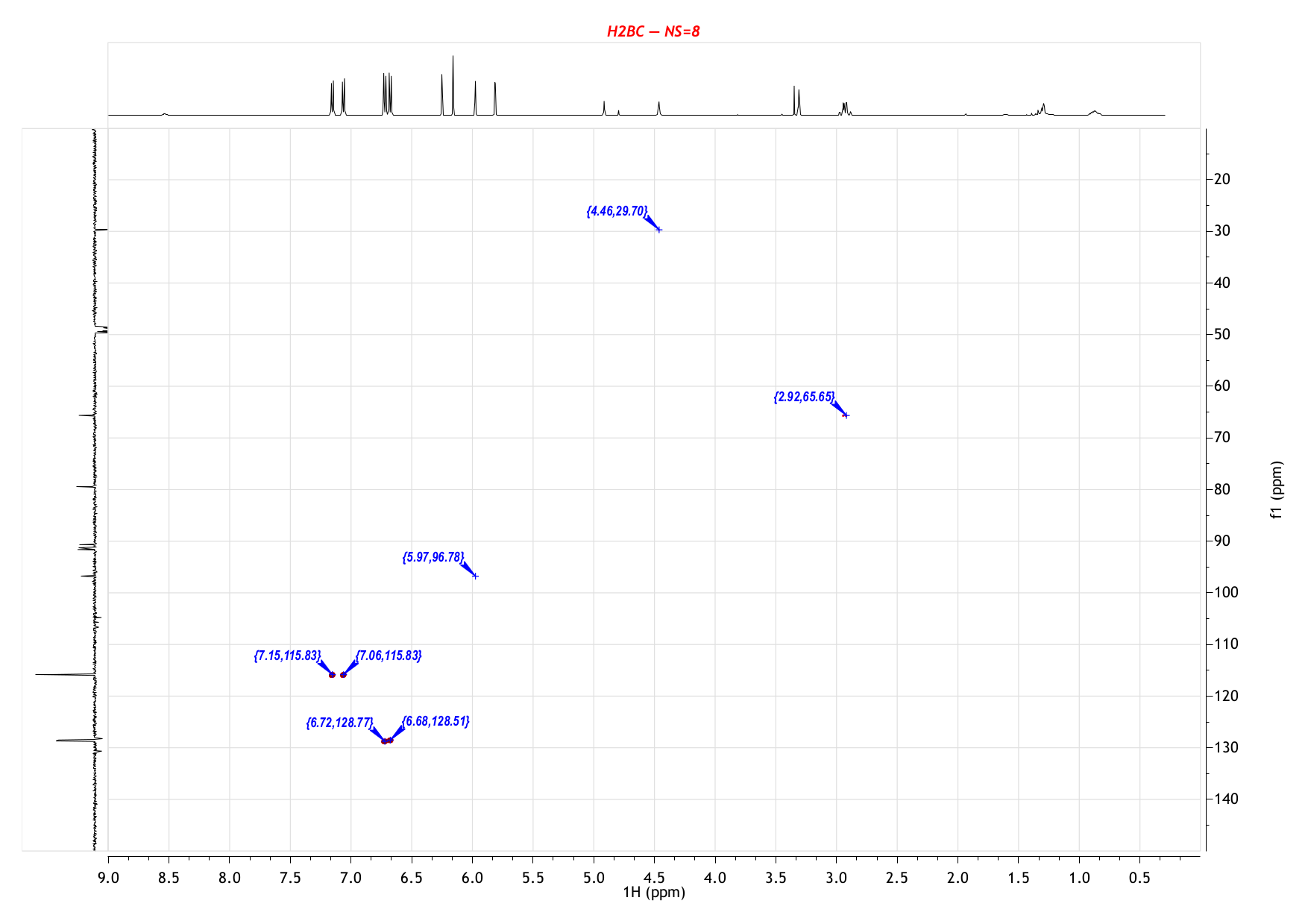


1. ^1^H-^13^C HMBC (8 Hz) NMR spectrum of yuccalechin A (**15**) (500/125 MHz, MeOH-*d_4_*, 30 °C).


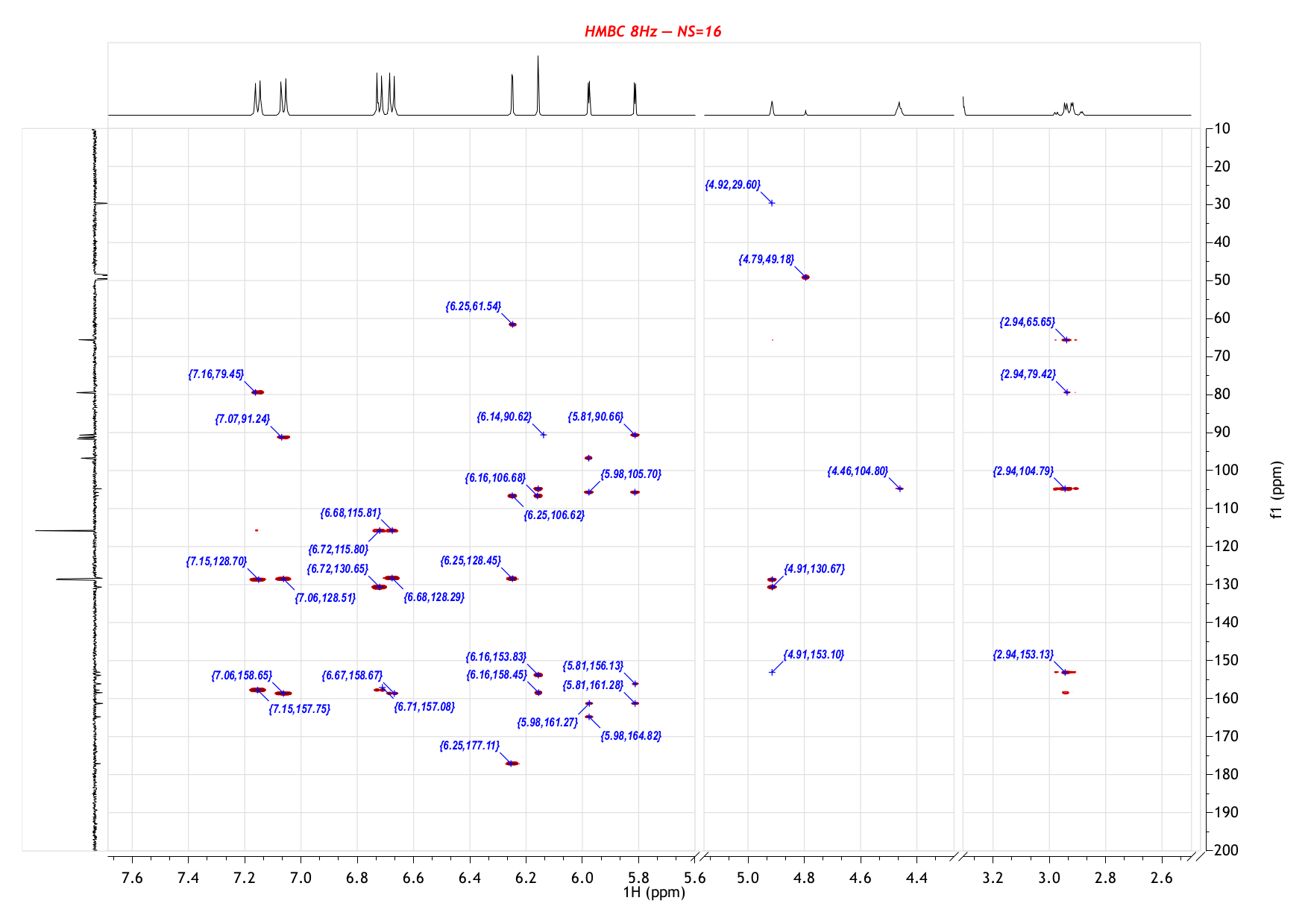


1. Optimized conformers of yuccalechin A (**15**) in DFT/B3LYP/6-31G(d,p)/IEFPCM/methanol level of theory.


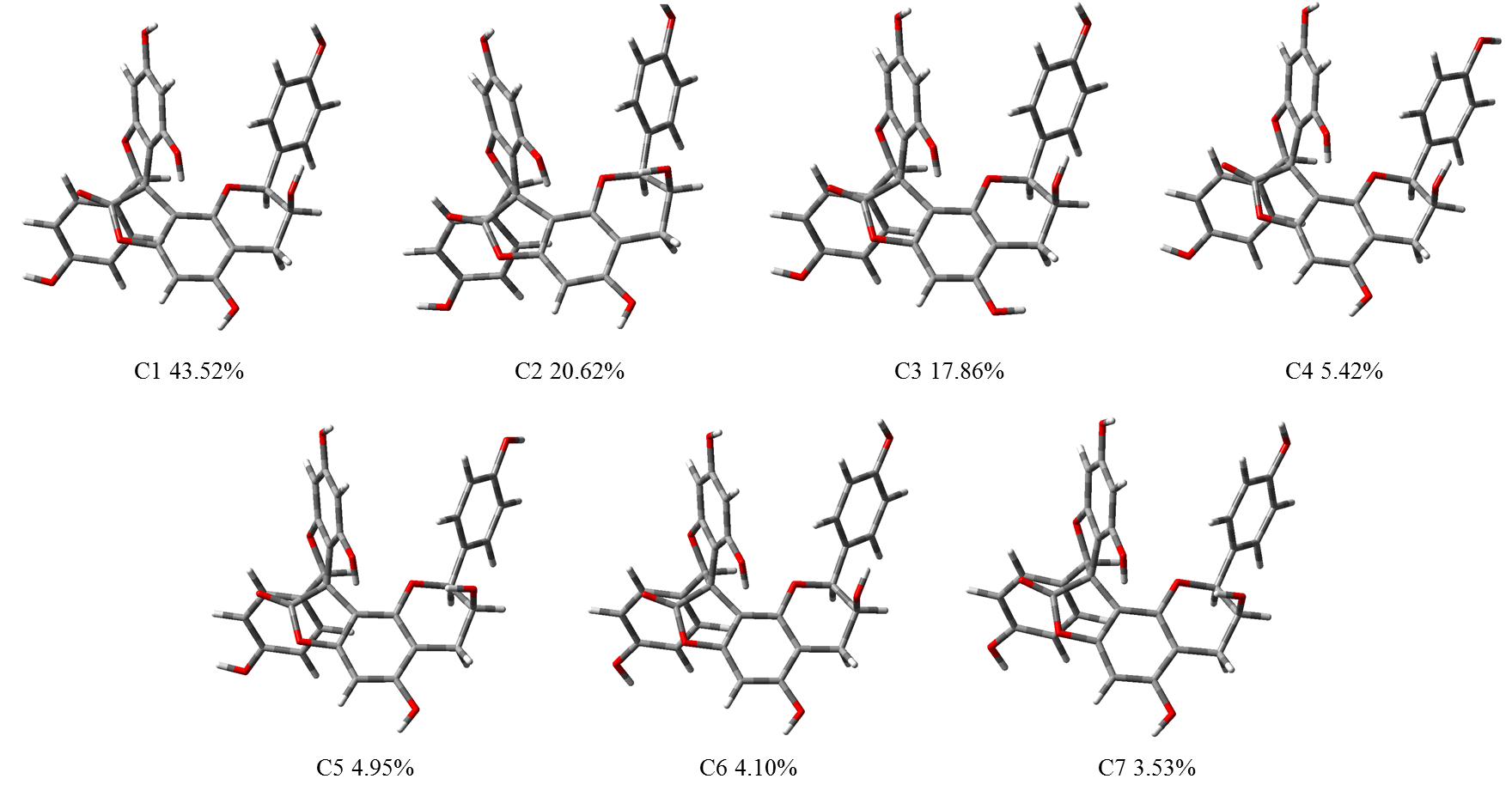


1.
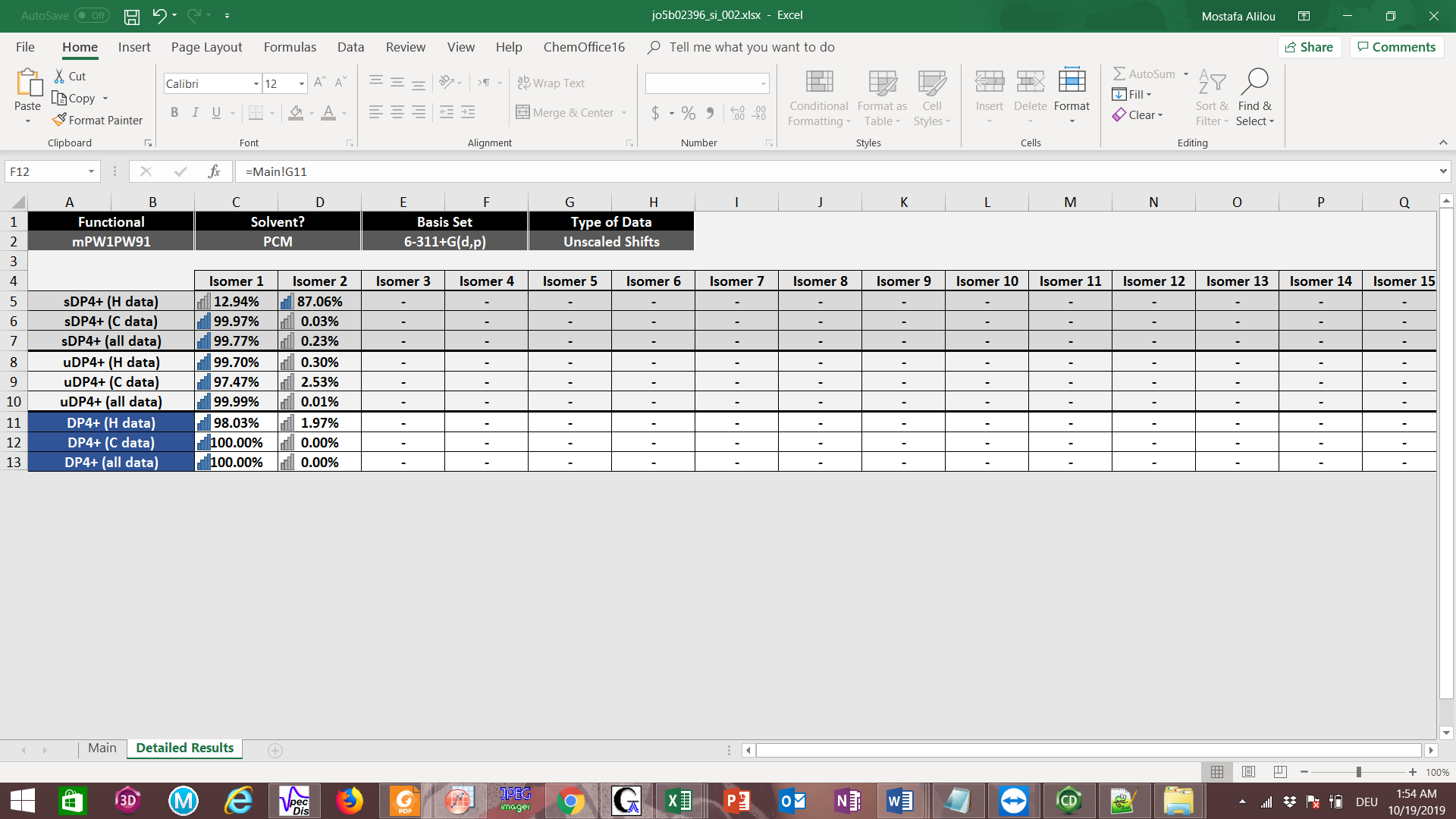
Calculated DP4+ probabilities of yuccalechin A (**15**) using mpw1pw91/6-111G+(d,p)/ IEFPCM/methanol level of theory. Isomer 1 is 2”*R*,3”*S*,2*R*,3*R* and isomer 2 is 2”*R*,3”S,2*S*,3*R*.

Table S1. Calculated and experimental chemical shift values used for DP4+ calculation for (**15**).

| **Atom** | **Exp.** | **Isomer 1** | **Isomer 2** |
| --- | --- | --- | --- |
| C | 161.3 | 168.2 | 167.9 |
| C | 96.7 | 100.2 | 99.7 |
| C | 156.1 | 161.7 | 161.4 |
| C | 105.7 | 109.7 | 109.0 |
| C | 164.8 | 170.4 | 170.8 |
| C | 90.6 | 96.3 | 95.3 |
| C | 91.2 | 94.9 | 95.4 |
| C | 61.5 | 66.9 | 67.0 |
| C | 177.1 | 185.2 | 185.1 |
| C | 153.8 | 161.2 | 161.2 |
| C | 106.7 | 104.6 | 105.0 |
| C | 91.6 | 96.2 | 96.5 |
| C | 158.4 | 165.7 | 165.1 |
| C | 104.8 | 109.8 | 111.8 |
| C | 153.1 | 160.8 | 160.6 |
| C | 128.3 | 131.7 | 131.6 |
| C | 128.5 | 136.5 | 136.6 |
| C | 115.8 | 120.2 | 120.1 |
| C | 158.6 | 165.4 | 165.5 |
| C | 115.8 | 119.6 | 120.1 |
| C | 128.5 | 136.1 | 136.3 |
| C | 29.6 | 30.1 | 31.0 |
| C | 65.6 | 70.4 | 72.5 |
| C | 79.4 | 84.2 | 87.6 |
| C | 130.6 | 136.0 | 135.0 |
| C | 128.7 | 134.5 | 138.3 |
| C | 115.8 | 120.1 | 120.3 |
| C | 157.7 | 164.7 | 165.2 |
| C | 115.8 | 121.2 | 121.0 |
| C | 128.7 | 136.1 | 135.3 |
| H | 5.81 | 6.16 | 5.97 |
| H | 5.98 | 6.34 | 6.12 |
| H | 6.25 | 6.23 | 6.40 |
| H | 6.16 | 6.33 | 6.37 |
| H | 7.06 | 7.94 | 8.00 |
| H | 6.68 | 7.11 | 7.11 |
| H | 6.68 | 6.90 | 7.01 |
| H | 7.06 | 7.05 | 7.19 |
| H | 2.96 | 3.02 | 3.33 |
| H | 2.9 | 3.16 | 2.67 |
| H | 4.46 | 4.08 | 3.99 |
| H | 4.91 | 5.23 | 4.61 |
| H | 7.15 | 7.42 | 7.58 |
| H | 6.72 | 6.95 | 7.20 |
| H | 6.72 | 7.23 | 7.13 |
| H | 7.15 | 7.60 | 7.83 |

1. HRESIMS (Q-TOF) analysis of yuccalechin A (**15**) in negative ion mode.


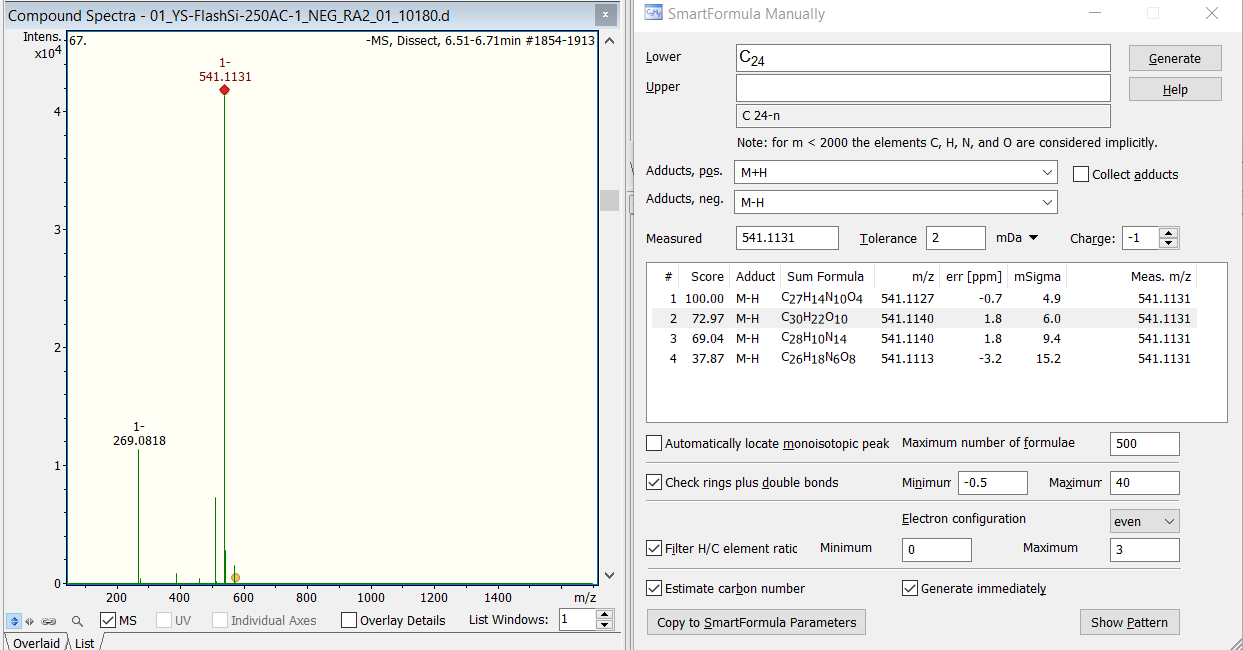


1. ^1^H NMR spectrum of aromadendrin (**16**) (500 MHz, MeOH-*d_4_*, 30 °C).


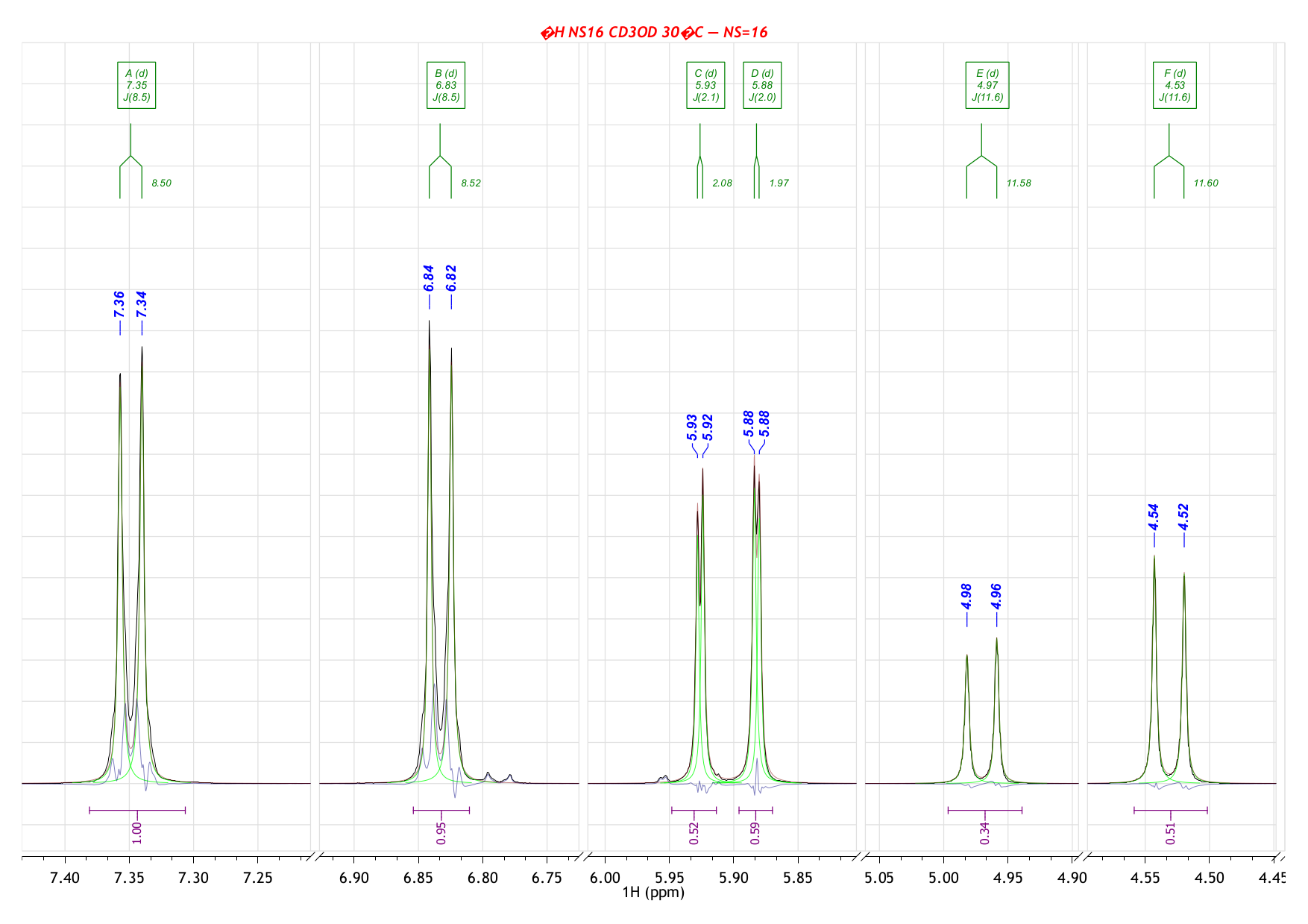


1. ^13^C NMR spectrum of aromadendrin (**16**) (125 MHz, MeOH-*d_4_*, 30 °C).


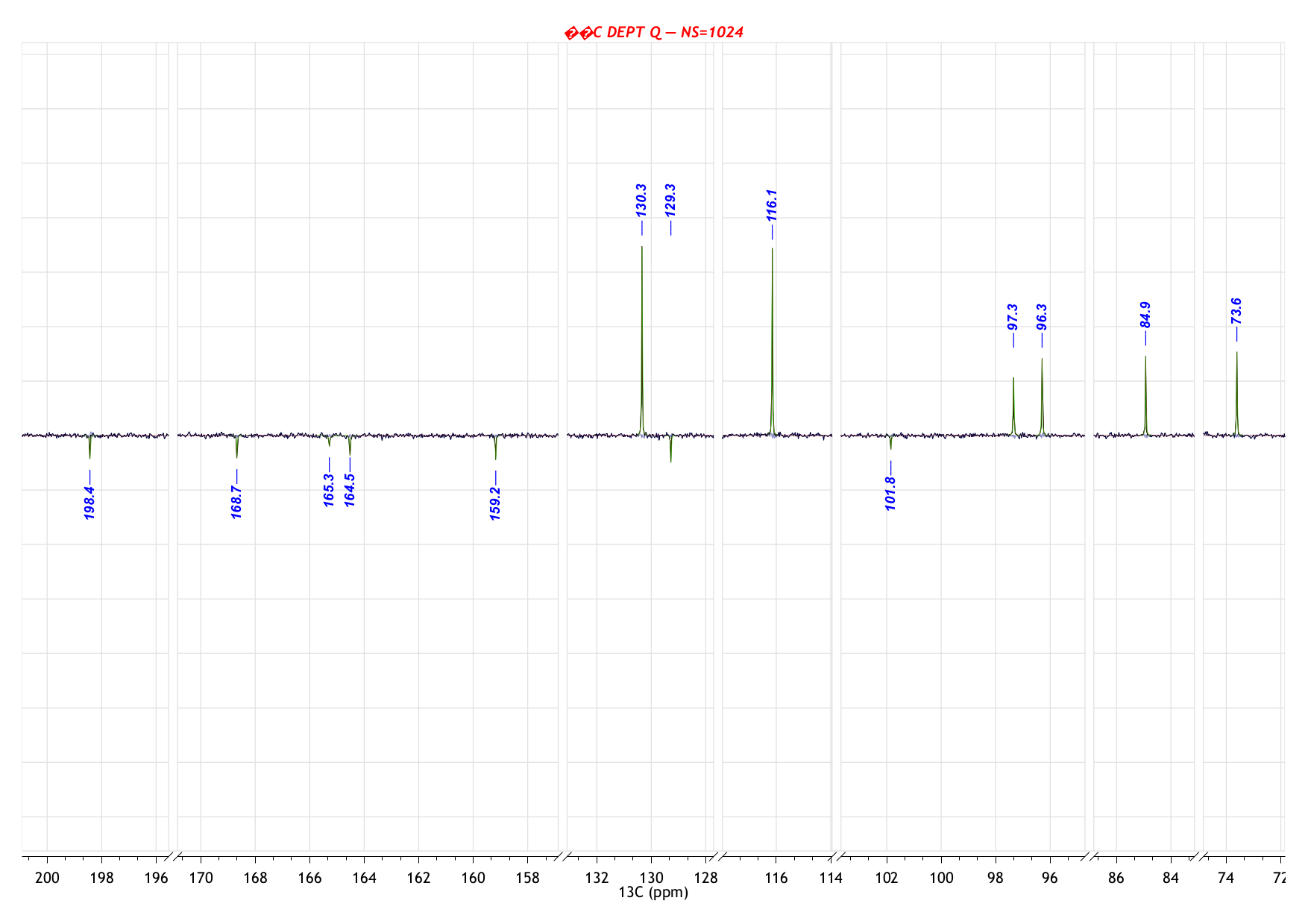


1. ^1^H NMR spectrum of *trans*-resveratrol (**21**) (500 MHz, MeOH-*d_4_*, 30 °C).


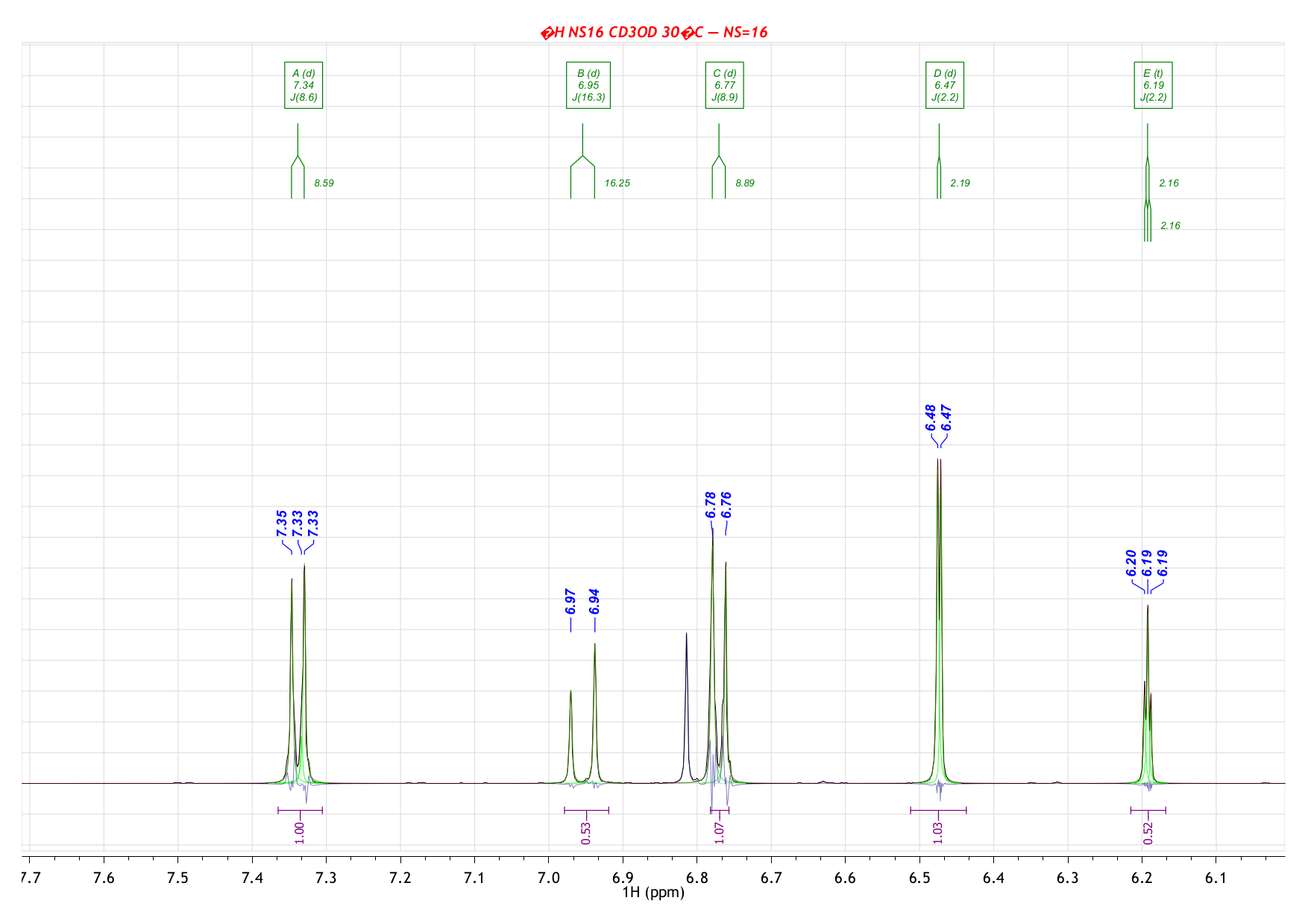


1. ^13^C NMR spectrum of *trans*-resveratrol (**21**) (125 MHz, MeOH-*d_4_*, 30 °C).


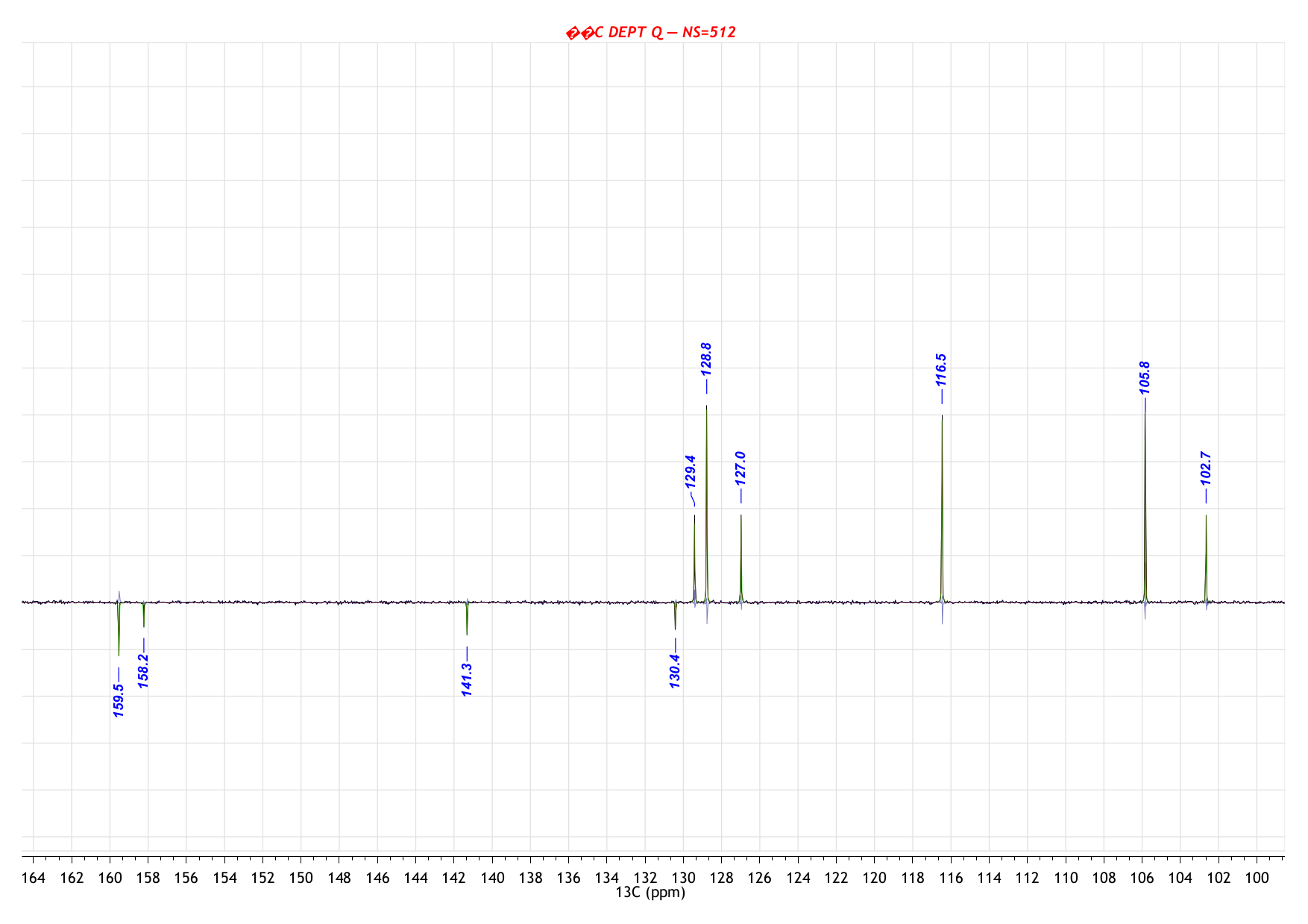


1. ^1^H NMR spectrum of yuccalechin B (**26**) (500 MHz, MeOH-*d_4_*, 30 °C).


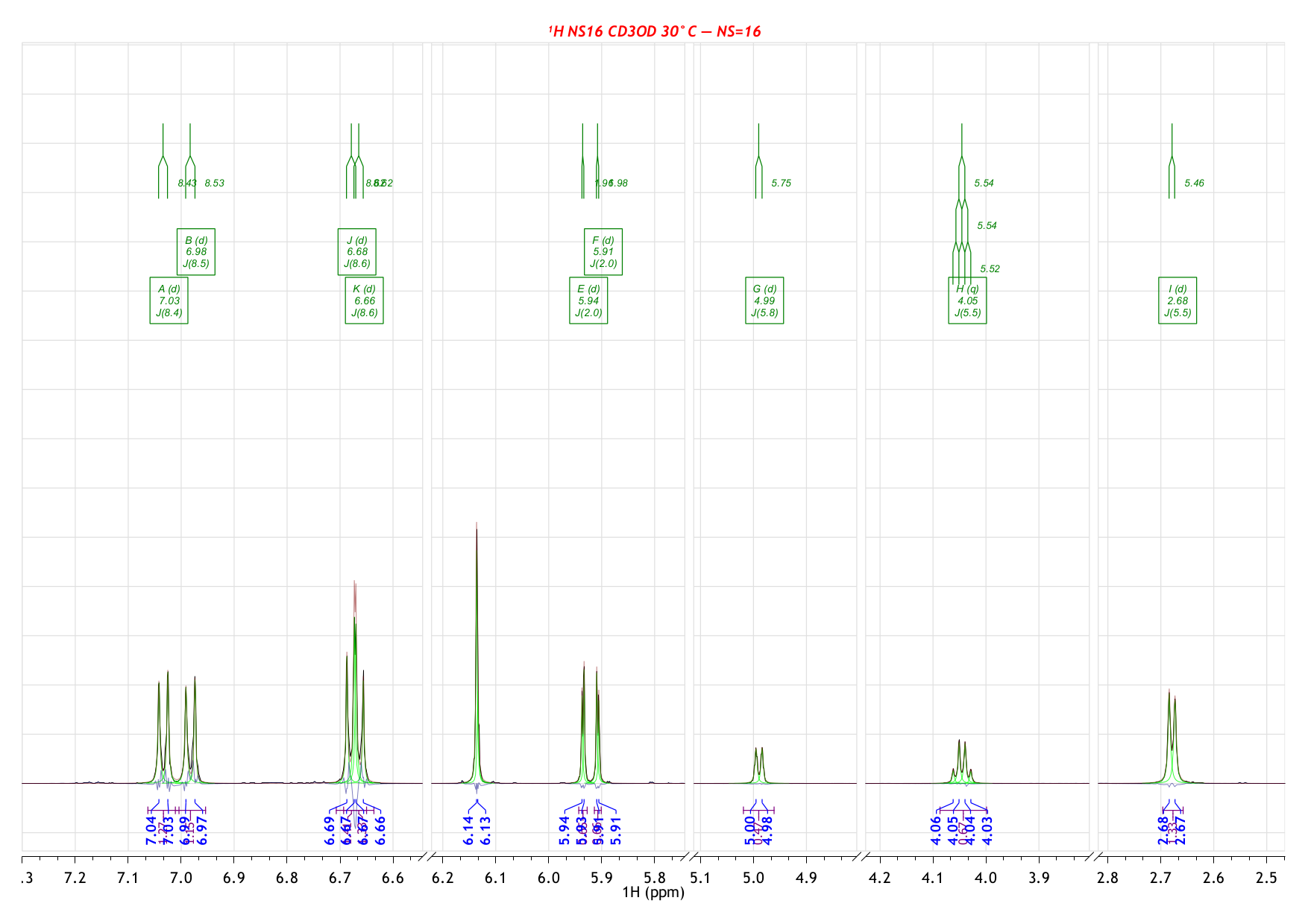


1. ^13^C NMR spectrum of yuccalechin B (**26**) (125 MHz, MeOH-*d_4_*, 30 °C).


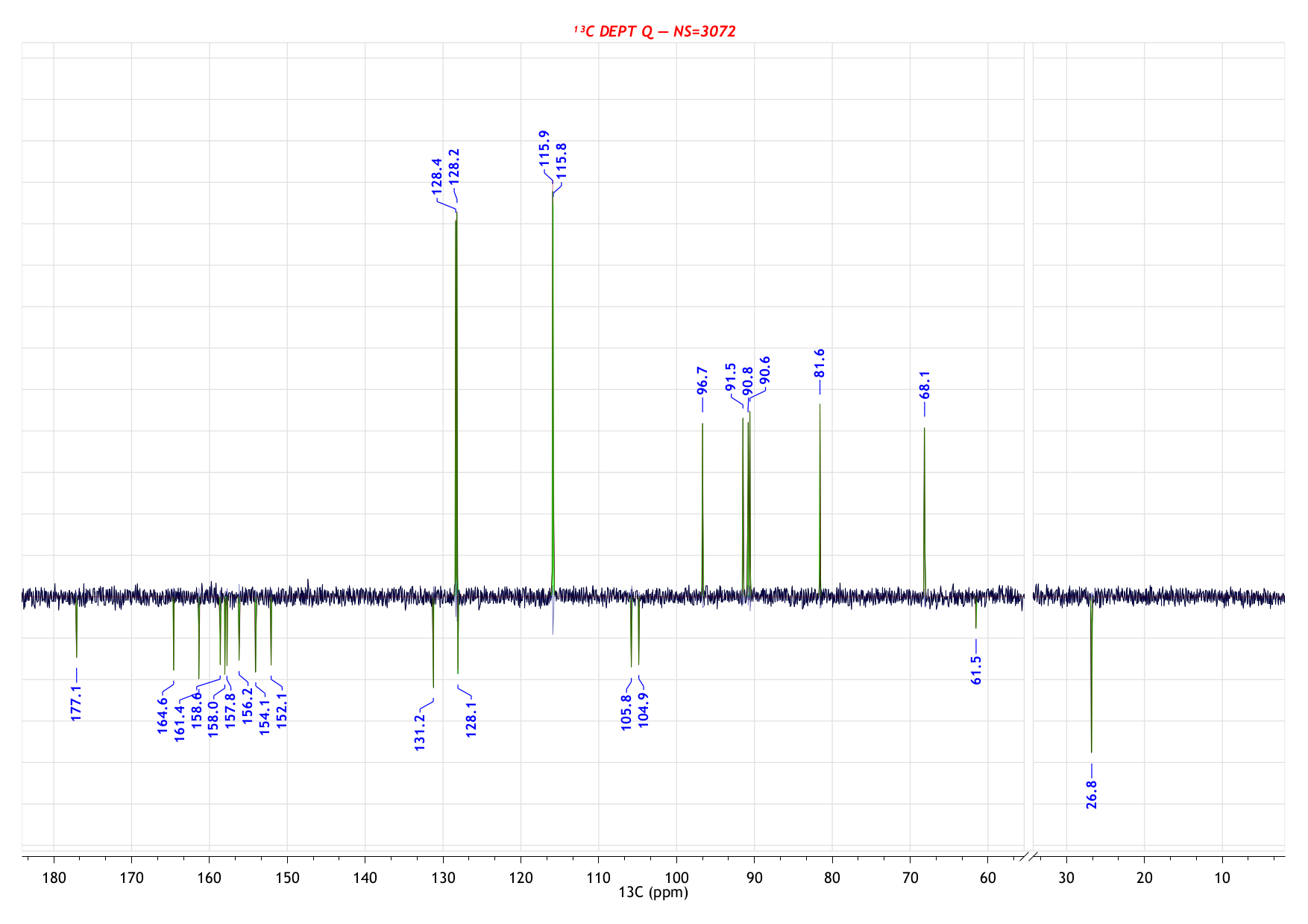


1. ^1^H-^1^H COSY NMR spectrum of yuccalechin B (**26**) (500 MHz, MeOH-*d_4_*, 30 °C).


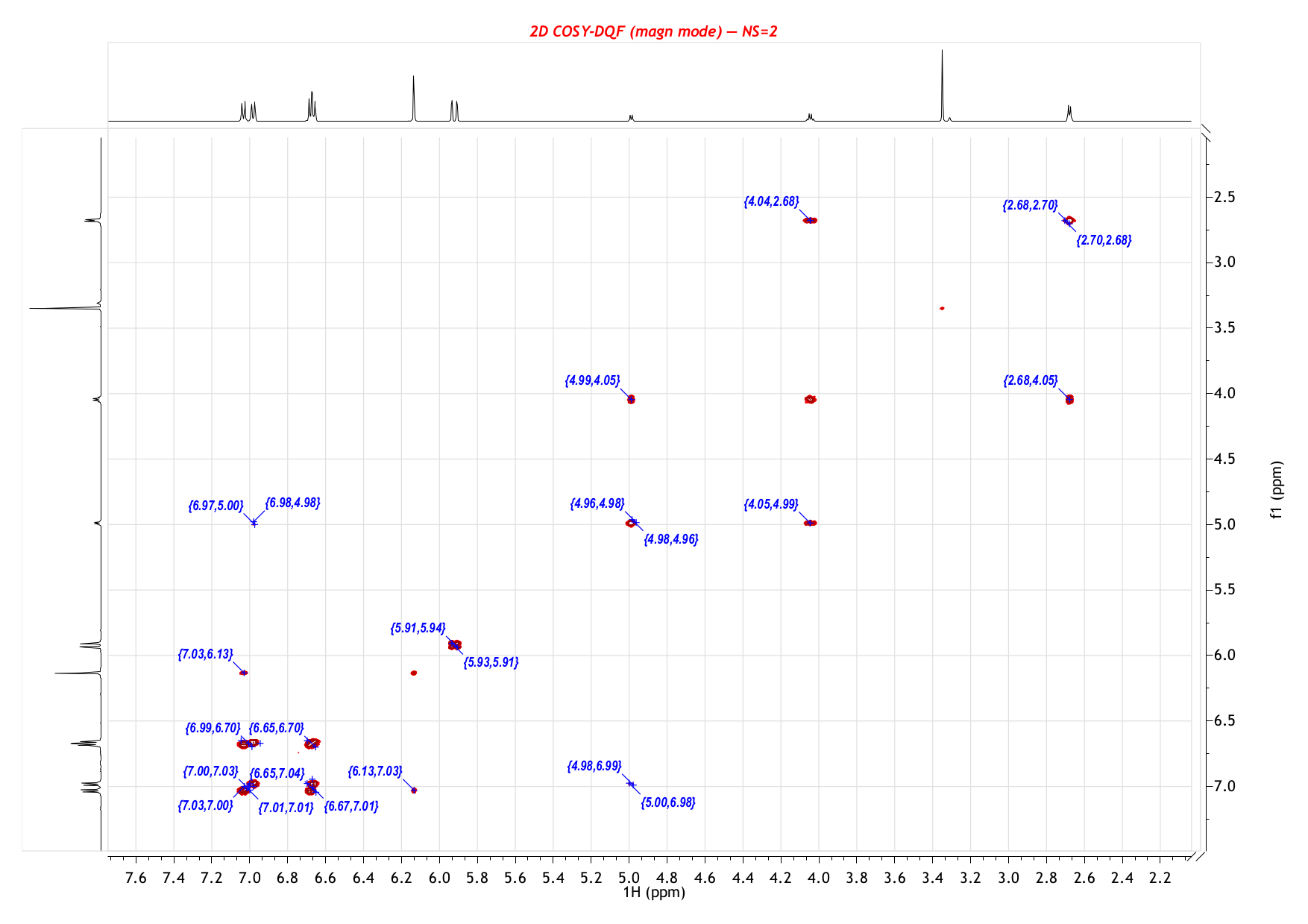


1. ^1^H-^1^H ROESY (250 ms) NMR spectrum of yuccalechin B (**26**) (500 MHz, MeOH-*d_4_*, 30 °C).


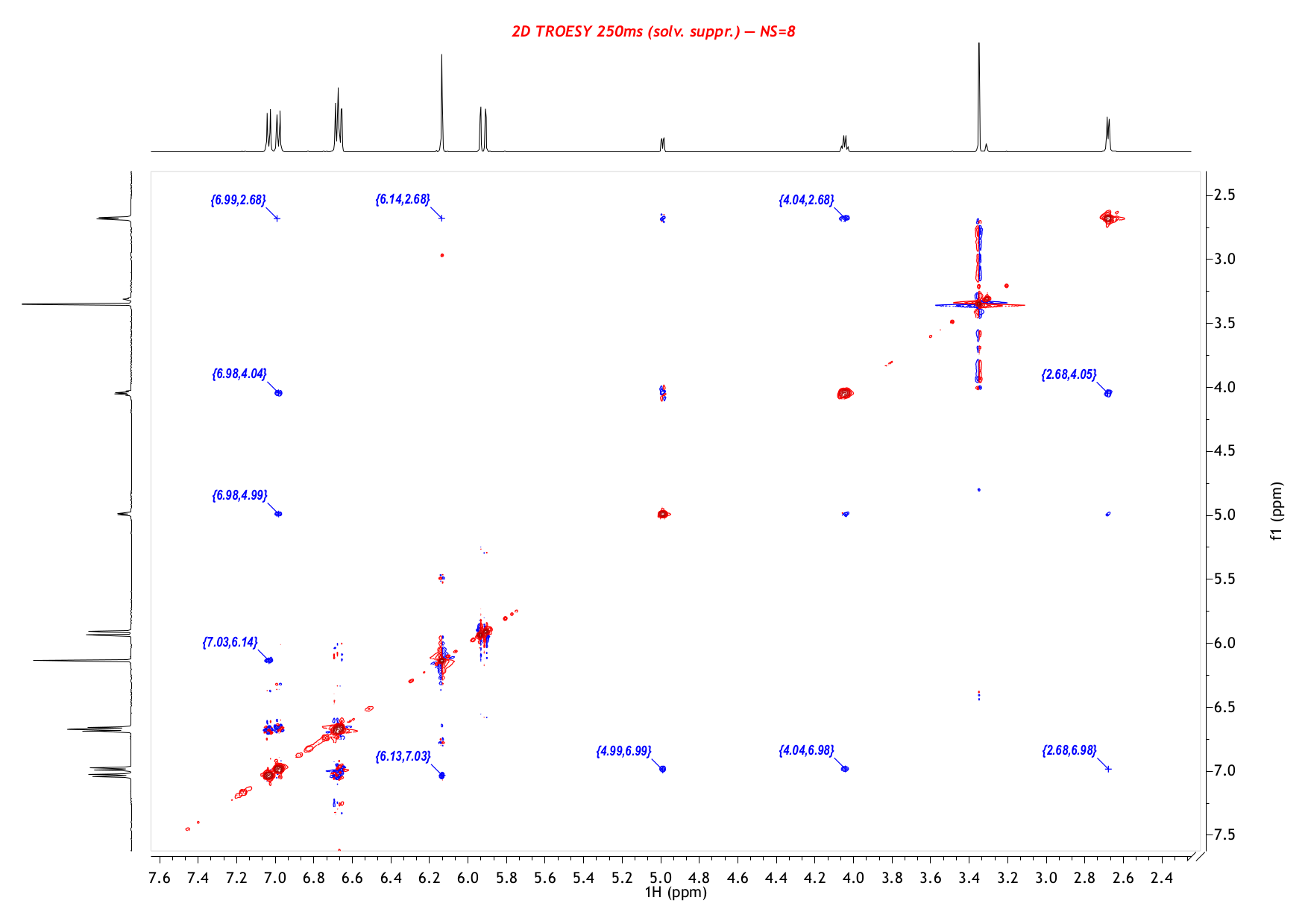


1. ^1^H-^13^C HSQC NMR spectrum of yuccalechin B (**26**) (500/125 MHz, MeOH-*d_4_*, 30 °C).


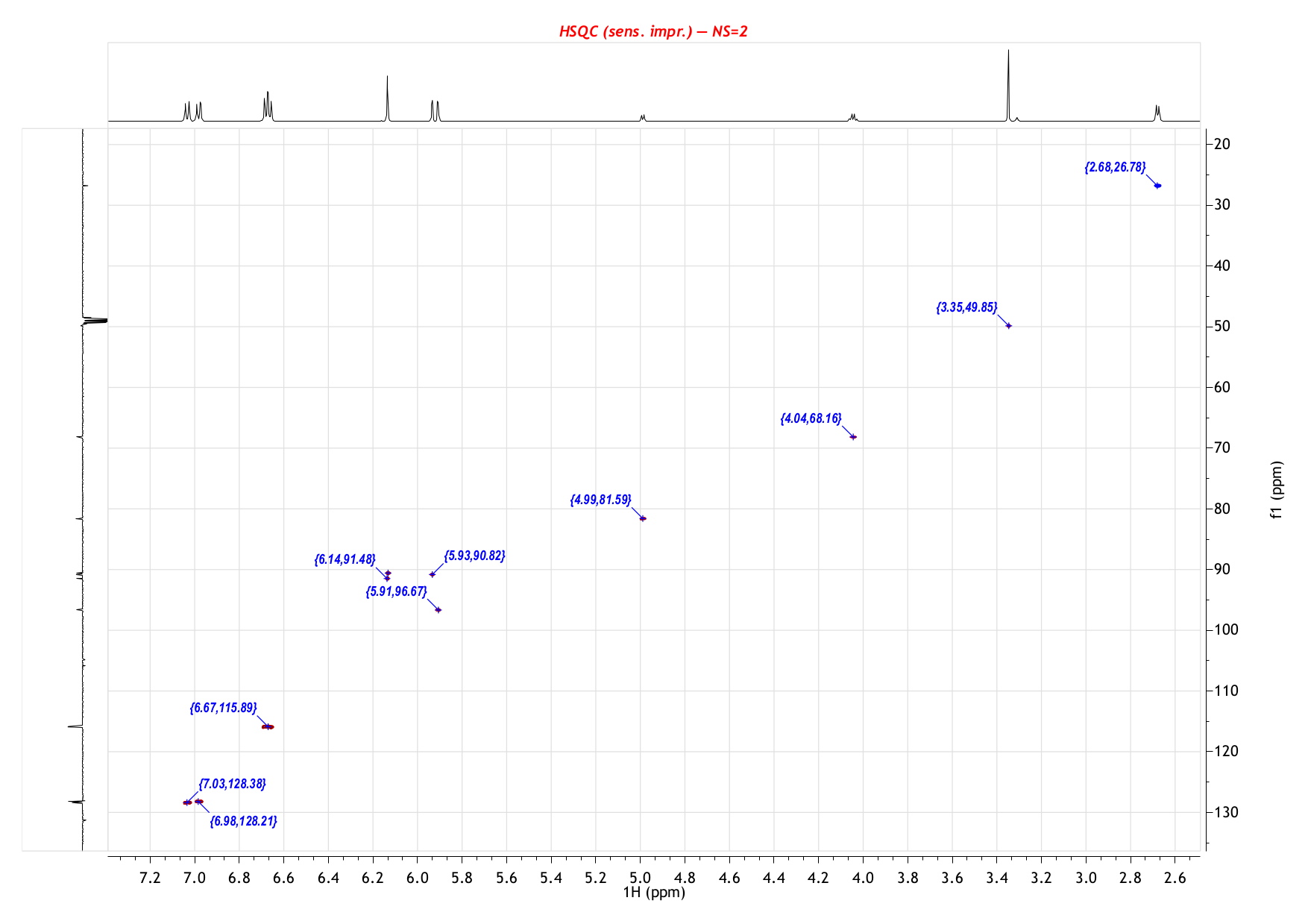


1. ^1^H-^13^C H2BC NMR spectrum of yuccalechin B (**26**) (500/125 MHz, MeOH-*d_4_*, 30 °C).


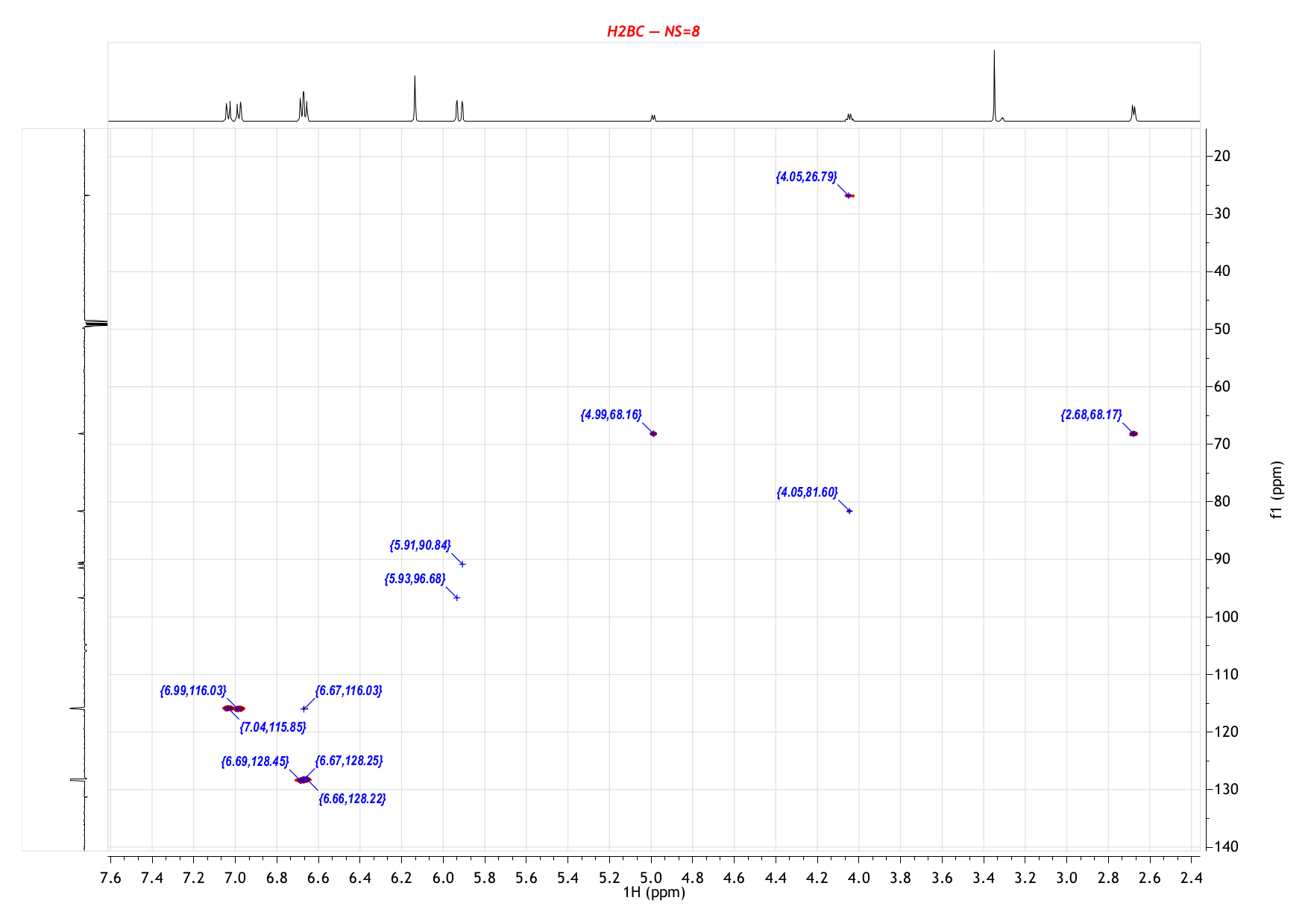


1. ^1^H-^13^C HMBC (8Hz) NMR spectrum of yuccalechin B (**26**) (500/125 MHz, MeOH-*d_4_*, 30 °C).


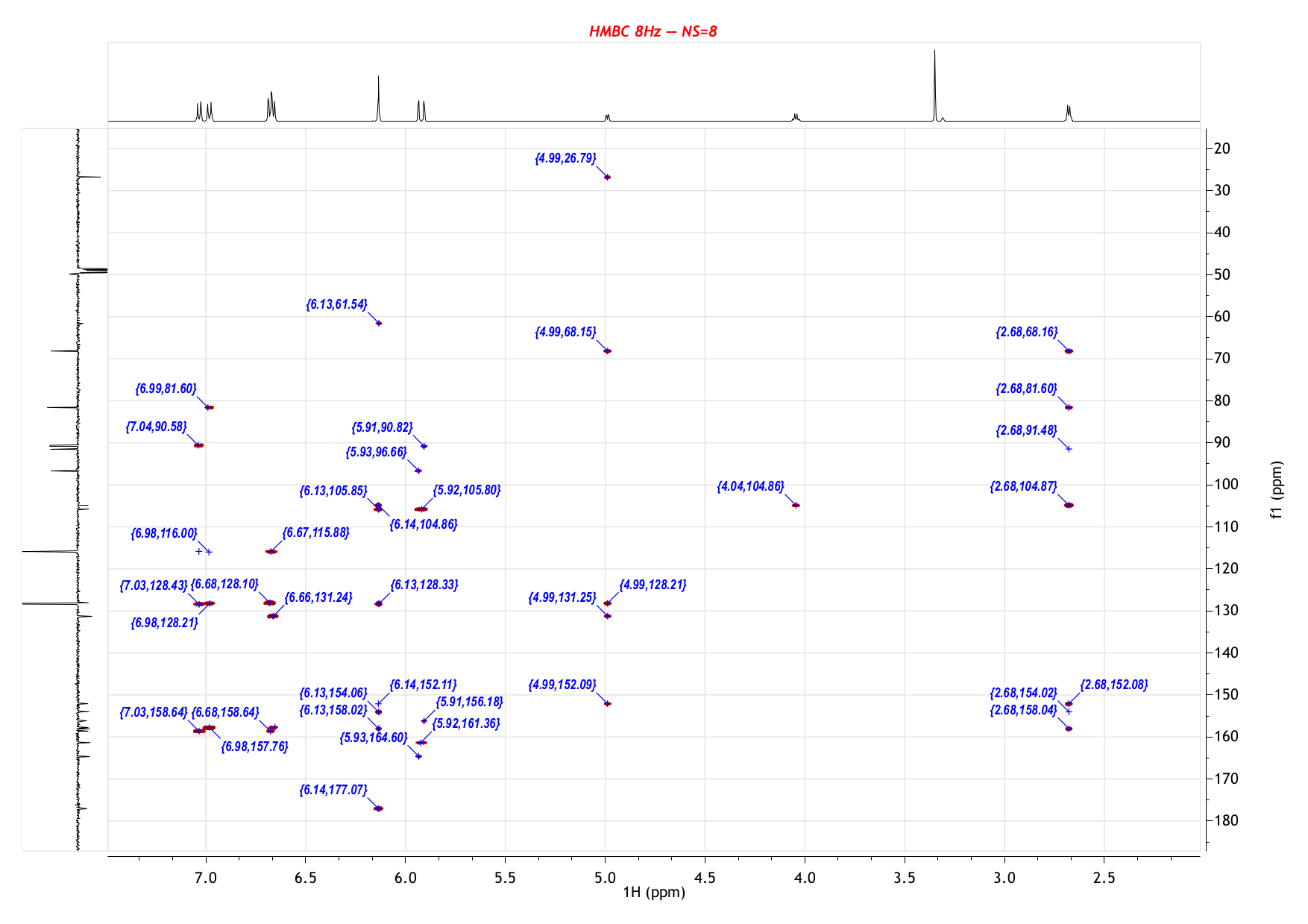


1.
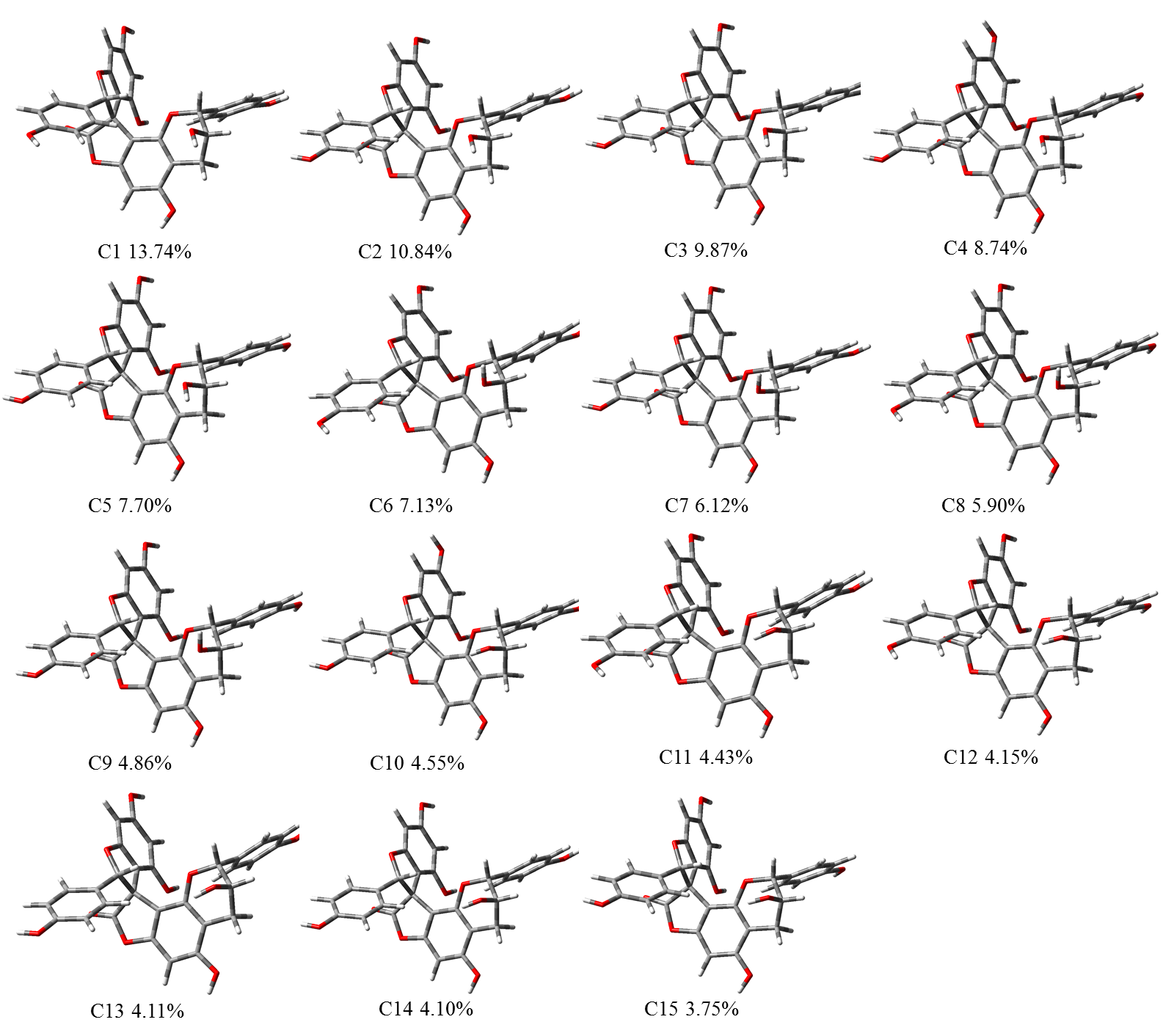
Optimized conformers of yuccalechin B (**26**) in DFT/B3LYP/6-31G(d,p)/IEFPCM/MeOH level of theory.
2. Calculated DP4+ probabilities of yuccalechin B (**26**) using mpw1pw91/6-111G+(d,p)/IEFPCM/ methanol level of theory. Isomer 1 is 2”*R*,3”*S*,2*S*,3*S* and isomer 2 is 2”*R*,3”*S*,2*R*,3*S*.


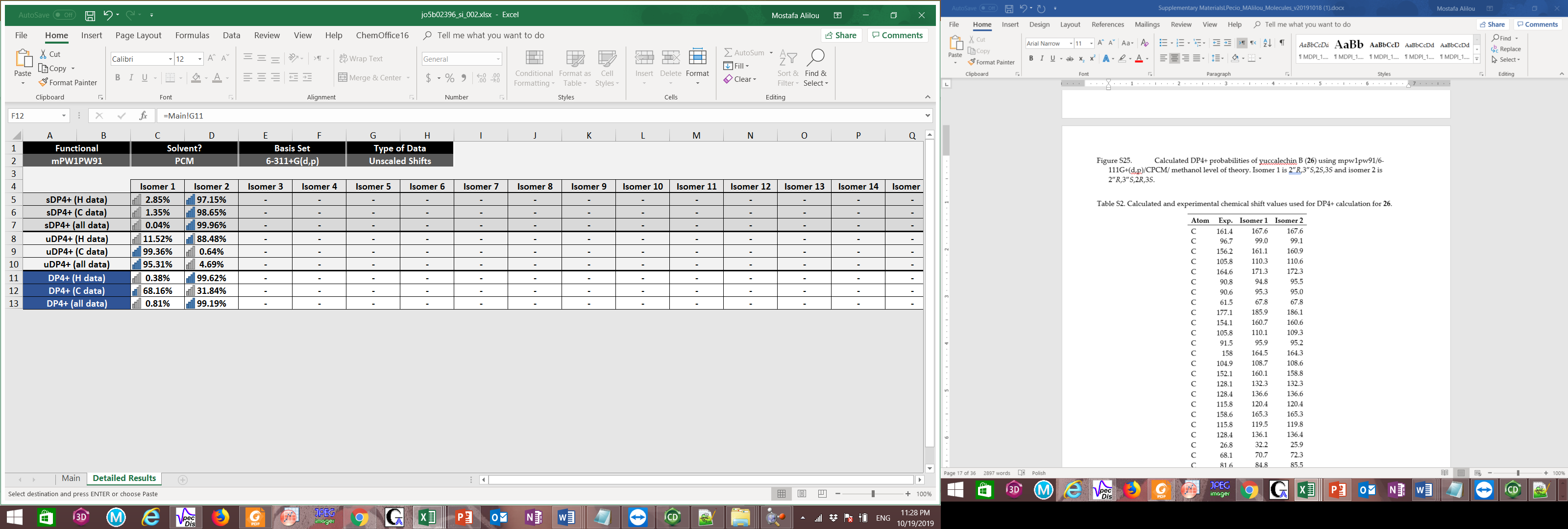


Table S2. Calculated and experimental chemical shift values used for DP4+ calculation for (**26**).

| **Atom** | **Exp.** | **Isomer 1** | **Isomer 2** |
| --- | --- | --- | --- |
| C | 161.4 | 167.6 | 167.6 |
| C | 96.7 | 99.0 | 99.1 |
| C | 156.2 | 161.1 | 160.9 |
| C | 105.8 | 110.3 | 110.6 |
| C | 164.6 | 171.3 | 172.3 |
| C | 90.8 | 94.8 | 95.5 |
| C | 90.6 | 95.3 | 95.0 |
| C | 61.5 | 67.8 | 67.8 |
| C | 177.1 | 185.9 | 186.1 |
| C | 154.1 | 160.7 | 160.6 |
| C | 105.8 | 110.1 | 109.3 |
| C | 91.5 | 95.9 | 95.2 |
| C | 158 | 164.5 | 164.3 |
| C | 104.9 | 108.7 | 108.6 |
| C | 152.1 | 160.1 | 158.8 |
| C | 128.1 | 132.3 | 132.3 |
| C | 128.4 | 136.6 | 136.6 |
| C | 115.8 | 120.4 | 120.4 |
| C | 158.6 | 165.3 | 165.3 |
| C | 115.8 | 119.5 | 119.8 |
| C | 128.4 | 136.1 | 136.4 |
| C | 26.8 | 32.2 | 25.9 |
| C | 68.1 | 70.7 | 72.3 |
| C | 81.6 | 84.8 | 85.5 |
| C | 131.2 | 136.7 | 137.5 |
| C | 128.2 | 136.4 | 133.2 |
| C | 115.9 | 120.2 | 120.5 |
| C | 157.8 | 164.7 | 163.6 |
| C | 115.9 | 120.3 | 119.8 |
| C | 128.2 | 136.6 | 132.9 |
| H | 5.91 | 5.86 | 5.88 |
| H | 5.94 | 6.05 | 6.27 |
| H | 6.13 | 6.51 | 6.46 |
| H | 6.14 | 6.37 | 6.27 |
| H | 7.03 | 7.68 | 7.85 |
| H | 6.68 | 7.15 | 7.18 |
| H | 6.68 | 6.99 | 7.00 |
| H | 7.03 | 7.51 | 7.32 |
| H | 2.68 | 3.05 | 2.26 |
| H | 2.68 | 3.10 | 2.88 |
| H | 4.05 | 3.99 | 4.40 |
| H | 4.99 | 4.92 | 5.50 |
| H | 6.98 | 7.80 | 7.52 |
| H | 6.66 | 7.15 | 7.13 |
| H | 6.66 | 7.15 | 7.01 |
| H | 6.98 | 7.58 | 7.48 |

1. HRESIMS (Q-TOF) analysis of yuccalechin B (**26**) in negative ion mode.


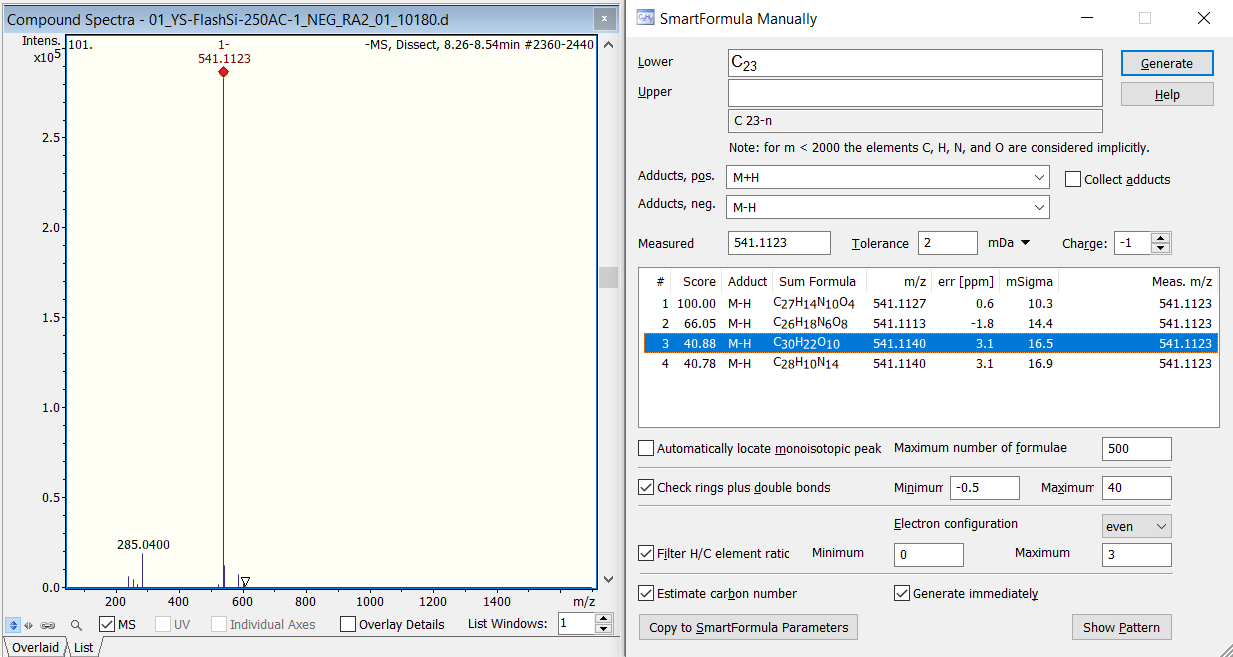


1. ^1^H NMR spectrum of yuccalechin C (**29**) (500 MHz, MeOH-*d_4_*, 30 °C).


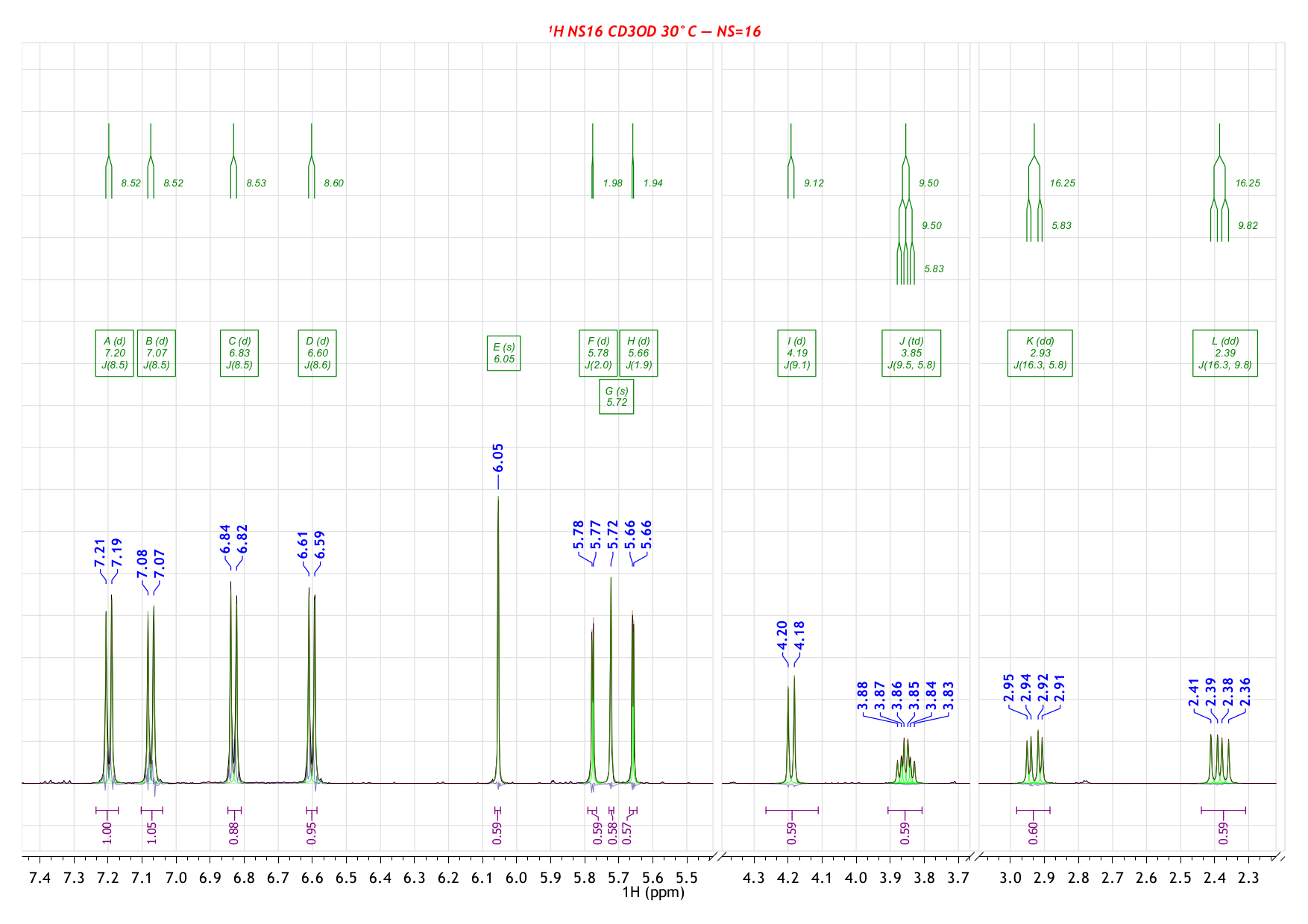


1. ^13^C NMR spectrum of yuccalechin C (**29**) (125 MHz, MeOH-*d_4_*, 30 °C).


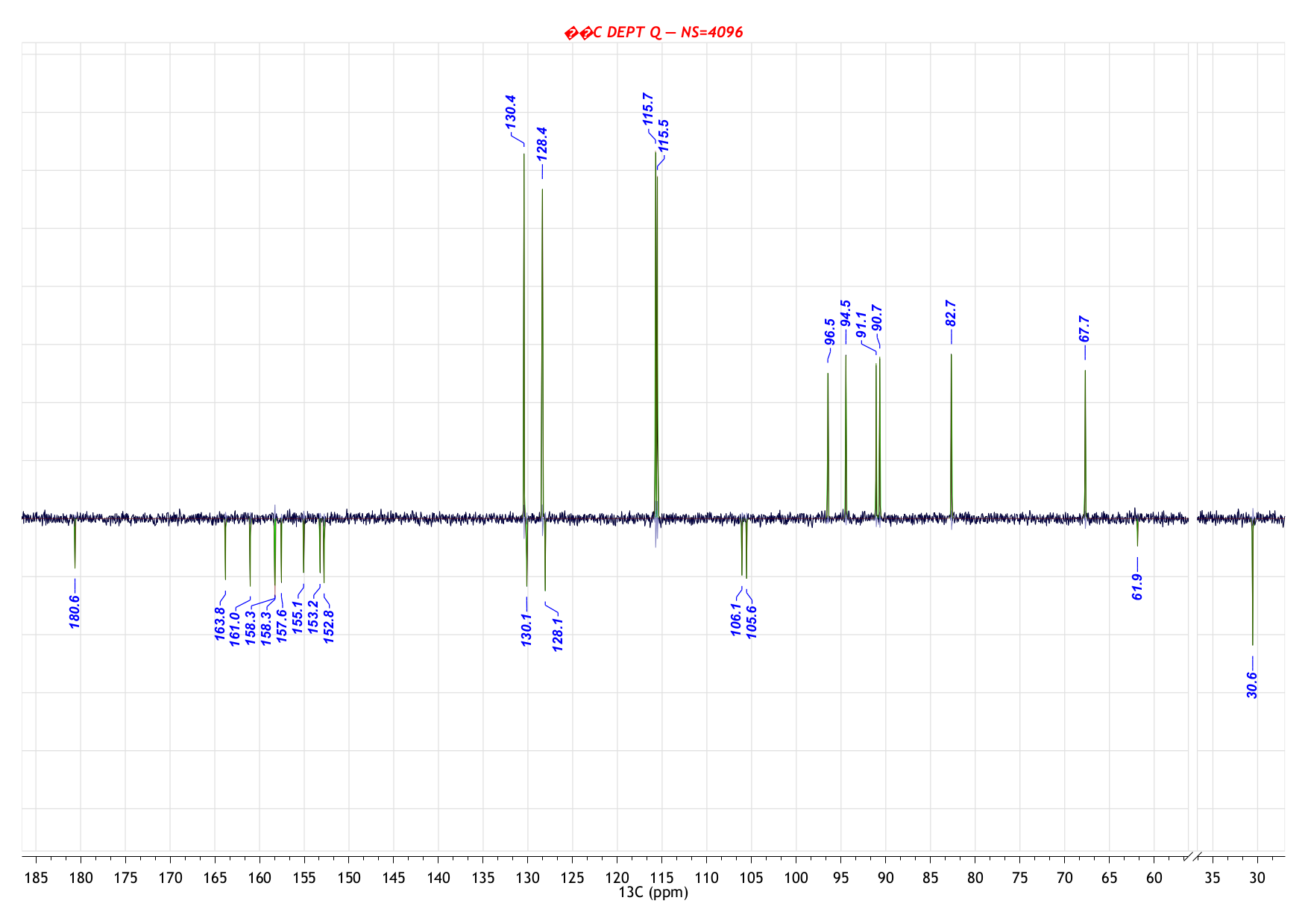


1. ^1^H-^1^H COSY NMR spectrum of yuccalechin C (**29**) (500 MHz, MeOH-*d_4_*, 30 °C).


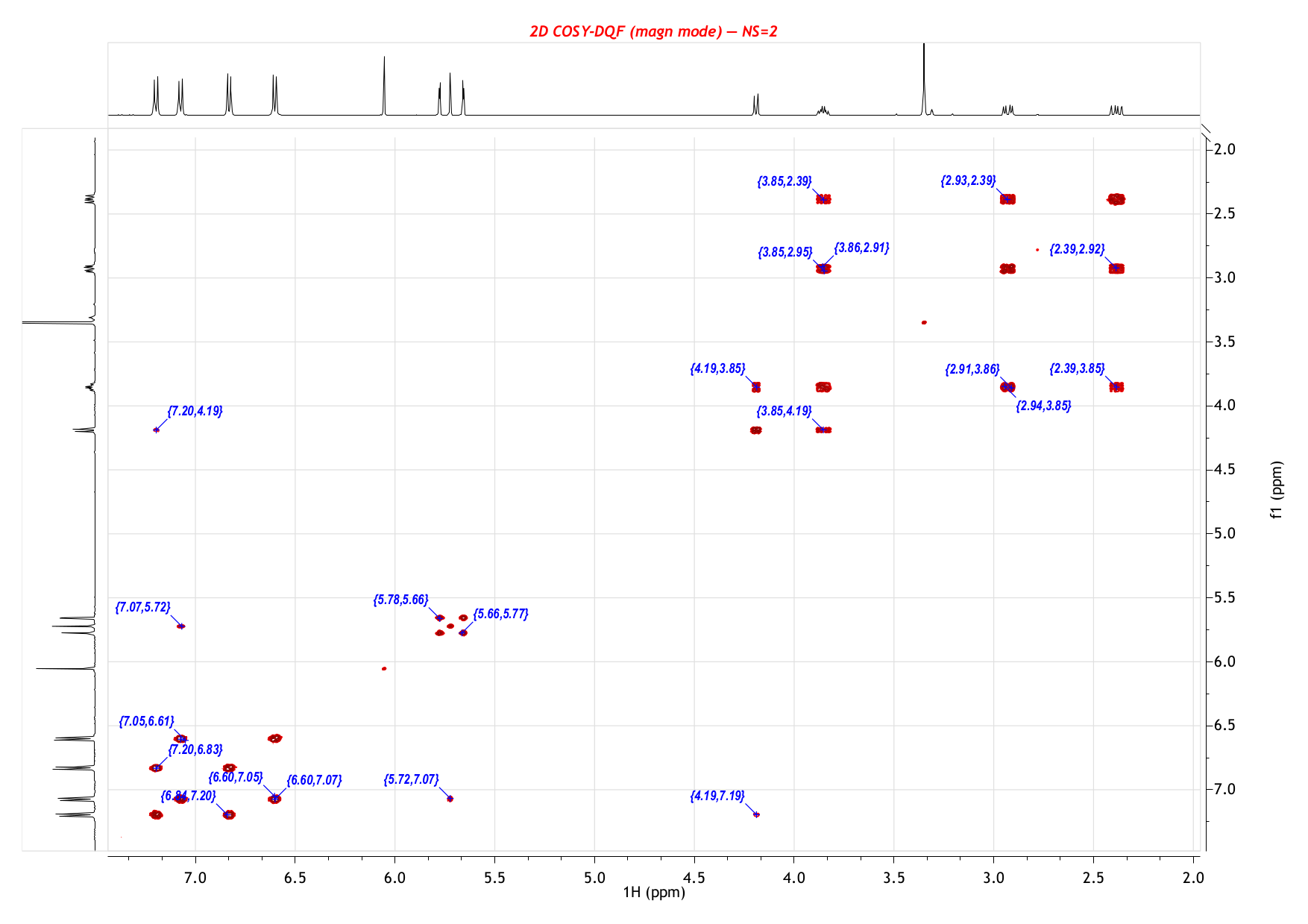


1. ^1^H-^1^H ROESY (250 ms) NMR spectrum of yuccalechin C (**29**) (500 MHz, MeOH-*d_4_*, 30 °C).


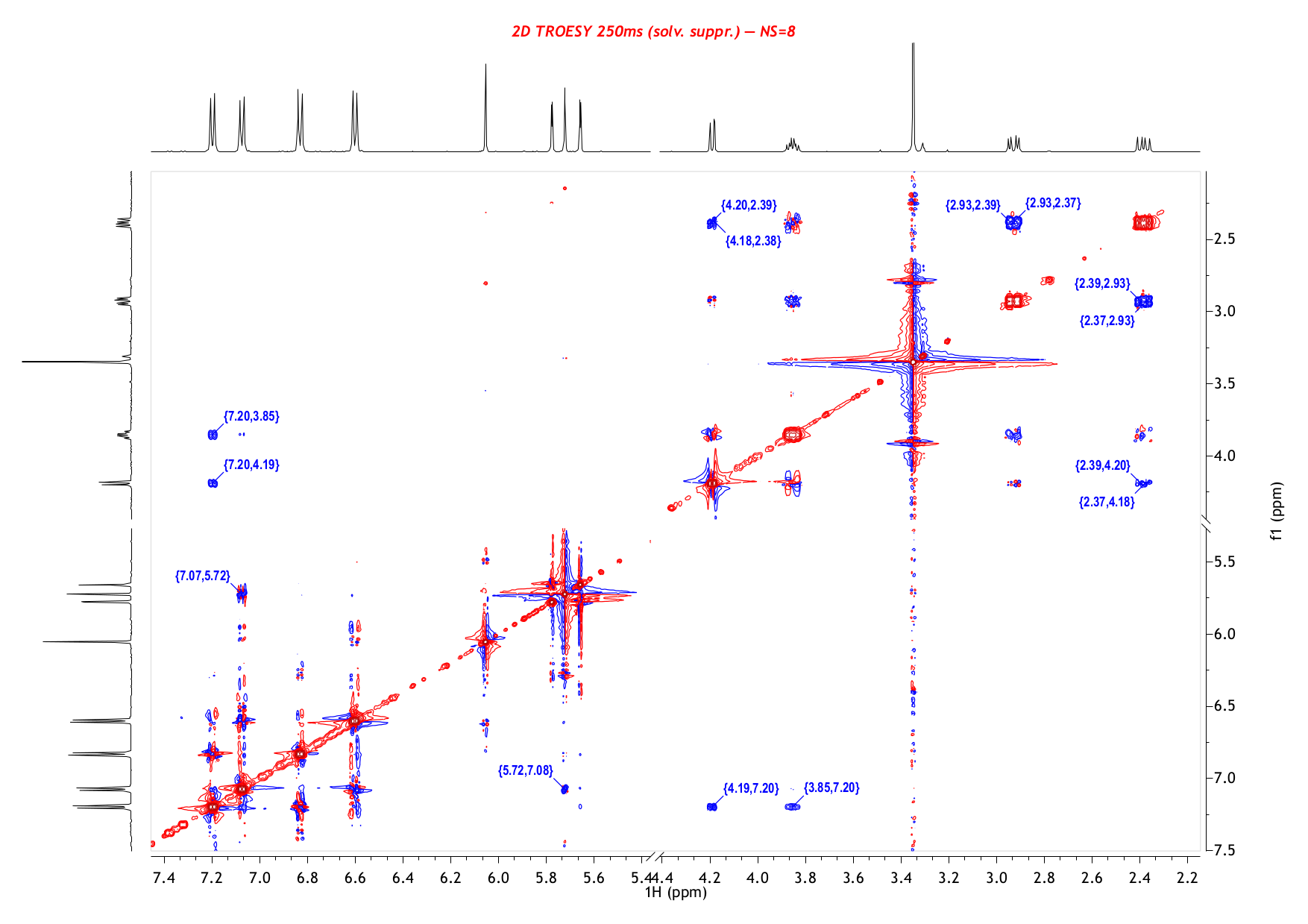


1. ^1^H-^13^C HSQC NMR spectrum of yuccalechin C (**29**) (500/125 MHz, MeOH-*d_4_*, 30 °C).


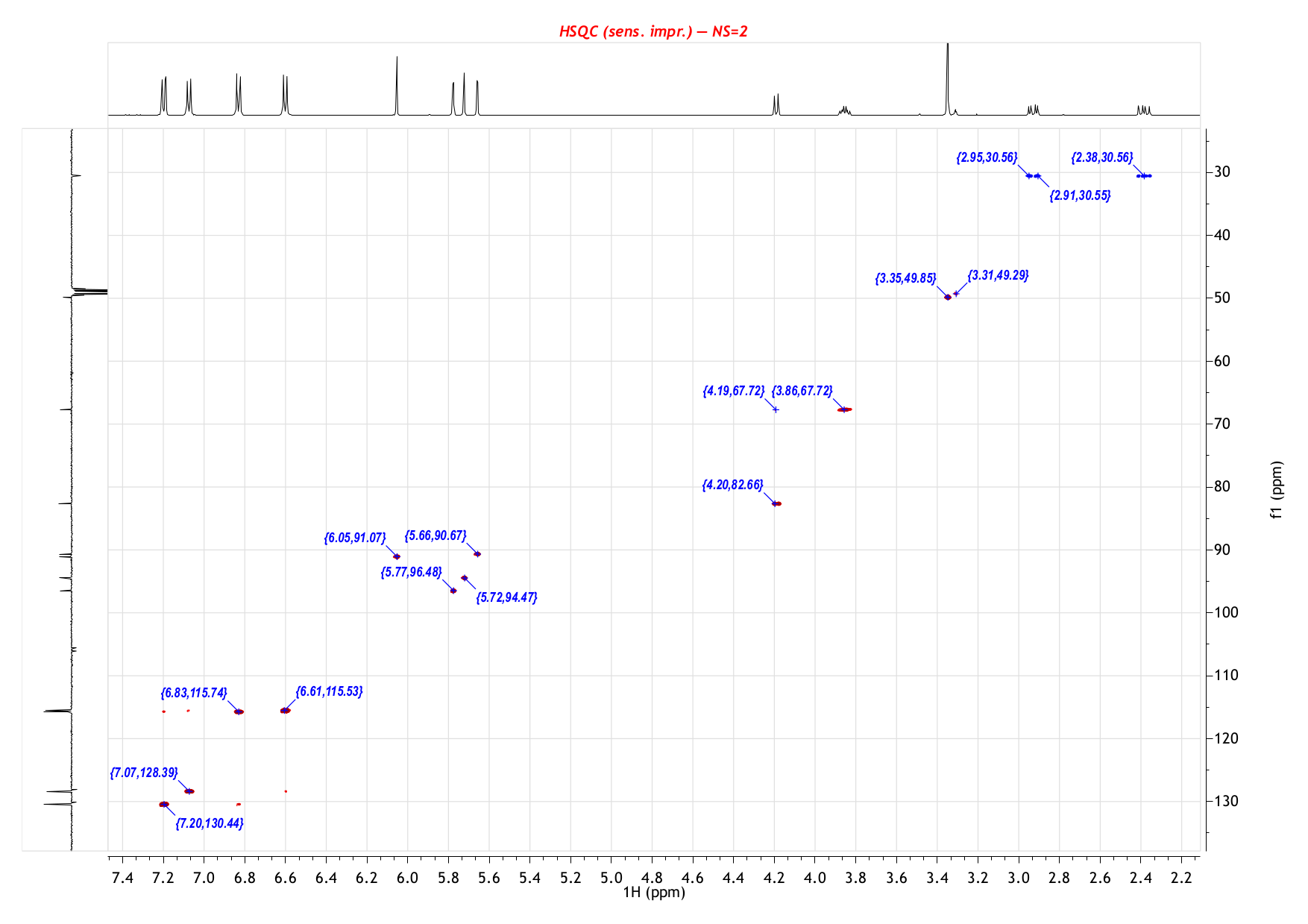


1. ^1^H-^13^C H2BC NMR spectrum of yuccalechin C (**29**) (500/125 MHz, MeOH-*d_4_*, 30 °C).


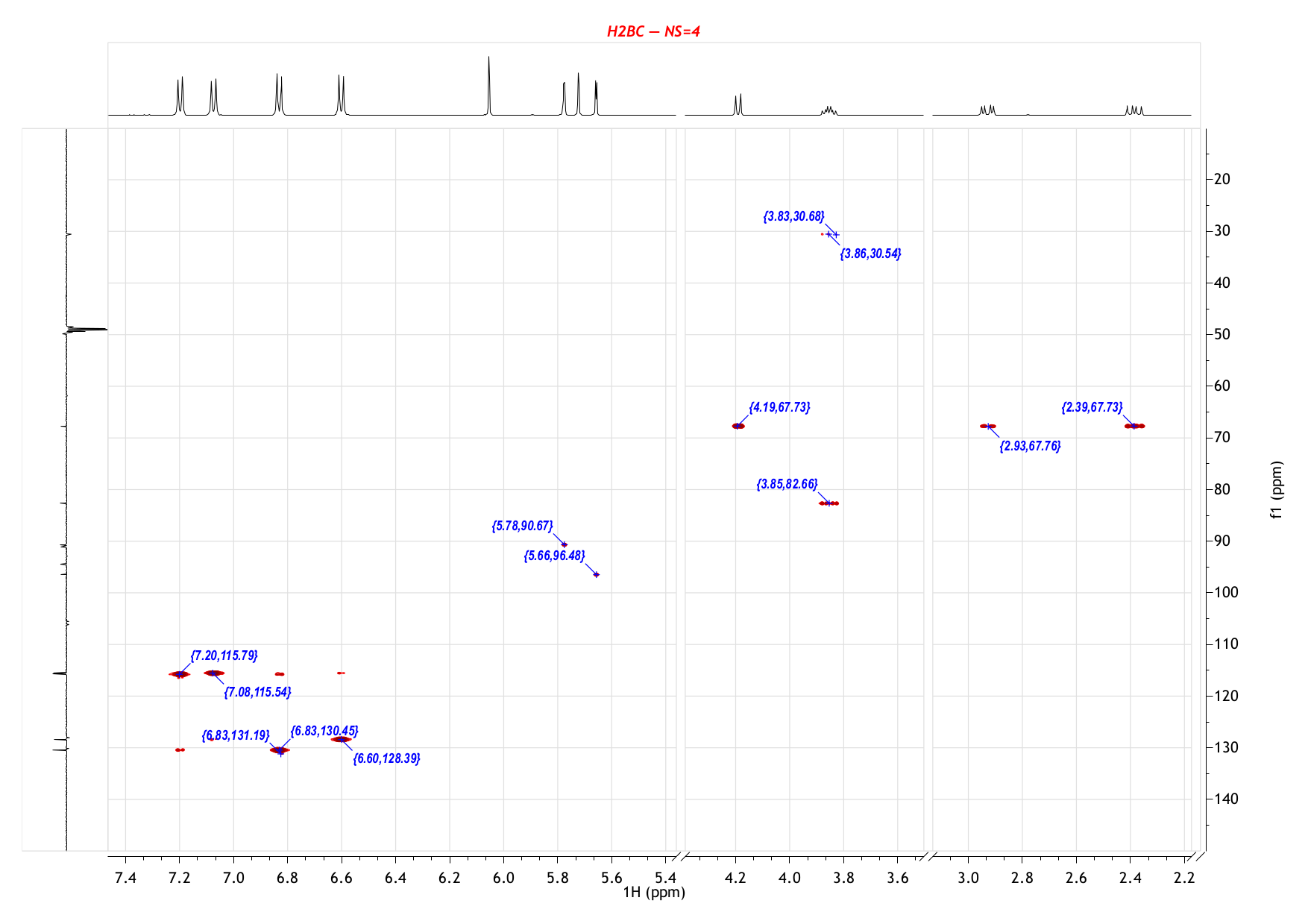


1. ^1^H-^13^C HMBC (8Hz) NMR spectrum of yuccalechin C (**29**) (500/125 MHz, MeOH-*d_4_*, 30 °C).


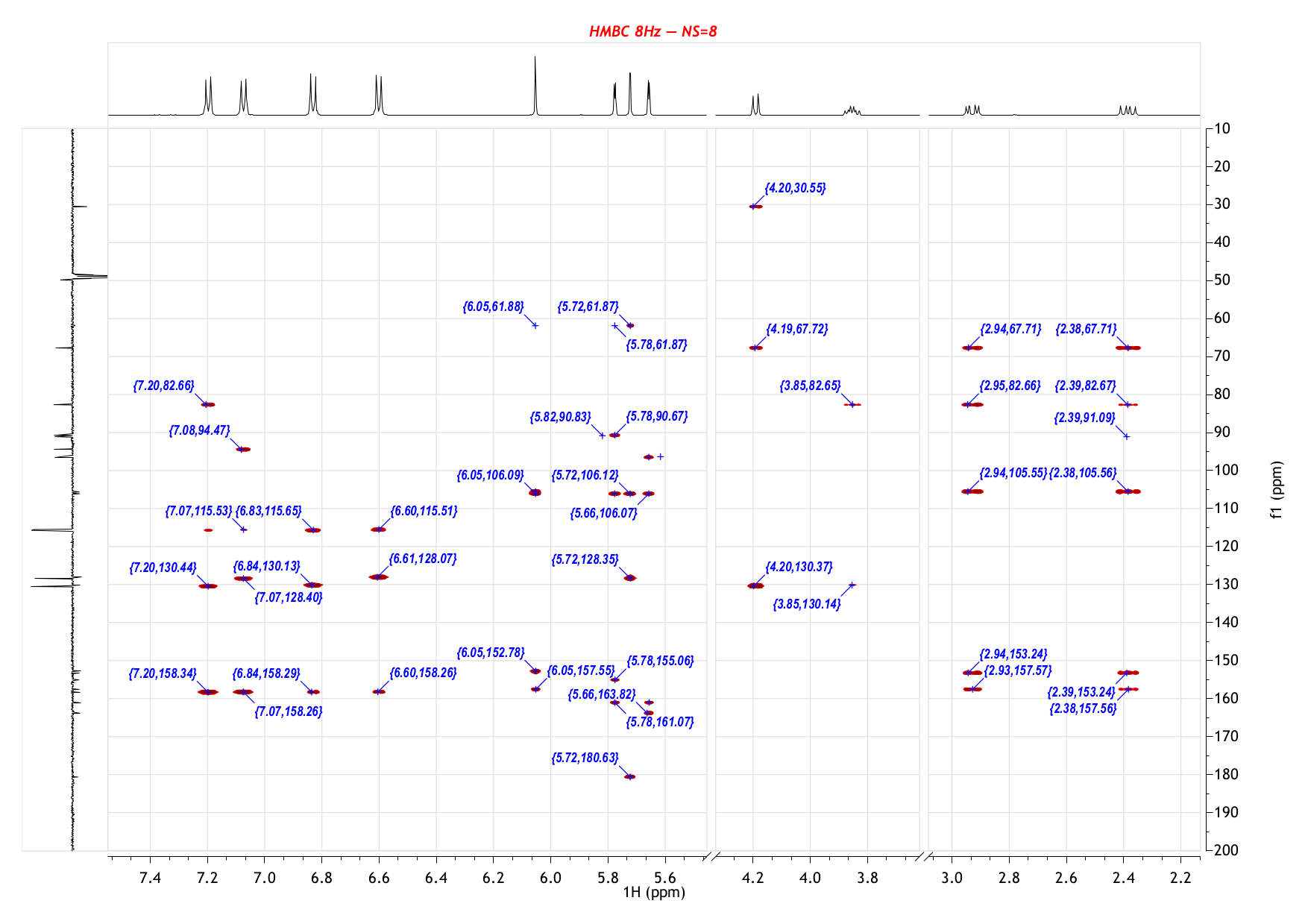


1. Optimized conformers of yuccalechin C (**29**) and their contribution to Boltzmann averaging at DFT/B3LYP/6-31G(d) level of theory in gas phase.


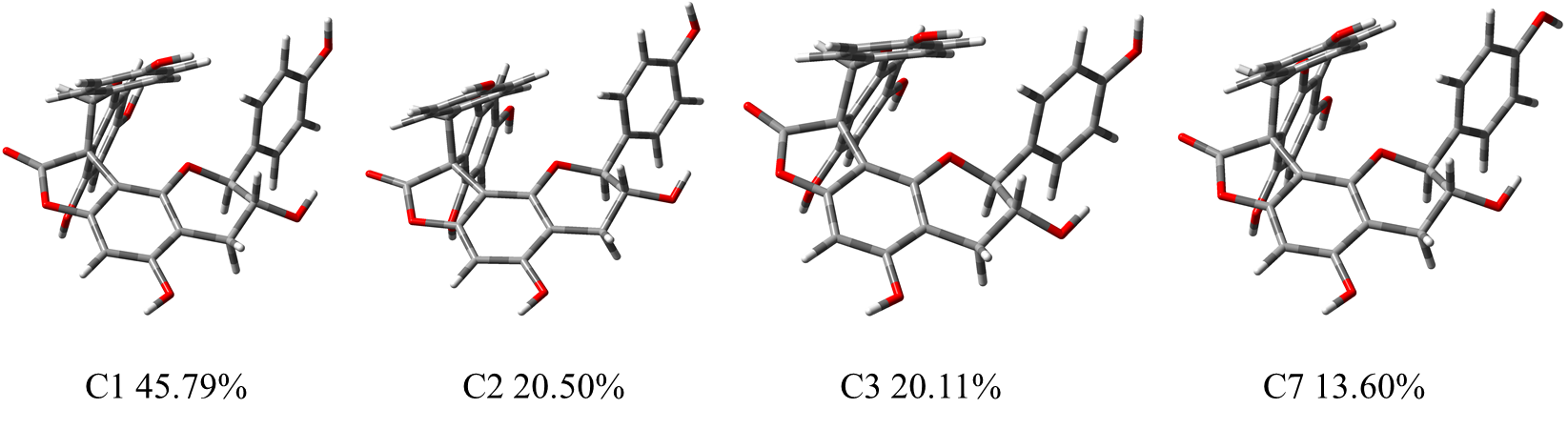


1. Calculated DP4+ probabilities of yuccalechin C (**29**) using mpw1pw91/6-111G+(d,p)/ CPCM/methanol level of theory. Isomer 1 is 2”*R*,3”*R*,2*R*,3*R* and isomer 2 is 2”*R*,3”*R*,2*R*,3*S* and isomer 3 is 2”*R*,3”*R*,2*S*,3*R*.


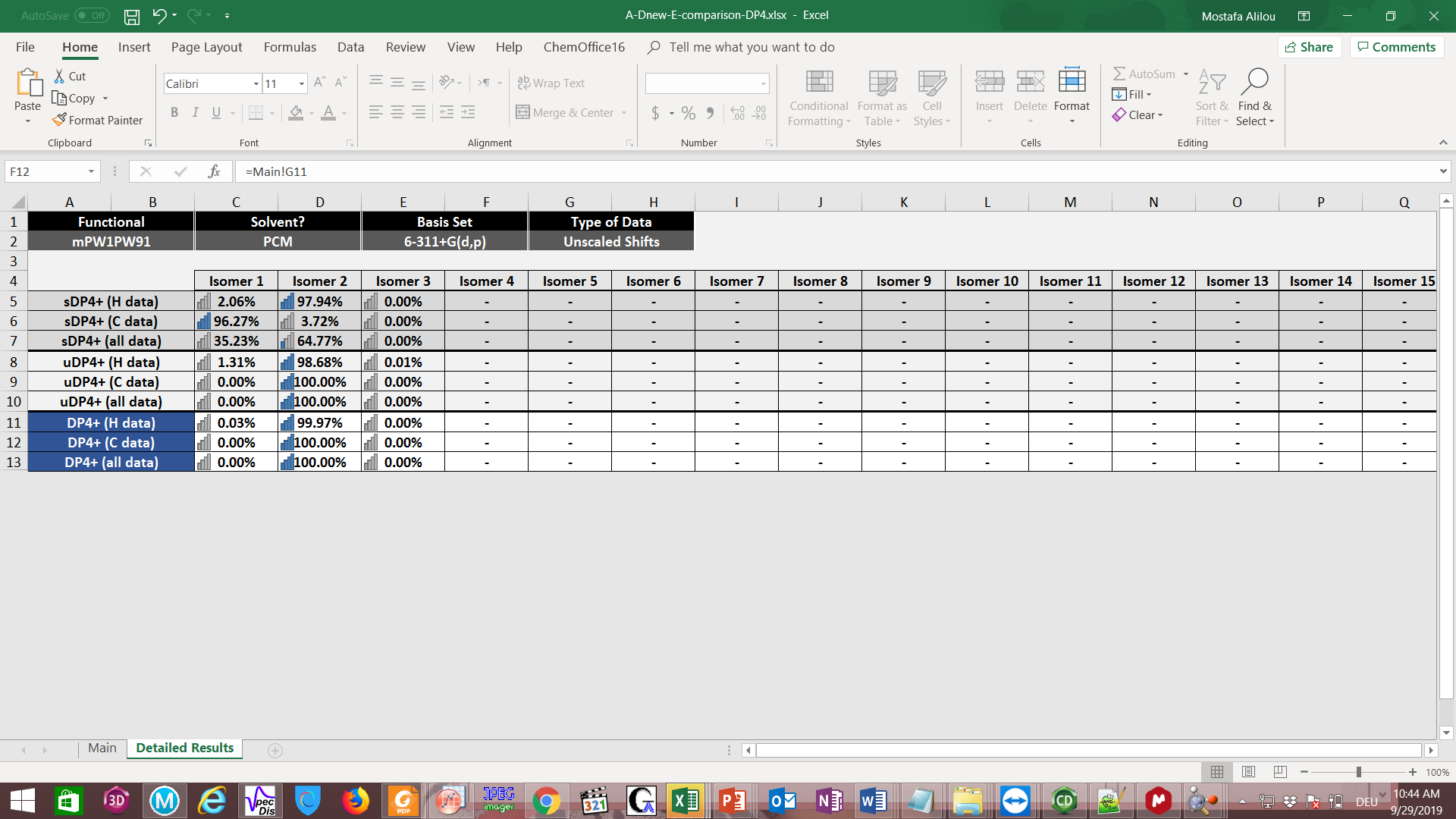


Table S3. Calculated and experimental chemical shift values used for DP4+ calculation for (**29**).

| **Atom** | **Exp.** | **Isomer 1** | **Isomer 2** | **Isomer 3** |
| --- | --- | --- | --- | --- |
| C | 161 | 160.8 | 167.5 | 167.7 |
| C | 96.5 | 95.7 | 98.6 | 99.5 |
| C | 155.1 | 154.0 | 159.4 | 160.5 |
| C | 106.1 | 105.5 | 109.6 | 109.8 |
| C | 163.8 | 163.0 | 170.5 | 171.5 |
| C | 90.7 | 91.4 | 93.5 | 94.7 |
| C | 94.5 | 95.2 | 99.7 | 99.8 |
| C | 61.9 | 64.3 | 67.3 | 68.0 |
| C | 180.6 | 181.0 | 189.5 | 189.2 |
| C | 152.8 | 151.4 | 158.6 | 159.8 |
| C | 106.1 | 105.1 | 111.7 | 110.1 |
| C | 91.1 | 91.4 | 95.3 | 95.4 |
| C | 157.6 | 157.4 | 163.4 | 164.6 |
| C | 105.6 | 105.0 | 111.4 | 107.7 |
| C | 153.2 | 154.0 | 160.4 | 157.3 |
| C | 128.1 | 128.1 | 133.7 | 134.2 |
| C | 128.4 | 129.2 | 134.1 | 134.5 |
| C | 115.5 | 114.2 | 120.1 | 120.5 |
| C | 158.3 | 157.5 | 164.8 | 165.0 |
| C | 115.5 | 114.5 | 119.5 | 119.7 |
| C | 128.4 | 128.2 | 134.1 | 134.8 |
| C | 30.5 | 29.8 | 31.7 | 26.2 |
| C | 67.7 | 67.4 | 72.2 | 71.7 |
| C | 82.7 | 80.0 | 86.7 | 85.6 |
| C | 130.1 | 130.1 | 134.5 | 136.8 |
| C | 130 | 129.2 | 138.2 | 132.2 |
| C | 115.7 | 115.4 | 120.2 | 119.8 |
| C | 158.3 | 157.2 | 165.0 | 163.3 |
| C | 115.7 | 114.6 | 120.8 | 119.6 |
| C | 130 | 129.4 | 136.6 | 132.0 |
| H | 5.78 | 6.01 | 5.71 | 5.97 |
| H | 5.66 | 6.08 | 5.71 | 6.22 |
| H | 5.72 | 5.99 | 6.14 | 6.20 |
| H | 6.05 | 6.24 | 6.32 | 6.30 |
| H | 7.07 | 7.43 | 7.80 | 7.33 |
| H | 6.6 | 6.69 | 7.15 | 7.06 |
| H | 6.6 | 6.75 | 7.09 | 7.04 |
| H | 7.07 | 6.95 | 7.98 | 8.05 |
| H | 2.39 | 2.71 | 2.33 | 2.03 |
| H | 2.93 | 2.61 | 2.96 | 2.58 |
| H | 3.85 | 3.74 | 3.79 | 4.14 |
| H | 4.19 | 4.76 | 3.92 | 5.39 |
| H | 7.2 | 7.18 | 7.57 | 7.09 |
| H | 6.83 | 7.03 | 7.19 | 6.98 |
| H | 6.83 | 6.90 | 7.29 | 6.94 |
| H | 7.2 | 6.80 | 8.15 | 6.97 |

1.
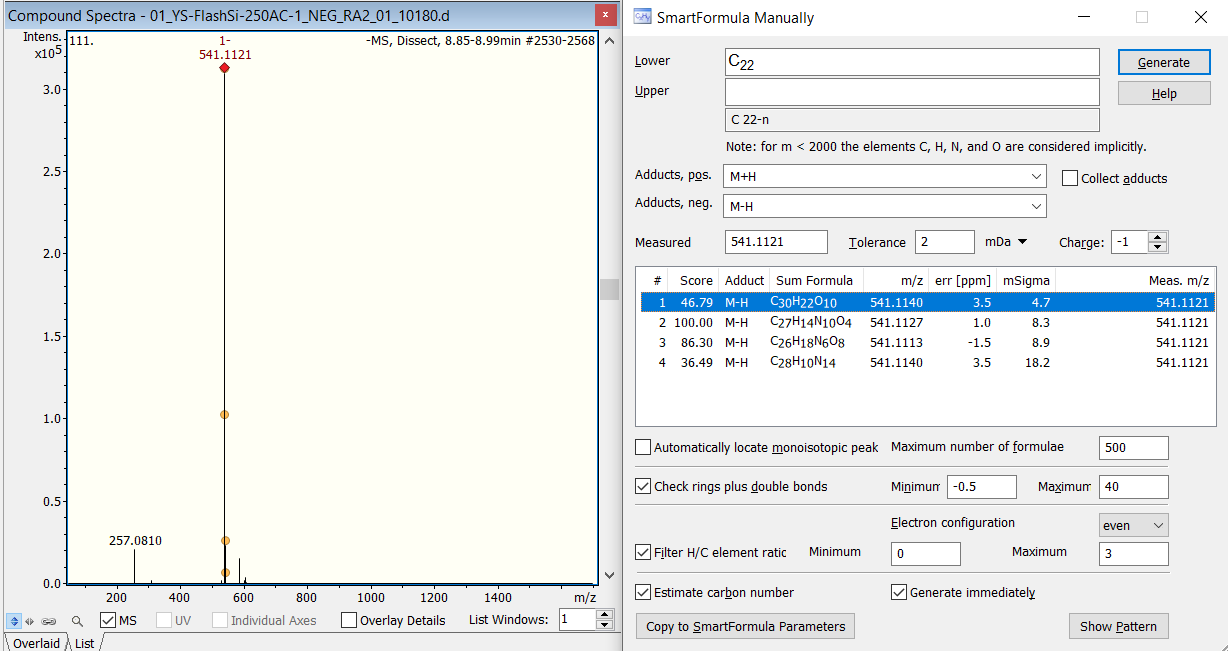
HRESIMS (Q-TOF) analysis of yuccalechin C (**29**) in negative ion mode.
2. ^1^H NMR spectrum of yuccaol E (**37**) (500 MHz, MeOH-*d_4_*, 30 °C).


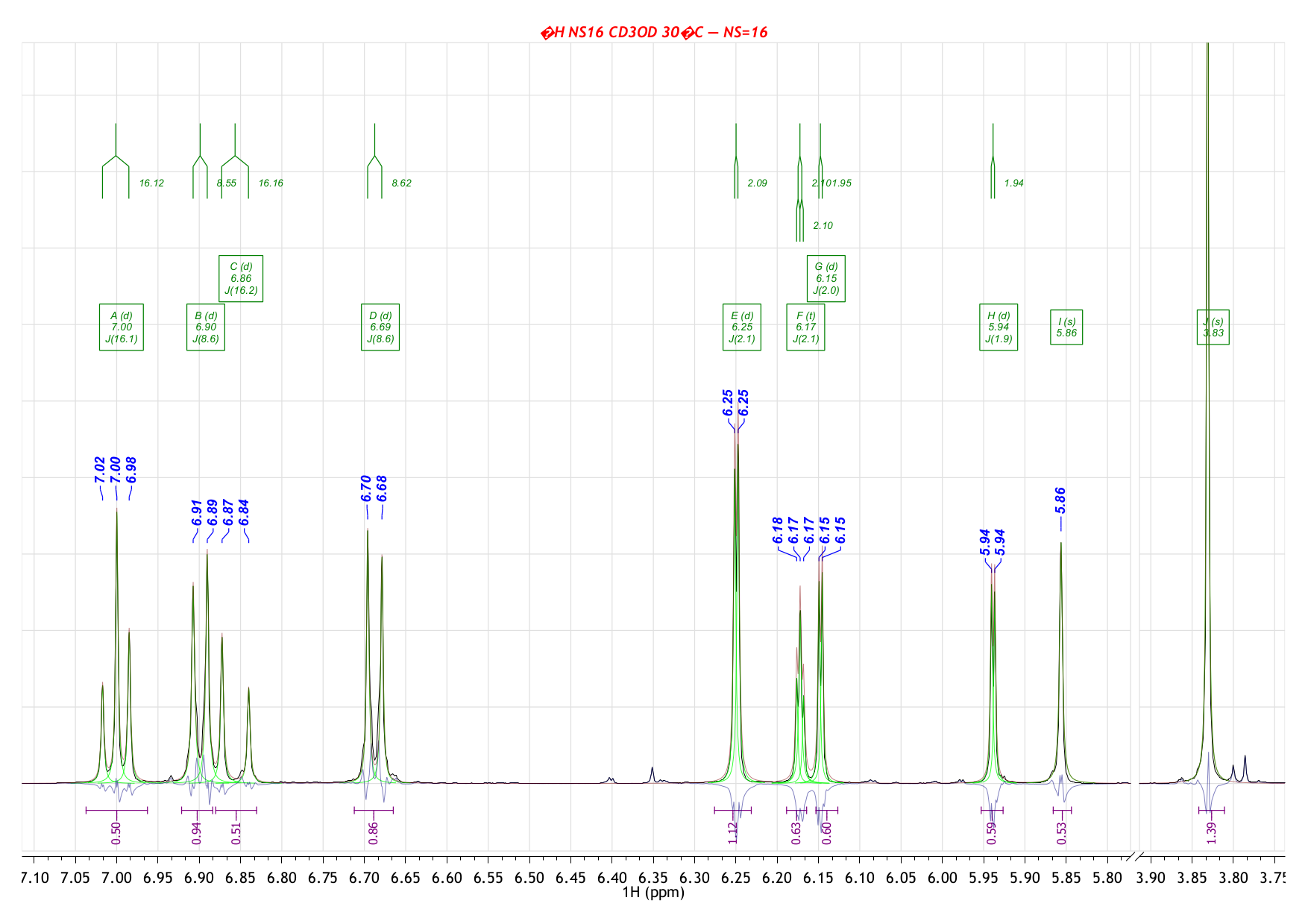


1. ^13^C NMR spectrum of yuccaol E (**37**) (125 MHz, MeOH-*d_4_*, 30 °C).


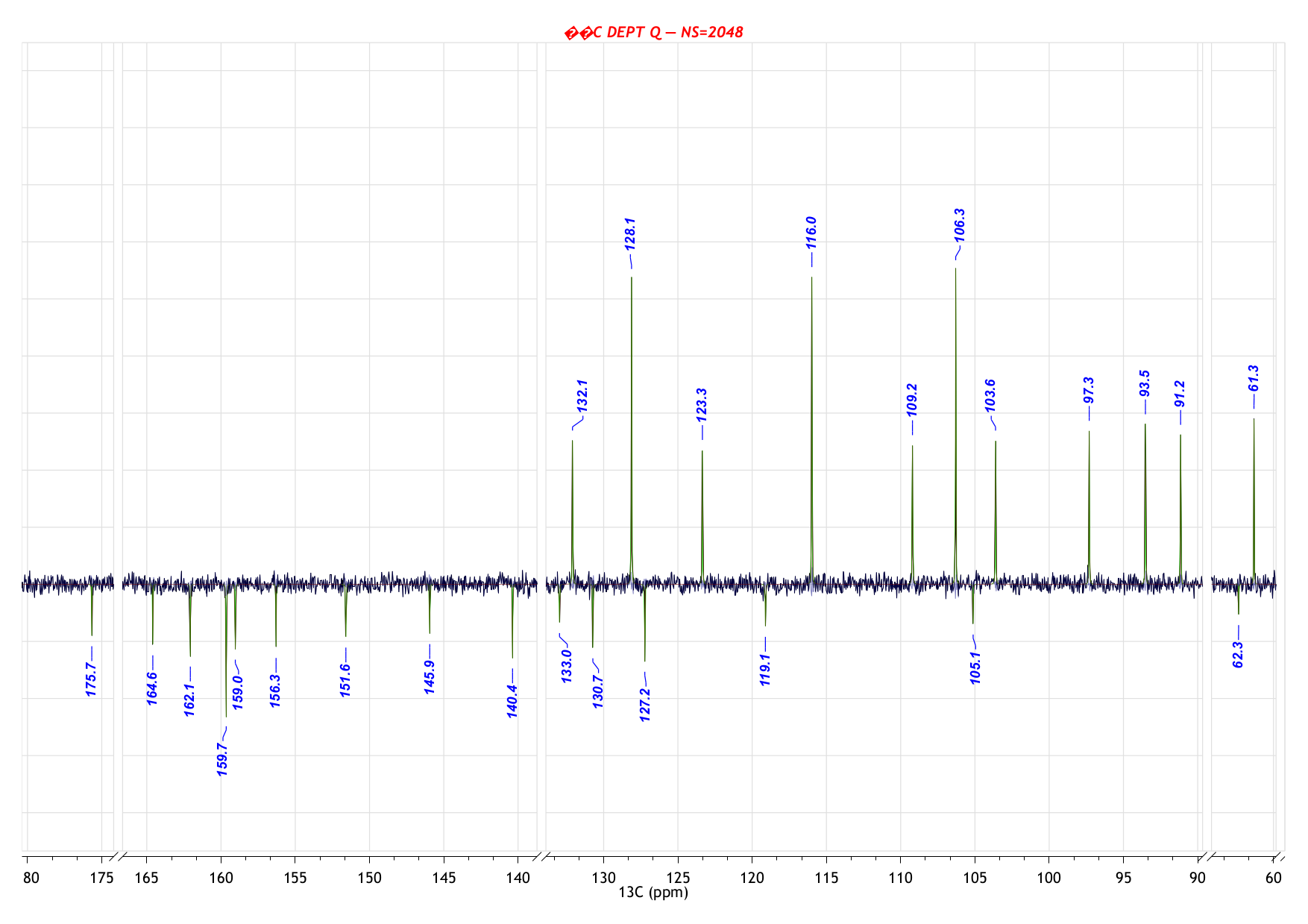


1. ^1^H NMR spectrum of naringenin (**38**) (500 MHz, MeOH-*d_4_*, 30 °C).


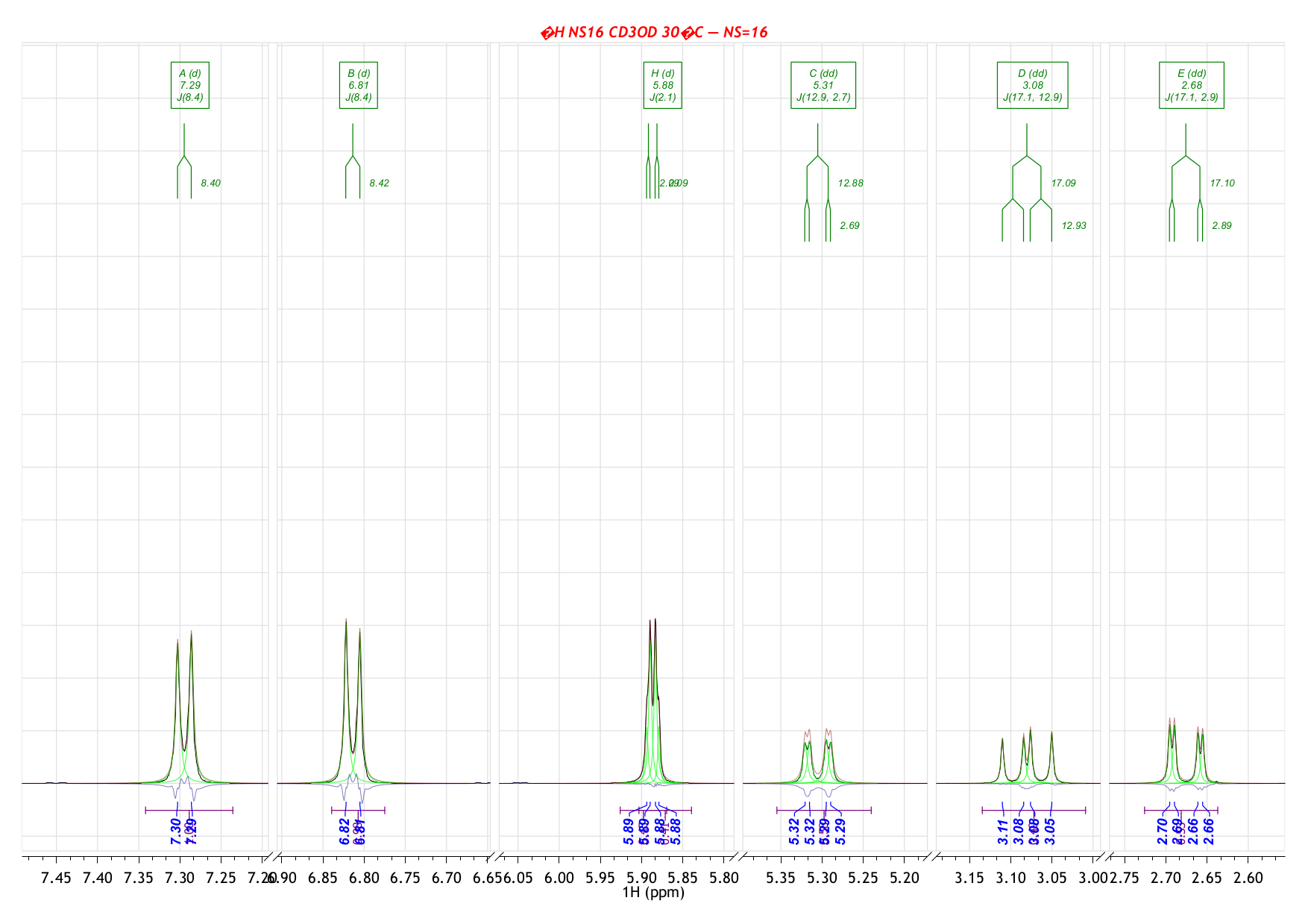


1. ^13^C NMR spectrum of naringenin (**38**) (125 MHz, MeOH-*d_4_*, 30 °C).


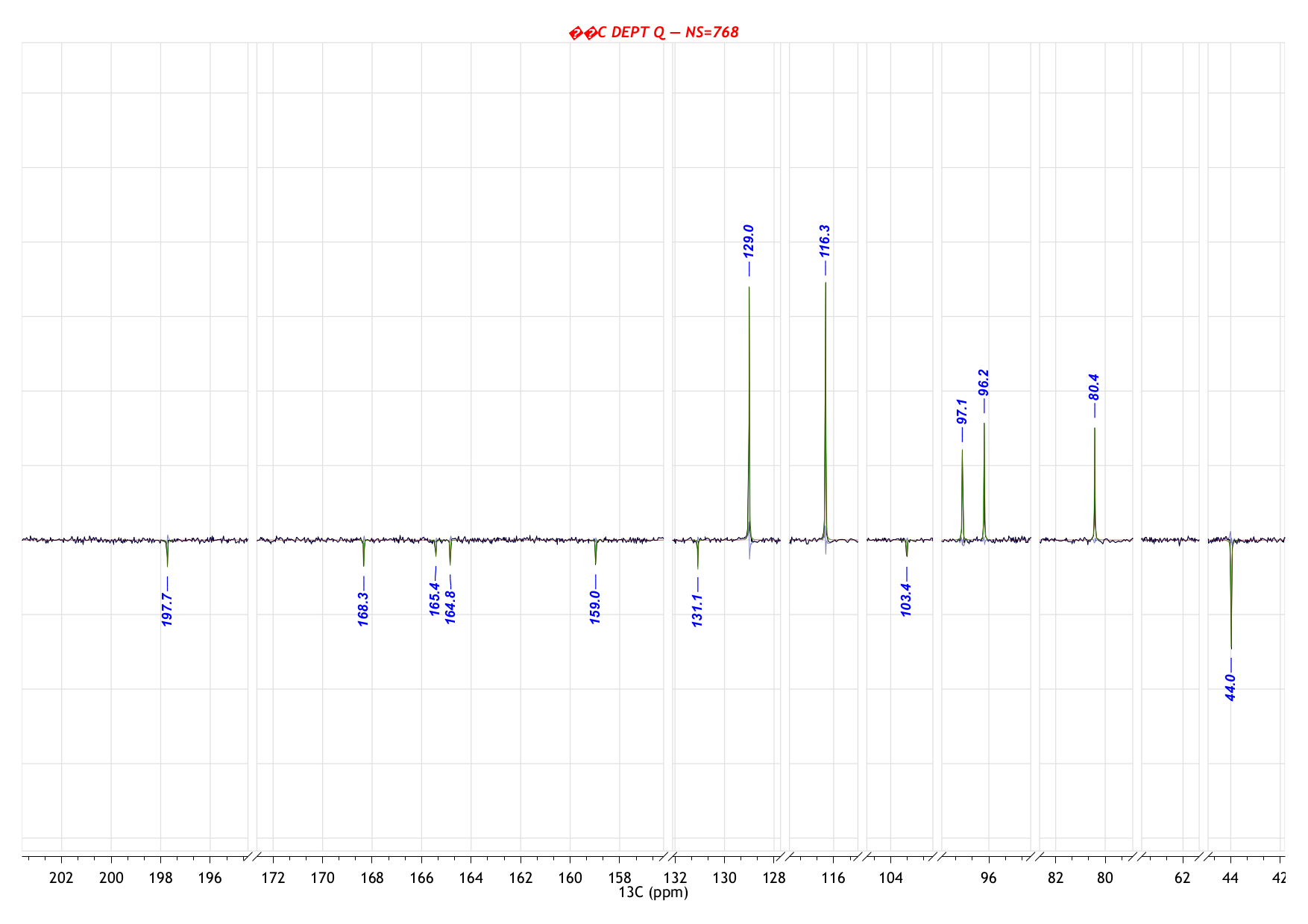


1. ^1^H NMR spectrum of yuccaol C (**39**) (500 MHz, MeOH-*d_4_*, 30 °C).


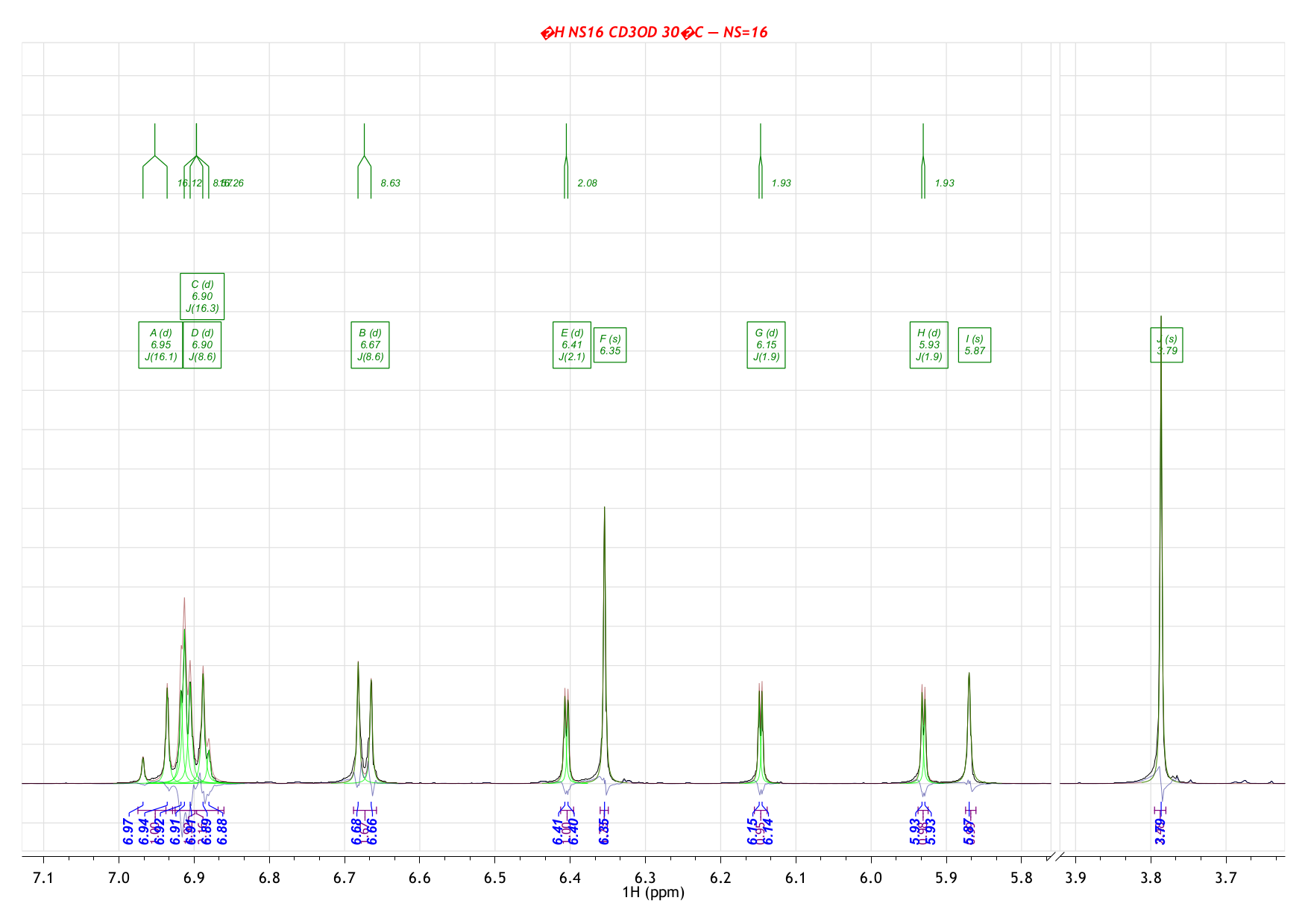


1. ^13^C NMR spectrum of yuccaol C (**39**) (125 MHz, MeOH-*d_4_*, 30 °C).


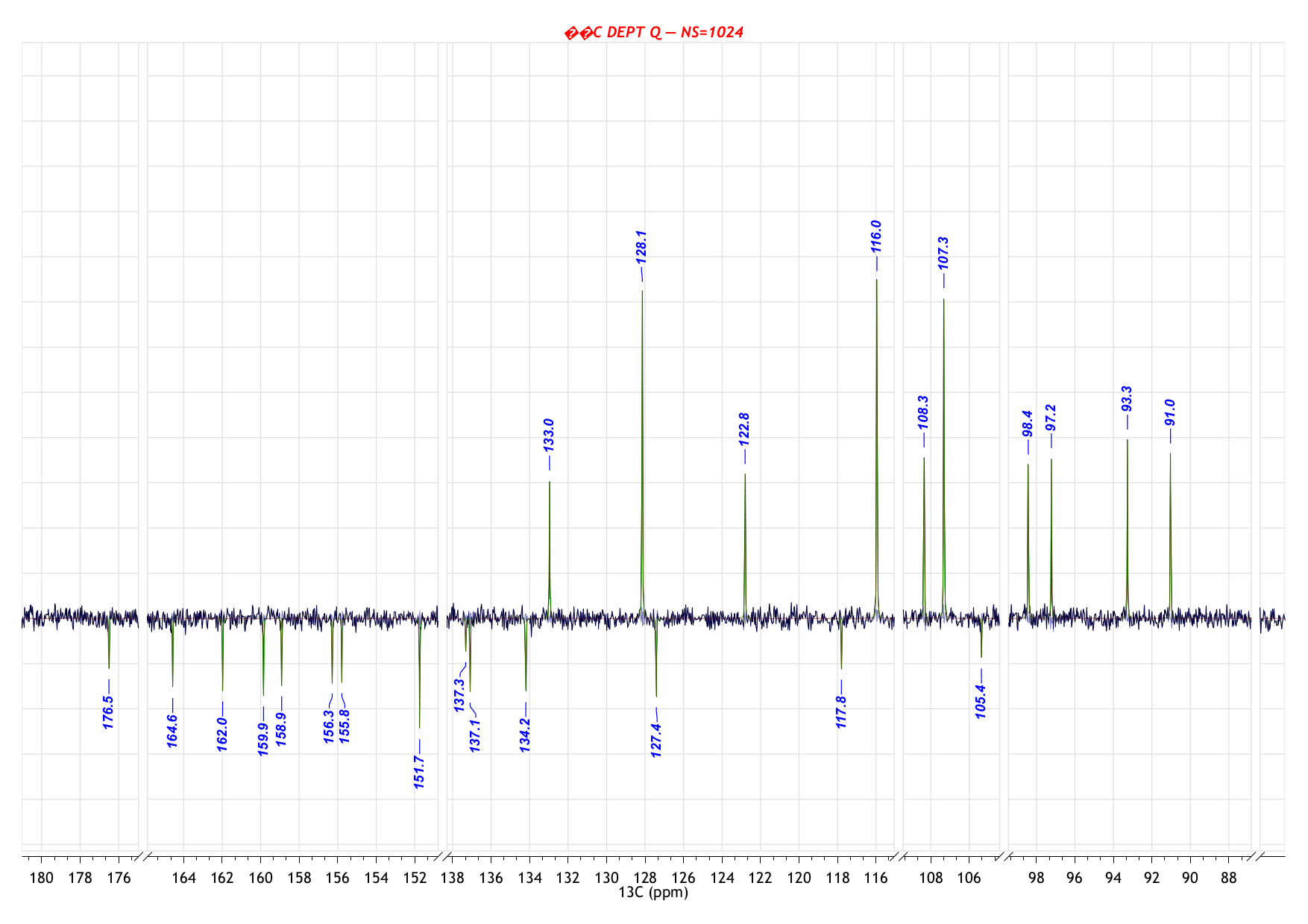


1. ^1^H NMR spectrum of yuccalide A (**40**) (500 MHz, MeOH-*d_4_*, 30 °C).


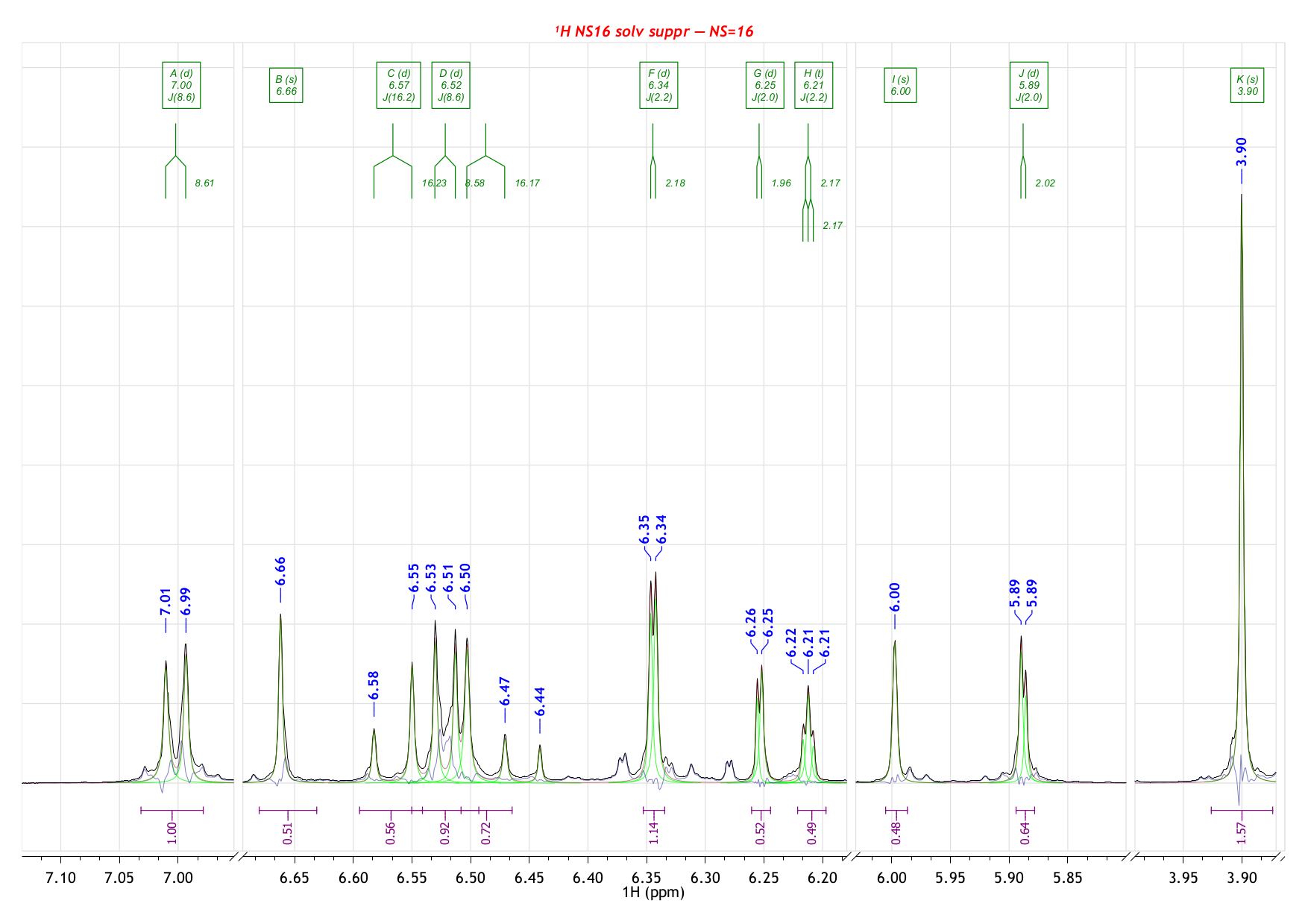


1. ^13^C NMR spectrum of yuccalide A (**40**) (125 MHz, MeOH-*d_4_*, 30 °C).


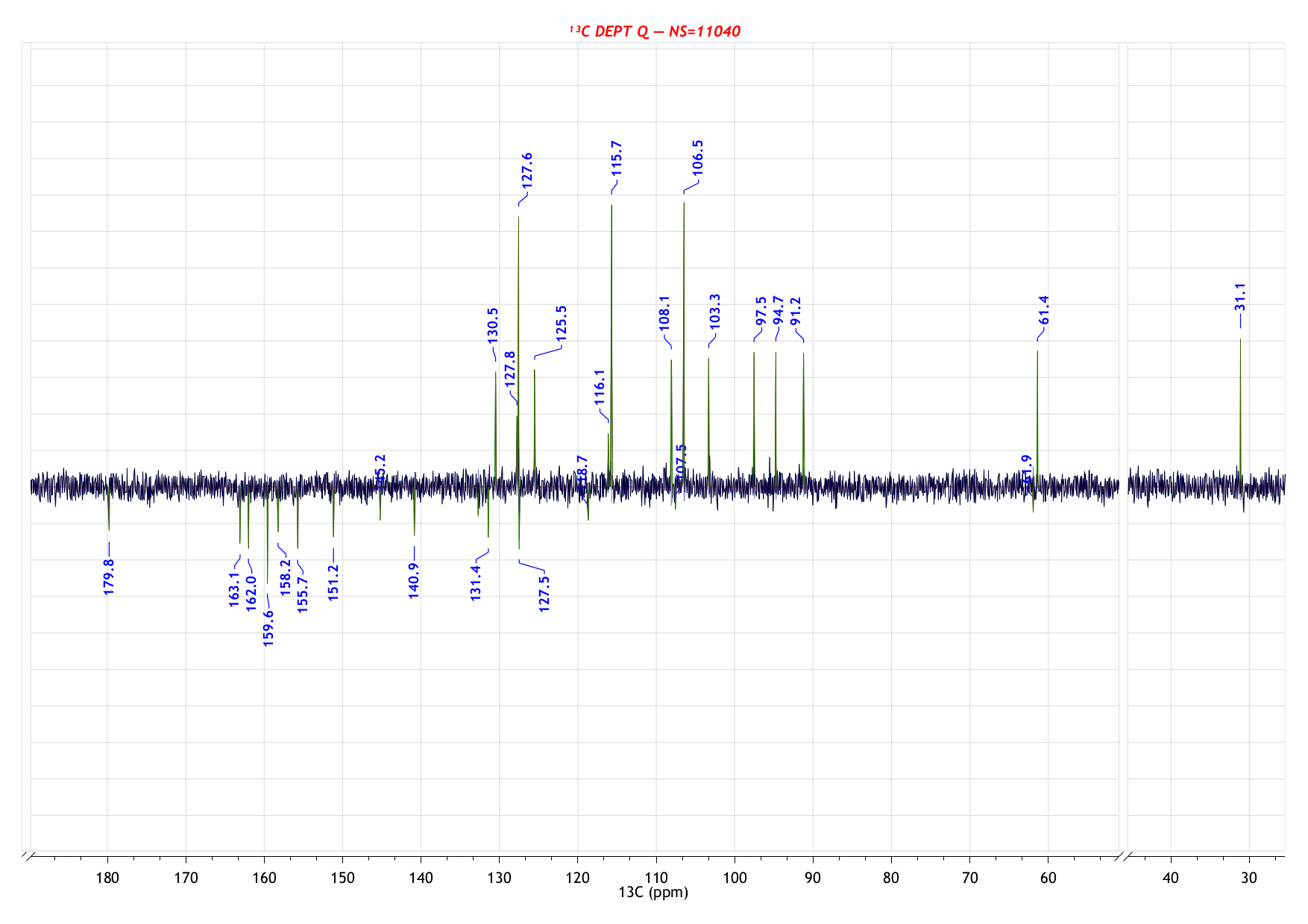


1. ^1^H NMR spectrum of yuccaol D (**42**) (500 MHz, MeOH-*d_4_*, 30 °C).


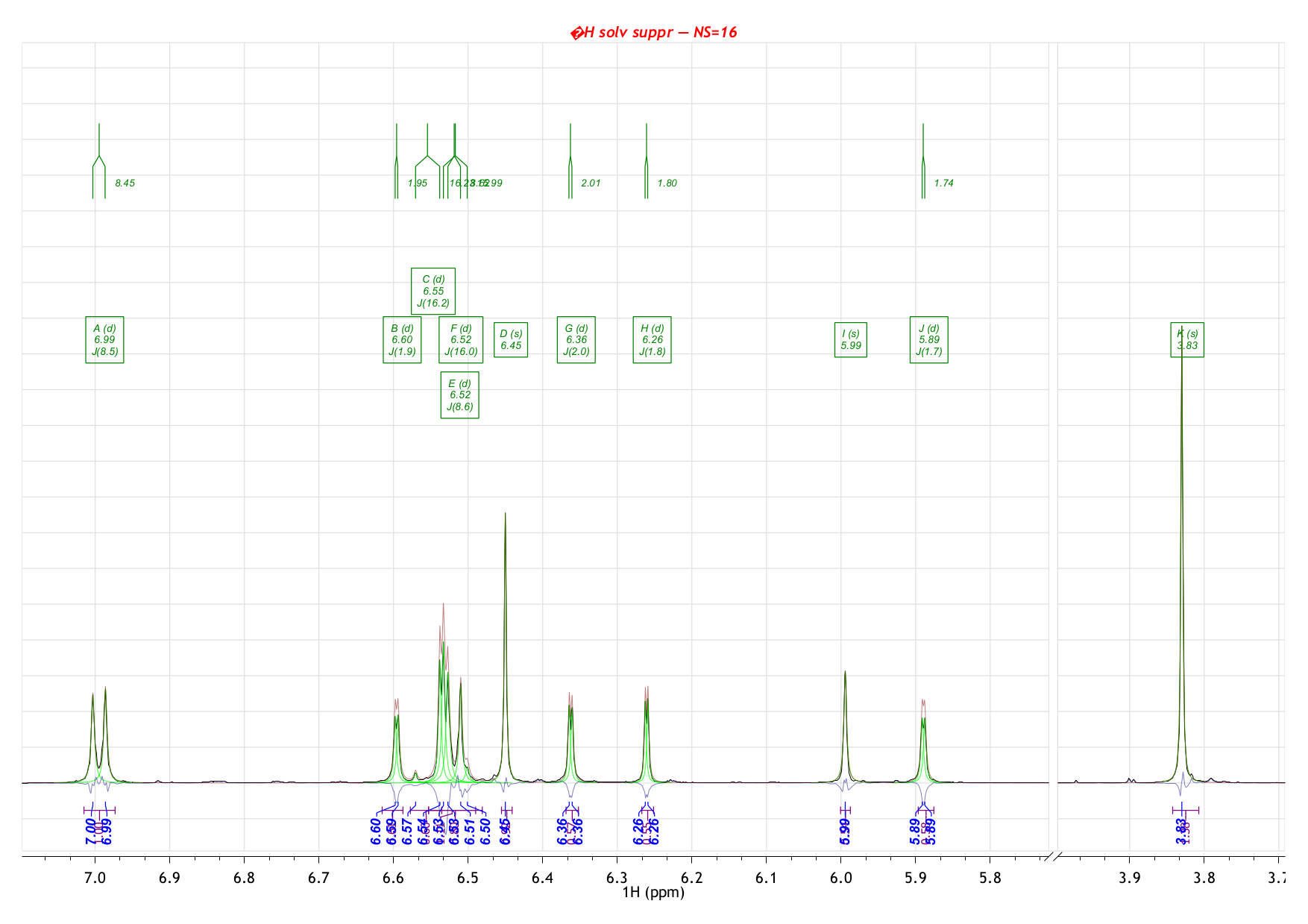


1. ^13^C NMR spectrum of yuccaol D (**42**) (125 MHz, MeOH-*d_4_*, 30 °C).


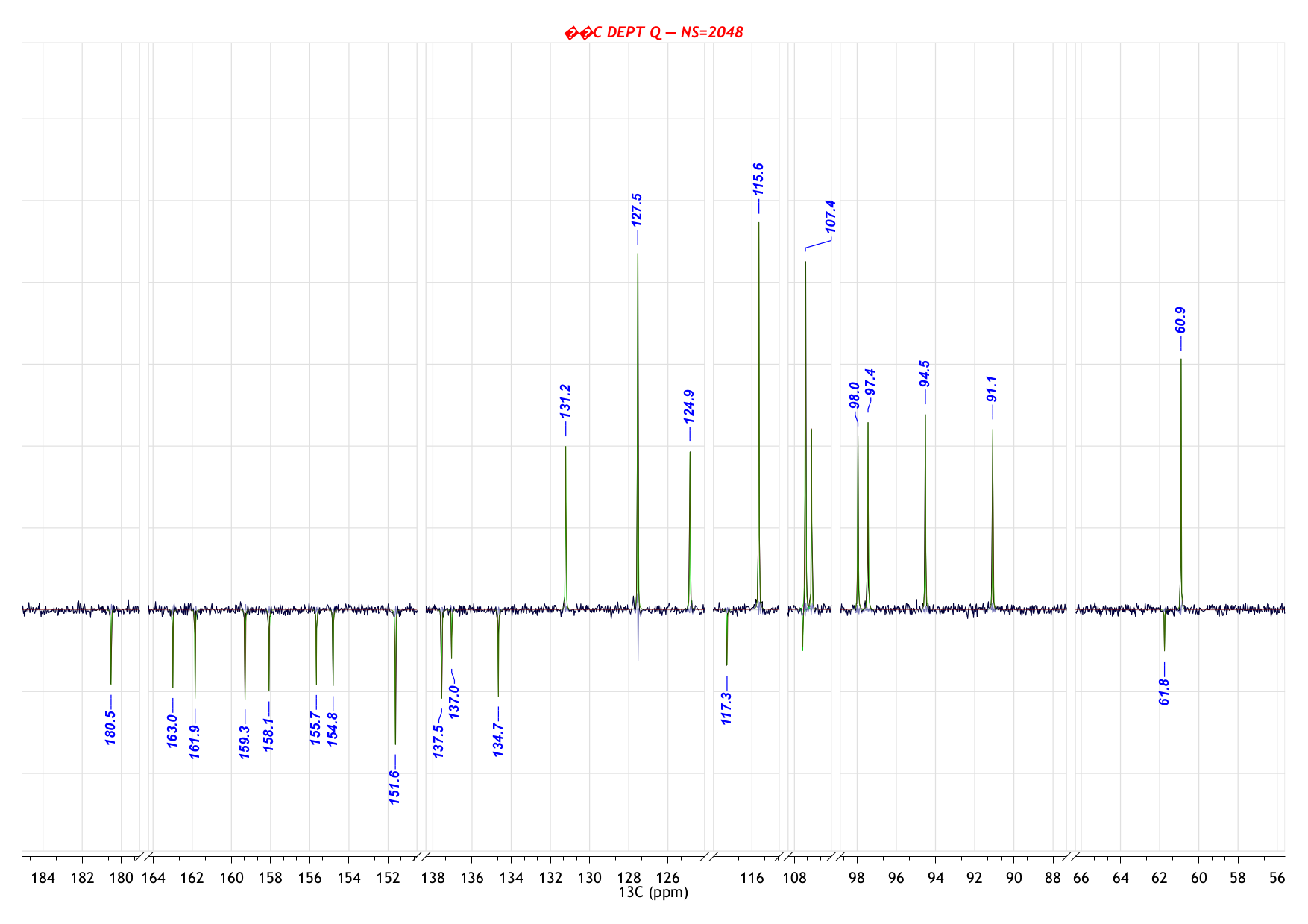


1. ^1^H NMR spectrum of kaempferol (**44**) (500 MHz, MeOH-*d_4_*, 30 °C).


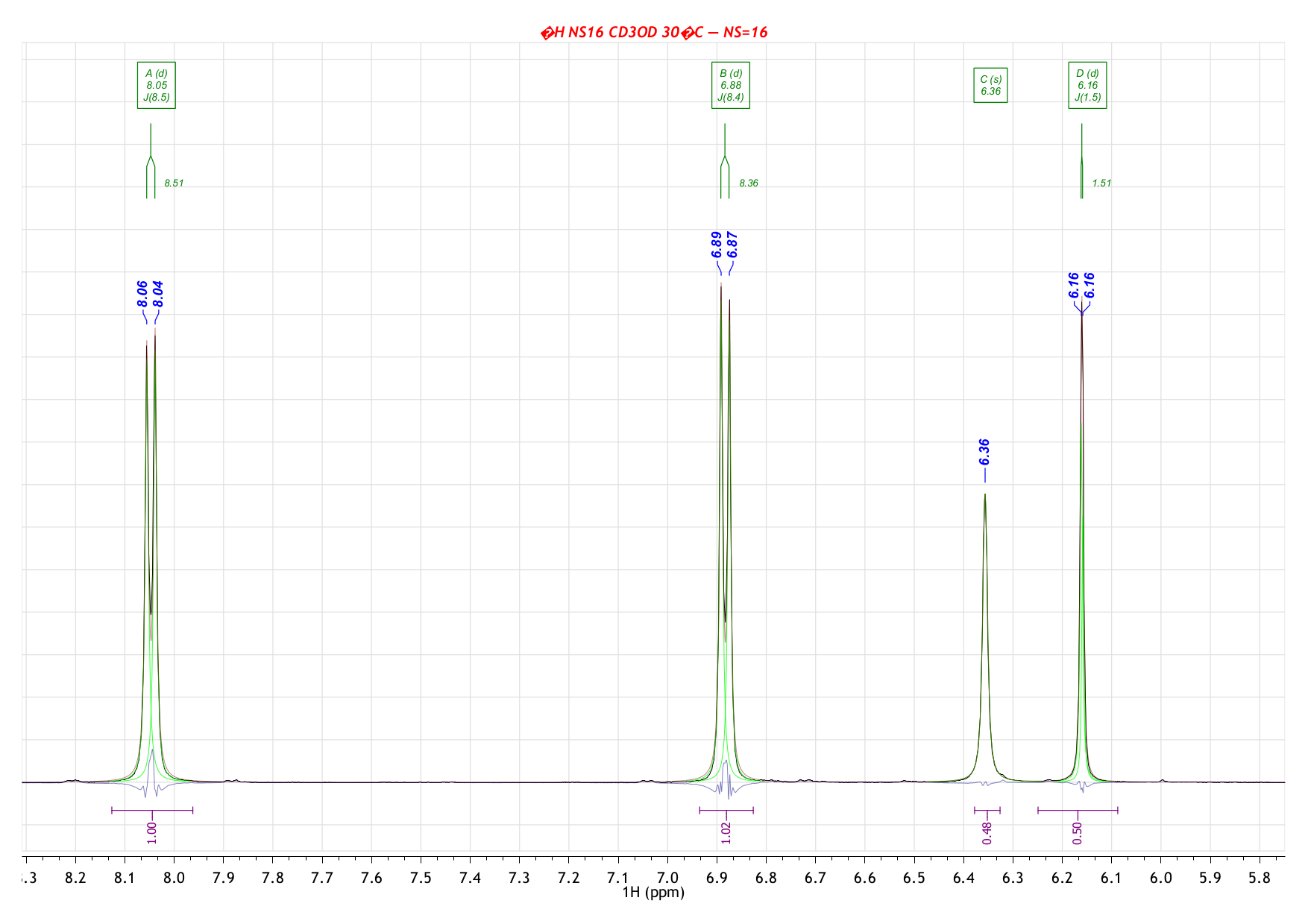


1. ^13^C NMR spectrum of kaempferol (**44**) (125 MHz, MeOH-*d_4_*, 30 °C).


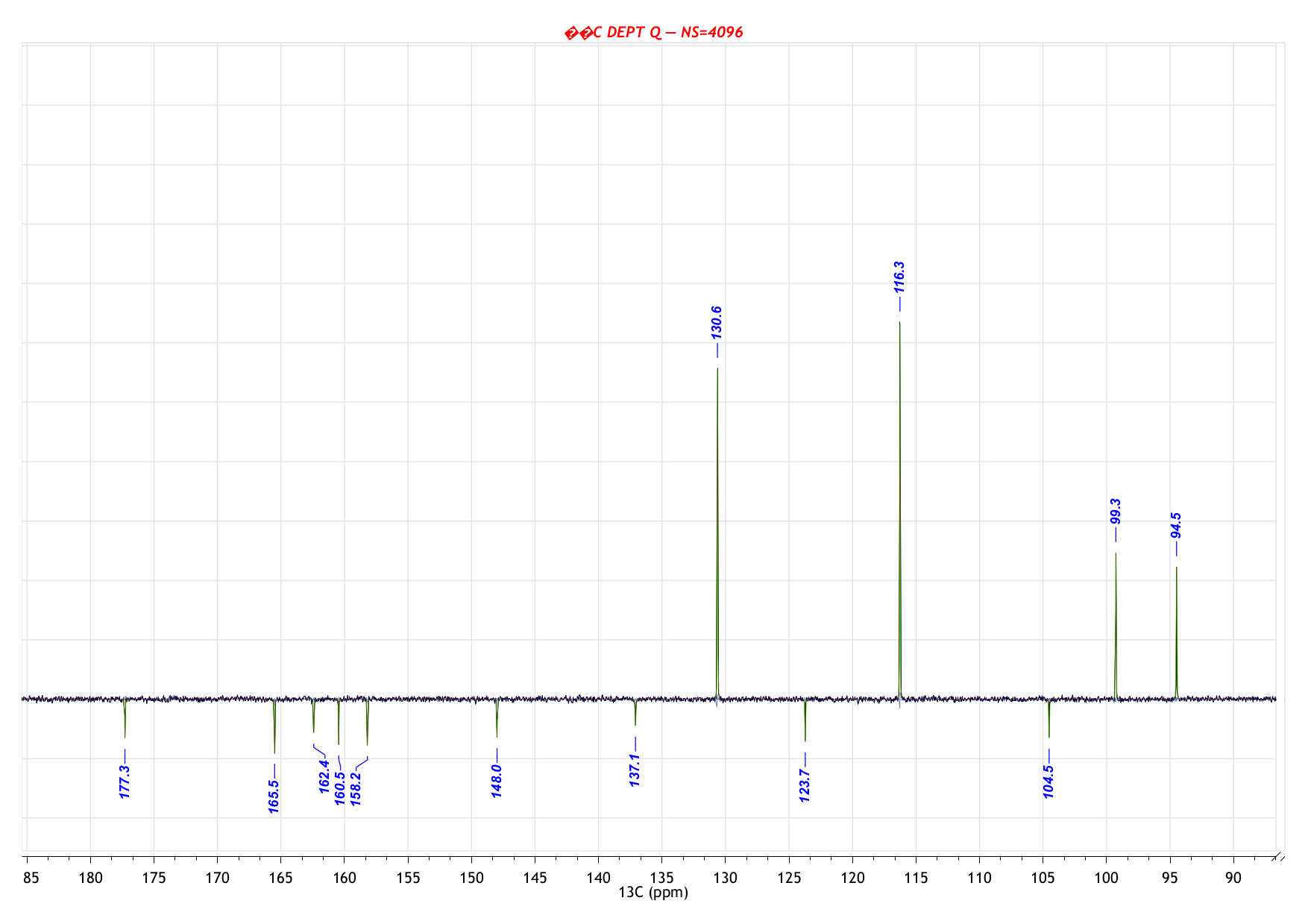


1. ^1^H NMR spectrum of yuccaol A (**47**) (500 MHz, MeOH-*d_4_*, 30 °C).


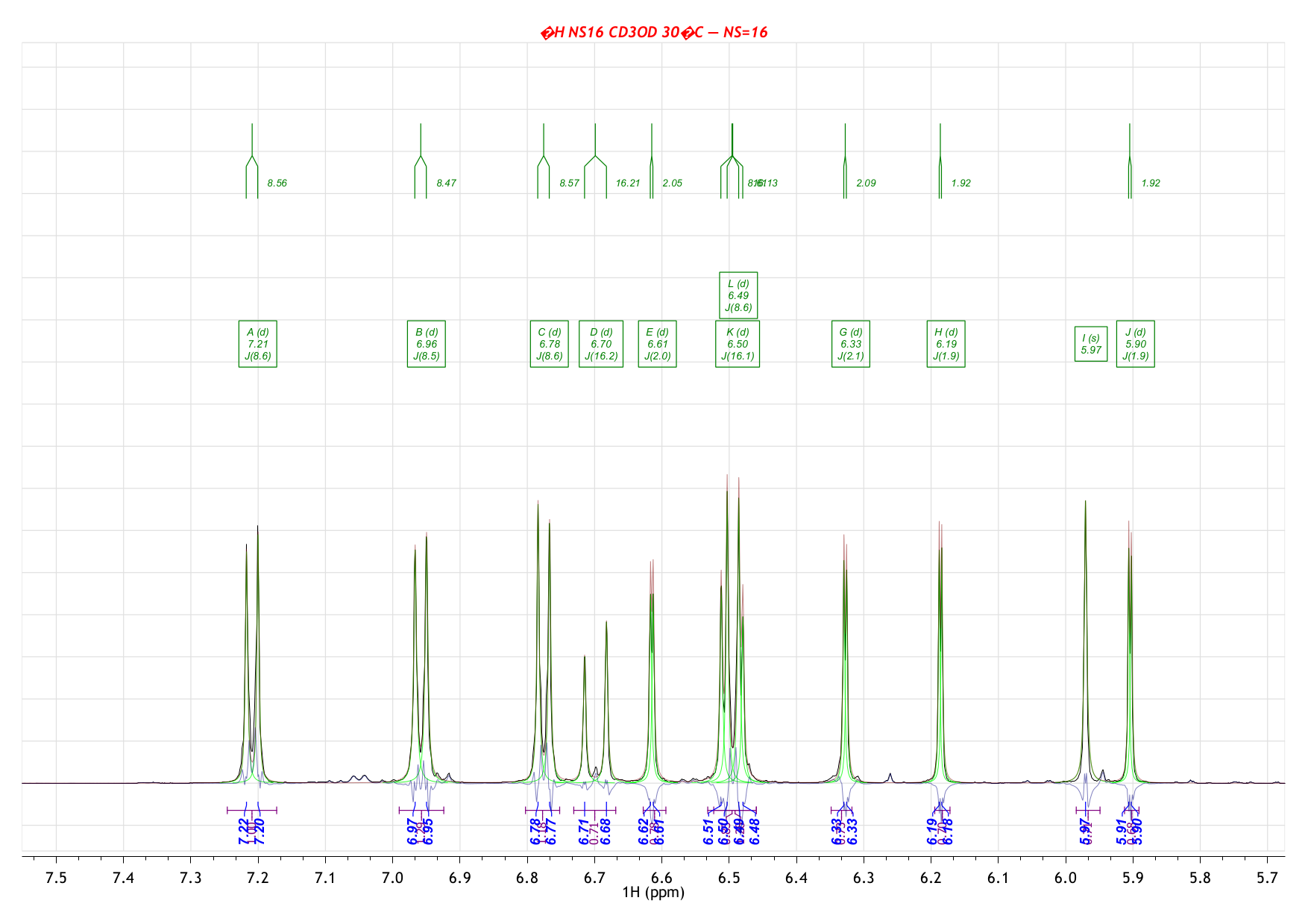


1. ^13^C NMR spectrum of yuccaol A (**47**) (125 MHz, MeOH-*d_4_*, 30 °C).


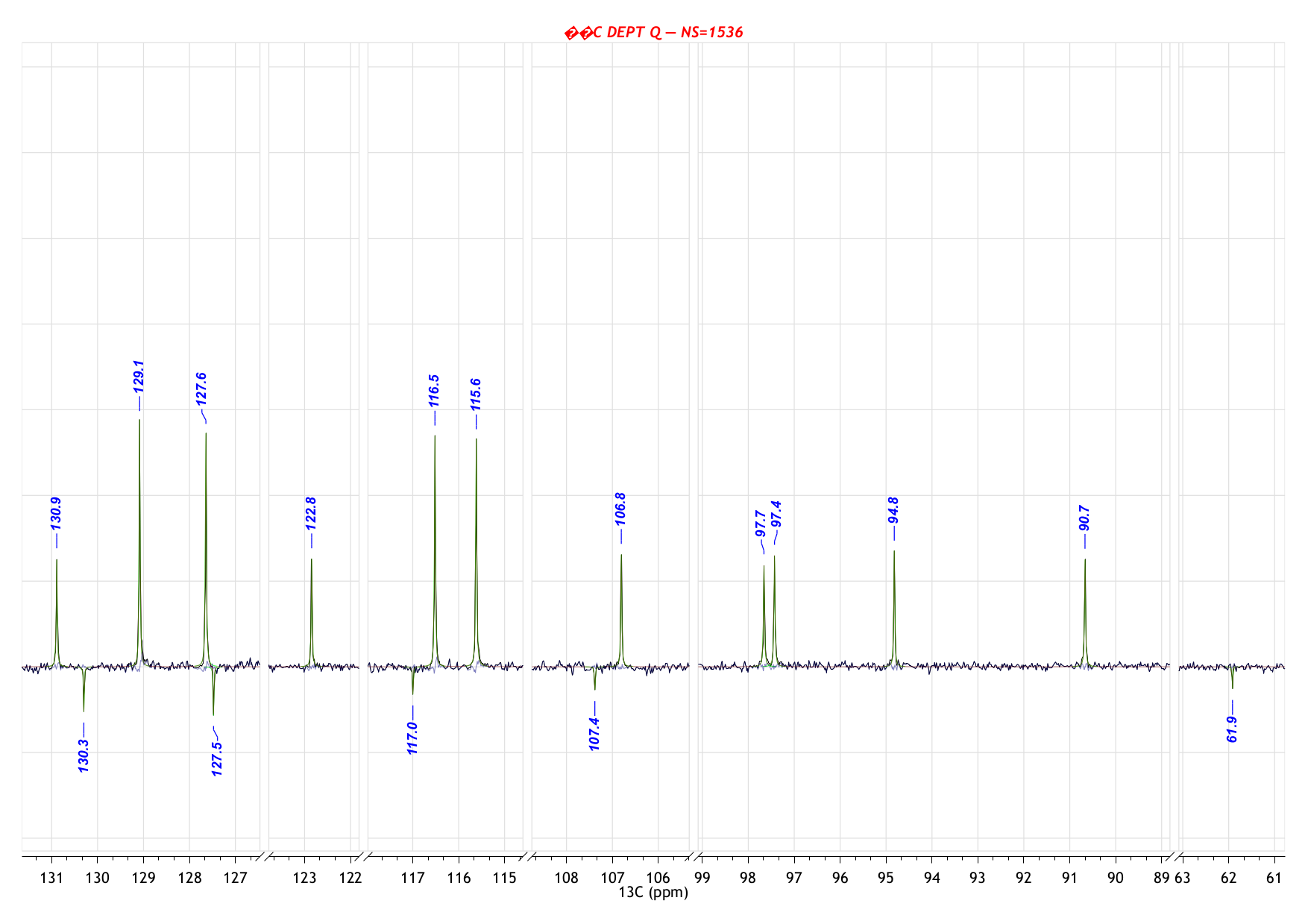


1. ^1^H NMR spectrum of yuccaol B (**48**) (500 MHz, MeOH-*d_4_*, 30 °C).


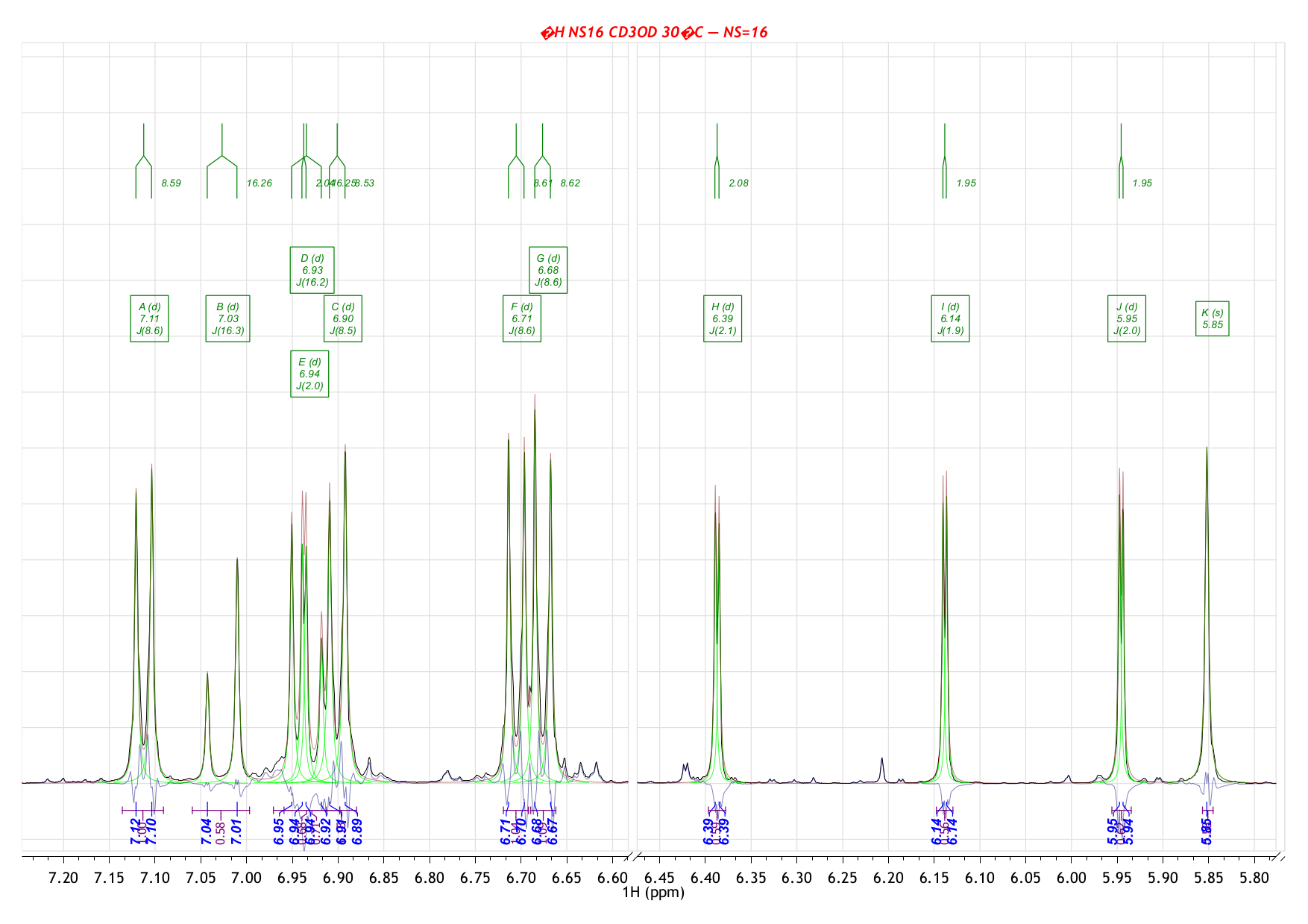


1. ^13^C NMR spectrum of yuccaol B (**48**) (125 MHz, MeOH-*d_4_*, 30 °C).


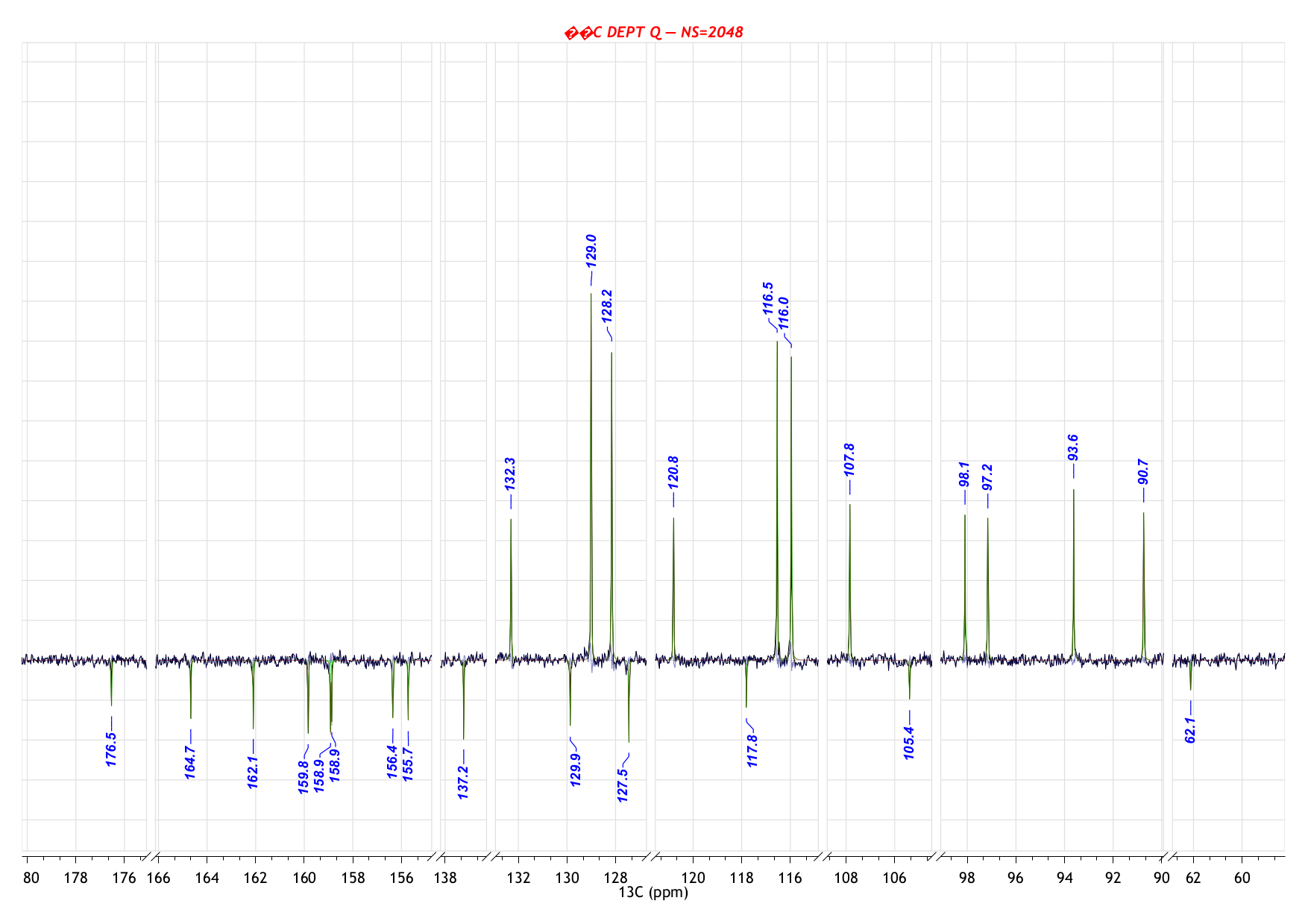


1. ^1^H NMR spectrum of gloriosaol E (**49**) (500 MHz, MeOH-*d_4_*, 30 °C).


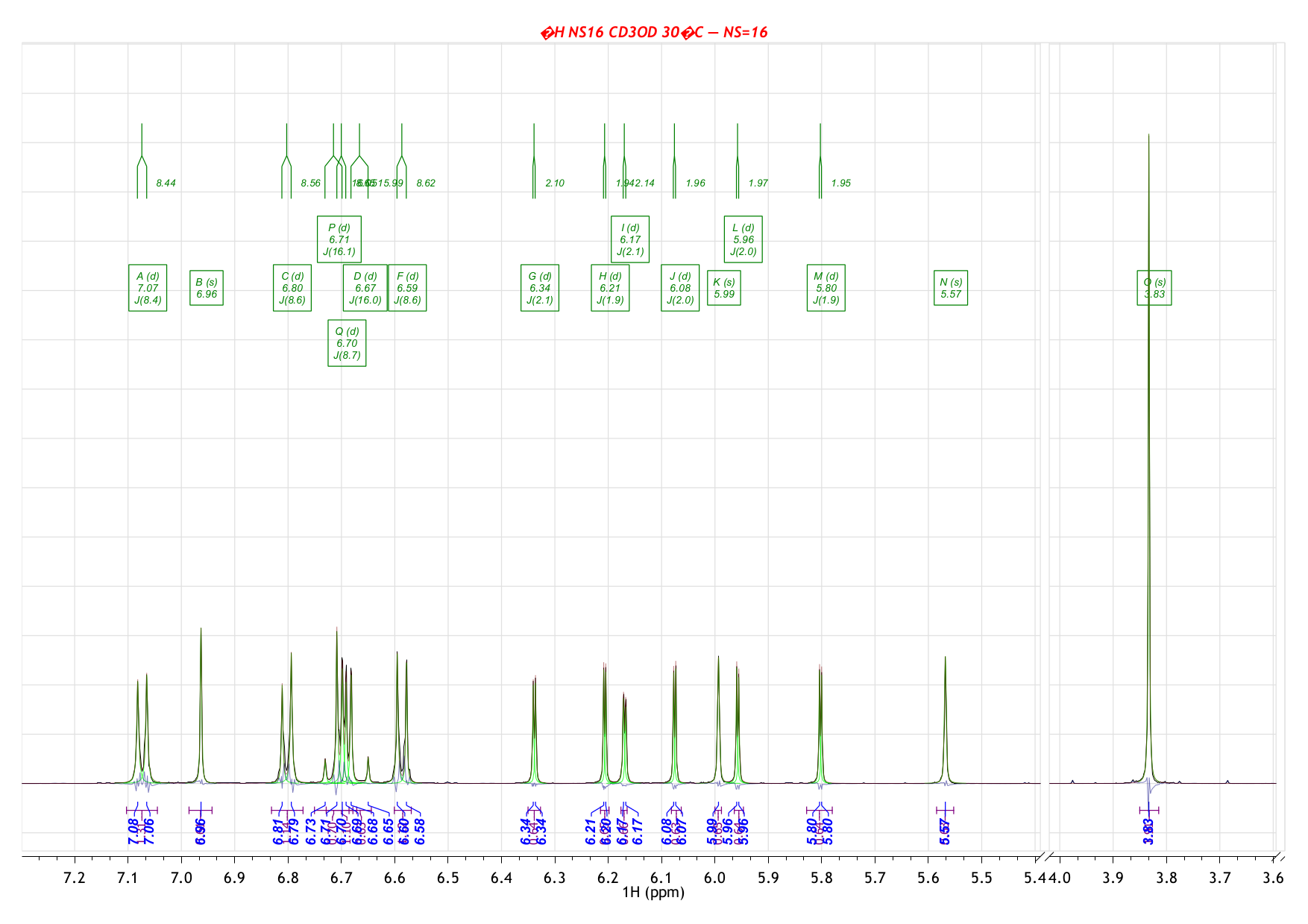


1. ^13^C NMR spectrum of gloriosaol E (**49**) (125 MHz, MeOH-*d_4_*, 30 °C).


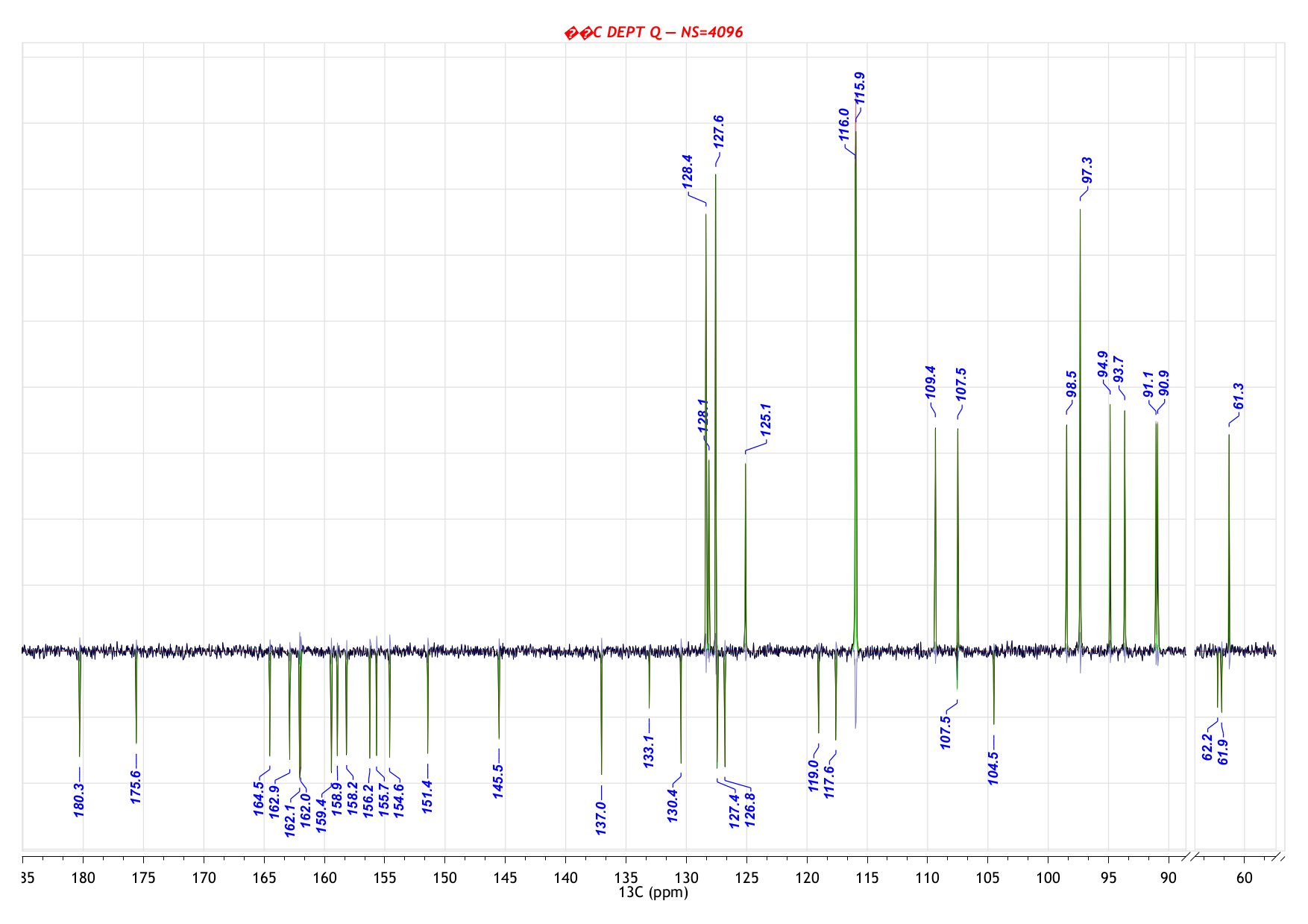


1. ^1^H NMR spectrum of gloriosaol D (**50**) (500 MHz, MeOH-*d_4_*, 30 °C).


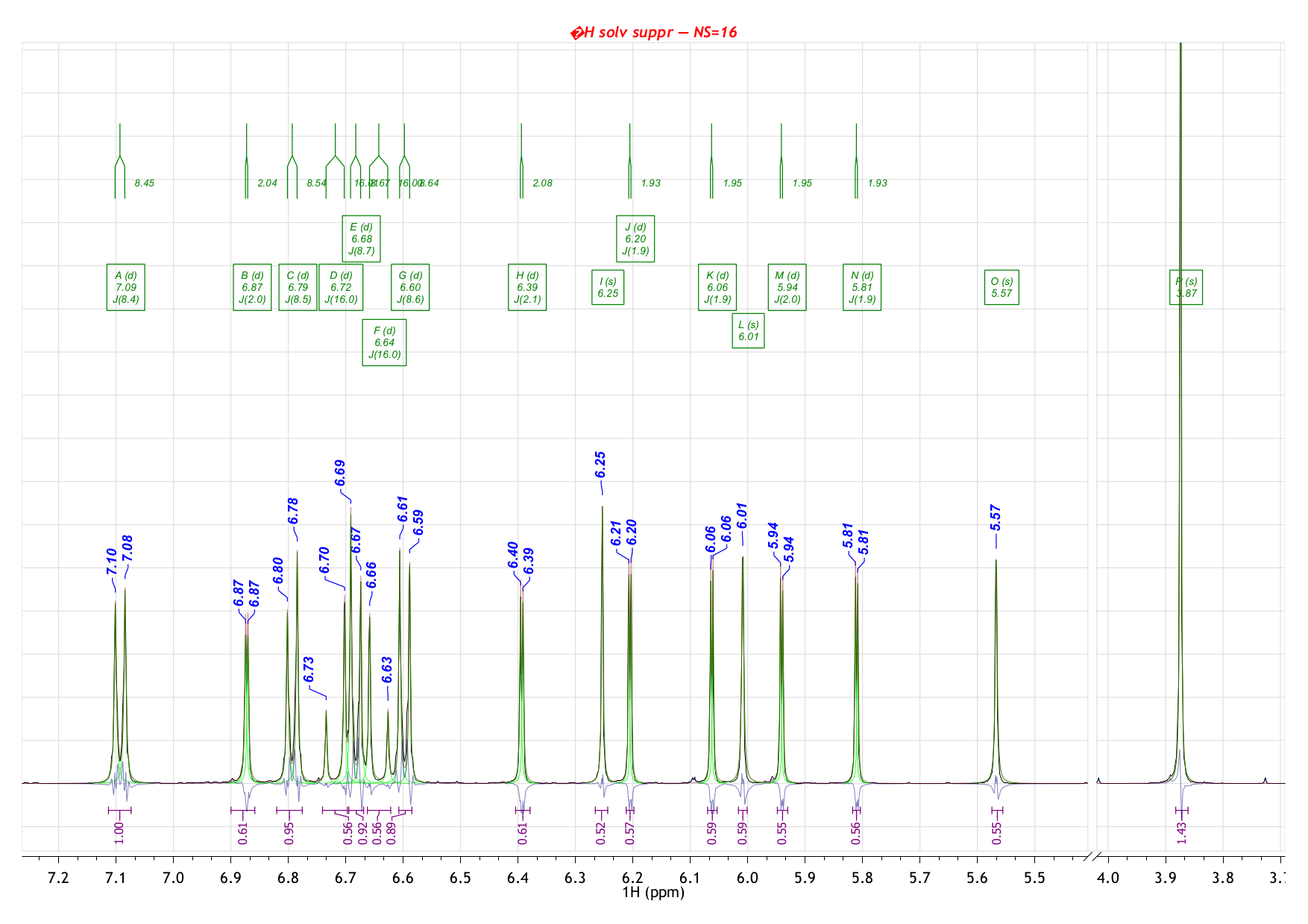


1. ^13^C NMR spectrum of gloriosaol D (**50**) (125 MHz, MeOH-*d_4_*, 30 °C).


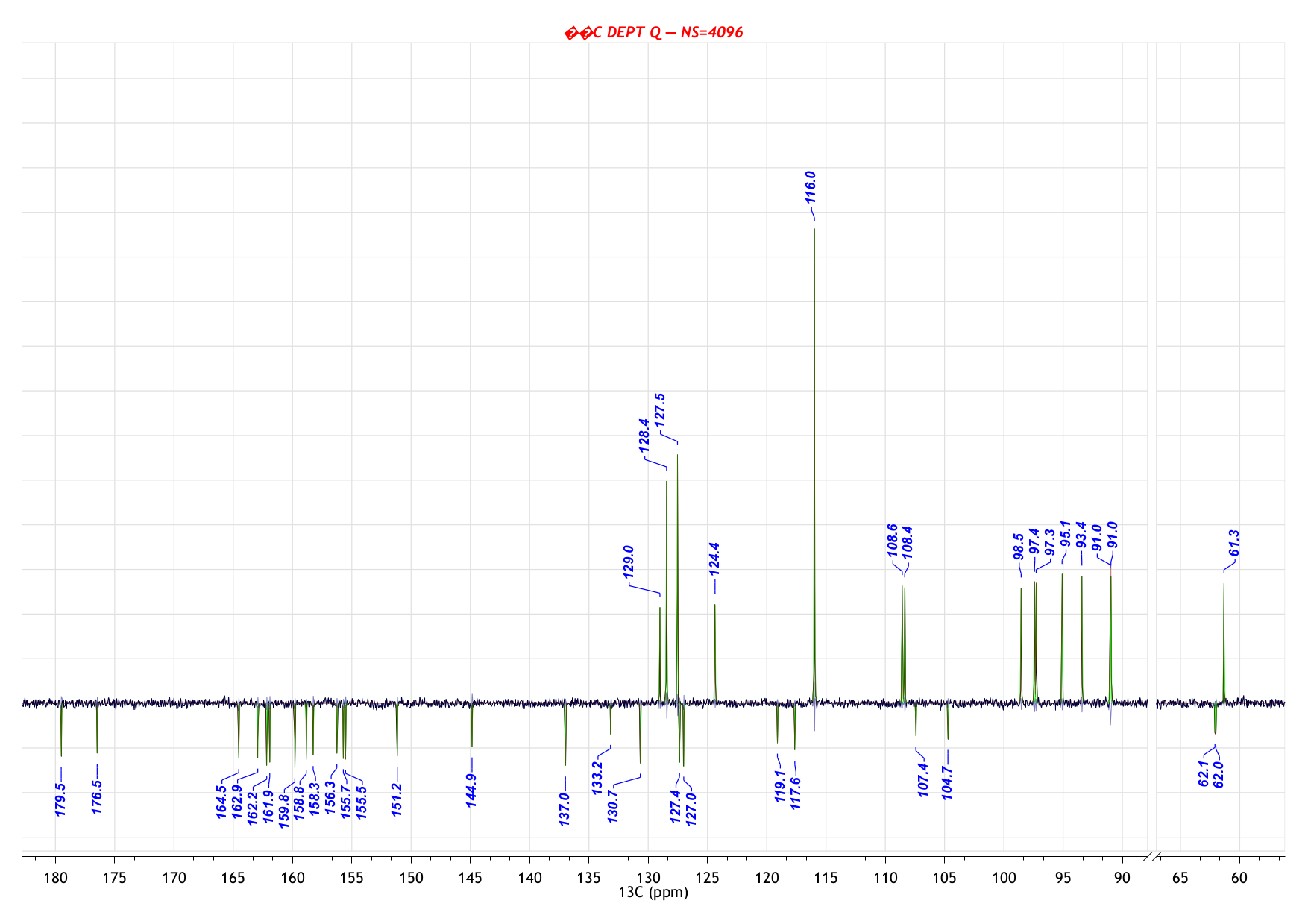


1. ^1^H NMR spectrum of gloriosaol A (**54**) (500 MHz, MeOH-*d_4_*, 30 °C).


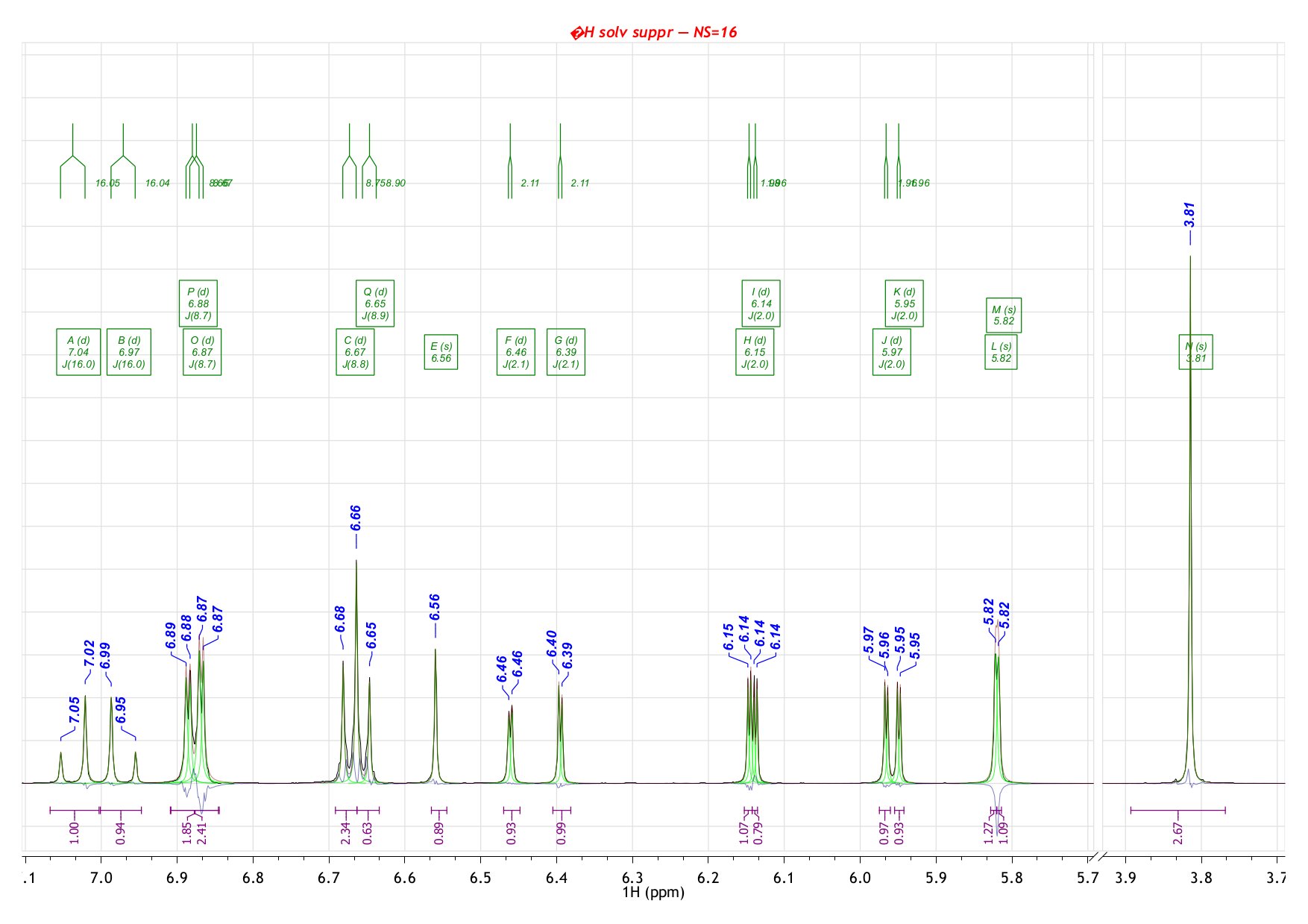


1. ^13^C NMR spectrum of gloriosaol A (**54**) (125 MHz, MeOH-*d_4_*, 30 °C).


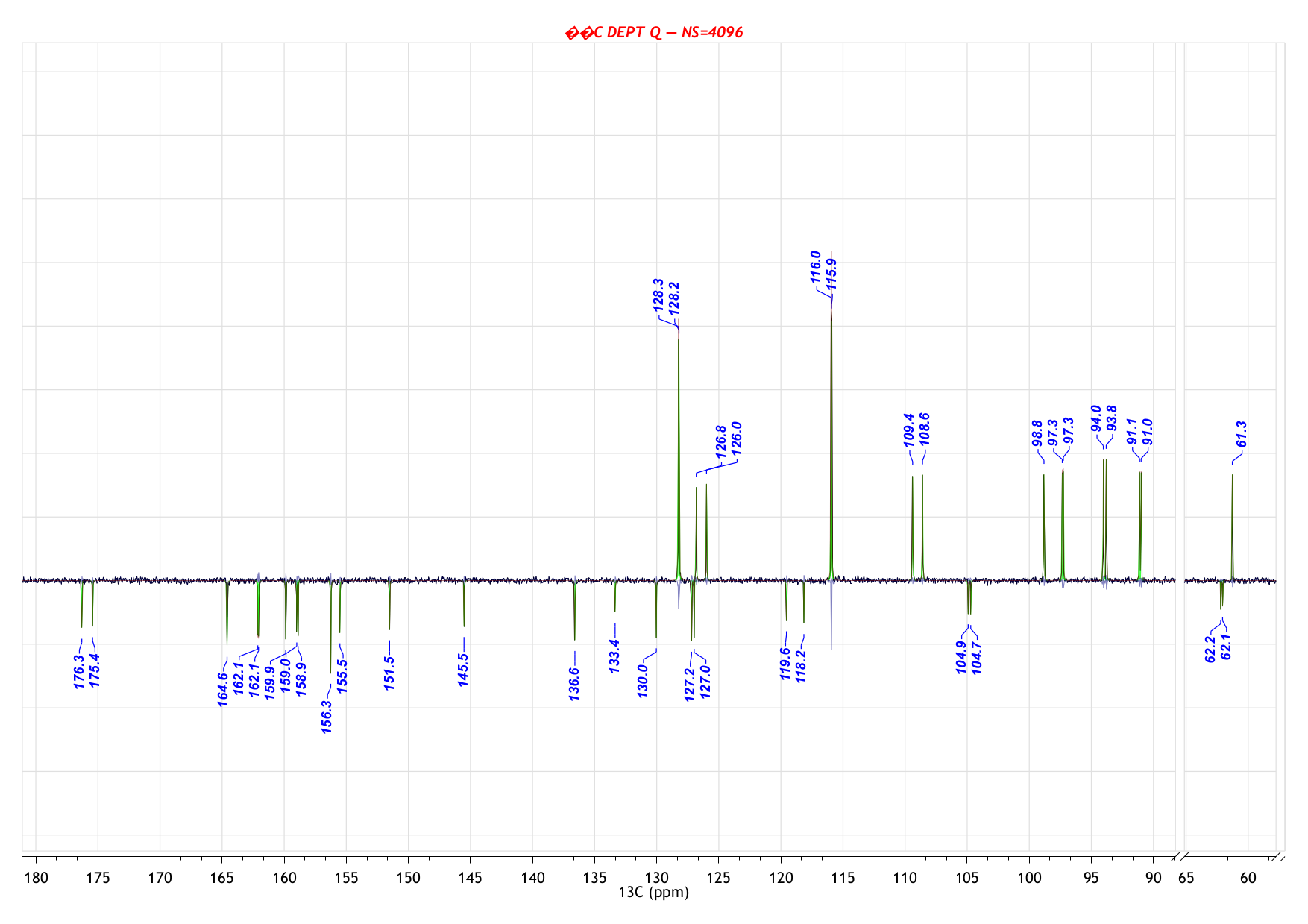


1. ^1^H NMR spectrum of gloriosaol C (**58**) (500 MHz, MeOH-*d_4_*, 30 °C).


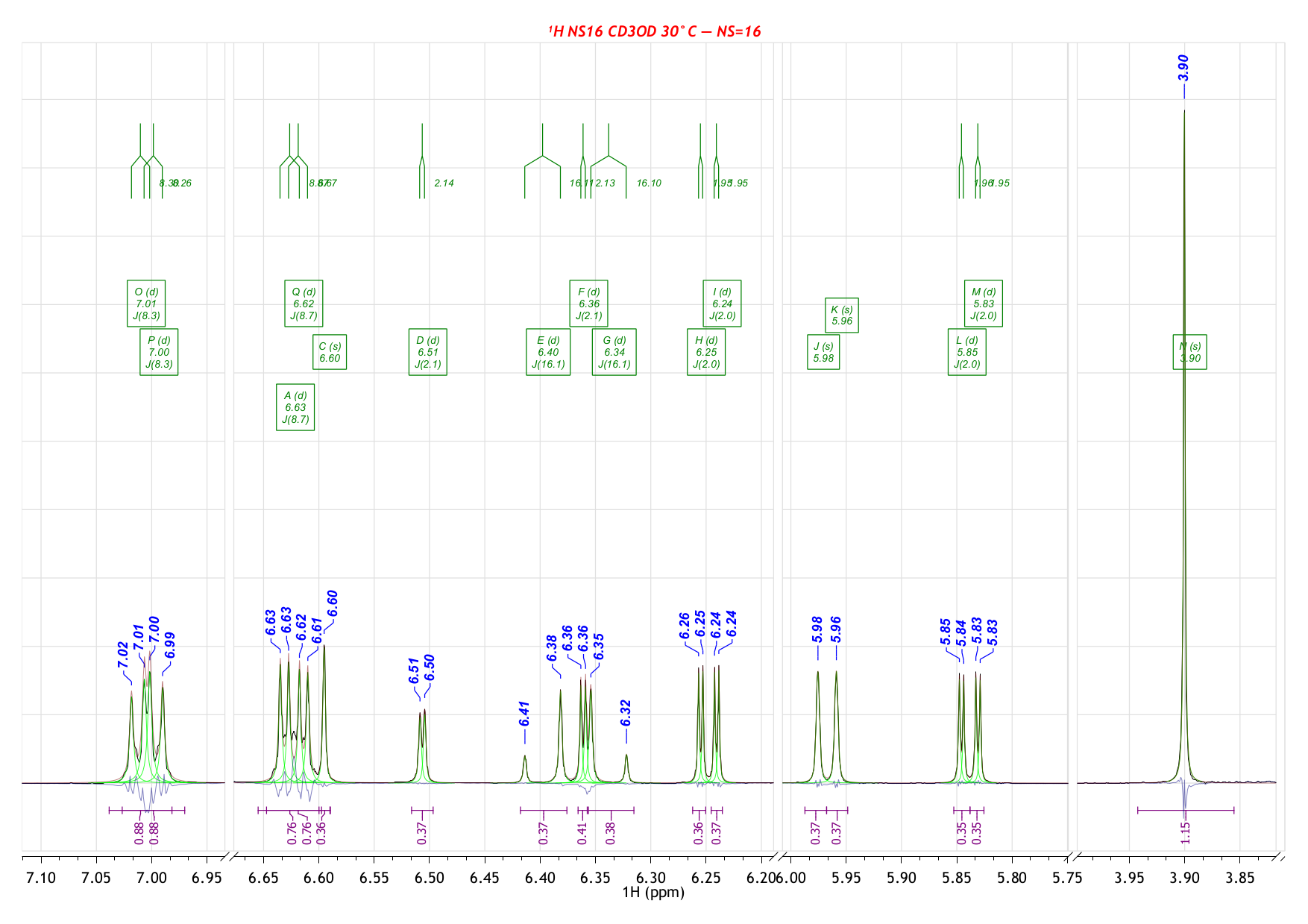


1. ^13^C NMR spectrum of gloriosaol C (**58**) (125 MHz, MeOH-*d_4_*, 30 °C).


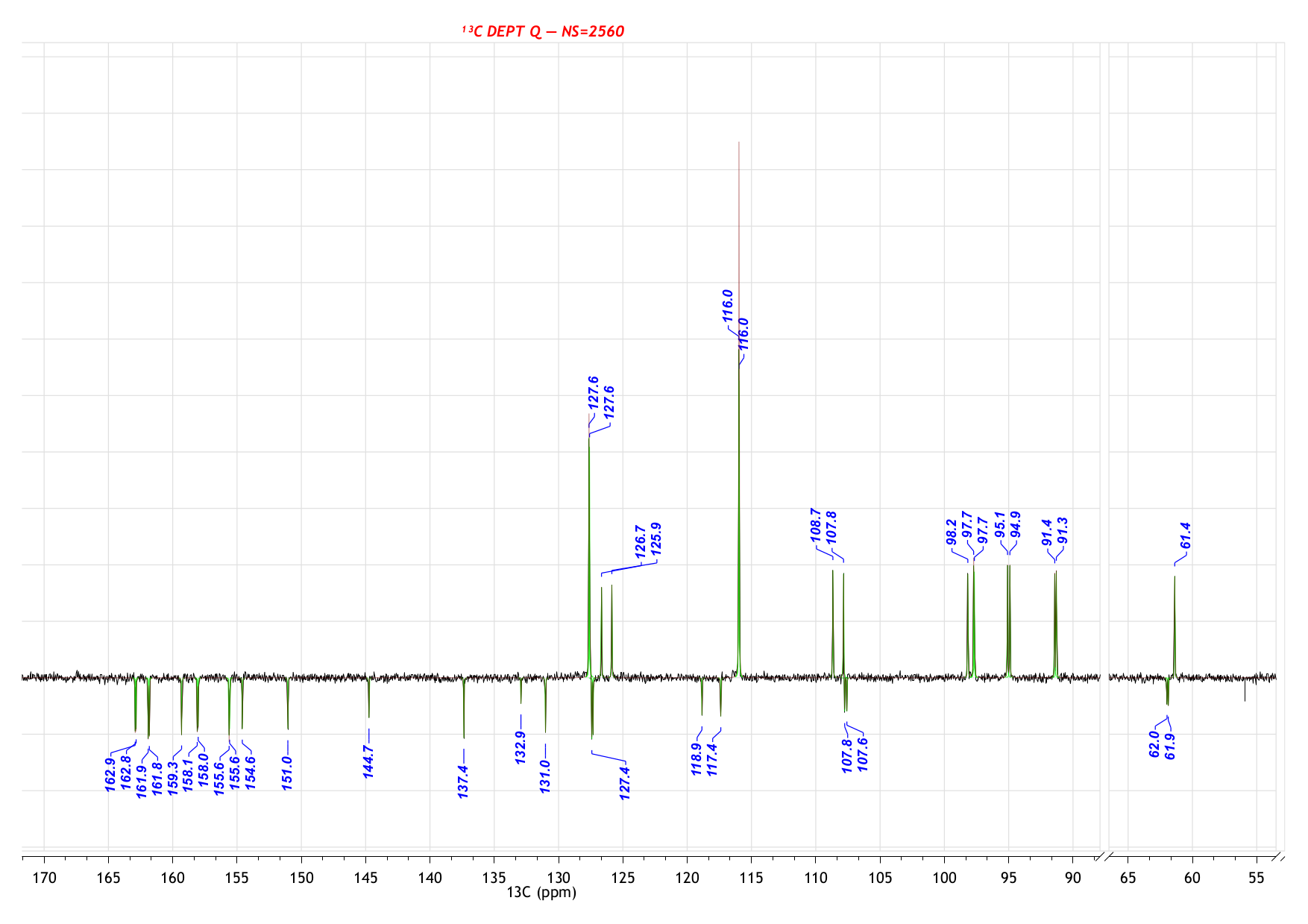

Supplement: Supplementary file 1 [file molecules-24-04162-s001.zip › Supplementary Materials_LPecio_MAlilou_v20191024.docx]
