# Supplementary figures and images for: Yuccalechins A–C from the Yucca schidigera Roezl ex Ortgies Bark: Elucidation of the Relative and Absolute Configurations of Three New Spirobiflavonoids and Their Cholinesterase Inhibitory Activities
Source: Molecules. 2019 Nov 16;24(22):4162. doi: 10.3390/molecules24224162 (PMC6891570; doi:10.3390/molecules24224162)

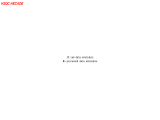

Supplement: Supplementary file 1 [file molecules-24-04162-s001.zip › yucca_schidigera_NMR_data/minus-epicatechin/HSQC-HECADE/pdata/1/thumb.png]
